# Catalytic 1,4-Rhodium(III) Migration Enables 1,3-Enynes to Function as One-Carbon Oxidative Annulation Partners in C–H Functionalizations**

**DOI:** 10.1002/anie.201406072

**Published:** 2014-07-22

**Authors:** David J Burns, Hon Wai Lam

**Affiliations:** School of Chemistry, University of Nottingham, University ParkNottingham, NG7 2RD (UK)

**Keywords:** catalysis, C–H functionalization, enyne, oxidation, rhodium

## Abstract

1,3-Enynes containing allylic hydrogens cis to the alkyne are shown to act as one-carbon partners, rather than two-carbon partners, in various rhodium-catalyzed oxidative annulations. The mechanism of these unexpected transformations is proposed to occur through double C–H activation, involving a hitherto rare example of the 1,4-migration of a Rh^III^ species. This phenomenon is general across a variety of substrates, and provides a diverse range of heterocyclic products.

The metal-catalyzed, directing-group-promoted oxidative C–H functionalization[1] of aromatic C
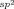
–H bonds with alkynes[2], [3] has been widely exploited to prepare a rich variety of heterocyclic[4] and carbocyclic products.[5] In the reactions of unsymmetrical alkynes, high regioselectivity is usually observed when the two substituents on the alkyne are electronically well-differentiated. For example, with alkynes containing one alkyl and one aryl substituent, the initial C–C bond formation usually occurs with high regioselectivity at the alkyne carbon bearing the sp^3^-hybridized group. This regioselectivity is maintained in the oxidative annulation of 1,3-enynes, as demonstrated by the groups of Fagnou (Scheme 1 a)[6a] and Ackermann,[6b] for example. Herein, we describe a new mode of oxidative annulation, in which 1,3-enynes are able to function as one-carbon,[7] rather than two-carbon reaction partners (Scheme 1 b). We propose this reactivity arises from a hitherto rare example of 1,4-Rh^III^ migration, which opens up new possibilities in C–H functionalization reactions.[8] This phenomenon is general for substrates containing directing groups such as enols, phenols, carboxylic acids, or imides, resulting in a range of heterocyclic products.

**Scheme 1:**
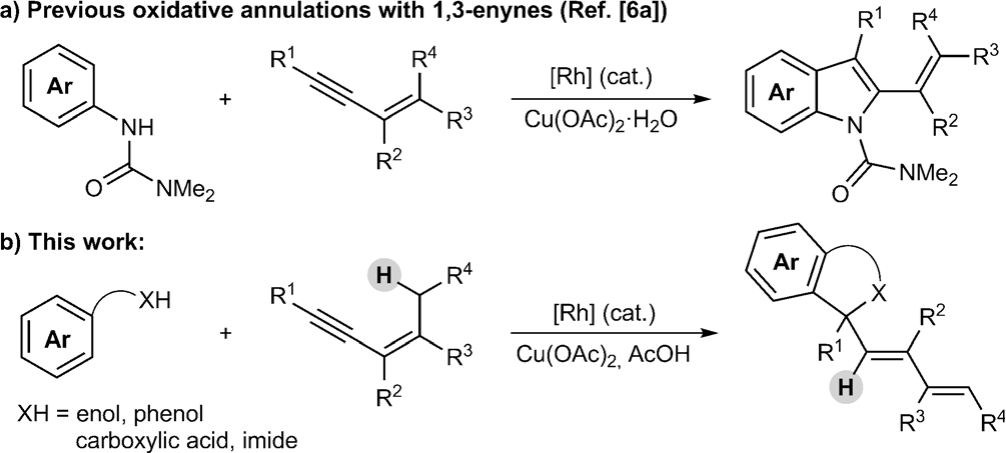
Oxidative annulation reactions of 1,3-enynes.

During our investigations into ruthenium-, rhodium-, and palladium-catalyzed oxidative annulations of 2-aryl cyclic 1,3-dicarbonyl compounds with alkynes,[5f,[g the reaction of substrate **1 a** with 1,3-enyne **2 a** in the presence of [{Cp*RhCl_2_}_2_] (2.5 mol %) and Cu(OAc)_2_ (2.1 equiv) in dioxane at 120 °C was conducted [Eq. (1)]. Surprisingly, in addition to providing the expected spiroindene **3 a** in 21 % yield, this reaction also gave benzopyran **4 a** in 25 % yield.[9]

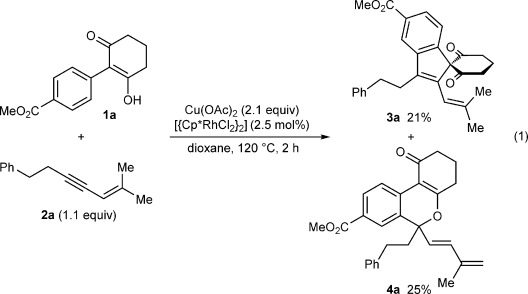


A possible mechanism for the formation of **4 a** is shown in Scheme 2. Generation of the rhodium diacetate complex **5** from [{Cp*RhCl_2_}_2_] and Cu(OAc)_2_ is followed by cyclorhodation of substrate **1 a** to provide the rhodacycle **6**. Coordination and migratory insertion of the 1,3-enyne **2 a** with the regioselectivity observed previously[6] can then provide a new rhodacycle **7**. Reductive elimination of **7** would then give the expected spiroindene **3 a** as described with alkynes.[5f,[g] However, an alternative pathway is the reversible protonolysis of **7** with AcOH to provide the alkenylrhodium species **8**, which can then undergo a 1,4-rhodium migration to give a new allylrhodium species **9 A**.[10] Notably, this process enables the activation of a C
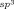
–H bond. The 1,4-migration of rhodium(I) is well-known,[11]–[13] but the corresponding 1,4-migrations of rhodium(III) are rare, with the only reports to date being stoichiometric studies of alkenyl to aryl migrations described by Ishii and co-workers.[8] Presumably, the σ-allylrhodium species **9 A** can interconvert with the π-allylrhodium species **9 B** through the intermediacy of other isomers (not shown). Nucleophilic attack of the π-allylrhodium(III) moiety[14], [15] of **9 B** by the enol oxygen would provide the benzopyran **4 a** and the rhodium(I) species **10**, which can be then be reoxidized to **5** by Cu(OAc)_2_.

**Scheme 2:**
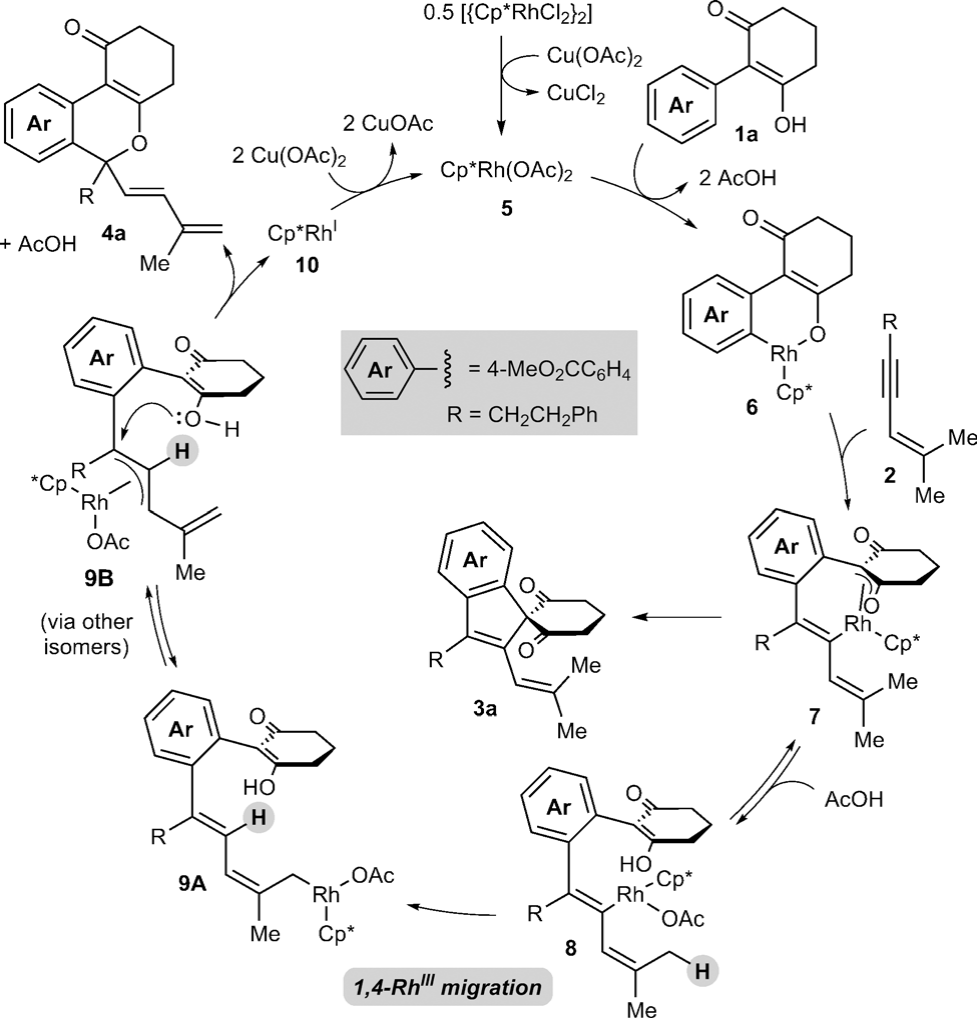
Proposed catalytic cycle.

A survey of reaction conditions[16] revealed that lowering the temperature to 60 °C led to higher yields of benzopyran **4 a**, but did not significantly alter the yield of spiroindene **3 a**. Furthermore, the addition of AcOH (0.1 equiv) led to more consistently reproducible results. Under these conditions, benzopyran **4 a** and spiroindene **3 a** were obtained in 86 % and 12 % yield, respectively (Table 1, entry 1).

**Table 1:** Oxidative annulation reactions of various 2-aryl-3-hydroxy-2-cyclohexenones with 1,3-enyne 2 a.[a] 
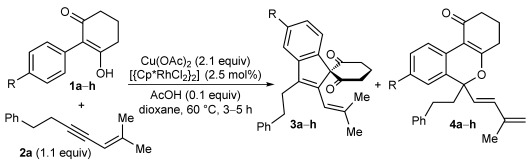

Entry	Substrate	Yield of3[%][b]	Yield of4[%][b]
1	**1 a** R=CO_2_Me	12	86
2	**1 b** R=H	60	20
3	**1 c** R=OMe	64	17
4	**1 d** R=F	26	44
5	**1 e** R=CF_3_	<5[c]	78
6	**1 f** R=COMe	<5[c]	65
7	**1 g** R=NO_2_	n.d.[d]	82
8	**1 h** R=SO_2_Me	<5[c]	84

[a] Reactions were conducted with 0.50 mmol of **1 a**–**h**.

[b] Yield of isolated products.

[c] The spiroindene was detected in trace amounts.

[d] Not detected.

The scope of this transformation with respect to the 2-aryl-3-hydroxy-2-cyclohexenone was then explored (Table 1). With substrates **1 b** and **1 c**, which contain phenyl or 4-methoxyphenyl groups, respectively, the spiroindenes **3** were the major products (Table 1, entries 2 and 3). With substrates containing more electron-withdrawing substituents at the 4-position of the aromatic ring, the benzopyran became the major product (Table 1, entries 4–8). The spiroindene was formed in only trace amounts in the reactions of substrates containing trifluoromethyl, acetyl, or sulfone substituents (Table 1, entries 5, 6, and 8), and was not detected when a nitro group was present (Table 1, entry 7). These observations can be rationalized by considering that spiroindene formation requires the reductive elimination of Rh^III^ from intermediates analogous to rhodacycle **7** (Scheme 2), with concomitant oxidation of the substrate. Therefore, it appears reasonable to assume that the activation barrier of this reductive elimination is increased with more electron-deficient substrates, as the substrate is more difficult to oxidize. The alternative pathway leading to the benzopyran **4** then becomes more competitive.

Next, the scope of this process with respect to the 1,3-enyne was investigated using substrate **1 g**, and various enynes containing allylic hydrogens *cis* to the alkyne were shown to be effective one-carbon oxidative annulation partners (Table 2). None of the alternative spiroindenes were detected in any of these reactions. 1,3-Enynes containing protected or unprotected 2-hydroxyethyl groups were tolerated (Table 2, entries 1 and 2). 1,3-Enynes **2 d** and **2 e**, which contain a phenyl group or a hydrogen atom *trans* to the alkyne, also reacted smoothly to provide benzopyrans **11 d** and **11 e** (Table 2, entries 3 and 4). The reaction is not limited to 1,3-enynes containing methyl substitution *cis* to the alkyne, as shown by the successful annulations of 1,3-enynes **2 f** and **2 g** (Table 2, entries 5 and 6). Notably, a silyl-protected hydroxymethyl substituent at the *trans-*position of 1,3-enyne **2 h** led to **11 h** in 61 % yield with >95:5 *E*:*Z* selectivity at the enol silane (Table 2, entry 7).[17] Finally, 1,3-enyne **2 h**, which contains a methyl group at the alkenyl carbon proximal to the alkyne was also effective, providing **11 i** with >95:5 *E*:*Z* selectivity (Table 2, entry 8).[17]

**Table 2:** Oxidative annulation reactions of 1 g with various 1,3-enynes.[a] 
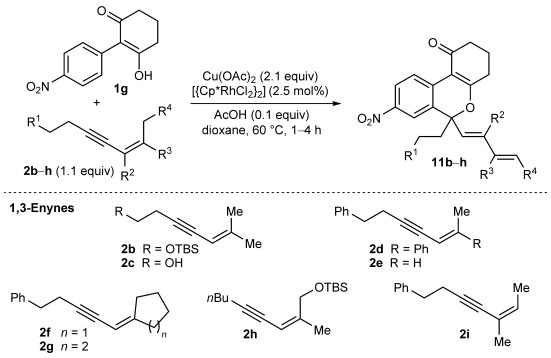

Entry	1,3-Enyne	Product		Yield [%][b]
1 2[c]	**2 b 2 c**	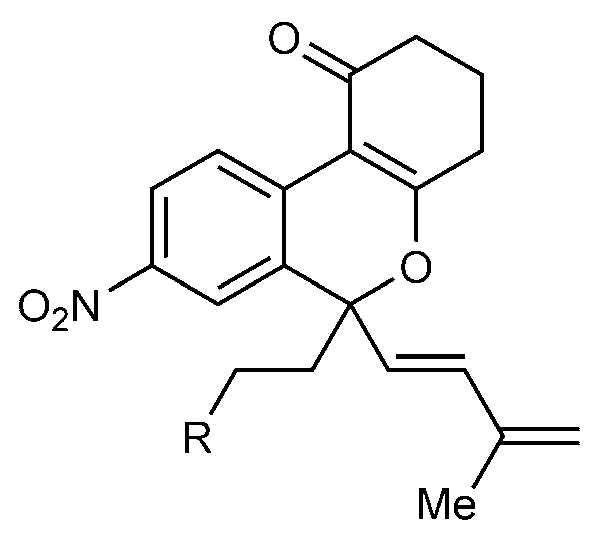	**11 b** R=OTBS **11 c** R=OH	84 62
3 4	**2 d 2 e**	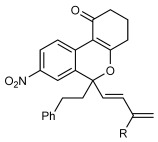	**11 d** R=Ph **11 e** R=H	73 64
5[d] 6	**2 f 2 g**	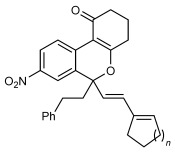	**11 f**­ *n*=1 **11 g**­ *n*=2	93 89
7	**2 h**	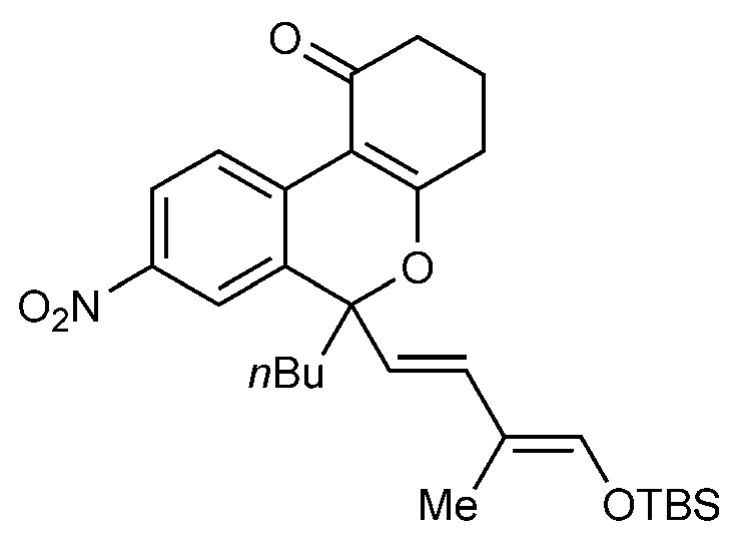	**11 h**	61
8	**2 i**	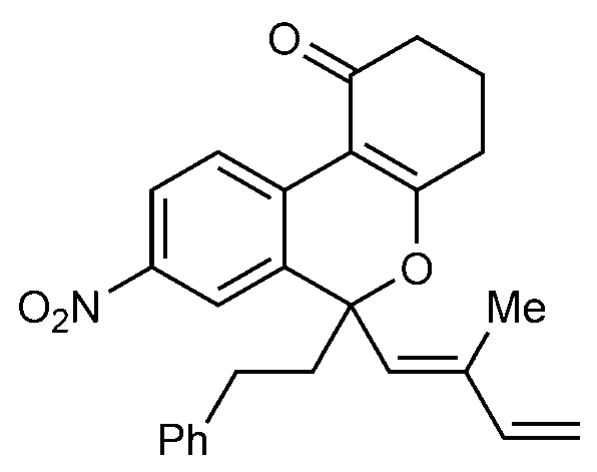	**11 i**	61

[a] Reactions were conducted with 0.50 mmol of **1 g**.

[b] Yield of isolated products.

[c] Reaction conducted at 90 °C.

[d] Reaction conducted using 0.37 mmol of **1 g**.

This unusual oxidative annulation was found to be a general phenomenon, and not merely limited to 2-aryl-3-hydroxy-2-cyclohexenones. Several other aromatic substrates containing enol, phenol, carboxylic acid, or imide directing groups underwent oxidative annulation with 1,3-enyne **2 a** to give a diverse range of five- or six-membered oxygen and nitrogen heterocycles **13 a**–**e** (Scheme 3).[18]

**Scheme 3:**
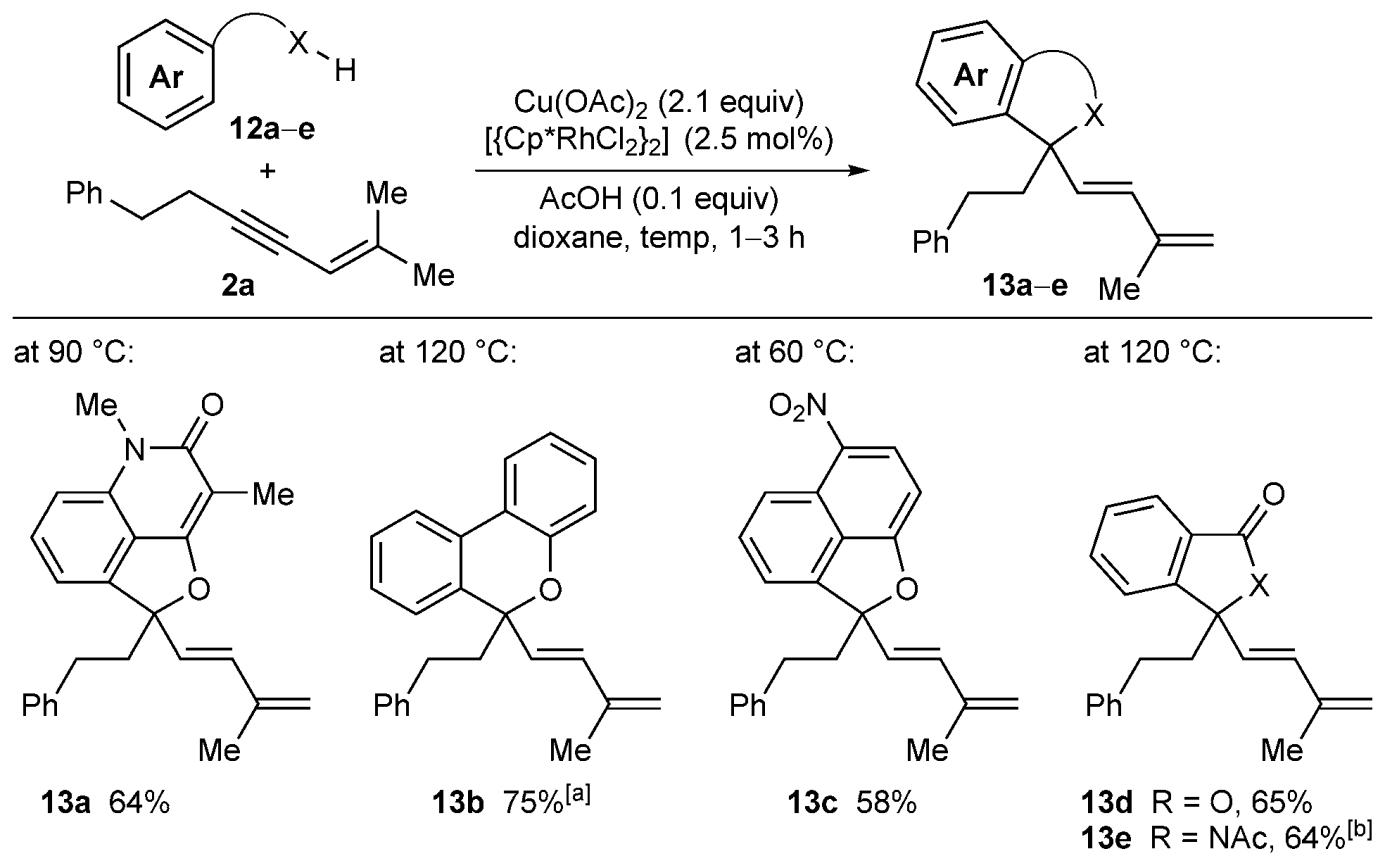
Oxidative annulation reactions of various substrates with 1,3-enyne 2 a. Yields are of isolated products. [a] Reaction conducted in the presence of K_2_CO_3_ (3.0 equiv), and a second portion of [{Cp*RhCl_2_}_2_] (2.5 mol %) was added after 1 h. [b] Using 5 mol % of [{Cp*RhCl_2_}_2_].

To verify the structural requirements of the 1,3-enyne for one-carbon annulation to occur, the reaction of **1 g** with enyne **14**, in which there are no allylic hydrogens *cis* to the alkyne, was performed. This reaction led to no conversion at the standard temperature of 60 °C. However, increasing the temperature to 90 °C gave the spiroindene **15** in 53 % yield and only 7 % of benzopyran **11 e** [Eq. (2)]. This experiment contrasts with that shown in Table 2, entry 4, in which the corresponding (*Z*)-1,3-enyne **2 e** gave benzopyran **11 e** only. These results suggest that 1,4-Rh^III^ migration (**8** to **9** in Scheme 2) occurs by a direct pathway that is contingent upon the close proximity of Rh with the *cis*-allylic hydrogens. We postulate that the formation of benzopyran **11 e** in 7 % yield in Equation (2) results from some type of *E*/*Z* isomerization occurring at the higher temperature of 90 °C.

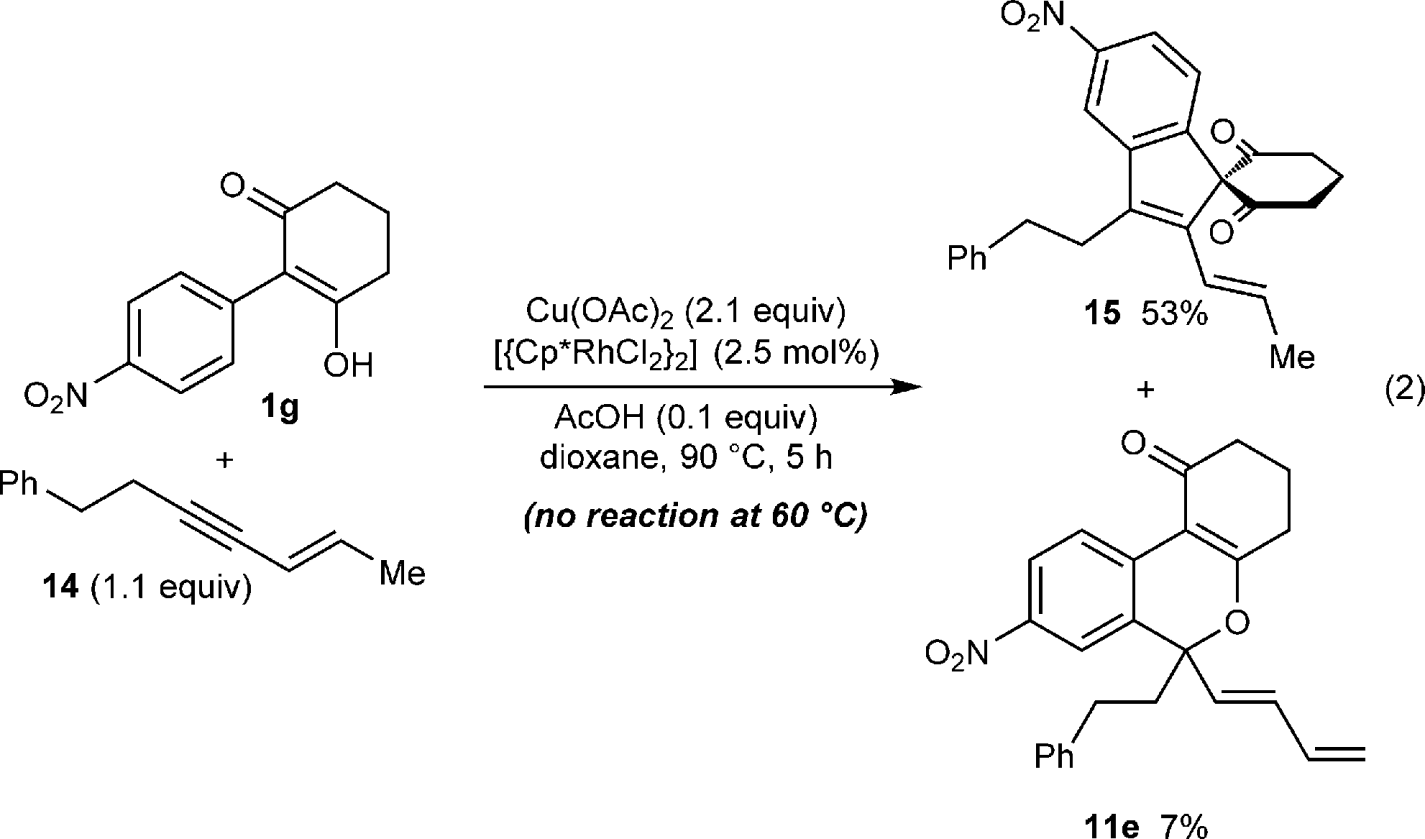


To gain further insight into this process, the reaction of **1 g** with the hexadeuterated 1,3-enyne [D]_6_-**2 a** was conducted [Eq. (3)]. Three compounds were isolated from this experiment: recovered [D]_6_-**2 a** in 20 % yield with no deuterium depletion detected, spiroindene [D]_6_-**3 g** in 19 % yield with no deuterium depletion detected, and benzopyran [D]_*n*_-**4 g** in 65 % yield, with incomplete deuteration (77 % D) at the alkenyl carbon adjacent to the quaternary center. Several conclusions can be drawn from these results.

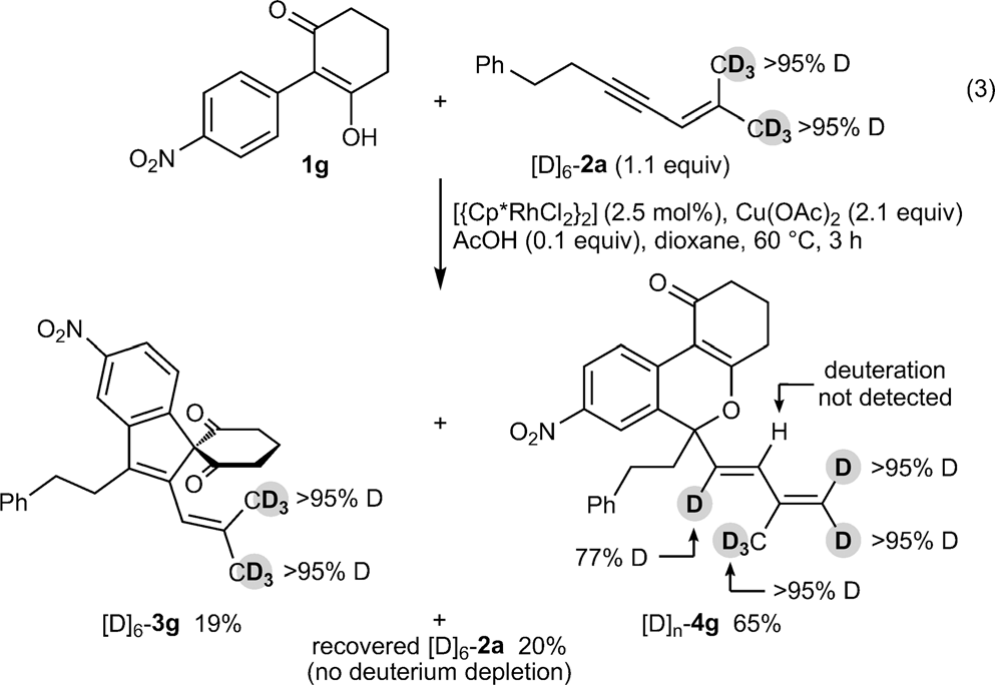


First, the formation of spiroindene [D]_6_-**3 g** suggests that C–H functionalization at the methyl group *cis* to the alkyne in the 1,3-enyne is involved in the product-determining step, since the reaction of **1 g** with the non-deuterated 1,3-enyne **2 a** led to none of the spiroindene **3 g** being detected (Table 1, entry 7). Second, the deuteration pattern in [D]_*n*_-**4 g** is consistent with the 1,4-Rh^III^ migration mechanism shown in Scheme 2. However, the incomplete deuteration (77 % D) at the internal alkene suggests that 1,4-Rh^III^ migration may occur by an acetate-assisted, concerted metalation–deprotonation of **16** to form rhodacycle **17**, followed by deuteronolysis with AcOD (Scheme 4).[19] Incomplete deuteration would arise as a result of competitive protonolysis of **17** with the AcOH that is also present in the reaction, or by adventitious water. Further support for this mechanism was provided by the reaction of **1 g** with 1,3-enyne **2 a** in a dioxane/D_2_O (5:1) mixture, which provided [D]_*n*_-**4 g** with partial deuteration (28 % D) at the alkenyl carbon, with no deuteration observed at any other site [Eq. (4)].[20]

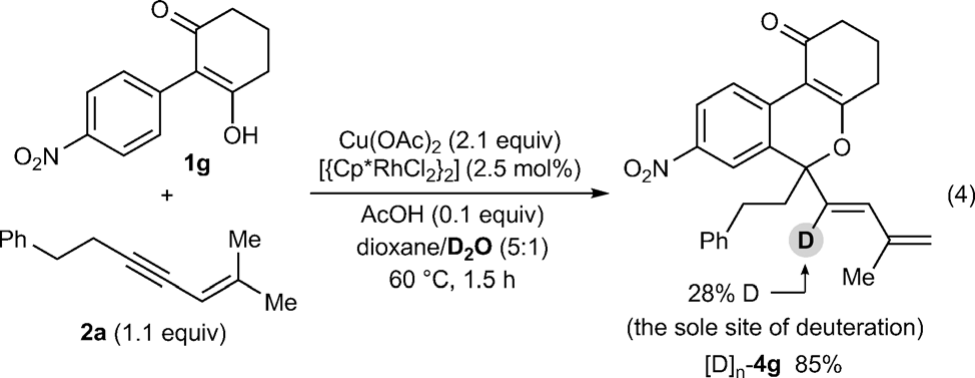


**Scheme 4:**
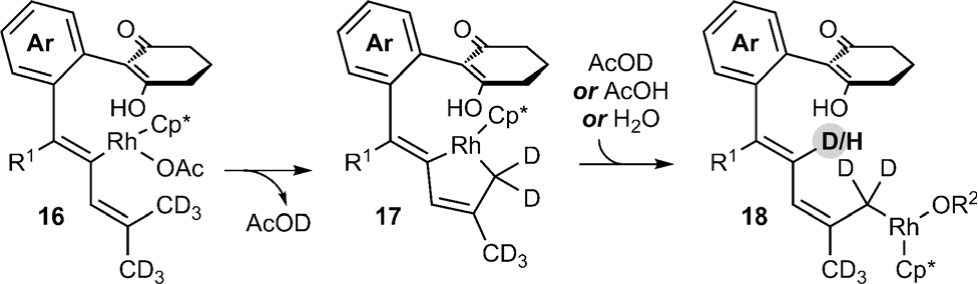
Possible mechanism of the 1,4-Rh^III^ migration.

In conclusion, we have reported an unexpected mode of oxidative annulation in Rh^III^-catalyzed C–H functionalizations when 1,3-enynes containing allylic hydrogens *cis* to the alkyne are present. The mechanism of these reactions is proposed to occur through double C–H activation, including that of a C
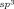
–H bond, involving a hitherto rare example of the 1,4-migration of a Rh^III^ species. Of broader significance, the generation of an allyl–metal species from sequential C–H functionalization–1,4-metal migration opens up new opportunities in synthesis, and exploitation of this pathway in other transformations is underway in our laboratories.
