# Supplementary material for: Catalytic 1,4-Rhodium(III) Migration Enables 1,3-Enynes to Function as One-Carbon Oxidative Annulation Partners in C–H Functionalizations
Source: Angew Chem Int Ed Engl. 2014 Jul 22;53(37):9931–5. doi: 10.1002/anie.201406072 (PMC4464529; doi:10.1002/anie.201406072)
Supplement: Supplementary file 1 [file anie0053-9931-sd1.pdf]

Angewandte Chemie
Eine Zeitschrift der Gesellschaft Deutscher Chemiker

Supporting Information

© Wiley-VCH 2014

69451 Weinheim, Germany

# Catalytic 1,4-Rhodium(III) Migration Enables 1,3-Enynes to Function as One-Carbon Oxidative Annulation Partners in C–H Functionalizations**

*David J. Burns and Hon Wai Lam**

anie_201406072_sm_miscellaneous_information.pdf

**Contents**

1. General Information…..................................................................................................... 2
2. Synthesis of Substrates..................................................................................................... 3
3. One-Carbon Oxidative Annulation Reactions of 1,3-Enynes................................................ 11
4. Deuterium Labeling Experiments........................................................................................ 29
5. NMR Spectra........................................................................................................................ 33

# 1. General Information

Unless specified otherwise, all reactions were carried out under an atmosphere of nitrogen using oven-dried glassware. Unless specified otherwise, all other commercially available reagents and solvents were used as received. Dioxane used for oxidative annulation reactions was non-anhydrous. THF was dried and purified by passage through activated alumina columns using a solvent purification system. All petroleum ether used was 40–60 °C petroleum ether. Thin layer chromatography (TLC) was performed on Merck DF-Alufoilien $60F_{254}$ 0.2 mm precoated plates. Compounds were visualized by exposure to UV light or by dipping the plates into solutions of potassium permanganate or vanillin followed by heating. Flash column chromatography was carried out using silica gel (Fisher Scientific 60 Å particle size 35–70 micron). Melting points were recorded on a Gallenkamp melting point apparatus and are uncorrected. The solvent of recrystallization is reported in parentheses. Infra-red spectra were recorded on a Nicolet Avatar 360 FT instrument on the neat compound using the attenuated total refraction technique. NMR spectra were acquired on Bruker AV400, AV(III)400, or DPX400 spectrometers. $^{1}H$ and $^{13}C$ NMR spectra were referenced to external tetramethylsilane *via* the residual protonated solvent ($^{1}H$) or the solvent itself ($^{13}C$). All chemical shifts are reported in parts per million (ppm). For $CDCl_3$, the shifts are referenced to 7.27 ppm for $^{1}H$ NMR spectroscopy and 77.0 ppm for $^{13}C$ NMR spectroscopy. For $(CD_3)_2CO$, the shifts are referenced to 2.05 ppm for $^{1}H$ NMR spectroscopy and 29.84 ppm for $^{13}C$ NMR spectroscopy. For $(CD_3)_2SO$, the shifts are referenced to 2.50 ppm for $^{1}H$ NMR spectroscopy and 39.52 ppm for $^{13}C$ NMR spectroscopy. All $^{19}F$ NMR spectra were proton decoupled. High-resolution mass spectra were recorded using electrospray ionization (ESI) techniques at the School of Chemistry, University of Nottingham.

## 2. Synthesis of Substrates

### Preparation of 2-Aryl-3-hydroxy-2-cyclohexenones

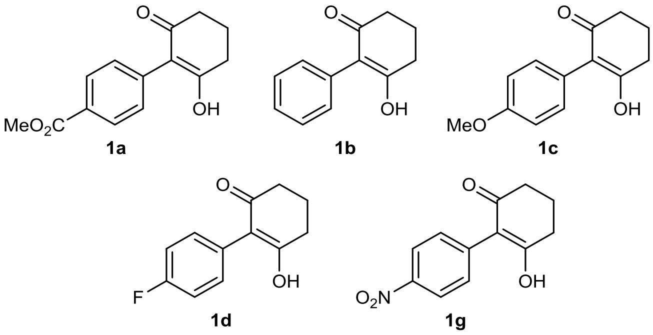


Substrates **1a**,[1] **1b**,[1] **1c**,[1] **1d**,[2] and **1g**[3] were prepared according to literature procedures.

### General Procedure A: Preparation of 2-Aryl-3-hydroxy-2-cyclohexenones

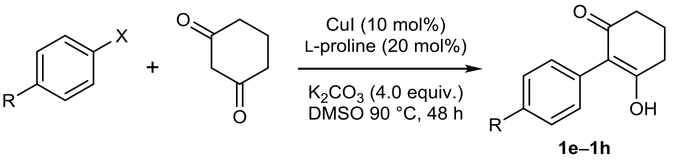


To a stirred solution of CuI (10 mol%), L-proline (20 mol%), $K_2CO_3$ (4.0 equiv), and 1,3-cyclohexanedione (3.0 equiv) in anhydrous DMSO was added the appropriate aryl halide (1.0 equiv). The reaction mixture was then stirred at 90 °C for 48 h. The reaction was cooled to 0 °C, acidified with 3.0 M $HCl_{(aq)}$ to pH 3–4 and EtOAc (200 mL) was added. The layers were separated and the aqueous extracted with EtOAc (2 × 100 mL). The combined organic extracts were washed with brine (50 mL), dried (MgSO4), filtered, and concentrated *in vacuo*. Purification of the residue by column chromatography (28:2:1 EtOAc:*i*-PrOH:$H_2O$) gave a mixture of the 2-aryl-3-hydroxy-2-cyclohexenone with 1,3-cyclohexanedione. The mixture was recrystallized from hot acetone and hexane to give the title 2-aryl-3-hydroxy-2-cyclohexenone.

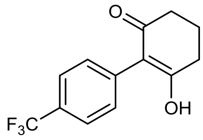


**3-Hydroxy-2-[4-(trifluoromethyl)phenyl]cyclohex-2-en-1-one (1e)**. The title compound was prepared according to General Procedure A using CuI (162 mg, 0.85 mmol), L-proline (196 mg, 1.70 mmol), $K_2CO_3$ (4.70 g, 34.0 mmol), 1,3-cyclohexanedione (2.86 g, 25.5 mmol) and 1-iodo-4-(trifluoromethyl)benzene (1.08 mL, 8.51 mmol) in anhydrous DMSO (25 mL) to give a white crystalline solid (630 mg, 29%). $R_f$ 0.38 (14:2:1 EtOAc:*I*-PrOH:$H_2O$); m.p. 241–242 ºC (acetone/petroleum ether); IR 1618 (C=O), 1560,

1. Reddy Chidipudi, S.; Khan, I.; Lam, H. W. *Angew. Chem., Int. Ed.* **2012**, *51*, 12115–12119.
2. Reddy Chidipudi, S.; Wieczysty, M. D.; Khan, I.; Lam, H. W. *Org. Lett.* **2013** *15*, 570–573.
3. Lin, Y.; Wu, C.; Lin, S.; Huang, J.; Sun, Y.; Yang, D. *Bioorg. Med. Chem.* **2002**, 10, 685–690.

1329, 1272, 1155, 1070, 989, 838 cm$^{-1}$; $^{1}$H NMR (400 MHz, $(CD_3)_2SO$) δ 10.99 (1H, br s, O**H**), 7.63 (2H, d, *J* = 8.0 Hz, 2 × Ar**H**), 7.36 (2H, d, *J* = 8.0 Hz, 2 × Ar**H**), 2.59–2.41 (4H, m, C**H**$_2$CH$_2$C**H**$_2$), 2.02–1.87 (m, 2H, CH$_2$C**H**$_2$CH$_2$); $^{13}$C NMR (100.6 MHz, $(CD_3)_2SO$) δ 138.8 (C), 131.6 (2 × CH), 126.3 (q, *J* = 31.5 Hz, C), 124.4 (q, *J* = 271.6 Hz, C), 123.9 (q, *J* = 3.7 Hz, 2 × CH), 115.1 (C), 20.2 (CH$_2$), some signals not observed due to keto–enol tautomerism; $^{19}$F NMR (376 MHz, $CDCl_3$) δ –60.8 (s); HRMS (ESI +ve) Exact mass calculated for $C_{13}H_{12}F_3O_2$ [M+H]$^+$: 257.0784, found: 257.0788.

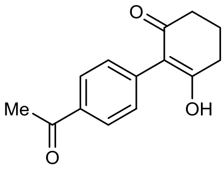


**2-(4-Acetylphenyl)-3-hydroxycyclohex-2-en-1-one (1f)**. The title compound was prepared according to General Procedure A using CuI (162 mg, 0.85 mmol), L-proline (196 mg, 1.70 mmol), $K_2CO_3$ (4.70 g, 34.0 mmol), 1,3-cyclohexanedione (2.86 g, 25.5 mmol) and 1-iodo-4-(trifluoromethyl)benzene (1.08 mL, 8.51 mmol) in anhydrous DMSO (25 mL) to give a white crystalline solid (630 mg, 29%). $R_f$ 0.38 (14:2:1 EtOAc:*i*-PrOH:$H_2O$); IR 1714 (C=O), 1681 (C=O), 1600, 1405, 1359, 1266, 1026, 838, 733 cm$^{-1}$; $^{1}$H NMR (400 MHz, $(CD_3)_2SO$) δ 11.0, (1H, br s, O**H**), 7.87 (2H, d, *J* = 8.2 Hz, 2 × Ar**H**), 7.29 (2H, d, *J* = 8.2 Hz, 2 × Ar**H**), 2.57 (3H, s, C**H**$_3$), 2.53–2.42 (4H, m, C**H**$_2$CH$_2$C**H**$_2$), 2.02–1.90 (2H, m, CH$_2$C**H**$_2$CH$_2$); $^{13}$C NMR (100.6 MHz, $(CD_3)_2SO$) δ 197.6 (C), 139.7 (C), 134.4 (C), 131.1 (2 × CH), 127.1 (2 × CH), 115.5 (C), 26.6 (CH$_2$), 20.2 (CH$_3$), some signals not observed due to keto–enol tautomerism; HRMS (ESI +ve) Exact mass calculated for $C_{13}H_{15}O_3$ [M+H]$^+$: 213.1016, found: 213.1022.

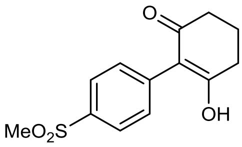


**3-Hydroxy-2-(4-methanesulfonylphenyl)cyclohex-2-en-1-one (1h)**. The title compound was prepared according to General Procedure B using CuI (81.0 mg, 0.43 mmol), *L*-proline (97.9 mg, 0.85 mmol), $K_2CO_3$ (2.35 g, 17.0 mmol), 1,3-cyclohexanedione (1.43 g, 12.8 mmol) and 1-bromo-4-methanesulfonylbenzene (1.00 g, 4.25 mmol) in anhydrous DMSO (15 mL) to give a white crystalline solid (200 mg, 18%). $R_f$ 0.38 (14:2:1 EtOAc:*i*-PrOH:$H_2O$); m.p. 266–267 ºC (acetone/petroleum ether); IR 1601 (C=O), 1558, 1300, 1268, 1152, 989, 960, 775 cm$^{-1}$; $^{1}$H NMR (400 MHz, $(CD_3)_2SO$) δ 11.07 (1H, br s, O**H**), 7.86–7.80 (2H, m, 2 × Ar**H**), 7.44–7.38 (2H, m, 2 × Ar**H**), 3.22 (3H, s, C**H**$_3$), 2.63–2.42 (4H, m, C**H**$_2$CH$_2$C**H**$_2$), 2.04–1.91 (2H, m, CH$_2$C**H**$_2$CH$_2$); $^{13}$C NMR (100.6 MHz, $(CD_3)_2SO$) δ 140.0 (C), 138.0 (C), 131.7 (2 × CH), 125.8 (2 × CH), 115.0 (C), 43.6 (CH$_3$), 20.2 (CH$_2$), some signals not observed due to keto–enol tautomerism; HRMS (ESI +ve) Exact mass calculated for $C_{13}H_{15}O_4S$ [M+H]$^+$: 267.3144, found: 267.0681.

**General Procedure B: Preparation of 1,3-Enynes**

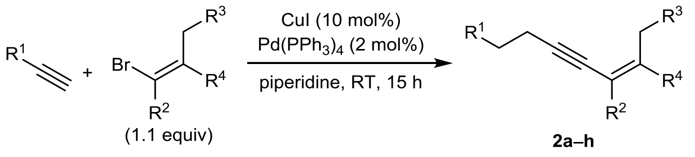


To a solution of the appropriate alkenyl bromide (1.1 equiv), $Pd(PPh_3)_4$ (2 mol%), CuI (10 mol%) in piperidine was added the appropriate alkyne (1.0 equiv) and the mixture was stirred at room temperature for 15 h. The reaction was diluted with $Et_2O$ (50 mL), washed with brine (50 mL), and the aqueous layer was further extracted with $Et_2O$ (2 × 50 mL). The organic layers were combined, dried ($MgSO_4$), and concentrated *in vacuo*. Purification of the residue by flash column chromatography (50:1 $Et_2O$:petroleum ether) gave the title 1,3-enyne.

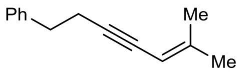

**(6-Methylhept-5-en-3-yn-1-yl)benzene (2a).**[4] The title compound was prepared according to General Procedure B using 1-bromo-2-methyl-1-propene (564 µL, 5.50 mmol), $Pd(PPh_3)_4$ (116 mg, 0.10 mmol), CuI (95.2 mg, 0.50 mmol), and 4-phenyl-1-butyne (703 µL, 5.00 mmol) in piperidine (20 mL) to give a colorless oil (902 mg, 98%). $R_f$ 0.63 (petroleum ether); IR 2360 (C≡C), 1731, 1696, 1453, 1270, 1076, 743, 697 $cm^{-1}$; $^1$H NMR (400 MHz, $CDCl_3$) δ 7.35–7.29 (2H, m, 2 × Ar**H**), 7.29–7.20 (3H, m, 3 × Ar**H**), 5.28–5.22 (1H, m, C**H**=), 2.89 (2H, t, *J* = 7.6 Hz, PhC$\mathbf{H}_2$), 2.66 (2H, td, *J* = 7.6, 1.7 Hz, $PhCH_2C\mathbf{H}_2$), 1.85 (3H, s, C$\mathbf{H}_3$), 1.80 (3H, d, *J* = 0.5 Hz, C$\mathbf{H}_3$); $^{13}$C NMR (100.6 MHz, $CDCl_3$) δ 147.1 (C), 140.9 (C), 128.5 (2 × CH), 128.3 (2 × CH), 126.2 (CH), 105.3 (CH), 91.2 (C). 79.1 (C), 35.5 ($CH_2$), 24.6 ($CH_2$), 21.7 ($CH_3$), 20.7 ($CH_3$); HRMS (ESI +ve) Exact mass calculated for $C_{14}H_{17}$ $[M+H]^+$: 185.1325, found: 185.1329. Physical and spectral properties were in accordance with the literature.[4]

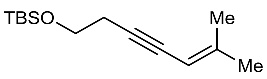

***tert*-Butyldimethyl[(6-methylhept-5-en-3-yn-1-yl)oxy]silane (2b)**. The title compound was prepared according to General Procedure B using 1-bromo-2-methyl-1-propene (1.12 mL, 11.0 mmol), $Pd(PPh_3)_4$ (231 mg, 0.20 mmol), CuI (190 mg, 1.00 mmol), and (but-3-yn-1-yloxy)(*tert*-butyl)dimethylsilane (2.06 mL, 10.0 mmol) in piperidine (40 mL) to give a colorless oil (1.51 g, 93%). $R_f$ 0.20 (25:1 petroleum ether:EtOAc); IR 2359 (C≡C), 1252, 1103, 834, 775 $cm^{-1}$; $^1$H NMR (400 MHz, $CDCl_3$) δ 5.23 (1H, s, C**H**=), 3.75 (2H, t, *J* = 7.3 Hz, OC$\mathbf{H}_2$), 2.56 (2H, td, *J* = 7.3, 1.9 Hz, $OCH_2C\mathbf{H}_2$), 1.87 (3H, s, =CC$\mathbf{H}_3$), 1.78 (3H, s, =CC$\mathbf{H}_3$), 0.91 (9H, s, C(C$\mathbf{H}_3$)$_3$), 0.08 (6H, s, 2 × SiC$\mathbf{H}_3$); $^{13}$C NMR (100.6 MHz, $CDCl_3$) δ 147.2 (C), 105.2 (CH), 88.6 (C), 79.5 (C), 62.3 ($CH_2$), 25.9 (3 × $CH_3$), 24.6 ($CH_3$), 23.9 ($CH_2$), 20.8 ($CH_3$), 18.3 (C), –5.3 (2 × $CH_3$); HRMS (ESI +ve) Exact mass calculated for $C_{14}H_{27}OSi$ $[M+H]^+$:

4. Sasaki, Y.; Horita, Y.; Zhong, C.; Sawamura, M.; Ito, H. *Angew. Chem., Int. Ed.* **2011**, *50*, 2778–2782.

239.1826, found: 239.1824.

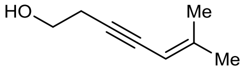

**6-Methylhept-5-en-3-yn-1-ol (2c).**[4] The title compound was prepared according to a modification of General Procedure B (in that the column eluent was 2:1 petroleum ether:EtOAc) using 1-bromo-2-methyl-1-propene (564 µL, 5.50 mmol), $Pd(PPh_3)_4$ (116 mg, 0.10 mmol), CuI (95.2 mg, 0.50 mmol), and but-3-yn-1-ol (703 µL, 5.00 mmol) in piperidine (20 mL) to give a colorless oil (584 mg, 94%). $R_f$ 0.29 (2:1 petroleum ether:EtOAc); IR 3351 (OH), 2359 (C≡C), 1445, 1378, 1335, 1044, 820, 642 $cm^{-1}$; $^1$H NMR (400 MHz, $CDCl_3$) δ 5.28–5.18 (1H, m, C**H**=), 3.78–3.66 (2H, OC$\mathbf{H}_2$), 2.60 (2H, td, *J* = 6.3, 2.0 Hz, $OCH_2C\mathbf{H}_2$), 2.11 (1H, s, O**H**), 1.86 (3H, s, C$\mathbf{H}_3$), 1.77 (3H, s, C$\mathbf{H}_3$); $^{13}$C NMR (100.6 MHz, $CDCl_3$) δ 147.7 (C), 104.9 (CH), 87.9 (C), 80.4 (C), 61.3 ($CH_2$), 24.6 ($CH_2$), 23.8 ($CH_3$), 20.8 ($CH_3$); HRMS (ESI +ve) Exact mass calculated for $C_8H_{12}ONa$ $[M+Na]^+$: 147.1704, found: 147.0766. Physical and spectral properties were in accordance with the literature.[4]

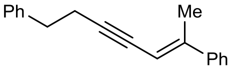

**[(*E*)-6-Phenylhept-5-en-3-yn-1-yl]benzene (2d)**. The title compound was prepared according to General Procedure B using [(*E*)-1-bromoprop-1-en-2-yl]benzene[5] (1.08 g, 5.50 mmol), $Pd(PPh_3)_4$ (116 mg, 0.10 mmol), CuI (95.2 mg, 0.50 mmol), and 4-phenyl-1-butyne (703 µL, 0.50 mmol) in piperidine (20 mL) to give a white solid (949 mg, 77%). $R_f$ 0.61 (10:1 petroleum ether:$Et_2O$); m.p. 38–39 ºC (petroleum ether); IR 2359 (C≡C), 1493, 1445, 1071, 1027, 756, 691 $cm^{-1}$; $^1$H NMR (400 MHz, $CDCl_3$) δ 7.49–7.43 (2H, m, 2 × Ar**H**), 7.41–7.24 (8H, m, 8 × Ar**H**), 5.94–5.88 (1H, m, C**H**=), 2.96 (2H, t, *J* = 7.5 Hz, PhC$\mathbf{H}_2$), 2.77 (2H, td, *J* = 7.5, 2.1 Hz, $PhCH_2C\mathbf{H}_2$), 2.32–2.25 (3H, m, C$\mathbf{H}_3$); $^{13}$C NMR (100.6 MHz, $CDCl_3$) δ 147.1 (C), 141.1 (C), 140.7 (C), 128.5 (2 × CH), 128.4 (2 × CH), 128.3 (2 × CH), 127.8 (CH), 126.3 (CH), 125.3 (2 × CH), 107.0 (CH), 95.4 (C), 79.7 (C), 35.3 ($CH_2$), 22.0 ($CH_2$), 18.4 ($CH_3$); HRMS (ESI +ve) Exact mass calculated for $C_{19}H_{19}$ $[M+H]^+$: 247.1481, found: 247.1482.

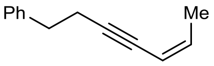

**(*Z*)-Hept-5-en-3-yn-1-ylbenzene (2e).**[6] The title compound was prepared according to General Procedure B using (*Z*)-1-bromoprop-1-ene (935 µL, 11.0 mmol), $Pd(PPh_3)_4$ (231 mg, 0.20 mmol), CuI (190 mg, 1.00 mmol), and 4-phenyl-1-butyne (1.40 mL, 10.0 mmol) in piperidine (40 mL) to give a colorless oil (296 mg, 98%). $R_f$ 0.59 (10:1 petroleum ether:$Et_2O$); IR 2360 (C≡C), 1496, 1453, 1362, 1077, 721, 697, 643 $cm^{-1}$; $^1$H NMR (400 MHz, $CDCl_3$) δ 7.39–7.31 (2H, m, 2 × Ar**H**), 7.31–7.23 (3H, m, 3 × Ar**H**), 5.93 (1H, dq, *J* = 10.7, 6.8 Hz, =C**H**$CH_3$), 5.55–5.45 (1H, m, C**H**=$CHCH_3$), 2.92 (2H, t, *J* = 7.6 Hz, PhC$\mathbf{H}_2$), 2.70 (2H,

5. Alem,K; Belder G.; Lodder, G.; Zuilhof, H. *J. Org. Chem.* **2005**, *70*, 179–190.
6. Sun, C.; O'Connor, M. J.; Lee, D.; Wink, D. J.; Milligan, R. D. *Angew. Chem., Int. Ed.* **2014**, *53*, 3197–3200.

ddd, *J* = 7.6, 2.0, 1.0 Hz, $PhCH_2C\mathbf{H}_2$), 1.86 (3H, dd, *J* = 6.8, 1.7 Hz, $C\mathbf{H}_3$); $^{13}C$ NMR (100.6 MHz, $CDCl_3$) δ 140.8 (C), 137.3 (CH), 128.5 (2 × CH), 128.3 (2 × CH), 126.2 (CH), 110.2 (CH), 94.0 (C), 77.8 (C), 35.3 ($CH_2$), 21.7 ($CH_2$), 15.7 ($CH_3$); HRMS (ESI +ve) Exact mass calculated for $C_{13}H_{15}$ $[M+H]^+$: 171.1168, found: 117.1173. Physical and spectral properties were in accordance with the literature.[6]

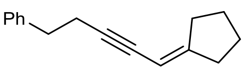

**(5-Cyclopentylidenepent-3-yn-1-yl)benzene (2f)**. The title compound was prepared according to General Procedure B using (bromomethylidene)cyclohexane[7] (726 µL, 5.50 mmol), $Pd(PPh_3)_4$ (116 mg, 0.10 mmol), CuI (95.2 mg, 0.50 mmol), and 4-phenyl-1-butyne (703 µL, 5.00 mmol) in piperidine (20 mL) to give a colorless oil (296 mg, 98%). $R_f$ 0.59 (10:1 petroleum ether:$Et_2O$); IR 2361 (C≡C), 1495, 1453, 1429, 1076, 697cm$^{-1}$; $^1H$ NMR (400 MHz, $CDCl_3$) δ 7.37–7.30 (2H, m, 2 × Ar**H**), 7.30–7.21 (3H, m, 3 × Ar**H**), 5.42–5.36 (1H, m, C**H**=), 2.90 (2H, t, *J* = 7.6 Hz, $PhC\mathbf{H}_2$), 2.67 (2H, t, *J* = 7.6 Hz, $PhCH_2C\mathbf{H}_2$), 2.46–2.31 (4H, m, $C\mathbf{H}_2CH_2CH_2C\mathbf{H}_2$), 1.79–1.64 (4H, $CH_2C\mathbf{H}_2C\mathbf{H}_2CH_2$); $^{13}C$ NMR (100.6 MHz, $CDCl_3$) δ 159.6 (C), 140.9 (C), 128.5 (2 × CH), 128.3 (2 × CH), 126.2 (CH), 100.2 (CH), 91.6 (C), 79.5 (C), 35.6 ($CH_2$), 33.5 ($CH_2$), 31.9 ($CH_2$), 26.6 ($CH_2$), 26.0 ($CH_2$), 21.8 ($CH_2$); HRMS (ESI +ve) Exact mass calculated for $C_{16}H_{19}$ $[M+H]^+$: 211.1481, found: 211.1489.

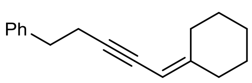

**(5-Cyclohexylidenepent-3-yn-1-yl)benzene (2g)**. The title compound was prepared according to General Procedure B using (bromomethylidene)cyclohexane (726 µL, 5.50 mmol), $Pd(PPh_3)_4$ (116 mg, 0.10 mmol), CuI (95.2 mg, 0.50 mmol), and 4-phenyl-1-butyne (703 µL, 5.00 mmol) in piperidine (20 mL) to give a colorless oil (296 mg, 98%). $R_f$ 0.59 (10:1 petroleum ether:$Et_2O$); IR 2360 (C≡C), 1495, 1446, 1076, 1030, 831, 759, 697 cm$^{-1}$; $^1H$ NMR (400 MHz, $CDCl_3$) δ 7.36–7.30 (2H, m, 2 × Ar**H**), 7.30–7.21 (3H, m, 3 × Ar**H**), 5.24–5.19 (1H, m, C**H**=), 2.90 (2H, t, *J* = 7.5 Hz, $PhC\mathbf{H}_2$), 2.66 (2H, td, *J* = 7.5, 2.0 Hz, $PhCH_2C\mathbf{H}_2$), 2.39–2.33 (2H, m, $=CC\mathbf{H}_2$), 2.22–2.12 (m, 2H, $=CC\mathbf{H}_2$), 1.63–1.54 (6H, m, $CH_2(C\mathbf{H}_2)_3CH_2$); $^{13}C$ NMR (100.6 MHz, $CDCl_3$) δ 154.5 (C), 140.9 (C), 128.5 (2 × CH), 128.3 (2 × CH), 126.1 (CH), 101.6 (CH), 91.0 (C), 78.7 (C), 35.8 ($CH_2$), 35.4 ($CH_2$), 31.3 ($CH_2$), 28.2 ($CH_2$), 27.5 ($CH_2$), 26.3 ($CH_2$), 21.8 ($CH_2$); HRMS (ESI +ve) Exact mass calculated for $C_{17}H_{21}$ $[M+H]^+$: 225.1638, found: 225.1635.

7. Wolinsky J.; Erickson K. L. *J. Org. Chem.* **1965**, *30*, 2208–2211.

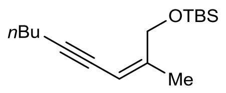


***tert*-Butyldimethyl{[(*Z*)-2-methylnon-2-en-4-yn-1-yl]oxy}silane (2h)**. The title compound was prepared according to a modification of General Procedure B (in that 1.0 equiv of alkenyl bromide and 1.1 equiv of alkyne were used) using {[(*Z*)-3-iodo-2-methylprop-2-en-1-yl]oxy}(*tert*-butyl)dimethylsilane[8] (400 mg, 1.28 mmol), $Pd(PPh_3)_4$ (29.6 mg, 25.6 µmol), CuI (24.4 mg, 0.13 mmol), and 1-hexyne (161 µL, 1.41 mmol) in piperidine (20 mL) to give a colorless oil (307 mg, 90%). $R_f$ 0.71 (10:1 petroleum ether:$Et_2O$); IR 2362 (C≡C), 1472, 1251, 1076, 835, 775 $cm^{-1}$; $^1H$ NMR (400 MHz, $CDCl_3$) δ 5.29 (1H, s, C**H**=), 4.38 (2H, s, OC**H**$_2$), 2.33 (2H, td, *J* = 6.8, 1.9 Hz, C**H**$_2$C≡), 1.82 (3H, s, =CC**H**$_3$), 1.59–1.37 (4H, m, C**H**$_2$C**H**$_2$CH$_2$C≡), 0.93 (3H, t, *J* = 7.3 Hz, C**H**$_3$CH$_2$), 0.92 (9H, s, C(C**H**$_3$)$_3$), 0.09 (6H, s, 2 × SiC**H**$_3$); $^{13}C$ NMR (100.6 MHz, $CDCl_3$) δ 149.0 (C), 105.7 (CH), 93.7 (C), 77.2 (C), 64.0 ($CH_2$), 31.0 ($CH_2$), 25.9 (3 × $CH_3$), 22.0 ($CH_2$), 19.6 ($CH_3$), 19.2 ($CH_2$), 18.3 (C), 13.6 ($CH_3$), –5.31 (2 × $CH_3$); HRMS (ESI +ve) Exact mass calculated for $C_{13}H_{15}$ $[M+H]^+$: 289.1958, found: 289.1951.

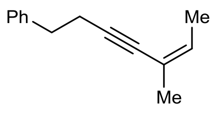


**[(*E*)-5-Methylhept-5-en-3-yn-1-yl]benzene (2i)**. The title compound was prepared according to a modification of General Procedure B (in that the reaction was heated at 60 °C) using (*Z*)-2-bromobut-2-ene (150 µL, 1.48 mmol), $Pd(PPh_3)_4$ (31.1 mg, 26.9 µmol), CuI (25.6 mg, 0.13 mmol), and 4-phenyl-1-butyne (116 µL, 1.34 mmol) in piperidine (5 mL) to give a colorless oil (150 mg, 55%). $R_f$ 0.63 (10:1 petroleum ether:$Et_2O$); IR 2261 (C≡C), 1604, 1496, 1453, 1349, 1076, 747, 697 $cm^{-1}$; $^1H$ NMR (400 MHz, $CDCl_3$) δ 7.47–7.23 (5H, m, 5 × Ar**H**), 5.73 (1H, qd, *J* = 6.7, 1.4 Hz, =C**H**), 2.97 (2H, t, *J* = 7.5 Hz, PhC**H**$_2$), 2.75 (2H, t, *J* = 7.5 Hz, PhCH$_2$C**H**$_2$), 1.95–1.88 (3H, m, C**H**$_3$), 1.85 (3H, ddd, *J* = 6.7, 3.0, 1.4 Hz, C**H**$_3$); $^{13}C$ NMR (100.6 MHz, $CDCl_3$) δ 140.7 (C), 131.0 (CH), 128.5 (2 × CH), 128.3 (2 × CH), 126.2 (CH), 118.9 (C), 93.1 (C), 80.5 (C), 35.4 ($CH_2$), 23.2 ($CH_3$), 21.7 ($CH_2$), 15.9 ($CH_3$); HRMS (ESI +ve) Exact mass calculated for $C_{14}H_{17}$ $[M+H]^+$: 185.1325, found: 185.1327.

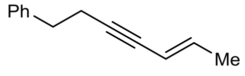


**(*E*)-Hept-5-en-3-yn-1-ylbenzene (14).[6]** The title compound was prepared according to General Procedure B using (*E*)-1-bromoprop-1-ene (180 µL, 1.96 mmol), $Pd(PPh_3)_4$ (41.2 mg, 35.6 µmol), CuI (34.3 mg, 0.18 mmol), and 4-phenyl-1-butyne (250 µL, 1.78 mmol) in piperidine (5 mL) to give a colorless oil (296 mg, 98%). $R_f$ 0.59 (10:1 petroleum ether:$Et_2O$); IR 2360 (C≡C), 1496, 1453, 952, 743, 697 $cm^{-1}$; $^1H$ NMR (400 MHz, $CDCl_3$) δ 7.38–7.29 (2H, m, 2 × Ar**H**), 7.28–7.20 (3H, m, 3 × Ar**H**), 6.08 (1H, dq, *J* = 15.7, 6.8 Hz, =C**H**CH$_3$), 5.53–5.45 (1H, dq, *J* = 15.7, 1.8 Hz, C**H**=CHCH$_3$), 2.87 (2H, t, *J* = 7.6 Hz, PhC**H**$_2$), 2.59 (2H, td, *J* = 7.6, 1.8 Hz, PhCH$_2$C**H**$_2$), 1.78 (3H, dd, *J* = 6.8, 1.8 Hz, C**H**$_3$); $^{13}C$ NMR (100.6

8. Werle, S.; Fey, T.; Neudörfl, J. M.; Schmalz, H. *Org. Lett.* **2007**, *9*, 3555–3558.

MHz, $CDCl_3$) δ 140.8 (C), 138.3 (CH), 128.4 (2 × CH), 128.3 (2 × CH), 126.2 (CH), 110.9 (CH), 87.6 (C), 79.8 (C), 35.3 ($CH_2$), 21.6 ($CH_2$), 18.4 ($CH_3$); HRMS (ESI +ve) Exact mass calculated for $C_{13}H_{15}$ $[M+H]^+$: 171.1168, found: 117.1174. Physical and spectral properties were in accordance with the literature.[6]

**3-($^2H_3$)Methyl(4,4,4-$^2H_3$)but-2-enoic acid ([D]$_6$-20)**

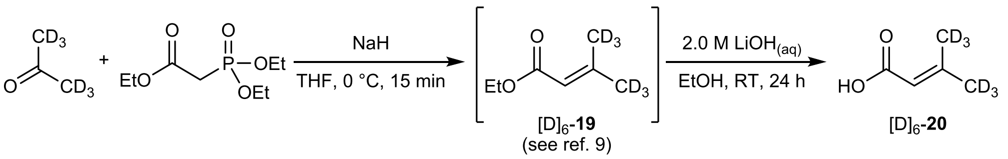


To a stirred solution of triethyl phosphonoacetate (71.4 mL, 0.36 mol) in THF (250 mL) was added NaH (12.0g of 60% wt in mineral oil, 0.30 mol) in 1.0 g portions over 10 min at 0 °C under $N_2$. The mixture was stirred at 0 °C for 1 h. Then, $D_6$-acetone (14.7 mL, 0.20 mol) was added dropwise over 15 min. The mixture was allowed to warm to room temperature and stirred for a further 3 h. Then, saturated $NH_4Cl_{(aq)}$ (500 mL) and $Et_2O$ (500 mL) were added, the layers were separated, and the aqueous layer was extracted with $Et_2O$ (2 × 250 mL). The combined organic layers were dried ($MgSO_4$) and concentrated *in vacuo*. The resulting oil was flushed through a silica plug using 10:1 petroleum ether:$Et_2O$ as eluent. The filtrate was concentrated *in vacuo* to leave the crude deuterio-ethyl ester [D]$_6$-**19**.[9] $R_f$ 0.38 (10:1 petroleum ether:$Et_2O$). To a stirred solution of crude ethyl ester [D]$_6$-**19** in EtOH (160 mL) was added 2.0 M $LiOH_{(aq)}$ (350 mL) at room temperature. The mixture was stirred at room temperature for 24 h. The reaction was cooled to 0 °C, acidified with 3.0 M $HCl_{(aq)}$ and $Et_2O$ (250 mL) was added. The layers were separated and the aqueous layer was extracted with $Et_2O$ (2 × 250 mL). The combined organic layers were dried ($MgSO_4$), filtered and concentrated *in vacuo* to give *deuterio-acid* [D]$_6$-**20** (18.2 g, 86% from $D_6$-acetone) as a white solid. $R_f$ 0.38 (1:1 petroleum ether:$Et_2O$); m.p. 71–72 ºC (petroleum ether); IR 2973 (OH), 1688 (C=O), 1621 (C=C), 1419, 1256, 1190, 1041, 923, 865, 697 $cm^{-1}$; $^1H$ NMR (400 MHz, $CDCl_3$) δ 11.93 (1H, br s, O**H**), 5.71 (1H, s, C**H**=); $^{13}C$ NMR (100.6 MHz, $CDCl_3$) δ 172.1 (C), 159.5 (C), 115.8 (CH).

9. Nagashima, T.; Rivkin, A.; Curran, D. P. *Can. J. Chem.*, **2000**, *78*, 791–799.

**2,3-Dibromo-3-($^2H_3$)methyl(4,4,4-$^2H_3$)butanoic acid ([D]$_6$-21)**

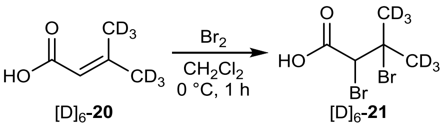


To a stirred solution of deuterio-acid [D]$_6$-**20** (6.00 g, 28.8 mmol) in $CH_2Cl_2$ (25 mL) was added bromine (1.63 mL, 31.7 mmol) dropwise over 10 min at 0 °C under $N_2$. The mixture was stirred at 0 °C for 1 h. Then, saturated $NaHSO_{3(aq)}$ (100 mL) was added, the layers were separated and the aqueous layer was extracted with $CH_2Cl_2$ (2 × 100 mL). The combined organic layers were dried ($MgSO_4$) and concentrated *in vacuo*. The resulting solid was recrystallized from $CH_2Cl_2$ and petroleum ether (liquor was cooled to 0 °C in order to initiate nucleation) to give *deuterio-dibromo acid* [D]$_6$-**21** (7.02 g, 92%) as colorless crystals. $R_f$ 0.18 (1:1 petroleum ether:$Et_2O$); $^1$H NMR (400 MHz, $CDCl_3$) δ 10.57 (1H, br s, O**H**), 4.68 (1H, s, C**H**=); $^{13}$C NMR (100.6 MHz, $CDCl_3$) δ 173.1 (C), 60.2 (C), 53.8 (CH).

**1-Bromo-2-($^2H_3$)methyl(3,3,3-$^2H_3$)prop-1-ene ([D]$_6$-22)**

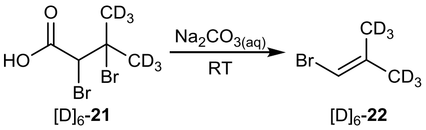


To[D]$_6$-**21** (7.02 g, 26.6 mmol) was added saturated $Na_2CO_{3(aq)}$ and when effervescence had ceased the reaction mixture was heated at 100 °C to distil off *deuterio-vinyl bromide* [D]$_6$-**22** (2.67 g, 72%) as a colorless oil. $R_f$ 0.59 (1:1 petroleum ether:$Et_2O$); b.p. 82 °C (1 atm); IR 1624, 1291, 1181, 1047, 982, 810 cm$^{-1}$; $^1$H NMR (400 MHz, $CDCl_3$) δ 5.86 (1H, s, C**H**=); $^{13}$C NMR (100.6 MHz, $CDCl_3$) δ 137.8 (C), 100.5 (CH), 23.2 (septet, $J$ = 19.7 Hz, $CD_3$), 19.9 (septet, $J$ = 19.4 Hz, $CD_3$).

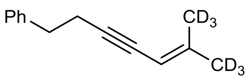

**[6-($^2H_3$)Methyl(7,7,7-$^2H_3$)hept-5-en-3-yn-1-yl]benzene ([D]$_6$-2a)**. The title compound was prepared according to General Procedure B using deuterio-vinyl bromide [D]$_6$-**22** (500 mg, 3.57 mmol), $Pd(PPh_3)_4$ (75.0 mg, 64.9 µmol), CuI (61.8 mg, 0.32 mmol), and 4-phenyl-1-butyne (367 µL, 3.25 mmol) in piperidine (15 mL) to give a colorless oil (427 mg, 63%). $R_f$ 0.63 (10:1 petroleum ether:$Et_2O$); IR 2191 (C≡C), 1496, 1453, 1340, 1076, 1045, 850, 697 cm$^{-1}$; $^1$H NMR (400 MHz, $CDCl_3$) δ 7.37–7.31 (2H, m, 2 × Ar**H**), 7.31–7.23 (3H, m, 3 × Ar**H**), 5.28 (1H, t, $J$ = 2.1 Hz, C**H**=), 2.91 (2H, t, $J$ = 7.6 Hz, PhC**H**$_2$), 2.68 (2H, td, $J$ = 7.6, 2.1 Hz, PhCH$_2$C**H**$_2$); $^{13}$C NMR (100.6 MHz, $CDCl_3$) δ 146.9 (C), 140.9 (CH), 128.4 (2 × CH), 128.3 (2 × CH), 126.2 (CH), 105.3 (C), 91.0 (C), 79.1 (C), 35.5 ($CH_2$), 21.7 ($CH_2$); HRMS (ESI +ve) Exact mass calculated for $C_{14}H_{10}D_6Na$ $[M+Na]^+$: 213.1521, found: 213.1520.

## 3. One-Carbon Oxidative Annulation Reactions of 1,3-Enynes

### General Procedure C

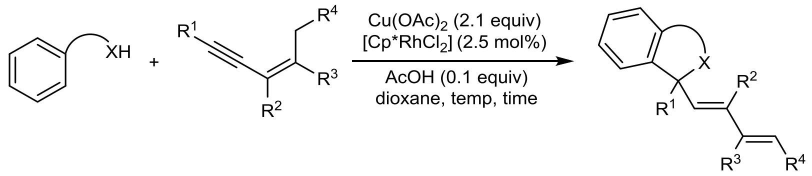


To a microwave vial, was added the appropriate C-H functionalization partner (0.50 mmol), $[Cp*RhCl_2]_2$ (7.7 mg, 13 µmol), $Cu(OAc)_2$ (191 mg, 1.05 mmol), AcOH (3 µL, 50 µmol), and the appropriate enyne (0.55 mmol). The vessel was then sealed, flushed with $N_2$, and 1,4-dioxane (5 mL) was added. The reaction was then heated at the indicated temperature for the specified time. The reaction was cooled to room temperature, filtered through a short pad of silica using $Et_2O$ (25 mL) as eluent and concentrated *in vacuo*. Purification of the residue by flash column chromatography gave the title compound(s).

**6-[(*E*)-3-Methylbuta-1,3-dien-1-yl]-1-oxo-6-(2-phenylethyl)-1*H*,2*H*,3*H*,4*H*,6*H*-benzo[*c*] benzopyran-8-carboxylate (4a) and 2'-(2-methylprop-1-en-1-yl)-2,6-dioxo-3'-(2-phenylethyl) spiro[cyclohexane-1,1'-indene]-5'-carboxylate (3a)**

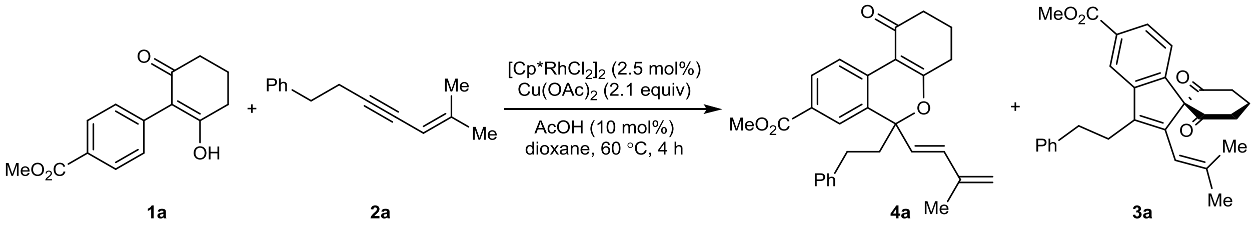


General procedure C was followed using 2-aryl-3-hydroxy-2-cyclohexenone **1a** (123 mg, 0.50 mmol) and enyne **2a** (101 mg, 0.55 mmol) at 60 °C for a reaction time of 4 h. Purification by flash column chromatography (3:1 petroleum ether:EtOAc) gave *benzopyran* **4a** (184 mg, 86%) as a yellow solid followed by *spiroindene* **3a** (25.9 mg, 12%) as a yellow oil.

### Data for benzopyran 4a

$R_f$ 0.54 (2:1 petroleum ether:EtOAc); IR 1718 (C=O), 1654 (C=O), 1608 (C=C), 1377, 1225, 908, 730, 647 $cm^{-1}$; $^1H$ NMR (400 MHz, $CDCl_3$) δ 8.66 (1H, d, *J* = 8.4 Hz, Ar**H**), 8.01 (1H, dd, *J* = 8.4, 1.8 Hz, Ar**H**), 7.85 (1H, d, *J* = 1.8 Hz, Ar**H**), 7.34–7.27 (2H, m, 2 × Ar**H**), 7.25–7.16 (3H, m, 3 × Ar**H**), 6.07 (1H, d, *J* = 15.8 Hz, C**H**=CHCCH$_3$), 5.75 (1H, d, *J* = 15.8 Hz, =C**H**CCH$_3$), 5.04 (1H, s, =C**H$_A$**H$_B$), 4.96 (1H, s, =CH$_A$**H$_B$**), 3.95 (3H, s, OC**H$_3$**), 2.82–2.69 (2H, m, PhC**H$_2$**), 2.64–2.50 (5H, m, C**H$_2$**CH$_2$C**H$_2$** and PhCH$_2$C**H$_A$**H$_B$), 2.42 (1H, ddd, *J* = 14.4, 10.0, 6.8 Hz PhCH$_2$CH$_A$**H$_B$**), 2.10–

1.99 (2H, m, $CH_2C\mathbf{H}_2CH_2$), 1.83 (3H, s, $CC\mathbf{H}_3$); $^{13}$C NMR (100.6 MHz, $CDCl_3$) δ 196.1 (C), 173.5 (C), 166.8 (C), 141.4 (C), 140.5 (C), 133.9 (CH), 131.9 (C), 131.5 (C), 130.6 (CH), 129.3 (CH), 128.4 (2 × CH), 128.3 (2 × CH), 128.2 (C), 125.9 (CH), 125.2 (CH), 125.2 (CH), 118.7 ($CH_2$), 111.1 (C), 84.5 (C), 52.1 ($CH_3$), 40.8 ($CH_2$), 38.4 ($CH_2$), 30.2 ($CH_2$), 29.8 ($CH_2$), 19.9 ($CH_2$), 18.5 ($CH_3$); HRMS (ESI +ve) Exact mass calculated for $C_{28}H_{29}O_4$ $[M+H]^+$: 429.2060, found: 429.2051.

**Data for spiroindene 3a**

$R_f$ 0.40 (2:1 petroleum ether:EtOAc); IR (film) 1720 (C=O), 1637 (C=C), 1439, 1250, 908, 758, 734 cm$^{-1}$; $^1$H NMR (400 MHz, $CDCl_3$) δ 7.97 (1H, d, *J* = 1.3 Hz, Ar**H**), 7.92 (1H, dd, *J* = 7.9, 1.3 Hz, Ar**H**), 7.32–7.25 (2H, m, 2 × Ar**H**), 7.23–7.15 (3H, m, 3 × Ar**H**), 5.35 (1H, s, =C**H**), 3.95 (3H, s, $OC\mathbf{H}_3$), 2.99–2.85 (4H, m, 2 × $C\mathbf{H}_2$), 2.85–2.71 (4H, m, 2 × $C\mathbf{H}_2$), 2.26–2.07 (2H, m, $C\mathbf{H}_2$), 1.81 (3H, d, *J* = 1.3 Hz, $CC\mathbf{H}_3$), 1.63 (3H, d, *J* = 1.1 Hz, $CC\mathbf{H}_3$); $^{13}$C NMR (100.6 MHz, $CDCl_3$) δ 204.1 (2 × C), 166.9 (C), 147.6 (C), 146.2 (C), 141.8 (C), 141.5 (C), 141.4 (C), 140.2 (C), 130.2 (C), 128.6 (2 × CH), 128.2 (2 × CH), 127.3 (CH), 125.9 (CH), 122.6 (CH), 121.2 (CH), 117.5 (CH), 85.2 (C), 52.2 ($CH_3$), 39.9 (2 × $CH_2$), 34.0 ($CH_2$), 28.1 ($CH_2$), 25.6 ($CH_3$), 20.1 ($CH_3$), 17.3 ($CH_2$); HRMS (ESI +ve) Exact mass calculated for $C_{28}H_{28}O_4Na$ $[M+Na]^+$ 451.1880, found 451.1887, exact mass calculated for $C_{28}H_{29}O_4$ $[M+H]^+$ 429.2060, found 429.2067.

**6-[(*E*)-3-Methylbuta-1,3-dien-1-yl]-6-(2-phenylethyl)-1*H*,2*H*,3*H*,4*H*,6*H*-benzo[*c*]chromen-1-one (4b) and 2'-(2-methylprop-1-en-1-yl)-3'-(2-phenylethyl)spiro[cyclohexane-1,1'-indene]-2,6-dione (3b)**

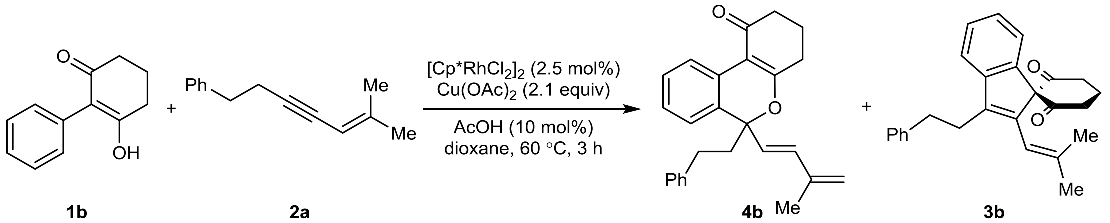


General procedure C was followed using 2-aryl-3-hydroxy-2-cyclohexenone **1b** (94.1 mg, 0.50 mmol) and enyne (101 mg, 0.55 mmol) at 60 °C for a reaction time of 3 h. Purification by flash column chromatography (3:1 petroleum ether:EtOAc) gave *benzopyran* **4b** (37.1 mg, 20%) as a yellow oil followed by *spiroindene* **3b** (111 mg, 60%) as a yellow solid.

**Data for benzopyran 4b**

$R_f$ 0.50 (2:1 petroleum ether:EtOAc); m.p. 108–109 °C ($CHCl_3$); IR 1647 (C=O), 1603 (C=C), 1487, 1376, 1122, 700 cm$^{-1}$; $^1$H NMR (400 MHz, $CDCl_3$) δ 8.59 (1H, dd, *J* = 7.8, 1.2 Hz, Ar**H**), 7.38 (1H, td, *J* = 7.8, 1.4 Hz, Ar**H**), 7.35–7.28 (3H, m, 3 × Ar**H**), 7.26–7.17 (4H, m, 4 × Ar**H**), 6.13

(1H, d, $J$ = 15.8 Hz, C**H**=CHCCH$_3$), 5.80 (1H, d, $J$ = 15.8 Hz, =C**H**CCH$_3$), 5.09–5.02 (1H, m, =C**H$_A$**H$_B$), 5.02–4.95 (1H, m, =CH$_A$**H$_B$**), 2.87–2.70 (2H, m, PhC**H**$_2$), 2.66–2.48 (5H, m, C**H**$_2$CH$_2$C**H**$_2$ and PhCH$_2$C**H$_A$**H$_B$), 2.46–2.33 (1H, m, PhCH$_2$CH$_A$**H$_B$**), 2.12–1.99 (2H, m, CH$_2$C**H**$_2$CH$_2$), 1.90–1.83 (3H, m, C**H**$_3$); $^{13}$C NMR (100.6 MHz, CDCl$_3$) δ 196.2 (C), 171.7 (C), 141.7 (C), 140.7 (C), 133.6 (CH), 131.5 (C), 131.1 (CH), 128.4 (2 × CH), 128.3 (2 × CH), 127.9 (CH), 127.3 (C), 126.9 (CH), 125.9 (CH), 125.3 (CH), 123.8 (CH), 118.3 (CH$_2$), 111.6 (C), 84.3 (C), 40.7 (CH$_2$), 38.5 (CH$_2$), 30.2 (CH$_2$), 29.6 (CH$_2$), 20.0 (CH$_2$), 18.5 (CH$_3$); HRMS (ESI +ve) Exact mass calculated for $C_{26}H_{27}O_2$ $[M+H]^+$: 371.2006, found: 371.2006.

**Data for spiroindene 3b**

R$_f$ 0.37 (2:1 petroleum ether:EtOAc); m.p. 118–119 °C (CHCl$_3$); IR (film) 1722 (C=O), 1696 (C=C), 1451, 1260, 1078, 741 cm$^{-1}$; $^1$H NMR (400 MHz, CDCl$_3$) δ 7.41–7.30 (5H, m, Ar**H**), 7.26–7.18 (4H, m, Ar**H**), 5.51 (1H, s, =C**H**), 3.03–2.89 (4H, m, 2 × C**H**$_2$), 2.86–2.74 (4H, m, 2 × C**H**$_2$), 2.27–2.16 (1H, m, C**H$_A$**H$_B$), 2.16–2.03 (1H, m, CH$_A$**H$_B$**), 1.85 (1H, d, $J$ = 1.3 Hz, C**H**$_3$), 1.72 (1H, d, $J$ = 1.1 Hz, C**H**$_3$); $^{13}$C NMR (100.6 MHz, CDCl$_3$) δ 204.6 (2 × C), 145.6 (C), 142.6 (C), 142.3 (C), 141.8 (C), 140.8 (C), 139.3 (C), 128.4 (2 × CH), 128.2 (2 × CH), 128.1 (CH), 125.8 (CH), 125.3 (CH), 122.3 (CH), 120.4 (CH), 117.8 (CH), 84.9 (C), 39.7 (2 × CH$_2$), 34.0 (CH$_2$), 28.3 (CH$_2$), 25.6 (CH$_3$), 20.2 (CH$_3$), 17.3 (CH$_2$); HRMS (ESI +ve) Exact mass calculated for $C_{26}H_{26}NaO_2$ $[M+Na]^+$: 393.1825, found: 393.1837; exact mass calculated for $C_{26}H_{27}O_2$ $[M+H]^+$: 371.2006, found: 371.1837.

**8-Methoxy-6-[(*E*)-3-methylbuta-1,3-dien-1-yl]-6-(2-phenylethyl)-1*H*,2*H*,3*H*,4*H*,6*H*-benzo[*c*]chromen-1-one (4c) and 5'-methoxy-2'-(2-methylprop-1-en-1-yl)-3'-(2-phenylethyl) spiro[cyclohexane-1,1'-indene]-2,6-dione (3c)**

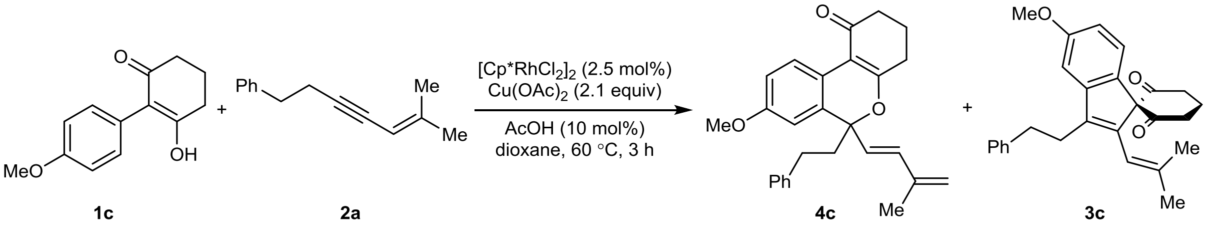


General procedure C was followed using 2-aryl-3-hydroxy-2-cyclohexenone **1c** (109 mg, 0.50 mmol) and enyne **2a** (101 mg, 0.55 mmol) at 60 °C for a reaction time of 3 h. Purification by flash column chromatography (3:1 petroleum ether:EtOAc) gave *benzopyran* **4c** (34.3 mg, 17%) as a yellow oil followed by *spiroindene* **3c** (129 mg, 64%) as a yellow solid.

**Data for benzopyran 4c**

$R_f$ 0.51 (2:1 petroleum ether:EtOAc); IR 1649 (C=O), 1601 (C=C), 1374, 1252, 1046, 732, 649 $cm^{-1}$; $^{1}$H NMR (400 MHz, $CDCl_3$) δ 8.59 (1H, d, *J* = 8.8 Hz, Ar**H**), 7.37 (2H, t, *J* = 7.6 Hz, 2 × Ar**H**), 7.31–7.21 (3H, m, 3 × Ar**H**), 6.96 (1H, dd, *J* = 8.8, 2.6 Hz, Ar**H**), 6.78 (1H, d, *J* = 2.6 Hz, Ar**H**), 6.20 (1H, d, *J* = 15.8 Hz, C**H**=CHCCH$_3$), 5.81 (1H, d, *J* = 15.8 Hz, =C**H**CCH$_3$), 5.09 (1H, s, =C$\mathbf{H_A}$H$_B$), 5.03 (1H, s, =CH$_A$$\mathbf{H_B}$), 3.91 (3H, s, OC$\mathbf{H_3}$), 2.92–2.73 (2H, m, PhC$\mathbf{H_2}$), 2.70–2.50 (5H, m, C$\mathbf{H_2}$CH$_2$C$\mathbf{H_2}$ and PhCH$_2$C$\mathbf{H_A}$H$_B$), 2.40 (1H, ddd, *J* = 14.4, 10.5, 6.4 Hz, PhCH$_2$CH$_A$$\mathbf{H_B}$), 2.15–2.04 (2H, m, CH$_2$C$\mathbf{H_2}$CH$_2$), 1.89 (3H, s, CC$\mathbf{H_3}$); $^{13}$C NMR (100.6 MHz, $CDCl_3$) δ 196.5 (C), 170.1 (C), 158.4 (C), 141.7 (C), 140.7 (C), 133.50 (CH), 133.45 (C), 131.0 (CH), 128.4 (2 × CH), 128.3 (2 × CH), 126.8 (CH), 125.9 (CH), 120.2 (C), 118.3 ($CH_2$), 111.9 (CH), 111.4 (C), 110.6 (CH), 84.0 (C), 55.3 ($CH_3$), 40.8 ($CH_2$), 38.5 ($CH_2$), 30.2 ($CH_2$), 29.5 ($CH_2$), 20.2 ($CH_2$), 18.5 ($CH_3$); HRMS (ESI +ve) Exact mass calculated for $C_{27}H_{29}O_3$ $[M+H]^+$: 401.2111, found: 401.2114.

**Data for spiroindene 3c**

$R_f$ 0.31 (2:1 petroleum ether:EtOAc); m.p. 115–116 °C ($CHCl_3$); IR (film) 1723 (C=O), 1690 (C=C), 1468, 1215, 1033, 756, 652 $cm^{-1}$; $^{1}$H NMR (400 MHz, $CDCl_3$) δ 7.36–7.28 (2H, m, 2 × Ar**H**), 7.27–7.19 (4H, m, 4 × Ar**H**), 6.91 (1H, d, *J* = 2.4 Hz, Ar**H**), 6.74 (1H, dd, *J* = 8.3, 2.4 Hz, Ar**H**), 5.51 (1H, s, =C**H**), 3.85 (3H, s, OC$\mathbf{H_3}$), 3.04–2.89 (4H, m, 2 × C$\mathbf{H_2}$), 2.86–2.71 (4H, m, 2 × C$\mathbf{H_2}$), 2.28–2.16 (1H, m, C$\mathbf{H_A}$H$_B$), 2.16–2.03 (1H, m, CH$_A$$\mathbf{H_B}$), 1.85 (3H, d, *J* = 0.8 Hz, CC$\mathbf{H_3}$), 1.72 (3H, s, CC$\mathbf{H_3}$); $^{13}$C NMR (100.6 MHz, $CDCl_3$) δ 204.8 (2 × C), 160.0 (C), 147.2 (C), 142.1 (2 × C), 141.7 (C), 139.2 (C), 134.7 (C), 128.4 (2 × CH), 128.2 (2 × CH), 125.8 (CH), 122.8 (CH), 117.9 (CH), 110.2 (CH), 106.8 (CH), 84.1 (C), 55.4 ($CH_3$), 39.6 (2 × $CH_2$), 34.0 ($CH_2$), 28.2 ($CH_2$), 25.7 ($CH_3$), 20.2 ($CH_3$), 17.3 ($CH_2$); HRMS (ESI +ve) Exact mass calculated for $C_{27}H_{28}NaO_3$ $[M+Na]^+$: 423.1931, found: 423.1928; exact mass calculated for $C_{27}H_{29}O_3$ $[M+H]^+$: 401.2111, found: 401.2116.

**8-Fluoro-6-[(*E*)-3-methylbuta-1,3-dien-1-yl]-6-(2-phenylethyl)-1*H*,2*H*,3*H*,4*H*,6*H*-benzo[*c*]chromen-1-one (4d) and 5'-fluoro-2'-(2-methylprop-1-en-1-yl)-3'-(2-phenylethyl)spiro[cyclohexane-1,1'-indene]-2,6-dione (3d)**

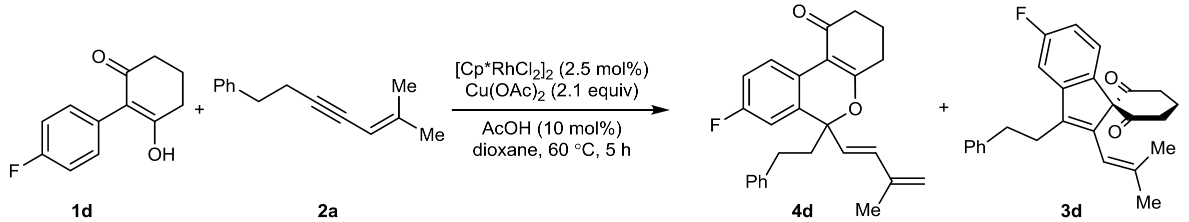


General procedure C was followed using 2-aryl-3-hydroxy-2-cyclohexenone **1d** (103 mg, 0.50 mmol) and enyne **2a** (101 mg, 0.55 mmol) at 60 °C for a reaction time of 5 h. Purification by flash column chromatography (3:1 petroleum ether:EtOAc) gave *benzopyran* **4d** (85.6 mg, 44%) as a yellow oil followed by *spiroindene* **3d** (50.1 mg, 26%) as a yellow solid.

**Data for benzopyran 4d**

$R_f$ 0.50 (2:1 petroleum ether:EtOAc); IR 1651 (C=O), 1601 (C=C), 1495, 1375, 1179, 909, 732, 640 $cm^{-1}$; $^1$H NMR (400 MHz, $CDCl_3$) δ 8.59 (1H, dd, *J* = 8.8, 6.0 Hz, Ar**H**), 7.33–7.27 (2H, m, Ar**H**), 7.24–7.16 (3H, m, 3 × Ar**H**), 7.04 (1H, td, *J* = 8.8, 2.7 Hz, Ar**H**), 6.87 (1H, dd, *J* = 9.5, 2.7 Hz, Ar**H**), 6.12 (1H, d, *J* = 15.8 Hz, C**H**=CHCCH$_3$), 5.73 (1H, d, *J* = 15.8 Hz, =C**H**CCH$_3$), 5.05 (1H, s, =C**H$_A$**H$_B$), 4.99 (1H, s, =CH$_A$**H$_B$**), 2.83–2.67 (2H, m, PhC**H$_2$**), 2.64–2.42 (5H, m, C**H$_2$**CH$_2$C**H$_2$** and PhCH$_2$C**H$_A$**H$_B$), 2.34 (1H, ddd, *J* = 14.4, 10.9, 6.0 Hz, PhCH$_2$CH$_A$**H$_B$**), 2.08–1.97 (2H, m, CH$_2$C**H$_2$**CH$_2$), 1.84 (3H, s, C**H$_3$**); $^{13}$C NMR (100.6 MHz, $CDCl_3$) δ 196.2 (C), 171.0 (C), 162.6 (d, *J* = 246.1 Hz, C), 141.4 (C), 140.5 (C), 134.0 (CH), 133.9 (C), 130.4 (CH), 128.4 (2 × CH), 128.3 (2 × CH), 127.4 (d, *J* = 7.6 Hz, CH), 126.0 (CH), 123.5 (d, *J* = 3.2 Hz, C), 118.7 ($CH_2$), 114.6 (d, *J* = 20.5 Hz, CH), 111.0 (C), 110.9 (d, *J* = 23.3 Hz, CH), 83.9 (d, *J* = 1.9 Hz, C), 40.7 ($CH_2$), 38.4 ($CH_2$), 30.1 ($CH_2$), 29.4 ($CH_2$), 20.0 ($CH_2$), 18.5 ($CH_3$); $^{19}$F NMR (376 MHz, $CDCl_3$) δ –114.1 (td, *J* = 8.8, 6.1 Hz); HRMS (ESI +ve) Exact mass calculated for $C_{26}H_{26}FO_2$ $[M+H]^+$: 389.1911, found: 389.1917.

**Data for spiroindene 3d**

$R_f$ 0.35 (2:1 petroleum ether:EtOAc); m.p. 144–145 °C ($CHCl_3$); IR (film) 1722 (C=O), 1692 (C=C), 1470, 1259, 1037, 807, 701, 651 $cm^{-1}$; $^1$H NMR (400 MHz, $CDCl_3$) δ 7.34–7.27 (2H, m, 2 × Ar**H**), 7.25–7.16 (4H, m, 4 × Ar**H**), 7.02 (1H, dd, *J* = 8.7, 2.4 Hz, Ar**H**), 6.89 (1H, t, *J* = 8.7 Hz, Ar**H**), 5.42 (1H, s, =C**H**), 2.99–2.75 (6H, m, 3 × C**H$_2$**), 2.75–2.66 (2H, m, 2 × C**H$_2$**), 2.25–2.08 (2H, m, C**H$_2$**), 1.83 (3H, d, *J* = 1.3 Hz, C**H$_3$**), 1.67 (3H, d, *J* = 1.0 Hz, C**H$_3$**); $^{13}$C NMR (100.6 MHz, $CDCl_3$) δ 204.5 (2 × C), 164.5 (d, *J* = 245.6 Hz, C), 148.2 (d, *J* = 8.5 Hz, C), 142.8 (C), 141.7 (d, *J*

= 2.8 Hz, C), 141.4 (C), 140.1 (C), 138.2 (d, *J* = 2.6 Hz, C), 128.5 (2 × CH), 128.3 (2 × CH), 126.0 (CH), 123.4 (d, *J* = 9.2 Hz, CH), 117.6 (CH), 111.8 (d, *J* = 23.4 Hz, CH), 107.9 (d, *J* = 23.7 Hz, CH), 84.3 (C), 39.8 (2 × $CH_2$), 34.0 ($CH_2$), 28.3 ($CH_2$), 25.6 ($CH_3$), 20.2 ($CH_3$), 17.3 ($CH_2$); $^{19}$F NMR (376 MHz, $CDCl_3$) δ –114.0 (td, *J* = 9.0, 4.8 Hz); HRMS (ESI +ve) Exact mass calculated for $C_{26}H_{25}FO_2Na$ [M+Na]$^+$: 411.1731, found: 411.1738; exact mass calculated for $C_{26}H_{26}FO_2$ [M+H]$^+$: 389.1911, found: 389.1917.

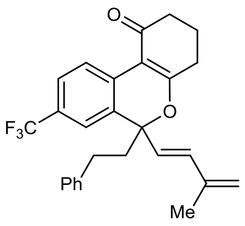


**6-[(*E*)-3-Methylbuta-1,3-dien-1-yl]-6-(2-phenylethyl)-8-(trifluoromethyl) -1*H*,2*H*,3*H*,4*H*,6*H*-benzo[*c*]chromen-1-one (4e).** The title compound was prepared according to General Procedure C from 2-aryl-3-hydroxy-2-cyclohexenone **E** (128 mg, 0.50 mmol) and enyne **2a** (101 mg, 0.55 mmol) at 60 °C for a reaction time of 3 h and was purified by flash column chromatography (3:1 petroleum ether:EtOAc) to give a yellow oil (172 mg, 78%). $R_f$ 0.62 (2:1 petroleum ether:EtOAc); IR 1655 (C=O), 1600 (C=C), 1378, 1331, 1163, 1121, 1081, 753, 653 cm$^{-1}$; $^1$H NMR (400 MHz, $CDCl_3$) δ 8.74 (1H, d, *J* = 8.4 Hz, Ar**H**), 7.62 (1H, dd, *J* = 8.4, 1.2 Hz, Ar**H**), 7.41 (1H, s, Ar**H**), 7.32 (2H, t, *J* = 7.3 Hz, 2 × Ar**H**), 7.26–7.17 (3H, m, 3 × Ar**H**), 6.13 (1H, d, *J* = 15.8 Hz, C**H**=CHCCH$_3$), 5.77 (1H, d, *J* = 15.8 Hz, =C**H**CCH$_3$), 5.08 (1H, s, =C$\mathbf{H_A}$H$_B$), 5.01 (1H, s, =CH$_A$$\mathbf{H_B}$), 2.90–2.67 (2H, m, PhC$\mathbf{H}_2$), 2.67–2.49 (5H, m, C$\mathbf{H}_2$CH$_2$C$\mathbf{H}_2$ and PhCH$_2$C$\mathbf{H_A}$H$_B$), 2.42 (1H, ddd, *J* = 14.5, 10.8, 6.0 Hz, PhCH$_2$CH$_A$$\mathbf{H_B}$), 2.14–1.96 (2H, m, CH$_2$C$\mathbf{H}_2$CH$_2$), 1.86 (3H, s, C$\mathbf{H}_3$); $^{13}$C NMR (100.6 MHz, $CDCl_3$) δ 195.9 (C), 173.2 (C), 141.2 (C), 140.4 (C), 134.2 (CH), 132.1 (C), 130.8 (C), 130.1 (CH), 128.6 (q, *J* = 32.4 Hz, C) 128.5 (2 × CH), 128.2 (2 × CH), 126.0 (CH), 125.6 (CH), 124.8 (q, *J* = 3.6 Hz, CH), 124.1 (q, *J* = 272.0 Hz, C), 120.6 (q, *J* = 3.1 Hz, CH), 118.9 ($CH_2$), 110.8 (C), 84.2 (C), 40.7 ($CH_2$), 38.3 ($CH_2$), 35.4 ($CH_2$), 30.2 ($CH_2$), 29.6 ($CH_2$), 19.8 ($CH_2$), 18.4 ($CH_3$); $^{19}$F NMR (376 MHz, $CDCl_3$) δ –62.0 (s); HRMS (ESI +ve) Exact mass calculated for [M+H]$^+$: $C_{27}H_{26}F_3O_2$, 439.1879 found: 439.1878.

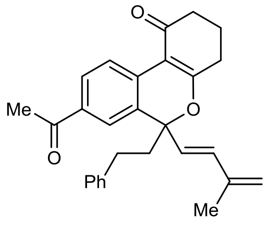


**8-Acetyl-6-[(*E*)-3-methylbuta-1,3-dien-1-yl]-6-(2-phenylethyl)-1*H*,2*H*,3*H*,4*H*,6*H*-benzo[*c*]chromen-1-one (4f).** The title compound was prepared according to General Procedure C from 2-aryl-3-hydroxy-2-cyclohexenone **1f** (115 mg, 0.50 mmol) and enyne **2a** (101 mg, 0.55 mmol) at 60 °C for a reaction time of 3 h and was purified by flash column chromatography (2:1 petroleum ether:EtOAc) to give a yellow oil (134 mg, 65%). $R_f$ 0.56 (2:1 petroleum ether:EtOAc); IR 1678 (C=O), 1655 (C=O), 1602 (C=C), 1377, 1221, 995, 752, 656 cm$^{-1}$; $^1$H NMR (400 MHz, $CDCl_3$) δ 8.67 (1H, d, *J* = 8.4 Hz, Ar**H**), 7.89 (1H, dd, *J* = 8.4, 1.8 Hz, Ar**H**),

7.80 (1H, d, *J* = 1.8 Hz, Ar**H**), 7.28 (2H, dd, *J* = 9.5, 5.2 Hz, 2 × Ar**H**), 7.22–7.14 (3H, m, 3 × Ar**H**), 6.06 (1H, d, *J* = 15.8 Hz, C**H**=CHCCH$_3$), 5.74 (1H, d, *J* = 15.8 Hz, =C**H**CCH$_3$), 5.02 (1H, s, =C**H$_A$**H$_B$), 4.94 (1H, s, =CH$_A$**H$_B$**), 2.81–2.66 (2H, m, PhC**H$_2$**), 2.62–2.50 (5H, m, C**H$_2$**CH$_2$C**H$_2$** and PhCH$_2$C**H$_A$**H$_B$), 2.61 (3H, s, COC**H$_3$**), 2.41 (1H, ddd, *J* = 14.4, 10.2, 6.4 Hz, PhCH$_2$CH$_A$**H$_B$**), 2.11–1.93 (2H, m, CH$_2$C**H$_2$**CH$_2$), 1.81 (3H, s, CC**H$_3$**); $^{13}$C NMR (100.6 MHz, CDCl$_3$) δ 197.2 (C), 196.0 (C), 173.6 (C), 141.3 (C), 140.4 (C), 135.2 (C), 133.8 (CH), 132.0 (C), 131.8 (C), 130.6 (CH), 128.6 (CH), 128.4 (2 × CH), 128.3 (2 × CH), 125.9 (CH), 125.1 (CH), 123.4 (CH), 118.7 (CH$_2$), 111.0 (C), 84.5 (C), 40.8 (CH$_2$), 38.4 (CH$_2$), 30.2 (CH$_2$), 29.7 (CH$_2$), 26.4 (CH$_3$), 19.8 (CH$_2$), 18.4 (CH$_3$); HRMS (ESI +ve) Exact mass calculated for $C_{28}H_{29}O_3$ [M+H]$^+$: 413.2111, found: 413.2121.

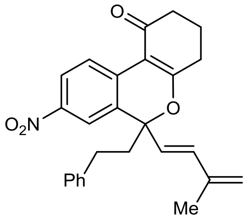


**6-[(*E*)-3-Methylbuta-1,3-dien-1-yl]-8-nitro-6-(2-phenylethyl)-1*H*,2*H*,3*H*,4*H*,6*H*-benzo[*c*]chromen-1-one (4g).** The title compound was prepared according to General Procedure C from 2-aryl-3-hydroxy-2-cyclohexenone **1g** (117 mg, 0.50 mmol) and enyne **2a** (101 mg, 0.55 mmol) at 60 °C for a reaction time of 3 h and was purified by flash column chromatography (3:1 petroleum ether:EtOAc) to give a yellow oil (170 mg, 82%). R$_f$ 0.44 (2:1 petroleum ether:EtOAc); IR 1657 (C=O), 1596 (C=C), 1515 (NO$_2$), 1377, 1336, 995, 752, 668 cm$^{-1}$; $^1$H NMR (400 MHz, CDCl$_3$) δ 8.77 (1H, d, *J* = 8.9 Hz, Ar**H**), 8.18 (1H, dd, *J* = 8.9, 2.4 Hz, Ar**H**), 8.00 (1H, d, *J* = 2.4 Hz, Ar**H**), 7.35–7.25 (2H, m, 2 × Ar**H**), 7.24–7.13 (3H, m, 3 × Ar**H**), 6.08 (1H, d, *J* = 15.8 Hz, C**H**=CHCCH$_3$), 5.73 (1H, d, *J* = 15.8 Hz, =C**H**CCH$_3$), 5.07 (1H, s, =C**H$_A$**H$_B$), 4.99 (1H, s, =CH$_A$**H$_B$**), 2.83–2.67 (2H, m, PhC**H$_2$**), 2.67–2.50 (5H, m, C**H$_2$**CH$_2$C**H$_2$** and PhCH$_2$C**H$_A$**H$_B$), 2.43 (1H, ddd, *J* = 14.6, 10.4, 6.2 Hz, PhCH$_2$CH$_A$**H$_B$**), 2.13–1.99 (2H, m, CH$_2$C**H$_2$**CH$_2$), 1.83 (3H, s, C**H$_3$**); $^{13}$C NMR (100.6 MHz, CDCl$_3$) δ 195.8 (C), 174.5 (C), 146.1 (C), 140.9 (C), 140.3 (C), 134.6 (CH), 133.8 (C), 132.6 (C), 129.7 (CH), 128.5 (2 × CH), 128.3 (2 × CH), 126.1 (CH), 126.0 (CH), 123.3 (CH), 119.4 (CH and CH$_2$), 110.6 (C), 84.4 (C), 40.7 (CH$_2$), 38.3 (CH$_2$), 30.2 (CH$_2$), 29.8 (CH$_2$), 19.7 (CH$_2$), 18.4 (CH$_3$); HRMS (ESI +ve) Exact mass calculated for $C_{26}H_{26}NO_4$ [M+H]$^+$: 416.1856, found: 416.1868.

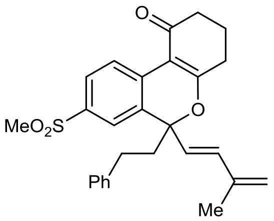


**8-Methanesulfonyl-6-[(*E*)-3-methylbuta-1,3-dien-1-yl]-6-(2-phenylethyl)-1*H*,2*H*,3*H*,4*H*,6*H*-benzo[*c*]chromen-1-one (4h).** The title compound was prepared according to General Procedure C from 2-aryl-3-hydroxy-2-cyclohexenone **1h** (133 mg, 0.50 mmol) and enyne **2a** (101 mg, 0.55 mmol) at 60 °C for a reaction time of 3 h and was purified by flash column chromatography (2:1 petroleum ether:EtOAc) to give a yellow oil (188 mg, 84%). R$_f$ 0.22 (2:1

petroleum ether:EtOAc); IR 1654 (C=O), 1594 (C=C), 1376 ($SO_2$), 1310, 1148 ($SO_2$), 964, 752, 642 $cm^{-1}$; $^1$H NMR (400 MHz, $CDCl_3$) δ 8.77 (1H, d, *J* = 8.5 Hz, Ar**H**), 7.86 (1H, dd, *J* = 8.5, 2.0 Hz, Ar**H**), 7.70 (1H, d, *J* = 2.0 Hz, Ar**H**), 7.28 (2H, *J* = 7.3 Hz, 2 × Ar**H**), 7.23–7.13 (3H, m, 3 × Ar**H**), 6.09 (1H, d, *J* = 15.8 Hz, C**H**=CHCCH$_3$), 5.72 (1H, d, *J* = 15.8 Hz, =C**H**CCH$_3$), 5.05 (1H, s, =C**H$_A$**H$_B$), 4.97 (1H, s, =CH$_A$**H$_B$**), 3.05 (3H, s, SC**H$_3$**), 2.80–2.66 (2H, m, PhC**H$_2$**), 2.64–2.47 (5H, m, C**H$_2$**CH$_2$C**H$_2$** and PhCH$_2$C**H$_A$**H$_B$), 2.40 (1H, ddd, *J* = 14.5, 10.5, 6.2 Hz, PhCH$_2$CH$_A$**H$_B$**), 2.12–1.96 (2H, m, CH$_2$C**H$_2$**CH$_2$), 1.81 (3H, s, CC**H$_3$**); $^{13}$C NMR (100.6 MHz, $CDCl_3$) δ 195.8 (C), 174.1 (C), 140.9 (C), 140.3 (C), 138.2 (C), 134.4 (CH), 132.7 (C), 132.7 (C), 129.6 (CH), 128.5 (2 × CH), 128.2 (2 × CH), 127.0 (CH), 126.1 (CH), 126.0 (CH), 122.7 (CH), 119.1 ($CH_2$), 110.6 (C), 84.3 (C), 44.5 ($CH_3$), 40.7 ($CH_2$), 38.3 ($CH_2$), 30.1 ($CH_2$), 29.6 ($CH_2$), 19.7 ($CH_2$), 18.4 ($CH_3$); HRMS (ESI +ve) Exact mass calculated for $C_{27}H_{29}O_4S$ $[M+H]^+$: 449.1781, found: 449.1786.

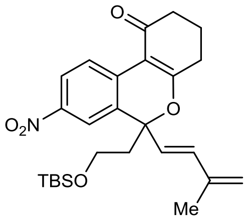


**6-{2-[(*tert*-Butyldimethylsilyl)oxy]ethyl}-6-[(*E*)-3-methylbuta-1,3-dien-1-yl]-9-nitro-1*H*,2*H*,3*H*,4*H*,6*H*-benzo[*c*]chromen-1-one (11b).** The title compound was prepared according to General Procedure C from 2-aryl-3-hydroxy-2-cyclohexenone **1g** (117 mg, 0.50 mmol) and enyne **2b** (131 mg, 0.55 mmol) at 60 °C for a reaction time of 3 h and was purified by flash column chromatography (3:1 petroleum ether:EtOAc) to give a yellow oil (197 mg, 84%). $R_f$ 0.57 (2:1 petroleum ether:EtOAc); IR 1664 (C=O), 1597 (C=C), 1511 ($NO_2$), 1376, 1334, 1070, 837, 740, 668 $cm^{-1}$; $^1$H NMR (400 MHz, $CDCl_3$) δ 8.70 (1H, d, *J* = 8.9 Hz, Ar**H**), 8.13 (1H, dd, *J* = 8.9, 2.4 Hz, Ar**H**), 7.99 (1H, d, *J* = 2.4 Hz, Ar**H**), 6.01 (1H, d, *J* = 15.9 Hz, C**H**=CHCCH$_3$), 5.75 (1H, d, *J* = 15.9 Hz, =C**H**CCH$_3$), 5.03 (1H, s, =C**H$_A$**H$_B$), 4.94 (1H, s, =CH$_A$**H$_B$**), 3.84–3.66 (2H, m, OC**H$_2$**), 2.69–2.59 (2H, m, C**H$_2$**CH$_2$CH$_2$), 2.59–2.44 (3H, m, CH$_2$CH$_2$C**H$_2$** and OCH$_2$C**H$_A$**H$_B$), 2.42–2.27 (1H, m, OCH$_2$CH$_A$**H$_B$**), 2.11–1.99 (2H, m, CH$_2$C**H$_2$**CH$_2$), 1.81 (3H, s, CC**H$_3$**), 0.84 (9H, s, C(C**H$_3$**)$_3$), –0.01 (3H, s, SiC**H$_3$**), –0.02 (3H, s, SiC**H$_3$**); $^{13}$C NMR (100.6 MHz, $CDCl_3$) δ 195.7 (C), 174.2 (C), 146.0 (C), 140.2 (C), 134.5 (C), 133.5 (CH), 132.7 (C), 129.7 (CH), 125.8 (CH), 123.2 (CH), 119.6 (CH), 119.3 ($CH_2$), 110.7 (C), 83.5 (C), 58.5 ($CH_2$), 41.4 ($CH_2$), 38.3 ($CH_2$), 29.8 ($CH_2$), 25.8 (3 × $CH_3$), 19.7 ($CH_2$), 18.4 ($CH_3$), 18.2 (C), –5.5 (2 × $CH_3$); HRMS (ESI +ve) Exact mass calculated for $C_{26}H_{36}NO_5Si$ $[M+H]^+$: 470.2357, found: 470.2346.

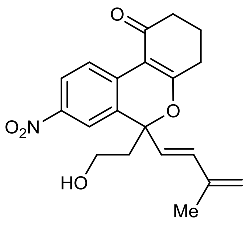


**6-(2-Hydroxyethyl)-6-[(*E*)-3-methylbuta-1,3-dien-1-yl]-9-nitro-1*H*,2*H*,3*H*,4*H*,6*H*-benzo[*c*]chromen-1-one (11c).** The title compound was prepared according to General Procedure C from 2-aryl-3-hydroxy-2-cyclohexenone **1g** (117 mg, 0.50 mmol) and enyne **2c** (68.3 mg, 0.55 mmol) at 90 °C for a reaction time of 3 h and was purified by flash column chromatography (2:1 petroleum ether:EtOAc) to give a yellow oil (110 mg, 62%). $R_f$ 0.26 (2:1 EtOAc:petroleum ether); IR 1664 (C=O), 1596 (C=C), 1512 ($NO_2$), 1377, 1335, 701, 843, 743 $cm^{-1}$; $^1H$ NMR (400 MHz, $CDCl_3$) δ 8.69 (1H, d, *J* = 8.8 Hz, Ar**H**), 8.14 (1H, dd, *J* = 8.8, 2.0 Hz, Ar**H**), 7.97 (1H, d, *J* = 2.0 Hz, Ar**H**), 6.05 (1H, d, *J* = 15.8 Hz, C**H**=CHCCH3), 5.74 (1H, d, *J* = 15.8 Hz, =C**H**CCH3), 5.05 (1H, s, =C**H$_A$**H$_B$), 4.97 (1H, s, =CH$_A$**H$_B$**), 3.82 (2H, t, *J* = 6.2 Hz, OC**H**2), 2.73–2.61 (2H, m, C**H**2CH2CH2), 2.59–2.46 (3H, m, CH2CH2C**H**2 and OCH2C**H$_A$**H$_B$), 2.46–2.33 (1H, m, OCH2CH$_A$**H$_B$**), 2.12–1.92 (3H, m, O**H** and CH2C**H**2CH2), 1.82 (3H, s, C**H**3); $^{13}C$ NMR (100.6 MHz, $CDCl_3$) δ 195.9 (C), 174.2 (C), 146.1 (C), 140.1 (C), 134.7 (CH), 133.5 (C), 132.7 (C), 129.1 (CH), 126.0 (CH), 123.4 (CH), 119.6 ($CH_2$), 119.3 (CH), 110.9 (C), 83.8 (C), 58.3 ($CH_2$), 41.2 ($CH_2$), 38.3 ($CH_2$), 29.8 ($CH_2$), 19.7 ($CH_2$), 18.4 ($CH_3$); HRMS (ESI +ve) Exact mass calculated for $C_{20}H_{22}NO_5$ $[M+H]^+$: 356.1419, found: 356.1493.

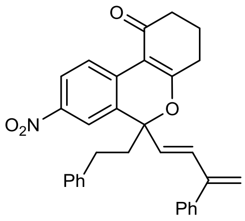


**9-Nitro-6-[(*E*)-3-phenylbuta-1,3-dien-1-yl]-6-(2-phenylethyl)-1*H*,2*H*,3*H*,4*H*,6*H*-benzo[*c*]chromen-1-one (11d).** The title compound was prepared according to General Procedure C from 2-aryl-3-hydroxy-2-cyclohexenone **1g** (117 mg, 0.50 mmol) and enyne **2d** (135 mg, 0.55 mmol) at 60 °C for a reaction time of 3 h and was purified by flash column chromatography (3:1 petroleum ether:EtOAc) to give a yellow oil (174 mg, 73%). $R_f$ 0.42 (2:1 petroleum ether:EtOAc); IR 1656 (C=O), 1597 (C=C), 1513 ($NO_2$), 1377, 1335, 701, 752, 699 $cm^{-1}$; $^1H$ NMR (400 MHz, $CDCl_3$) δ 8.80 (1H, d, *J* = 8.9 Hz, Ar**H**), 8.21 (1H, dd, *J* = 8.9, 2.4 Hz, Ar**H**), 8.02 (1H, d, *J* = 2.4 Hz, Ar**H**), 7.42–7.16 (10H, m, 10 × Ar**H**), 6.27 (1H, d, *J* = 15.8 Hz, C**H**=CHCPh), 5.81 (1H, d, *J* = 15.8 Hz, =C**H**CPh), 5.30–5.29 (1H, m, =C**H$_A$**H$_B$), 5.28–5.27 (1H, m, =CH$_A$**H$_B$**), 2.88–2.70 (2H, m, PhC**H**2), 2.70–2.51 (5H, m, C**H**2CH2C**H**2 and PhCH2C**H$_A$**H$_B$), 2.40 (1H, ddd, *J* = 14.6, 10.4, 6.2 Hz, PhCH2CH$_A$**H$_B$**), 2.16–1.99 (2H, m, CH2C**H**2CH2); $^{13}C$ NMR (100.6 MHz, $CDCl_3$) δ 195.8 (C), 174.5 (C), 146.2 (C), 146.1 (C), 140.8 (C), 139.3 (C), 133.8 (C), 133.4 (CH), 133.0 (CH), 132.5 (C), 128.5 (2 × CH), 128.33 (2 × CH), 128.27 (2 × CH), 128.0 (2 × CH), 127.8 (CH), 126.2 (CH), 126.0 (CH), 123.4 (CH), 119.4 (CH), 119.0 ($CH_2$), 110.8 (C), 84.4 (C), 40.5 ($CH_2$), 38.3 ($CH_2$), 30.2 ($CH_2$), 29.7 ($CH_2$), 19.7 ($CH_2$); HRMS (ESI +ve) Exact mass calculated for $C_{31}H_{28}NO_4$ $[M+H]^+$: 478.2013, found: 478.2005.

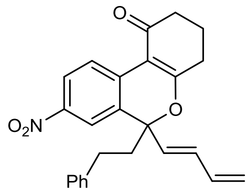

**6-[(*E*)-Buta-1,3-dien-1-yl]-9-nitro-6-(2-phenylethyl)-1*H*,2*H*,3*H*,4*H*,6*H*-benzo[*c*]chromen-1-one (11e).** The title compound was prepared according to General Procedure C from 2-aryl-3-hydroxy-2-cyclohexenone **1g** (117 mg, 0.50 mmol) and enyne **2e** (93.5 mg, 0.55 mmol) at 60 °C for a reaction time of 3 h and was purified by flash column chromatography (3:1 petroleum ether:EtOAc) to give a yellow oil (128 mg, 64%). $R_f$ 0.49 (2:1 petroleum ether:EtOAc); IR 1654 (C=O), 1596 (C=C), 1516 ($NO_2$), 1378, 1337, 1002, 733, 646 $cm^{-1}$; $^1$H NMR (400 MHz, $CDCl_3$) δ 8.77 (1H, d, *J* = 8.9 Hz, Ar**H**), 8.17 (1H, dd, *J* = 8.9, 2.4 Hz, Ar**H**), 8.00 (1H, d, *J* = 2.4 Hz, Ar**H**), 7.37–7.24 (2H, m, 2 × Ar**H**), 7.25–7.10 (3H, m, 3 × Ar**H**), 6.32 (1H, dt, *J* = 16.9, 10.2 Hz, C**H**=$CH_2$), 5.98 (1H, dd, *J* = 15.5, 10.2 Hz, =C**H**CH=$CH_2$), 5.85 (1H, d, *J* = 15.5 Hz, C**H**=CHCH=$CH_2$), 5.23 (1H, d, *J* = 16.9 Hz, =C$\mathbf{H_A}$$H_B$), 5.20 (1H, d, *J* = 10.2 Hz, =C$H_A$$\mathbf{H_B}$), 2.81–2.68 (2H, m, PhC$\mathbf{H_2}$), 2.68–2.48 (5H, m, C$\mathbf{H_2}$$CH_2$C$\mathbf{H_2}$ and Ph$CH_2$C$\mathbf{H_A}$$H_B$), 2.41 (1H, ddd, *J* = 14.6, 10.4, 6.2 Hz, Ph$CH_2$C$H_A$$\mathbf{H_B}$), 2.12–1.96 (2H, m, $CH_2$C$\mathbf{H_2}$$CH_2$); $^{13}$C NMR (100.6 MHz, $CDCl_3$) δ 195.8 (C), 174.4 (C), 146.0 (C), 140.8 (C), 135.2 (CH), 133.8 (C), 133.5 (CH), 132.6 (CH), 132.3 (C), 128.5 (2 × CH), 128.2 (2 × CH), 126.1 (CH), 126.0 (CH), 123.3 (CH), 120.1 ($CH_2$), 119.3 (CH), 110.6 (C), 84.1 (C), 40.4 ($CH_2$), 38.3 ($CH_2$), 30.1 ($CH_2$), 29.7 ($CH_2$), 19.7 ($CH_2$); HRMS (ESI +ve) Exact mass calculated for $C_{25}H_{24}NO_4$ $[M+H]^+$: 402.1700, found: 402.1698.

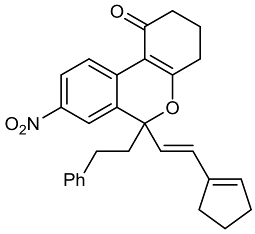

**6-[(*E*)-2-(Cyclopent-1-en-1-yl)ethenyl]-9-nitro-6-(2-phenylethyl)-1*H*,2*H*, 3*H*,4*H*,6*H*-benzo[*c*]chromen-1-one (11f).** To a microwave vial, was added 2-aryl-3-hydroxy-2-cyclohexenone **1g** (86.3 mg, 0.37 mmol), $[Cp^*RhCl_2]_2$ (7.7 mg, 13 μmol), $Cu(OAc)_2$ (191 mg, 1.05 mmol), AcOH (3 μL, 50 μmol) and enyne **2f** (85.6 mg, 0.41 mmol). The vessel was then sealed, flushed with $N_2$, and 1,4-dioxane (4 mL) was added. The reaction was then heated at 60 °C for 4 h. The reaction was cooled to room temperature, filtered through a short pad of silica using $Et_2O$ (25 mL) as eluent and concentrated *in vacuo*. Purification of the residue by flash column chromatography (3:1 petroleum ether:EtOAc) gave *benzoyran* **11f** (151 mg, 93%) as a yellow oil. $R_f$ 0.42 (2:1 petroleum ether:EtOAc); IR 1654 (C=O), 1596 (C=C), 1515 ($NO_2$), 1378, 1335, 733, 644 $cm^{-1}$; $^1$H NMR (400 MHz, $CDCl_3$) δ 8.77 (1H, d, *J* = 8.9 Hz, Ar**H**), 8.17 (1H, dd, *J* = 8.9, 2.4 Hz, Ar**H**), 8.00 (1H, d, *J* = 2.4 Hz, Ar**H**), 7.33–7.24 (2H, m, 2 × Ar**H**), 7.24–7.13 (3H, m, 3 × Ar**H**), 6.21 (1H, d, *J* = 15.7 Hz, OCC**H**=), 5.76 (1H, s, =C**H**$CH_2$), 5.62 (1H, d, *J* = 15.7 Hz, =C**H**C=CH), 2.86–2.67 (2H, m, PhC$\mathbf{H_2}$), 2.67–2.49 (5H, m, C$\mathbf{H_2}$$CH_2$C$\mathbf{H_2}$ and Ph$CH_2$C$\mathbf{H_A}$$H_B$), 2.49–2.31 (5H, m, =CHC$\mathbf{H_2}$$CH_2$C$\mathbf{H_2}$ and Ph$CH_2$C$H_A$$\mathbf{H_B}$), 2.14–1.98 (2H, m, CO$CH_2$C$\mathbf{H_2}$$CH_2$CO), 1.99–1.84 (2H, m, =CH$CH_2$C$\mathbf{H_2}$$CH_2$); $^{13}$C NMR (100.6 MHz, $CDCl_3$) δ 195.8 (C), 174.6 (C), 146.0 (C), 140.9 (C),

140.8 (C), 134.3 (CH), 133.9 (C), 132.6 (C), 129.5 (CH), 129.0 (CH), 128.5 (2 × CH), 128.3 (2 × CH), 126.1 (CH), 125.9 (CH), 123.2 (CH), 119.5 (CH), 110.5 (C), 84.6 (C), 40.7 ($CH_2$), 38.3 ($CH_2$), 32.9 ($CH_2$), 31.1 ($CH_2$), 30.2 ($CH_2$), 29.8 ($CH_2$), 22.9 ($CH_2$), 19.7 ($CH_2$); HRMS (ESI +ve) Exact mass calculated for $C_{28}H_{28}NO_4$ $[M+H]^+$: 373.1587, found: 373.1578.

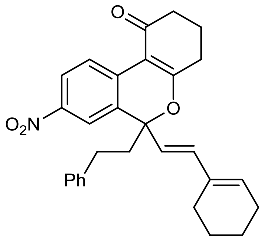


**6-[(*E*)-2-(Cyclohex-1-en-1-yl)ethenyl]-9-nitro-6-(2-phenylethyl)-1*H*,2*H*, 3*H*,4*H*,6*H*-benzo[*c*]chromen-1-one (11g).** The title compound was prepared according to General Procedure C from 2-aryl-3-hydroxy-2-cyclohexenone **1g** (117 mg, 0.50 mmol) and enyne **2g** (123 mg, 0.55 mmol) at 60 °C for a reaction time of 3 h and was purified by flash column chromatography (3:1 petroleum ether:EtOAc) to give a yellow oil (203 mg, 89%). $R_f$ 0.44 (2:1 petroleum ether:EtOAc); IR 1654 (C=O), 1596 (C=C), 1515 ($NO_2$), 1378, 1335, 752, 736 $cm^{-1}$; $^1H$ NMR (400 MHz, $CDCl_3$) δ 8.77 (1H, d, *J* = 8.9 Hz, Ar**H**), 8.16 (1H, dd, *J* = 8.9, 2.4 Hz, Ar**H**), 7.99 (1H, d, *J* = 2.4 Hz, Ar**H**), 7.32–7.24 (2H, m, 2 × Ar**H**), 7.23–7.14 (3H, m, 3 × Ar**H**), 5.96 (1H, d, *J* = 15.8 Hz, OCC**H**=), 5.75 (1H, t, *J* = 3.9 Hz, =C**H**$CH_2$), 5.63 (1H, d, *J* = 15.8 Hz, =C**H**C=CH), 2.83–2.67 (2H, m, PhC$\mathbf{H_2}$), 2.67–2.48 (5H, m, COC$\mathbf{H_2}$$CH_2$C$\mathbf{H_2}$CO and Ph$CH_2$C$\mathbf{H_A}$$H_B$), 2.48–2.37 (1H, m, Ph$CH_2$$CH_A$$\mathbf{H_B}$), 2.17–1.96 (6H, m, CO$CH_2$C$\mathbf{H_2}$$CH_2$CO and =CHC$\mathbf{H_2}$$CH_2$$CH_2$C$\mathbf{H_2}$), 1.72–1.62 (2H, m, =CH$CH_2$C$\mathbf{H_2}$$CH_2$$CH_2$), 1.62–1.53 (2H, m, =CH$CH_2$$CH_2$C$\mathbf{H_2}$$CH_2$); $^{13}C$ NMR (100.6 MHz, $CDCl_3$) δ 195.8 (C), 174.6 (C), 146.0 (C), 141.0 (C), 135.6 (CH), 134.1 (C), 133.9 (C), 132.9 (C), 132.8 (CH), 128.6 (2 × CH), 128.2 (2 × CH), 126.0 (CH), 125.8 (CH), 125.5 (CH), 123.1 (CH), 119.4 (CH), 110.5 (C), 84.7 (C), 40.8 ($CH_2$), 38.3 ($CH_2$), 30.2 ($CH_2$), 29.8 ($CH_2$), 25.8 ($CH_2$), 24.4 ($CH_2$), 22.1 ($CH_2$), 22.1 ($CH_2$), 19.7 ($CH_2$); HRMS (ESI +ve) Exact mass calculated for $C_{29}H_{30}NO_4$ $[M+H]^+$: 456.2169, found: 456.2190.

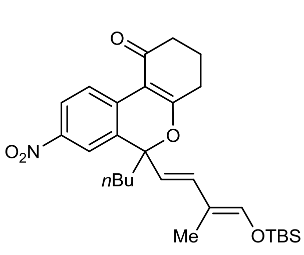


**6-Butyl-6-[(1*E*,3*E*)-4-[(*tert*-butyldimethylsilyl)oxy]-3-methylbuta-1,3-dien-1-yl]-8-nitro-1*H*,2*H*,3*H*,4*H*,6-benzo[*c*]chromen-1-one (11h).** The title compound was prepared according to General Procedure C from 2-aryl-3-hydroxy-2-cyclohexenone **1g** (117 mg, 0.50 mmol) and enyne **2h** (146 mg, 0.55 mmol) at 60 °C for a reaction time of 1 h and was purified by flash column chromatography (3:1 petroleum ether:EtOAc) to give a yellow oil (152 mg, 61%). Product was unstable on prolonged exposure to silica and hence was purified using the briefest column possible. $R_f$ 0.63 (2:1 petroleum ether:EtOAc); IR 1643 (C=O), 1595 (C=C), 1515 ($NO_2$), 1378, 1338, 1178, 838, 732 $cm^{-1}$; $^1H$ NMR (400 MHz, $CDCl_3$) δ 8.72 (1H, d, *J* = 8.9 Hz, Ar**H**), 8.14 (1H, dd, *J* = 8.9, 2.4 Hz, Ar**H**), 7.91 (1H, d, *J* = 2.4 Hz, Ar**H**), 6.30 (1H, s, =C**H**OTBS), 5.77

(1H, d, $J$ = 15.5 Hz, $C\mathbf{H}{=}CHCCH_3$), 5.52 (1H, d, $J$ = 15.5 Hz, $=C\mathbf{H}CCH_3$), 2.70–2.43 (4H, m, $COC\mathbf{H}_2CH_2C\mathbf{H}_2CO$), 2.29–2.14 (1H, m, $CH_3CH_2CH_2C\mathbf{H_A}H_B$), 2.14–1.96 (3H, m, $COCH_2C\mathbf{H}_2CH_2CO$ and $CH_3CH_2CH_2CH_A\mathbf{H_B}$), 1.68 (3H, d, $J$ = 1.2 Hz, $CC\mathbf{H}_3$), 1.43–1.24 (4H, m, $CH_3C\mathbf{H}_2C\mathbf{H}_2$), 0.93–0.88 (3H, m, $C\mathbf{H}_3CH_2$), 0.90 (9H, s, $C(C\mathbf{H}_3)_3$), 0.13 (3H, s, $SiC\mathbf{H}_3$), 0.13 (3H, s, $SiC\mathbf{H}_3$); $^{13}C$ NMR (100.6 MHz, $CDCl_3$) δ 195.9 (C), 175.0 (C), 145.9 (C), 143.8 (CH), 134.2 (C), 133.3 (C), 133.3 (CH), 125.6 (CH), 124.6 (CH), 123.0 (CH), 119.8 (CH), 116.2 (C), 110.5 (C), 85.5 (C), 39.1 ($CH_2$), 38.4 ($CH_2$), 30.1 ($CH_2$), 26.0 ($CH_2$), 25.4 (3 × $CH_3$), 22.8 ($CH_2$), 19.8 ($CH_2$), 18.0 (C), 13.9 ($CH_2$), 9.3 ($CH_3$), –5.3 ($CH_3$), –5.4 ($CH_3$); HRMS (ESI +ve) Exact mass calculated for $C_{28}H_{39}NO_5Si$ $[M+H]^+$: 498.2670, found: 498.2683.

### *by NOESY NMR*

The presence of $H_b$–$H_c$ and $H_a$–Me enhancements, combined with the absence of $H_a$–$H_b$ and $H_c$–Me enhancements provides strong evidence in support of the diene geometry .

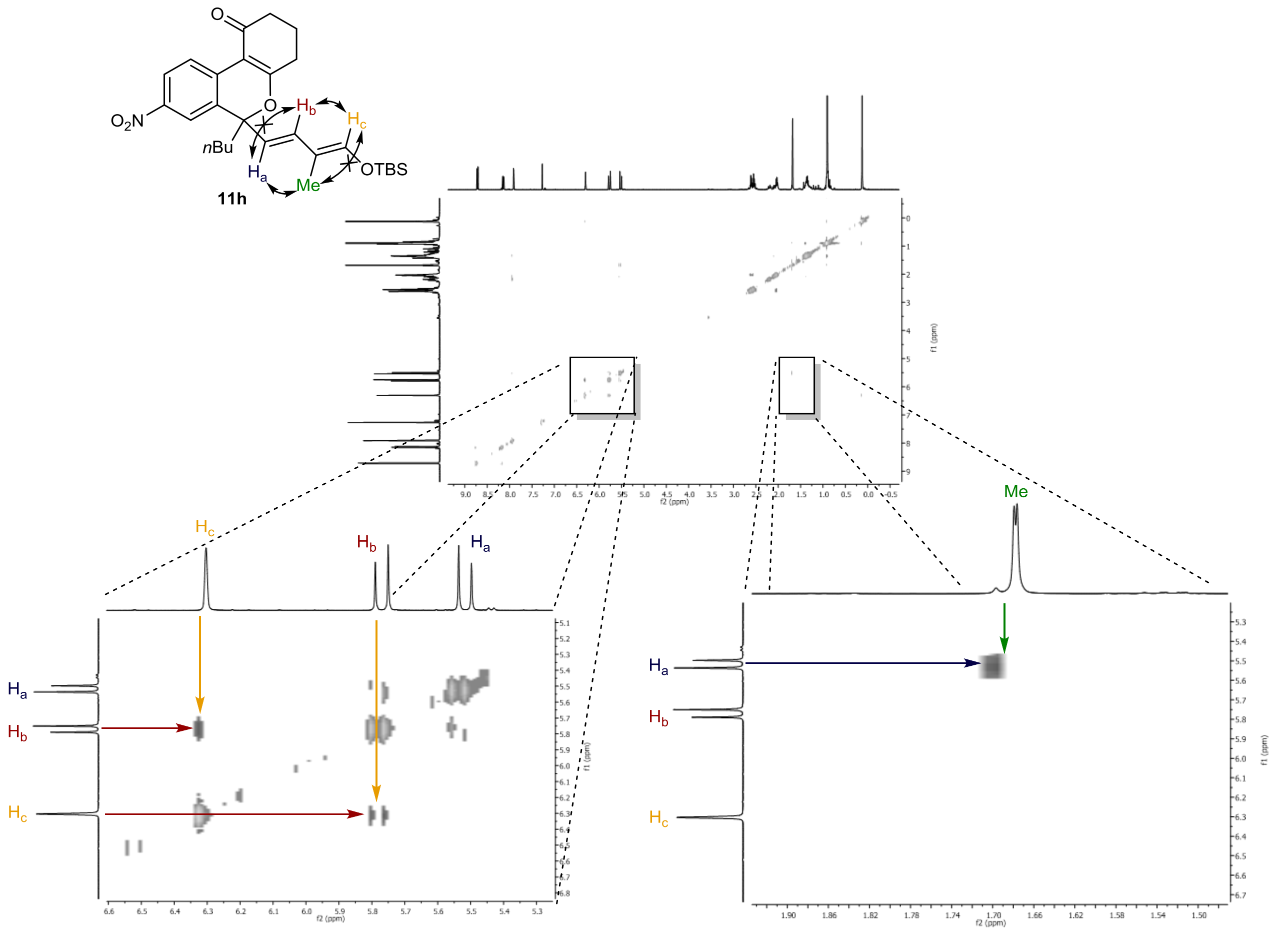

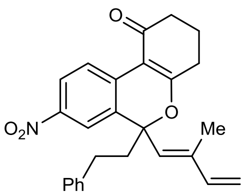


**6-[(*E*)-2,3-Dimethylbuta-1,3-dien-1-yl]-8-nitro-6-(2-phenylethyl)-1*H*,2*H*,3*H*,4*H*,6*H*-benzo[*c*]chromen-1-one (11i).** The title compound was prepared according to General Procedure C from 2-aryl-3-hydroxy-2-cyclohexenone **1g** (117 mg, 0.50 mmol) and enyne **2i** (101 mg, 0.55 mmol) at 60 °C for a reaction time of 3 h and was purified by flash column chromatography (3:1 petroleum ether:EtOAc) to give a yellow oil (127 mg, 61%). $R_f$ 0.42 (2:1 petroleum ether:EtOAc); IR 1654 (C=O), 1595 (C=C), 1515 ($NO_2$), 1376, 1336, 908, 732 cm$^{-1}$; $^1$H NMR (400 MHz, $CDCl_3$) δ 8.78 (1H, d, *J* = 8.9 Hz, Ar**H**), 8.13 (1H, dd, *J* = 8.9, 2.4 Hz, Ar**H**), 7.91 (1H, d, *J* = 2.4 Hz, Ar**H**), 7.31–7.24 (2H, m, 2 × Ar**H**), 7.22–7.16 (1H, m, Ar**H**), 7.15–7.10 (2H, m, 2 × Ar**H**), 6.49 (1H, dd, *J* = 17.4, 11.0 Hz, C**H**=$CH_2$), 5.99 (1H, s, C**H**=$CCH_3$), 5.34 (1H, d, *J* = 17.4 Hz, =C$\mathbf{H_A}$$H_B$), 5.20 (1H, d, *J* = 11.0 Hz, =C$H_A$$\mathbf{H_B}$), 2.78–2.65 (2H, m, PhC$\mathbf{H_2}$), 2.65–2.47 (3H, m, C$\mathbf{H_2}$$CH_2CH_2$ and Ph$CH_2$C$\mathbf{H_A}$$H_B$), 2.47–2.35 (2H, m, $CH_2CH_2$C$\mathbf{H_2}$), 2.31–2.19 (1H, m, Ph$CH_2$C$H_A$$\mathbf{H_B}$), 2.09–1.97 (2H, m, $CH_2$C$\mathbf{H_2}$$CH_2$), 1.64 (3H, d, *J* = 1.1 Hz, C$\mathbf{H_3}$); $^{13}$C NMR (100.6 MHz, $CDCl_3$) δ 195.8 (C), 174.4 (C), 146.2 (C), 142.6 (C), 140.5 (C), 140.3 (CH), 134.7 (C), 133.1 (C), 131.8 (CH), 128.6 (2 × CH), 128.2 (2 × CH), 126.2 (CH), 125.6 (CH), 123.2 (CH), 119.7 (CH), 115.2 ($CH_2$), 110.1 (C), 83.9 (C), 42.7 ($CH_2$), 38.4 ($CH_2$), 29.7 ($CH_2$), 29.5 ($CH_2$), 19.8 ($CH_2$), 13.8 ($CH_3$); HRMS (ESI +ve) Exact mass calculated for $C_{26}H_{25}NNaO_4$ $[M+Na]^+$: 438.1676, found: 438.1672.

***by NOESY NMR***

The $H_a$–$H_b$ enhancement suggests the shown diene conformation while the absence of an $H_a$–Me enhancement provides strong evidence in support of the diene geometry.

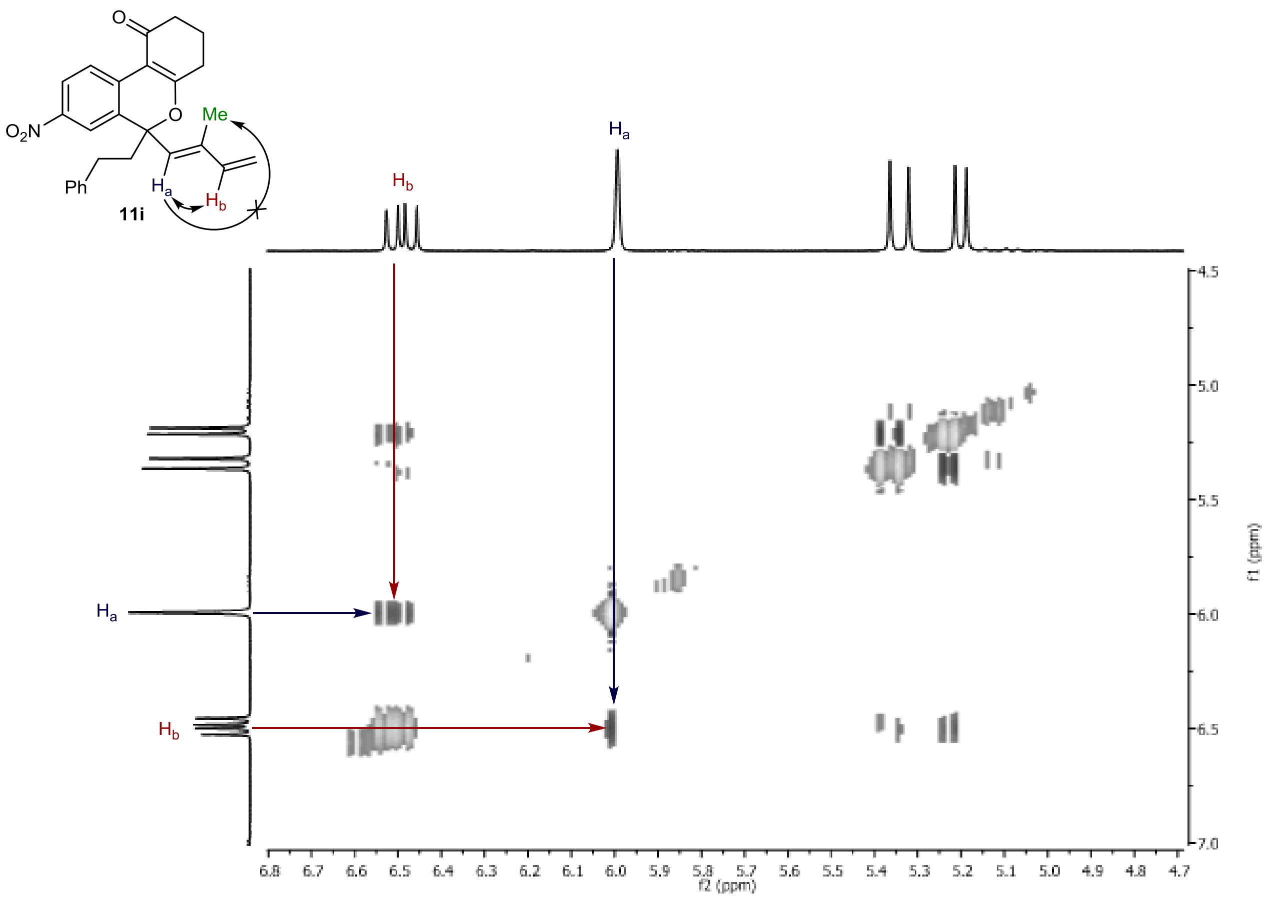


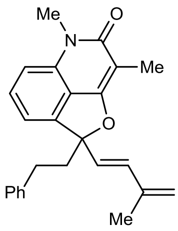


**9,11-Dimethyl-3-[(*E*)-3-methylbuta-1,3-dien-1-yl]-3-(2-phenylethyl)-2-oxa-9-azatricyclo[6.3.1.0$^{4,12}$]dodeca-1(11),4,6,8(12)-tetraen-10-one (13a).** The title compound was prepared according to General Procedure C from hydroxyquinolinone **12a**[10] (94.5 mg, 0.50 mmol) and enyne **2a** (101 mg, 0.55 mmol) at 90 °C for a reaction time of 3 h and was purified by flash column chromatography ($Et_2O$) to give a yellow oil (123 mg, 64%). $R_f$ 0.28 ($Et_2O$); IR 1671 (C=C), 1620 (C=O), 1595, 1493, 1394, 1139, 748, 730 635 cm$^{-1}$; $^1$H NMR (400 MHz, $CDCl_3$) δ 7.57 (1H, dd, *J* = 8.0, 7.3 Hz, Ar**H**), 7.27–7.20 (2H, m, 2 × Ar**H**), 7.20–7.07 (4H, m, 4 × Ar**H**), 7.02 (1H, d, *J* = 7.3 Hz, Ar**H**), 6.39 (1H, d, *J* = 15.9 Hz, C**H**=CHCC$H_3$), 5.97 (1H, d, *J* = 15.9 Hz, =C**H**CC$H_3$), 5.05 (1H, s, *J* = 8.4 Hz, =C**$H_A$**$H_B$), 5.02 (1H, s, =C$H_A$**$H_B$**), 3.64 (3H, s, NC**$H_3$**), 2.76–2.62 (1H, m, PhC$H_2$C**$H_A$**$H_B$), 2.62–2.48 (1H, m, PhC$H_2$C$H_A$**$H_B$**), 2.47–2.32 (2H, m, PhC**$H_2$**), 2.15 (3H, s,

10. Burns, A. R.; Solana González, J.; Lam, H. W. *Angew. Chem., Int. Ed.* **2012**, *51*, 10827–10831.

$CH_3CC{=}O$), 1.86 (3H, s, $CH_3C{=}CH_2$); $^{13}C$ NMR (100.6 MHz, $CDCl_3$) δ 166.5 (C), 162.3 (C), 142.0 (C), 141.0 (C), 140.7 (C), 136.0 (C), 133.5 (CH), 131.7 (CH), 128.7 (CH), 128.3 (2 × CH), 128.2 (2 × CH), 125.9 (CH), 118.8 ($CH_2$), 118.0 (C), 113.8 (CH), 111.1 (CH), 101.7 (C), 98.8 (C), 41.5 ($CH_2$), 29.7 ($CH_2$), 29.1 ($CH_3$), 18.4 ($CH_3$), 9.5 ($CH_3$); HRMS (ESI +ve) Exact mass calculated for $C_{25}H_{26}NO_2$ $[M+H]^+$: 372.1958, found: 372.1962.

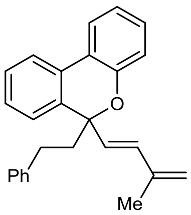


**6-[(*E*)-3-Methylbuta-1,3-dien-1-yl]-6-(2-phenylethyl)-6*H*-benzo[*c*]benzopyran (13b).** To a microwave vial, was added 2-phenylphenol (**12a**, 85.0 mg, 0.50 mmol), $K_2CO_3$ (207 mg, 1.50 mmol), $[Cp^*RhCl_2]_2$ (7.7 mg, 13 μmol), $Cu(OAc)_2$ (191 mg, 1.05 mmol), AcOH (3 μL, 50 μmol) and enyne **2a** (101 mg, 0.55 mmol). The vessel was then sealed, flushed with $N_2$, and 1,4-dioxane (5 mL) was added. The reaction was then heated at 120 °C for 1 h. Then second loading of 2.5 mol% (7.7 mg) $[Cp^*RhCl_2]_2$ in 1,4-dioxane 1 mL was added to the reaction and the mixture was heated at 120 °C for a further 2.5 h. The reaction was cooled to room temperature, filtered through a short pad of silica using $Et_2O$ (25 mL) as eluent and concentrated *in vacuo*. Purification of the residue by flash column chromatography (50:1 petroleum ether:$Et_2O$) gave *benzopyran* **13b** (132 mg, 75%) as a yellow oil. $R_f$ 0.53 (10:1 petroleum ether:$Et_2O$); IR 1496, 1453, 1437, 1248, 967, 748, 698 $cm^{-1}$; $^1H$ NMR (400 MHz, $CDCl_3$) δ 7.80 (1H, dd, $J$ = 7.6, 1.5 Hz, Ar**H**), 7.75 (1H, dd, $J$ = 7.6, 1.5 Hz, Ar**H**), 7.40 (1H, td, $J$ = 7.5, 1.6 Hz, Ar**H**), 7.34 (1H, td, $J$ = 7.5, 1.6 Hz, Ar**H**), 7.31–7.24 (4H, m, 4 × Ar**H**), 7.21–7.15 (3H, m, 3 × Ar**H**), 7.08–7.02 (2H, m, 2 × Ar**H**), 6.22 (1H, d, $J$ = 15.9 Hz, C**H**=$CHCCH_3$), 5.88 (1H, d, $J$ = 15.9 Hz, =C**H**$CCH_3$), 4.99–4.94 (1H, m, =$C\mathbf{H_A}H_B$), 4.94–4.89 (1H, m, =$CH_A\mathbf{H_B}$), 2.93–2.72 (2H, m, $PhC\mathbf{H_2}$), 2.49 (1H, ddd, $J$ = 14.2, 12.0, 5.0 Hz, $PhCH_2C\mathbf{H_A}H_B$), 2.31 (1H, ddd, $J$ = 14.2, 12.0, 5.0 Hz, $PhCH_2CH_A\mathbf{H_B}$), 1.81 (3H, d, $J$ = 0.5 Hz, $C\mathbf{H_3}$); $^{13}C$ NMR (100.6 MHz, $CDCl_3$) δ 152.7 (C), 142.3 (C), 141.1 (C), 135.9 (C), 133.6 (CH), 132.2 (CH), 129.5 (C and CH), 128.3 (2 × [2 × CH]), 127.9 (CH), 127.8 (CH), 125.73 (CH), 125.0 (CH), 122.9 (CH), 122.3 (CH), 122.1 (C), 121.6 (CH), 117.9 (CH), 117.5 ($CH_2$), 81.5 (C), 41.0 ($CH_2$), 30.4 ($CH_2$), 18.5 ($CH_3$); HRMS (ESI +ve) Exact mass calculated for $C_{26}H_{24}NaO$ $[M+Na]^+$: 375.1719, found: 375.1730.

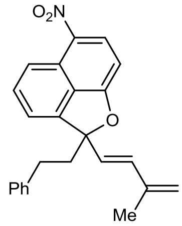


**3-[(*E*)-3-Methylbuta-1,3-dien-1-yl]-9-nitro-3-(2-phenylethyl)-2-oxatricyclo[6.3.1.0$^{4,12}$]dodeca-1(11),4(12),5,7,9-pentaene (13c).** The title compound was prepared according to General Procedure C from 4-nitronaphthalen-1-ol (**12c**, 94.5 mg, 0.50 mmol) and enyne **2a** (101 mg, 0.55 mmol) at 60 °C for a reaction time of 2 h and was purified by flash column chromatography (10:1 petroleum ether:EtOAc) to give a red oil (107 mg, 58%). $R_f$ 0.18 (10:1 petroleum ether:EtOAc); IR

1630, 1591, 1508, 1489 1311, 1265, 907, 811, 732 $cm^{-1}$; $^{1}H$ NMR (400 MHz, $CDCl_3$) δ 8.81 (1H, d, *J* = 8.5 Hz, Ar**H**), 8.75 (1H, d, *J* = 8.5 Hz, Ar**H**), 7.89 (1H, dd, *J* = 8.5, 7.0 Hz, Ar**H**), 7.41 (1H, d, *J* = 7.0 Hz, Ar**H**), 7.31–7.23 (2H, m, 2 × Ar**H**), 7.23–7.17 (1H, m, Ar**H**), 7.15–7.08 (2H, m, 2 × Ar**H**), 6.84 (1H, d, *J* = 8.5 Hz, Ar**H**), 6.43 (1H, d, *J* = 15.8 Hz, C**H**=$CHCCH_3$), 6.03 (1H, d, *J* = 15.8 Hz, =C**H**$CCH_3$), 5.10 (1H, s, =$C\mathbf{H_A}H_B$), 5.06 (1H, s, =$CH_A\mathbf{H_B}$), 2.80–2.57 (2H, m, $PhC\mathbf{H_2}$), 2.52–2.38 (2H, m, $PhCH_2C\mathbf{H_2}$), 1.89 (3H, s, $C\mathbf{H_3}$); $^{13}C$ NMR (100.6 MHz, $CDCl_3$) δ 165.9 (C), 143.1 (C), 140.7 (C), 140.5 (C), 135.5 (C), 134.1 (CH), 132.61 (CH), 132.57 (CH), 128.4 (C and 2 × CH), 128.1 (2 × CH), 127.9 (CH), 126.0 (CH), 125.5 (C), 122.9 (CH), 119.2 ($CH_2$), 118.1 (CH), 100.7 (CH), 99.7 (C), 41.8 ($CH_2$), 29.6 ($CH_2$), 18.4 ($CH_3$); HRMS (ESI +ve) Exact mass calculated for $C_{24}H_{21}NNaO_3$ $[M+Na]^+$: 394.1414, found: 394.1400.

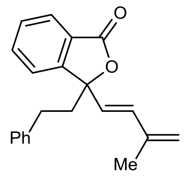

**3-[(*E*)-3-Methylbuta-1,3-dien-1-yl]-3-(2-phenylethyl)-1,3-dihydro-2-benzofuran-1-one (13d).** The title compound was prepared according to General Procedure C from benzoic acid (**12d**, 61.0 mg, 0.50 mmol) and enyne **2a** (101 mg, 0.55 mmol) at 120 °C for a reaction time of 1 h and was purified by flash column chromatography (10:1 petroleum ether:acetone) to give a yellow oil (99 mg, 65%). $R_f$ 0.17 (10:1 petroleum ether:acetone); IR 1760 (C=O), 1611, 1285, 1096, 752, 698 $cm^{-1}$; $^{1}H$ NMR (400 MHz, $CDCl_3$) δ 7.95 (1H, dt, *J* = 7.5, 1.0 Hz, Ar**H**), 7.73 (1H, td, *J* = 7.5, 1.0 Hz, Ar**H**), 7.57 (1H, td, *J* = 7.5, 1.0 Hz, Ar**H**), 7.47 (1H, dt, *J* = 7.5, 1.0 Hz, Ar**H**), 7.30–7.24 (2H, m, 2 × Ar**H**), 7.22–7.16 (1H, m, Ar**H**), 7.17–7.10 (2H, m, 2 × Ar**H**), 6.50 (1H, d, *J* = 15.8 Hz, C**H**=$CHCCH_3$), 5.90 (1H, d, *J* = 15.8 Hz, =C**H**$CCH_3$), 5.11–5.07 (1H, m, =$C\mathbf{H_A}H_B$), 5.07–5.04 (1H, m, =$CH_A\mathbf{H_B}$), 2.73 (1H, td, *J* = 12.0, 4.3 Hz, $PhC\mathbf{H_A}H_B$), 2.53 (1H, ddd, *J* = 13.4, 12.0, 4.3 Hz, $PhCH_2C\mathbf{H_A}H_B$), 2.46–2.36 (1H, m, $PhCH_A\mathbf{H_B}$), 2.30 (ddd, *J* = 13.4, 12.0, 4.3 Hz, $PhCH_2CH_A\mathbf{H_B}$), 1.86 (3H, dd, *J* = 1.2, 0.7 Hz, $C\mathbf{H_3}$); $^{13}C$ NMR (100.6 MHz, $CDCl_3$) δ 169.8 (C), 151.7 (C), 140.8 (C), 140.5 (C), 134.2 (CH), 133.2 (CH), 129.2 (CH), 128.4 (2 × CH), 128.2 (2 × CH), 127.8 (CH), 126.0 (CH), 125.9 (CH), 125.7 (C), 121.6 (CH), 118.8 ($CH_2$), 88.6 (C), 40.9 ($CH_2$), 29.7 ($CH_2$), 18.3 ($CH_3$); HRMS (ESI +ve) Exact mass calculated for $C_{21}H_{21}O_2$ $[M+H]^+$: 305.1536, found: 305.1521.

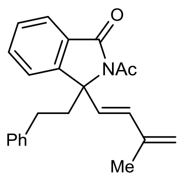

**6-[(*E*)-3-Methylbuta-1,3-dien-1-yl]-6-(2-phenylethyl)-6*H*-benzo[*c*]benzopyran (13e).** The title compound was prepared according to a modification of General Procedure C (in that of 5.0 mol% of $[Cp^*RhCl_2]_2$ (15.4 mg, 26 μmol) was used) from *N*-benzoylacetamide[11] (**12e**, 81.5 mg, 0.50 mmol) and enyne **2a** (101 mg, 0.55 mmol) at 120 °C for a reaction time of 2.5 h and was purified by flash column chromatography

11. Lee, J.; Hong, M.; Jung, Y.; Cho, E. J.; Rhe, H. *Tetrahedron*, **2012**, *68*, 2045–2051.

(10:1 petroleum ether:acetone) to give a yellow solid (110 mg, 64%). $R_f$ 0.53 (10:1 petroleum ether:$Et_2O$); m.p. 102–103 °C ($CH_2Cl_2$/petroleum ether); IR 1730 (C=O), 1696 (C=O), 1369, 1313, 1271, 698 $cm^{-1}$; $^1$H NMR (400 MHz, $CDCl_3$) δ 7.96 (1H, d, *J* = 7.6 Hz, Ar**H**), 7.73 (1H, td, *J* = 7.6, 1.0 Hz, Ar**H**), 7.56 (1H, td, *J* = 7.5, 1.0 Hz, Ar**H**), 7.40 (1H, dt, *J* = 7.6, 1.0 Hz, Ar**H**), 7.24–7.18 (2H, m, 2 × Ar**H**), 7.17–7.11 (1H, m, Ar**H**), 6.99–6.94 (2H, m, 2 × Ar**H**), 6.10 (1H, d, *J* = 16.1 Hz, C**H**=CHC$CH_3$), 6.02 (1H, d, *J* = 16.1 Hz, =C**H**C$CH_3$), 5.00–4.94 (1H, m, =C$\mathbf{H_A}H_B$), 4.92–4.86 (1H, m, =C$H_A\mathbf{H_B}$), 3.29 (1H, ddd, *J* = 13.8, 11.0, 6.0 Hz, PhC$\mathbf{H_A}H_B$), 2.60 (3H, s, COC$\mathbf{H}_3$), 2.36 (1H, ddd, *J* = 13.8, 11.0, 4.6 Hz, PhC$H_A\mathbf{H_B}$), 2.18 (1H, ddd, *J* = 13.6, 11.0, 6.0 Hz, Ph$CH_2$C$\mathbf{H_A}H_B$), 1.93 (1H, ddd, *J* = 13.6, 11.0, 4.6 Hz, Ph$CH_2$C$H_A\mathbf{H_B}$), 1.81–1.78 (3H, m, C$\mathbf{H}_3$C=$CH_2$); $^{13}$C NMR (100.6 MHz, $CDCl_3$) δ 170.8 (C), 168.4 (C), 148.8 (C), 141.2 (C), 140.6 (C), 134.6 (CH), 133.4 (CH), 130.9 (CH), 130.1 (C), 129.0 (CH), 128.4 (2 × CH), 128.2 (2 × CH), 126.0 (CH), 125.0 (CH), 122.8 (CH), 117.9 ($CH_2$), 70.5 (C), 37.5 ($CH_2$), 29.6 ($CH_2$), 26.4 ($CH_3$), 18.5 ($CH_3$); HRMS (ESI +ve) Exact mass calculated for $C_{23}H_{23}NNaO_2$ $[M+Na]^+$: 368.1621, found: 368.1622.

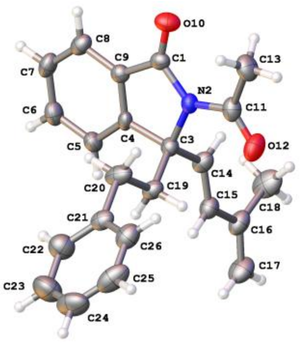


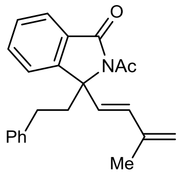


CCDC 1006281
**13e**

A single crystal was prepared by slow evaporation from a solution of **13e** in $CH_2Cl_2$. There is some disorder which is not shown, for clarity.

**6-[(*E*)-Buta-1,3-dien-1-yl]-8-nitro-6-(2-phenylethyl)-1*H*,2*H*,3*H*,4*H*,6*H*-benzo[*c*]chromen-1-one (11e) and 5'-nitro-3'-(2-phenylethyl)-2'-[(*E*)-prop-1-en-1-yl]spiro[cyclohexane-1,1'-indene]-2,6-dione (15)**

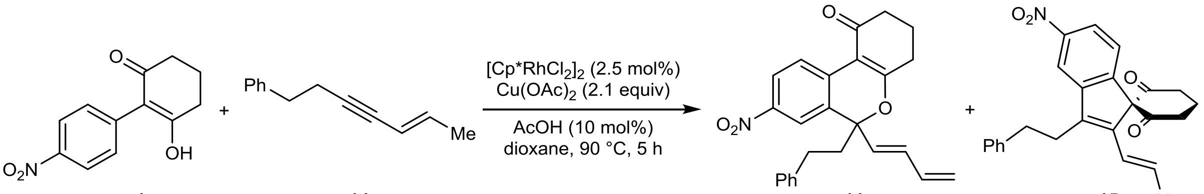


**1g** **14** **11e** **15**

General procedure C was followed using 2-aryl-3-hydroxy-2-cyclohexenone **1g** (117 mg, 0.50 mmol) and enyne **14** (93.5 mg, 0.55 mmol) at 90 °C for a reaction time of 5 h. Purification by flash column chromatography (3:1 petroleum ether:EtOAc) gave benzopyran **11e** (13.9 mg, 7%) as a yellow oil followed by *spiroindene* **15** (106 mg, 53%) as a yellow solid.

**For data of benzopyran 11e, see earlier**

**Data for spiroindene 15**

$R_f$ 0.14 (2:1 petroleum ether:EtOAc); m.p. 200–201 °C ($CH_2Cl_2$/petroleum ether); IR (film) 1723 (C=O), 1696 (C=C), 1526, 1346, 1194, 947, 736 $cm^{-1}$; $^1H$ NMR (400 MHz, $CDCl_3$) δ 8.01 (1H, dd, *J* = 8.3, 2.1 Hz, Ar**H**), 7.81 (1H, d, *J* = 2.1 Hz, Ar**H**), 7.51 (1H, d, *J* = 8.3 Hz, Ar**H**), 7.32–7.24 (4H, m, 4 × Ar**H**), 7.22–7.14 (1H, m, Ar**H**), 6.39 (1H, dq, *J* = 15.7, 1.4 Hz, C**H**=CHCH$_3$), 5.11 (1H, dq, *J* = 15.7, 6.7 Hz, =C**H**CH$_3$), 3.19–2.88 (8H, m, 4 × C**H**$_2$), 2.58–2.39 (1H, m, C**H**$_A$H$_B$), 2.26–2.06 (1H, m, C**H**$_A$H$_B$), 1.86 (3H, dd, *J* = 6.7, 1.4 Hz, C**H**$_3$); $^{13}C$ NMR (100.6 MHz, $CDCl_3$) δ 202.1 (2 × C), 148.6 (C), 147.8 (C), 146.7 (C), 144.5 (C), 141.6 (C), 140.8 (C), 130.5 (CH), 128.7 (2 × CH), 128.4 (2 × CH), 126.2 (CH), 122.6 (CH), 121.3 (CH), 120.5 (CH), 115.1 (CH), 82.5 (C), 39.2 ($CH_2$), 35.1 ($CH_2$), 27.5 ($CH_2$), 19.2 ($CH_3$), 17.3 ($CH_2$); HRMS (ESI +ve) Exact mass calculated for $C_{25}H_{23}NNaO_4$ $[M+Na]^+$: 424.1519, found: 424.1519; exact mass calculated for $C_{25}H_{24}NO_4$ $[M+H]^+$: 402.1700, found: 402.1703.

# 4. Deuterium Labeling Experiments

**Reversible C-H Functionlization:**

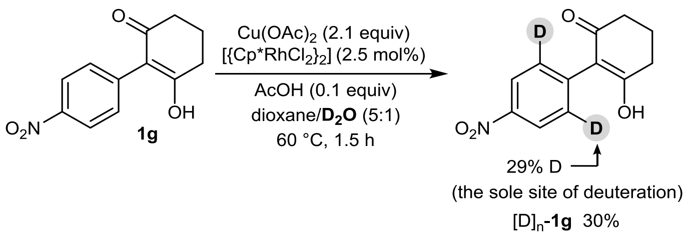


To a microwave vial, was added 2-aryl-3-hydroxy-2-cyclohexenone **1g** (23.4 mg, 0.10 mmol), $[Cp*RhCl_2]_2$ (1.5 mg, 2.6 µmol), and $Cu(OAc)_2$ (38.2 mg, 0.21 mmol). The vessel was then sealed, flushed with $N_2$, and to the vial was added 1,4-dioxane (1 mL) and $D_2O$ (200 µL). The reaction was then heated at 60 °C for 1.5 h. After cooling to room temperature 10% $HCl_{(aq)}$ (10 mL) and EtOAc (10 mL) were added, the layers were separated, and the aqueous layer was extracted with EtOAc (2 × 10 mL). The combined organic layers were dried ($MgSO_4$) and concentrated *in vacuo*. Purification of the residue by flash column chromatography (10:1 $CH_2Cl_2$:MeOH) gave 2-aryl-3-hydroxy-2-cyclohexenone $[D]_n$-**1g** (7.0 mg, 30%) as a yellow oil with 28% deuterium incorperation.

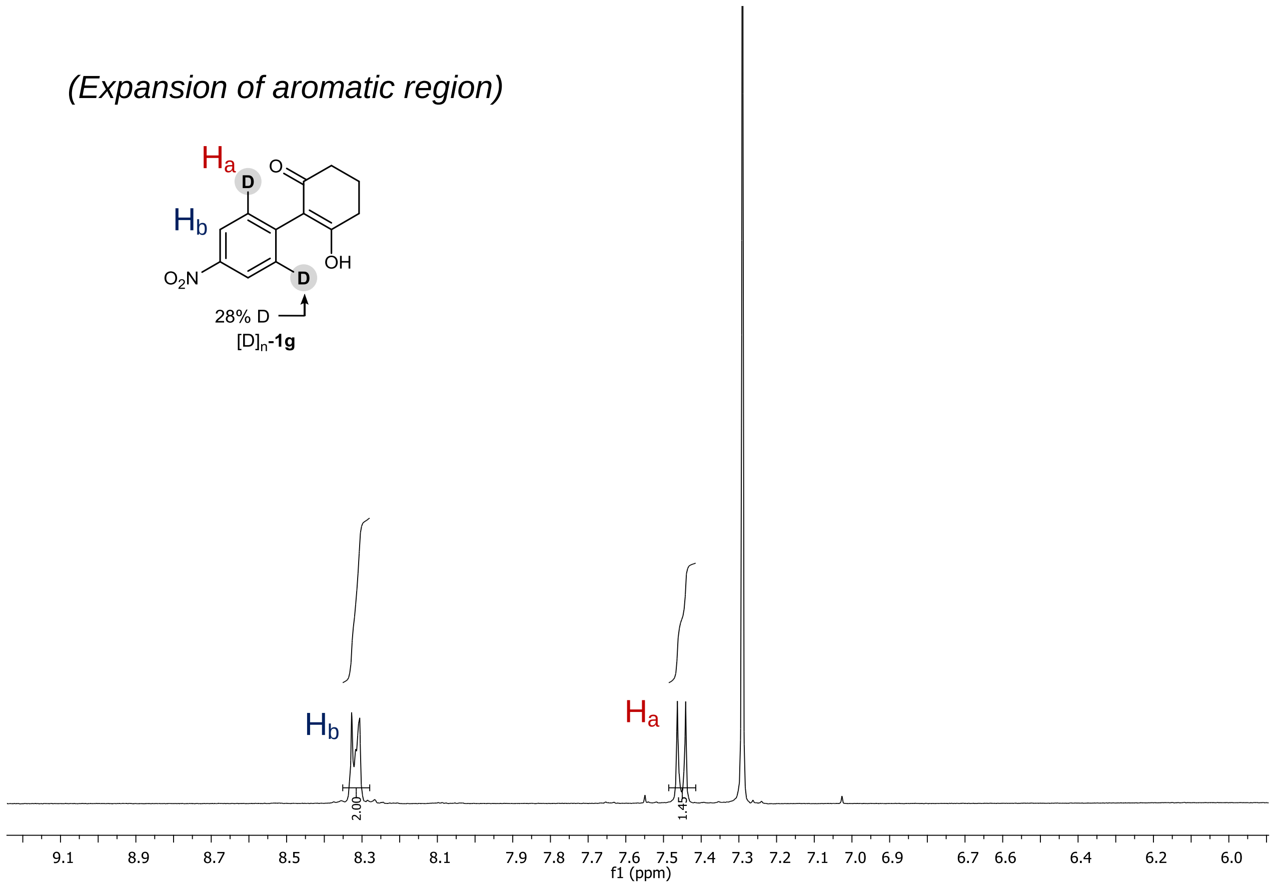

## Reaction with Deuterio-enyne $[D]_6$-2a:

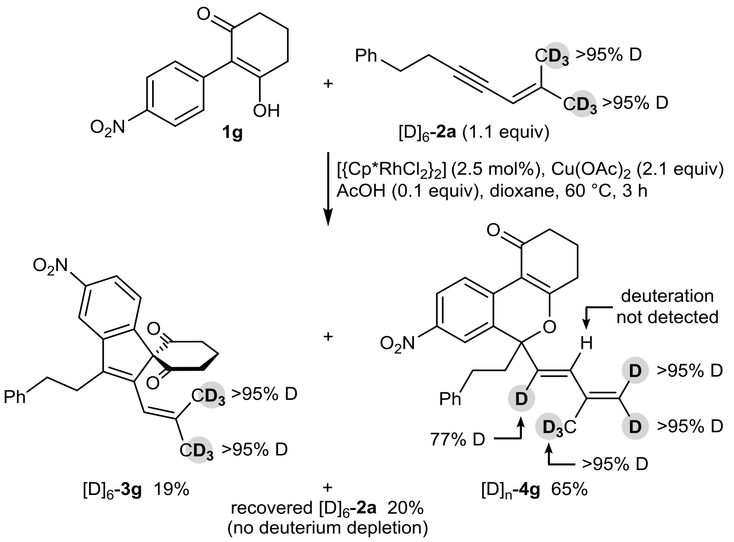


General procedure C was followed using 2-aryl-3-hydroxy-2-cyclohexenone **1g** (117 mg, 0.50 mmol) and deuterio-enyne $[D]_6$-**2a** (105 mg, 0.55 mmol) at 60 °C for a reaction time of 3 h. Purification by flash column chromatography (3:1 petroleum ether:EtOAc) gave *benzopyran* [D]n-**4g** (137 mg, 65%) as a yellow oil followed by *spiroindene* $[D]_n$-**4a** (40 mg, 19%) as a yellow oil.

*(Expansion of aromatic and alkenyl regions)*

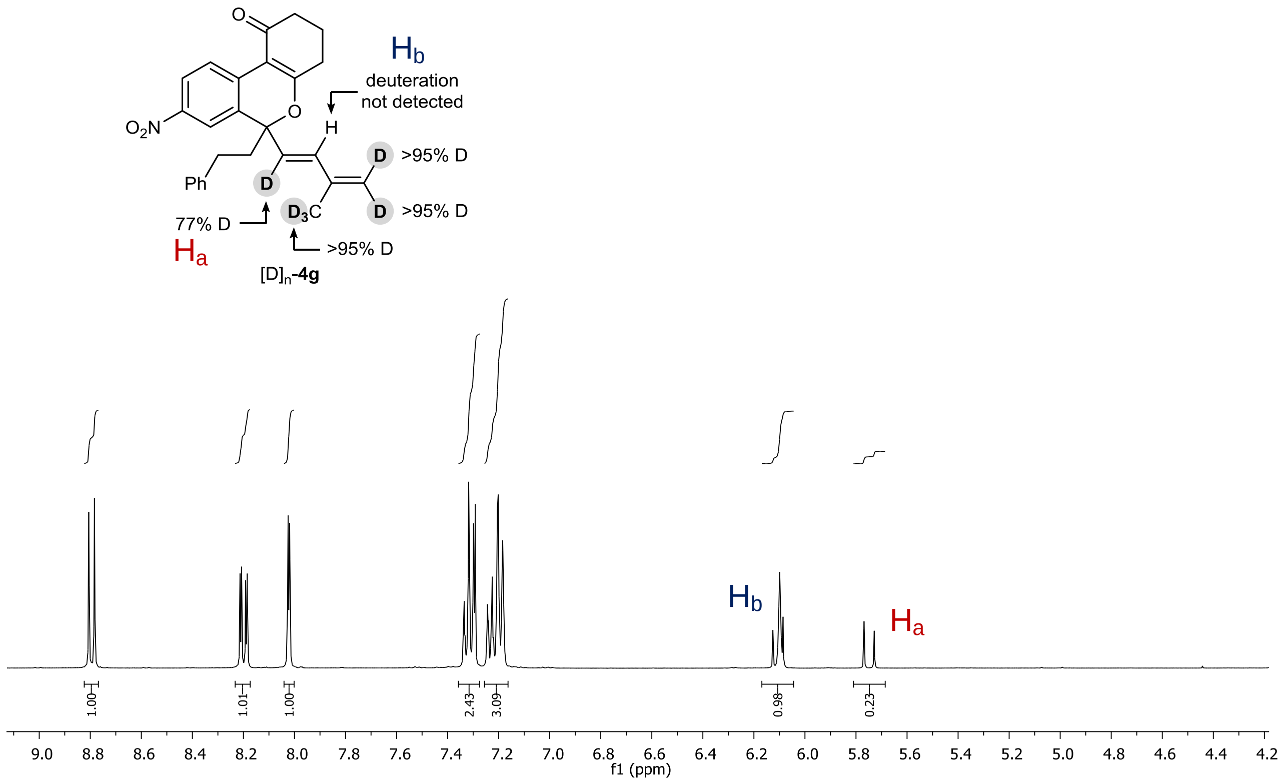

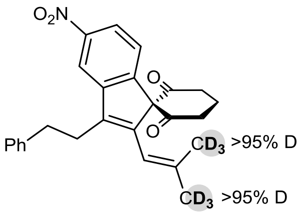


[D]$_6$-**3g**

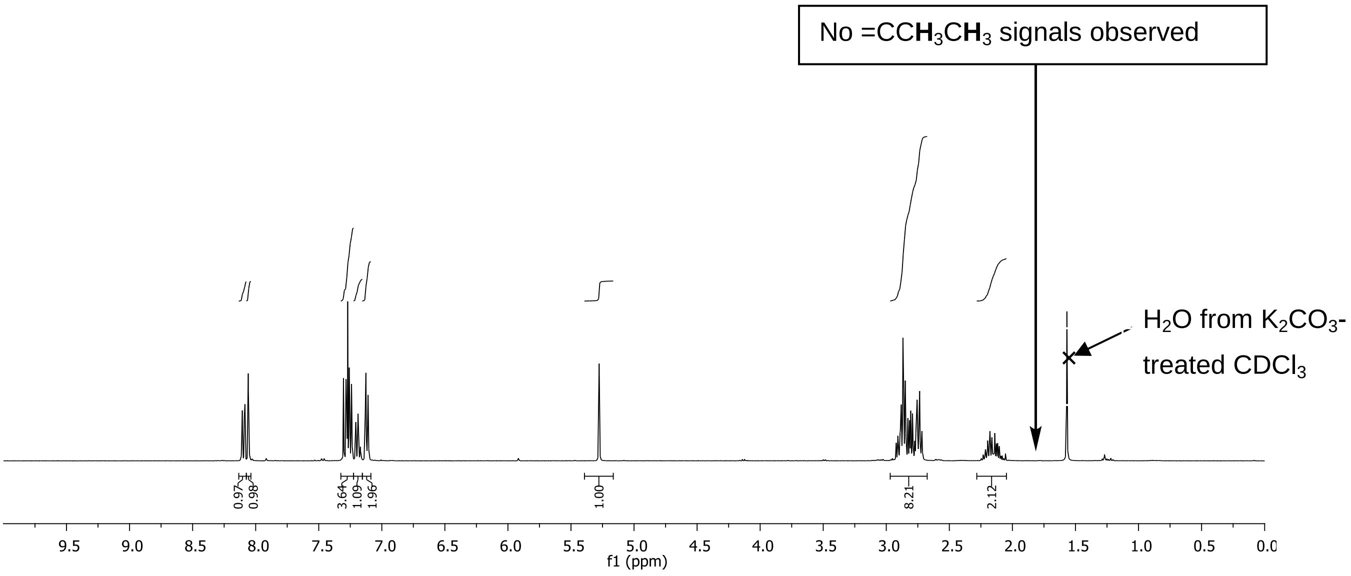


**Data for Spiroindene [D]$_6$-3g**

$R_f$ 0.17 (10:1 petroleum ether:EtOAc); IR 1725, 1696, 1518, 1346, 739, 643 cm$^{-1}$; $^1$H NMR (400 MHz, $CDCl_3$) δ 8.10 (1H, dd, *J* = 8.2, 2.1 Hz, Ar**H**), 8.06 (1H, d, *J* = 2.1 Hz, Ar**H**), 7.33–7.23 (4H, m, 4 × Ar**H**), 7.23–7.15 (1H, m, Ar**H**), 7.15–7.09 (2H, m, 2 × Ar**H**), 5.28 (1H, s, =C**H**), 2.95–2.69 (8H, m, PhC**H**$_2$C**H**$_2$ and C**H**$_2$CH$_2$C**H**$_2$), 2.26–2.05 (2H, m, CH$_2$C**H**$_2$CH$_2$); $^{13}$C NMR (100.6 MHz, $CDCl_3$) δ 203.5 (C), 149.6 (C), 148.4 (C), 147.7 (C), 143.1 (C), 141.0 (C), 140.9 (C), 128.7 (2 × CH), 128.3 (2 × CH), 126.1 (CH), 123.5 (CH), 121.1 (CH), 117.1 (C), 115.0 (CH), 85.3 (C), 40.0 ($CH_2$), 34.0 ($CH_2$), 28.1 ($CH_2$), 17.4 ($CH_2$); HRMS (ESI +ve) Exact mass calculated for $C_{26}D_6H_{19}NO_4Na$ [M+Na]$^+$: 444.2160, found: 444.2058.

**Oxidative Annulation Reaction with $D_2O$ present:**

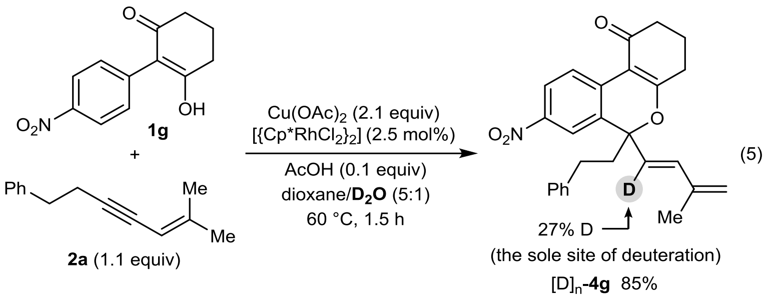


To a microwave vial, was added 2-aryl-3-hydroxy-2-cyclohexenone **1g** (23.4 mg, 0.10 mmol), [Cp*$RhCl_2$]$_2$ (1.5 mg, 2.6 µmol), $Cu(OAc)_2$ (38.2 mg, 0.21 mmol), AcOH (1 µL, 10 µmol), and enyne **2a** (20.2 mg, 0.11 mmol). The vessel was then sealed, flushed with $N_2$, and to the vial was added 1,4-dioxane (1 mL) and $D_2O$ (200 µL). The reaction was then heated at 60 °C for 1.5 h. The reaction was cooled to room temperature, filtered through a short pad of silica using $Et_2O$ (25 mL) as eluent, and concentrated *in vacuo*. Purification of the residue by flash column chromatography (3:1 petroleum ether:EtOAc)gave *benzopyran* $[D]_n$-**4g** (35.6 mg, 85%) as a yellow oil.

*(Expansion of aromatic and alkenyl regions)*

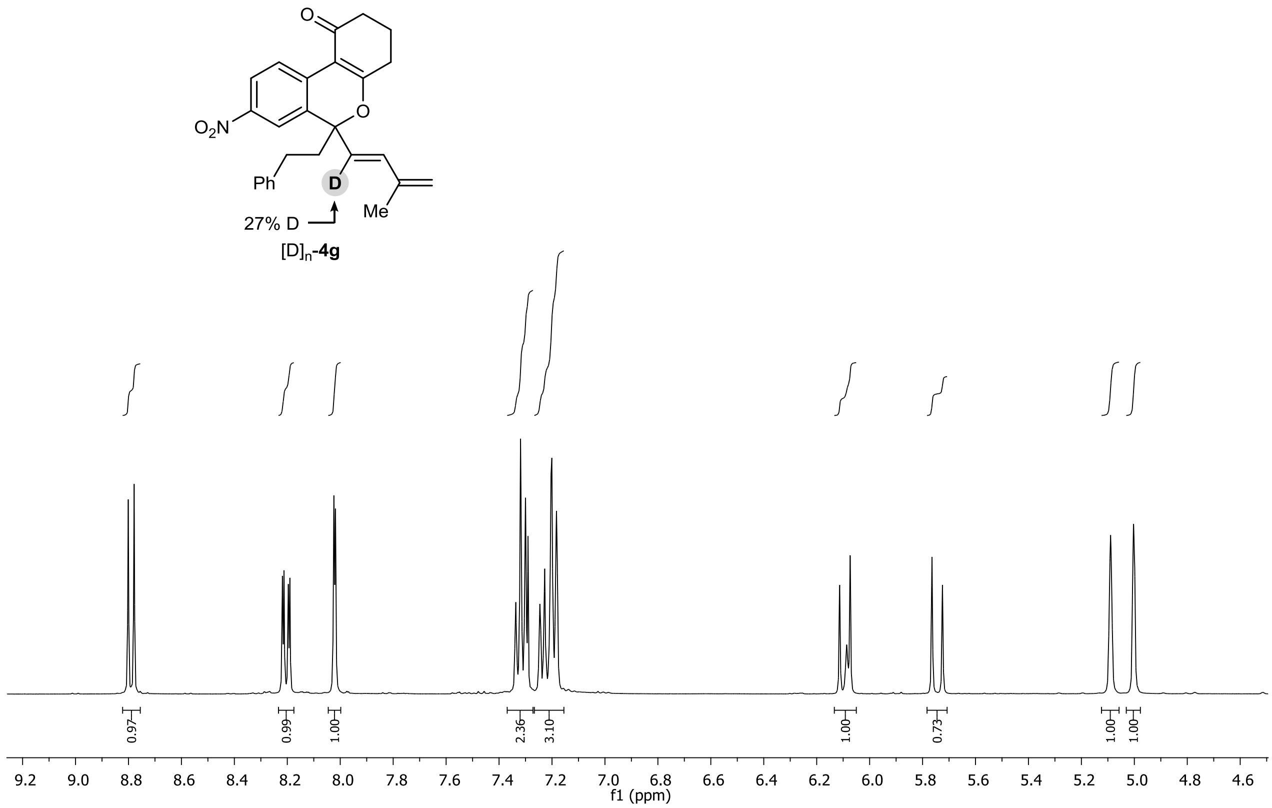

# 5. NMR Spectra

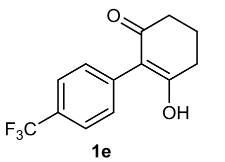


**1e**

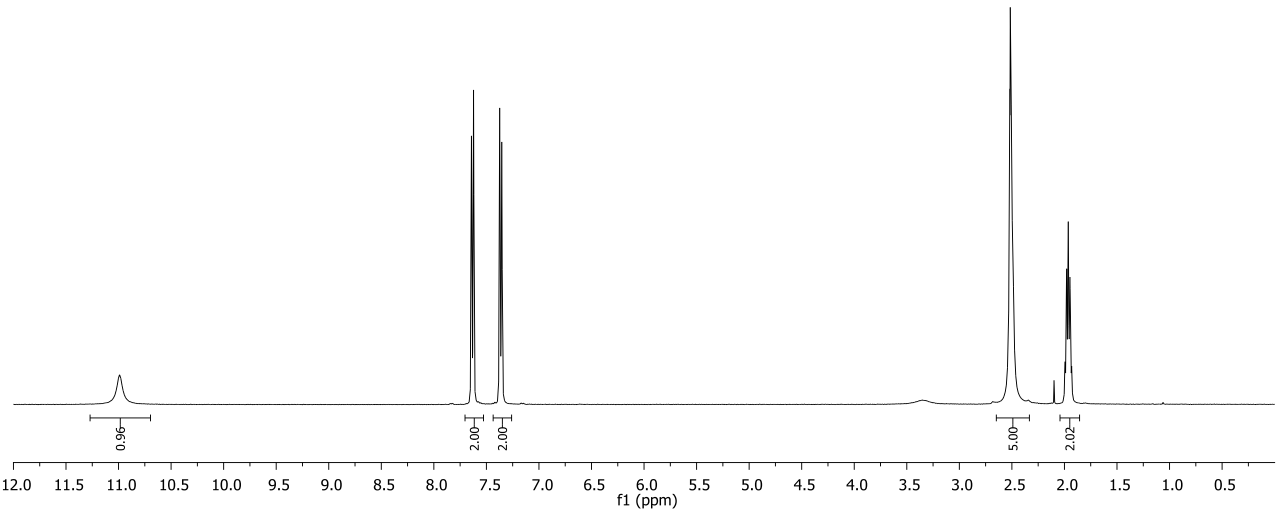


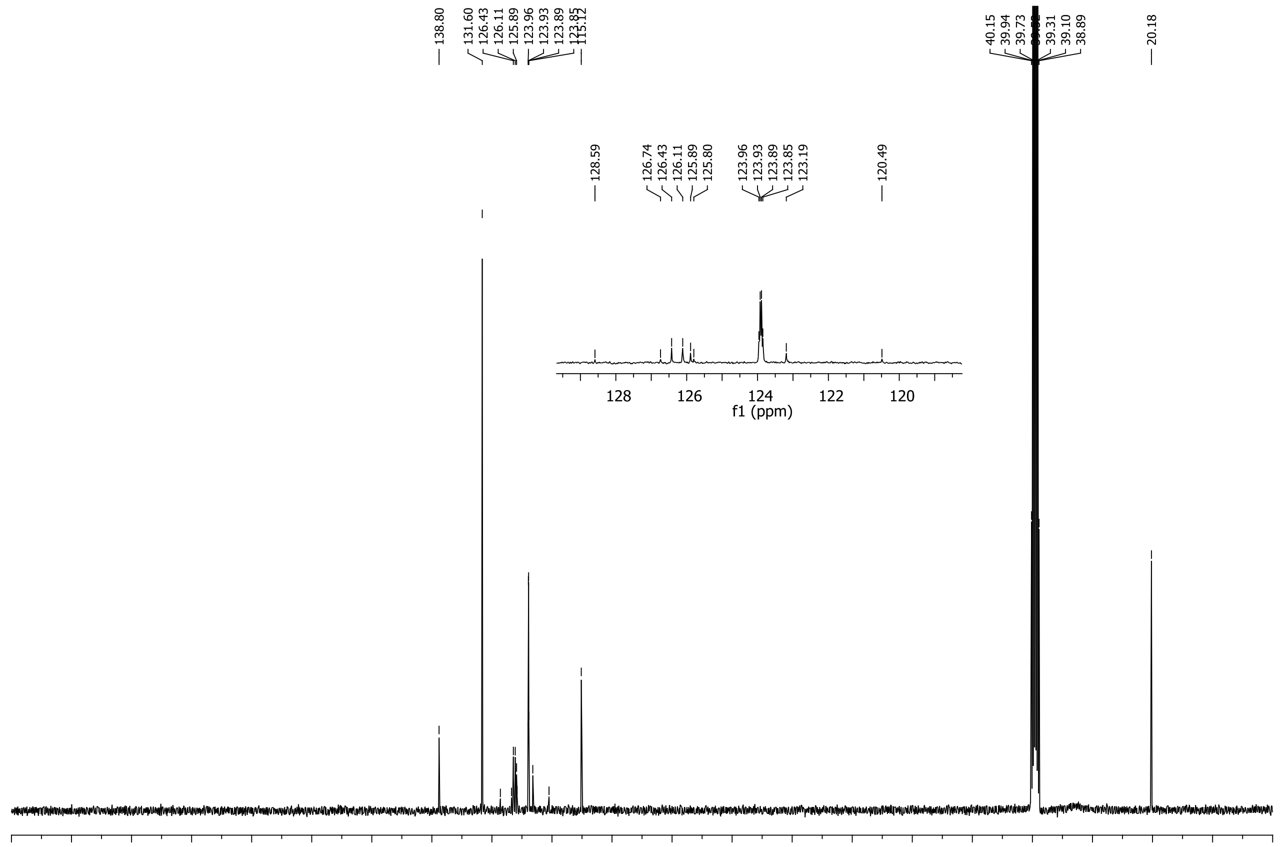

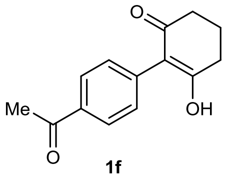
O
Me
OH
O

**1f**

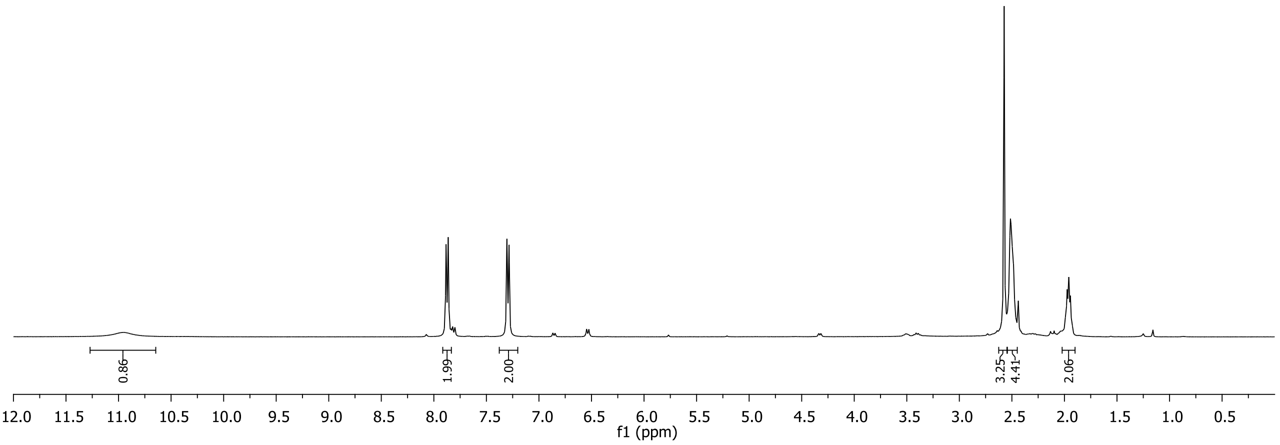
0.86
1.99
2.00
3.25
4.41
2.06
12.0 11.5 11.0 10.5 10.0 9.5 9.0 8.5 8.0 7.5 7.0 6.5 6.0 5.5 5.0 4.5 4.0 3.5 3.0 2.5 2.0 1.5 1.0 0.5
f1 (ppm)

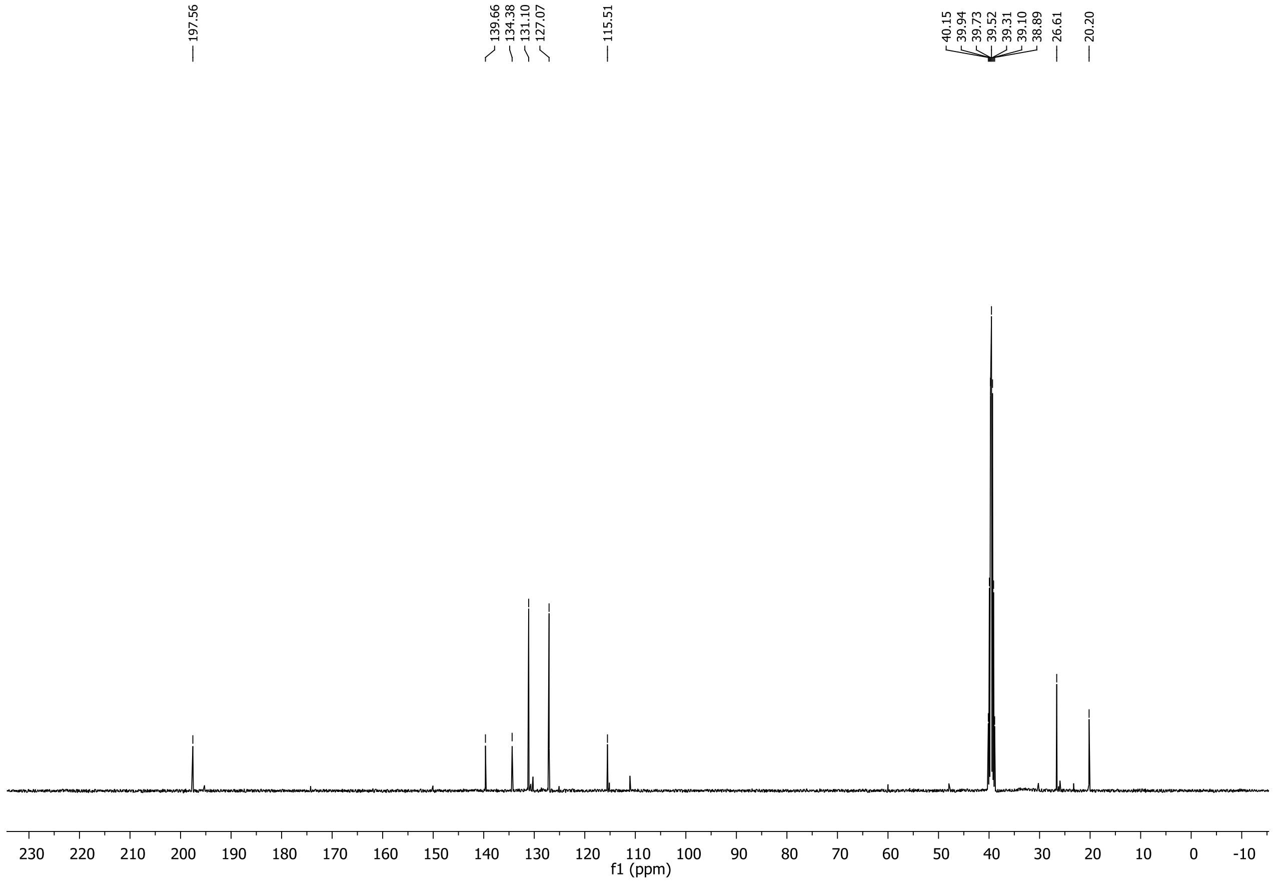
197.56
139.66
134.38
131.10
127.07
115.51
40.15
39.94
39.73
39.52
39.31
39.10
38.89
26.61
20.20
230 220 210 200 190 180 170 160 150 140 130 120 110 100 90 80 70 60 50 40 30 20 10 0 -10
f1 (ppm)

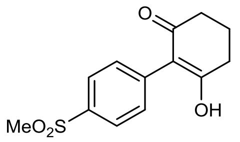
O
OH
$MeO_2S$

**1h**

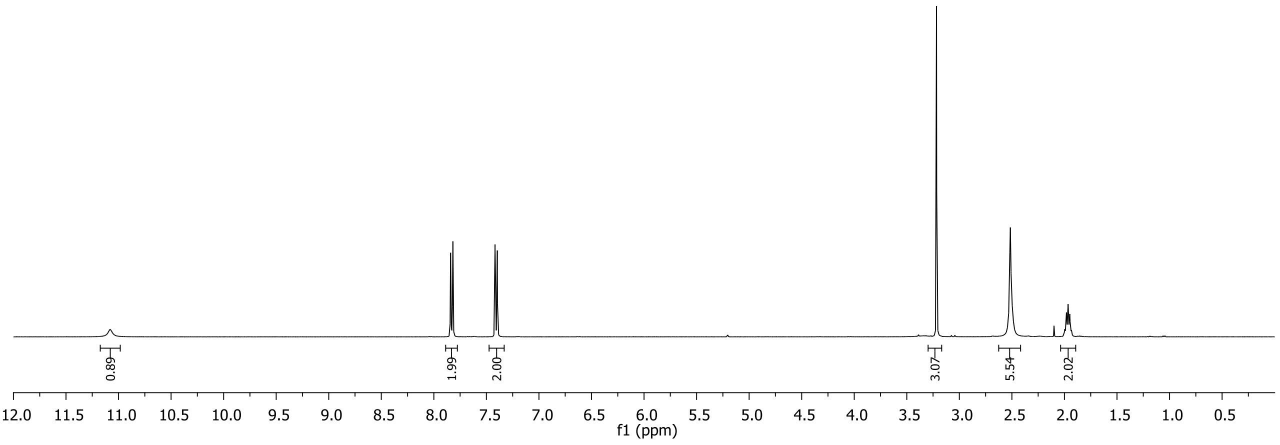
0.89
1.99
2.00
3.07
5.54
2.02
12.0 11.5 11.0 10.5 10.0 9.5 9.0 8.5 8.0 7.5 7.0 6.5 6.0 5.5 5.0 4.5 4.0 3.5 3.0 2.5 2.0 1.5 1.0 0.5
f1 (ppm)

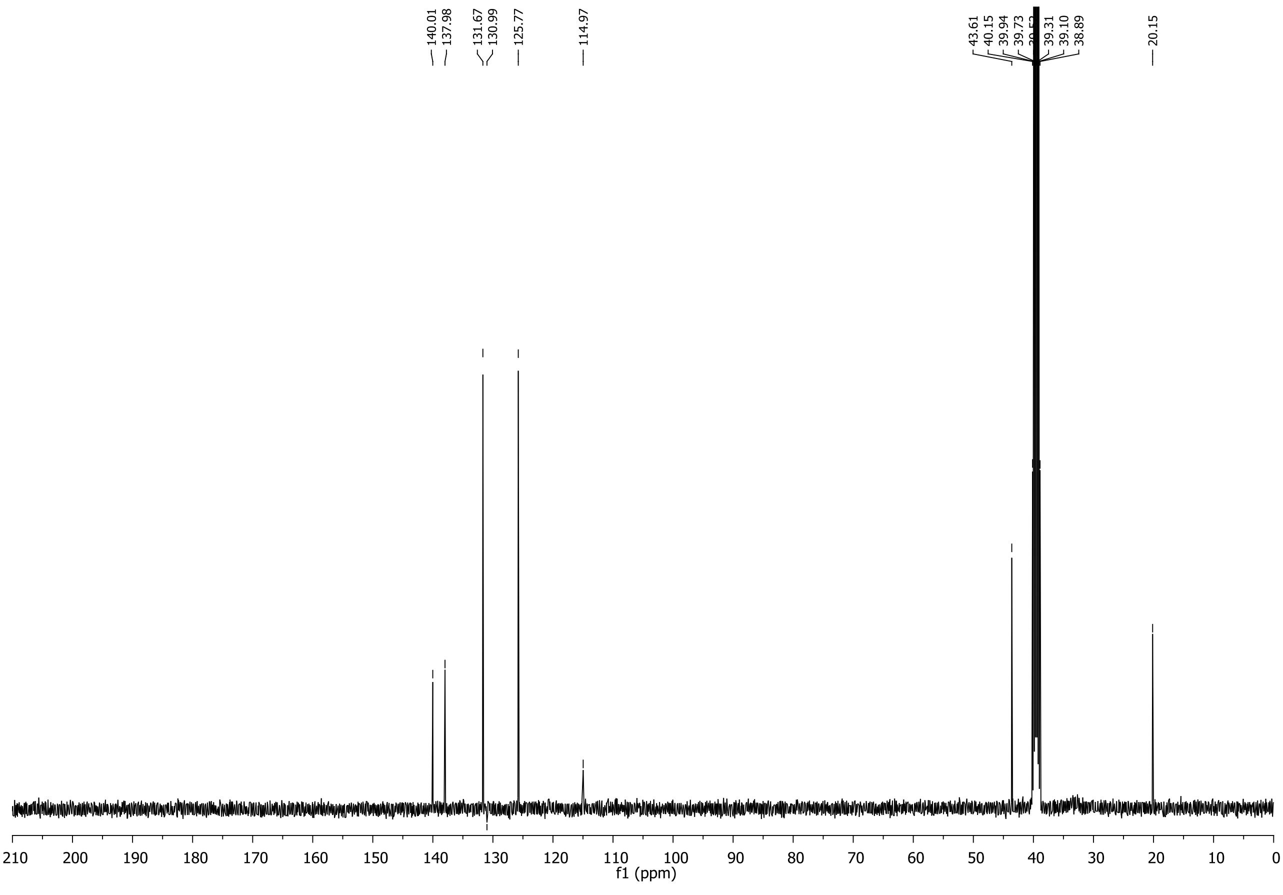
140.01
137.98
131.67
130.99
125.77
114.97
43.61
40.15
39.94
39.73
39.31
39.10
38.89
20.15
210 200 190 180 170 160 150 140 130 120 110 100 90 80 70 60 50 40 30 20 10 0
f1 (ppm)

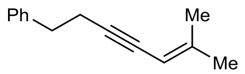
Ph
Me
Me

**2a**

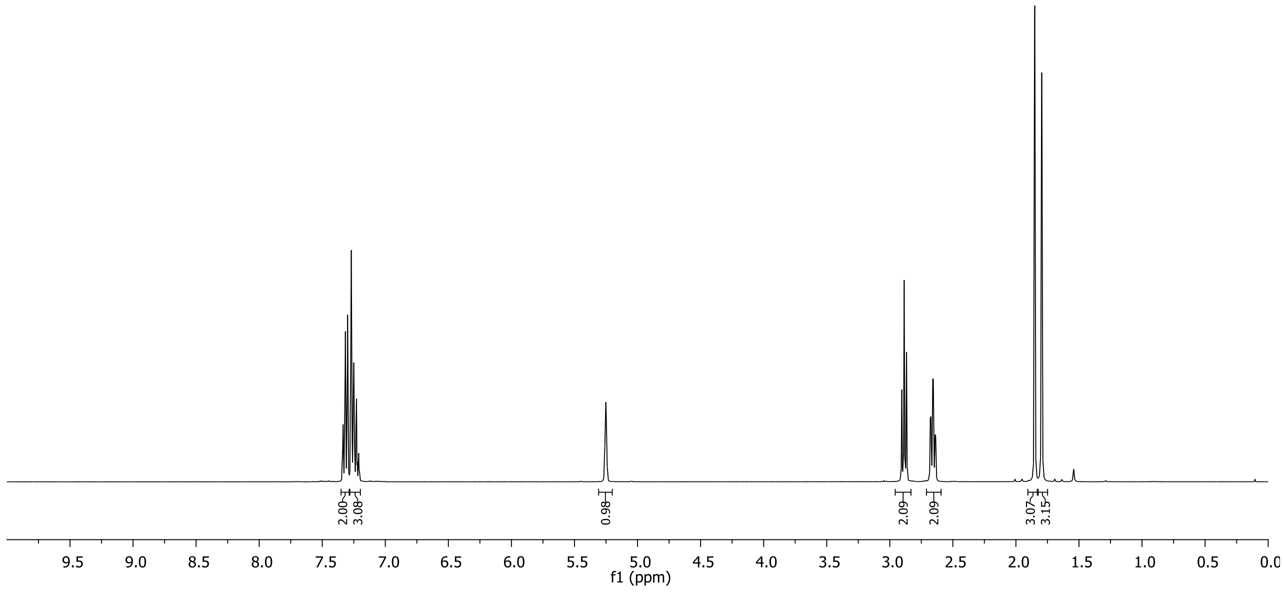
2.00
3.08
0.98
2.09
2.09
3.07
3.15
9.5 9.0 8.5 8.0 7.5 7.0 6.5 6.0 5.5 5.0 4.5 4.0 3.5 3.0 2.5 2.0 1.5 1.0 0.5 0.0
f1 (ppm)

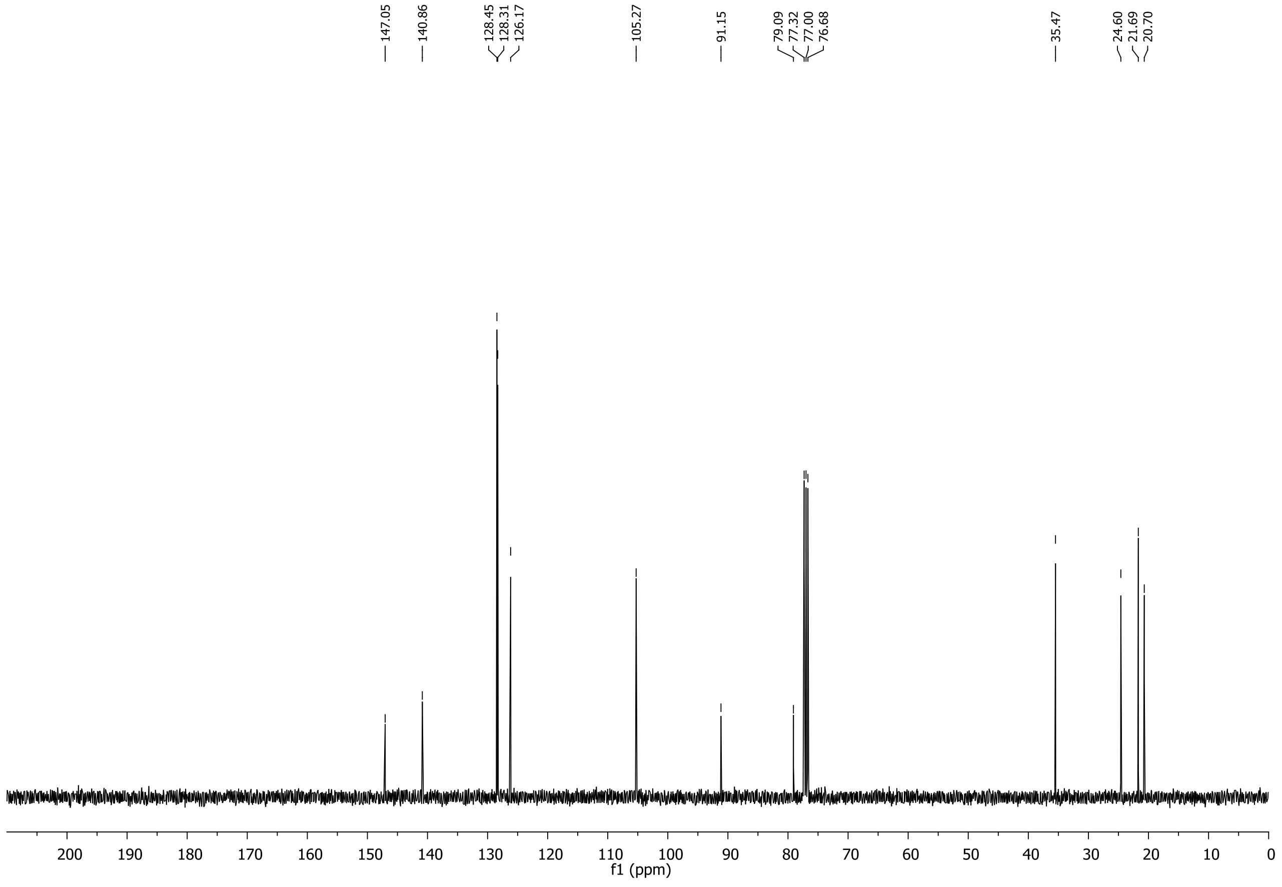
147.05
140.86
128.45
128.31
126.17
105.27
91.15
79.09
77.32
77.00
76.68
35.47
24.60
21.69
20.70
200 190 180 170 160 150 140 130 120 110 100 90 80 70 60 50 40 30 20 10 0
f1 (ppm)

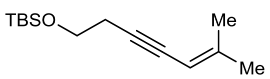
TBSO
Me
Me

**2b**

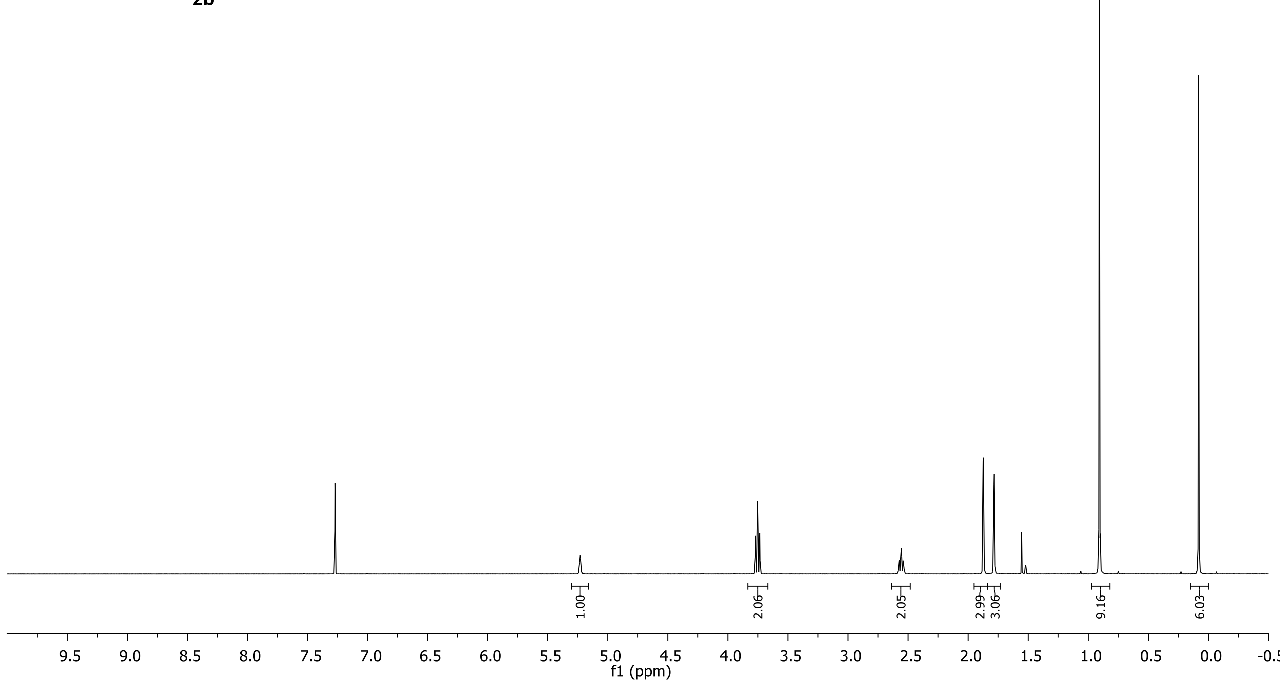
1.00
2.06
2.05
2.99
3.06
9.16
6.03
f1 (ppm)

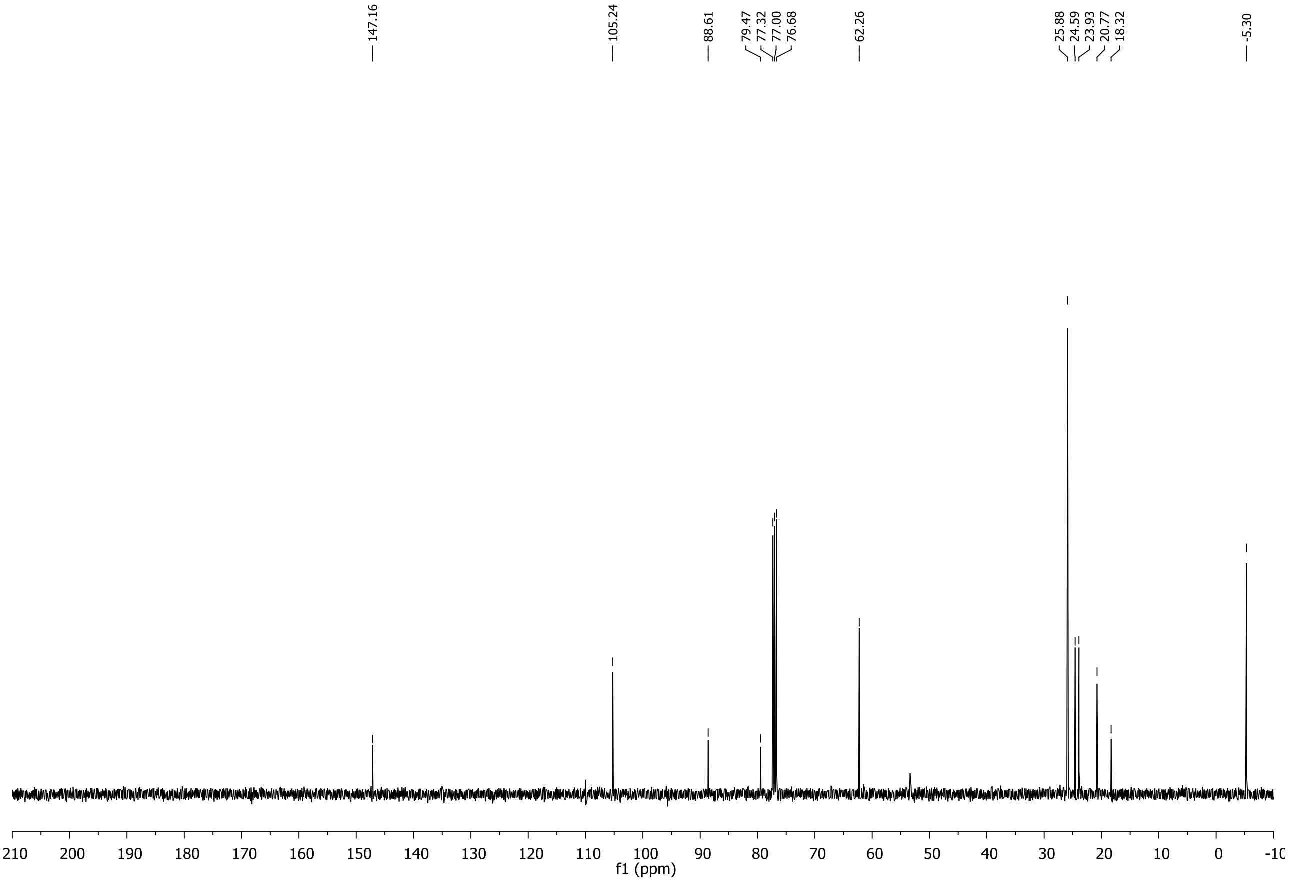
147.16
105.24
88.61
79.47
77.32
77.00
76.68
62.26
25.88
24.59
23.93
20.77
18.32
-5.30
f1 (ppm)

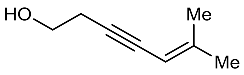
HO
Me
Me

**2c**

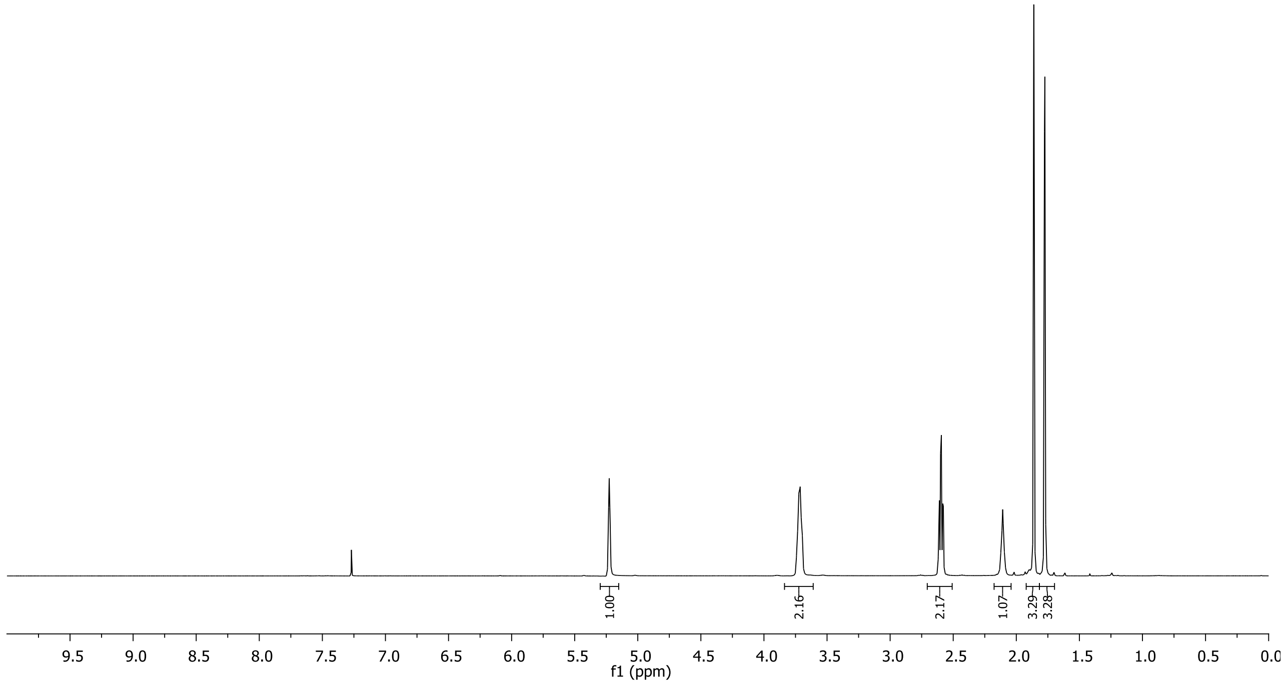
1.00
2.16
2.17
1.07
3.29
3.28
9.5 9.0 8.5 8.0 7.5 7.0 6.5 6.0 5.5 5.0 4.5 4.0 3.5 3.0 2.5 2.0 1.5 1.0 0.5
f1 (ppm)

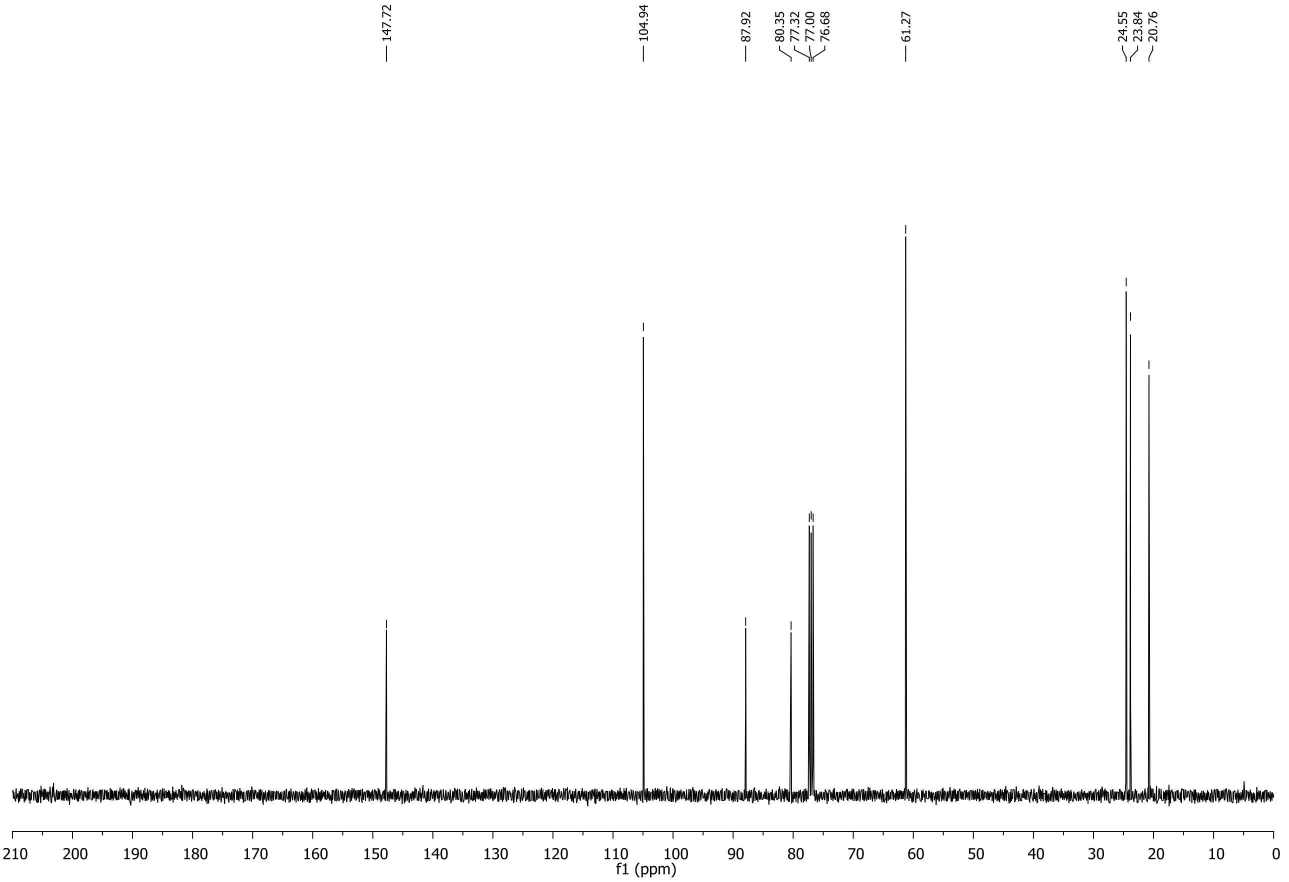
147.72
104.94
87.92
80.35
77.32
77.00
76.68
61.27
24.55
23.84
20.76
210 200 190 180 170 160 150 140 130 120 110 100 90 80 70 60 50 40 30 20 10 0
f1 (ppm)

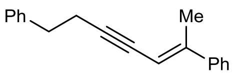
Ph
Me
Ph

**2d**

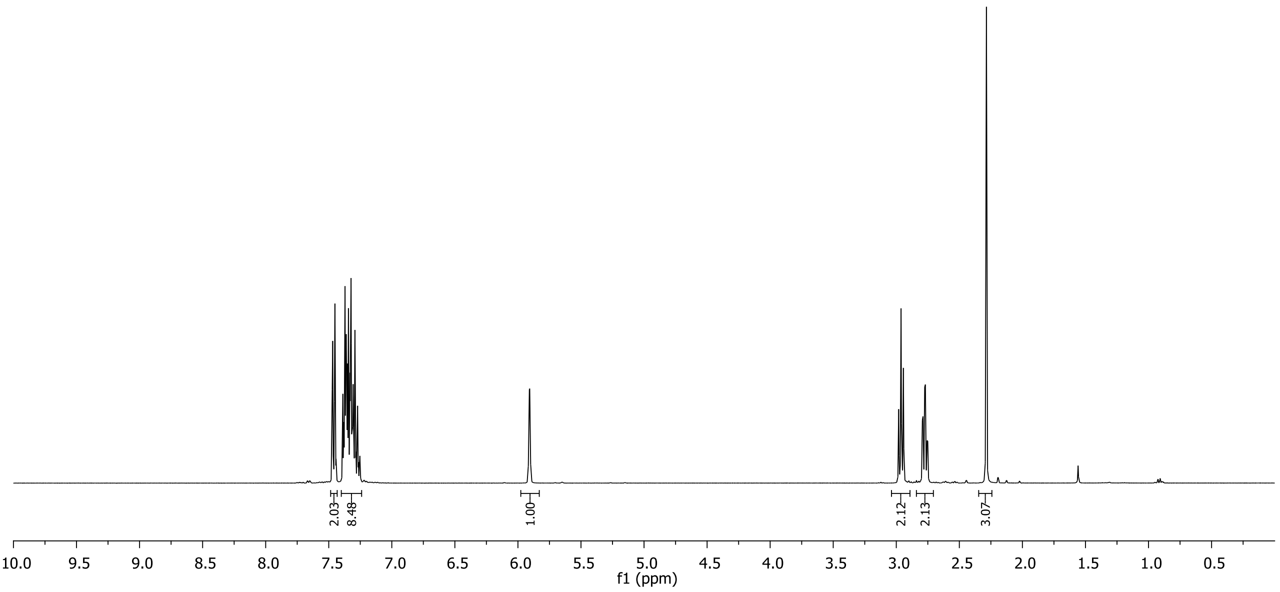
2.03
8.48
1.00
2.12
2.13
3.07
10.0 9.5 9.0 8.5 8.0 7.5 7.0 6.5 6.0 5.5 5.0 4.5 4.0 3.5 3.0 2.5 2.0 1.5 1.0 0.5
f1 (ppm)

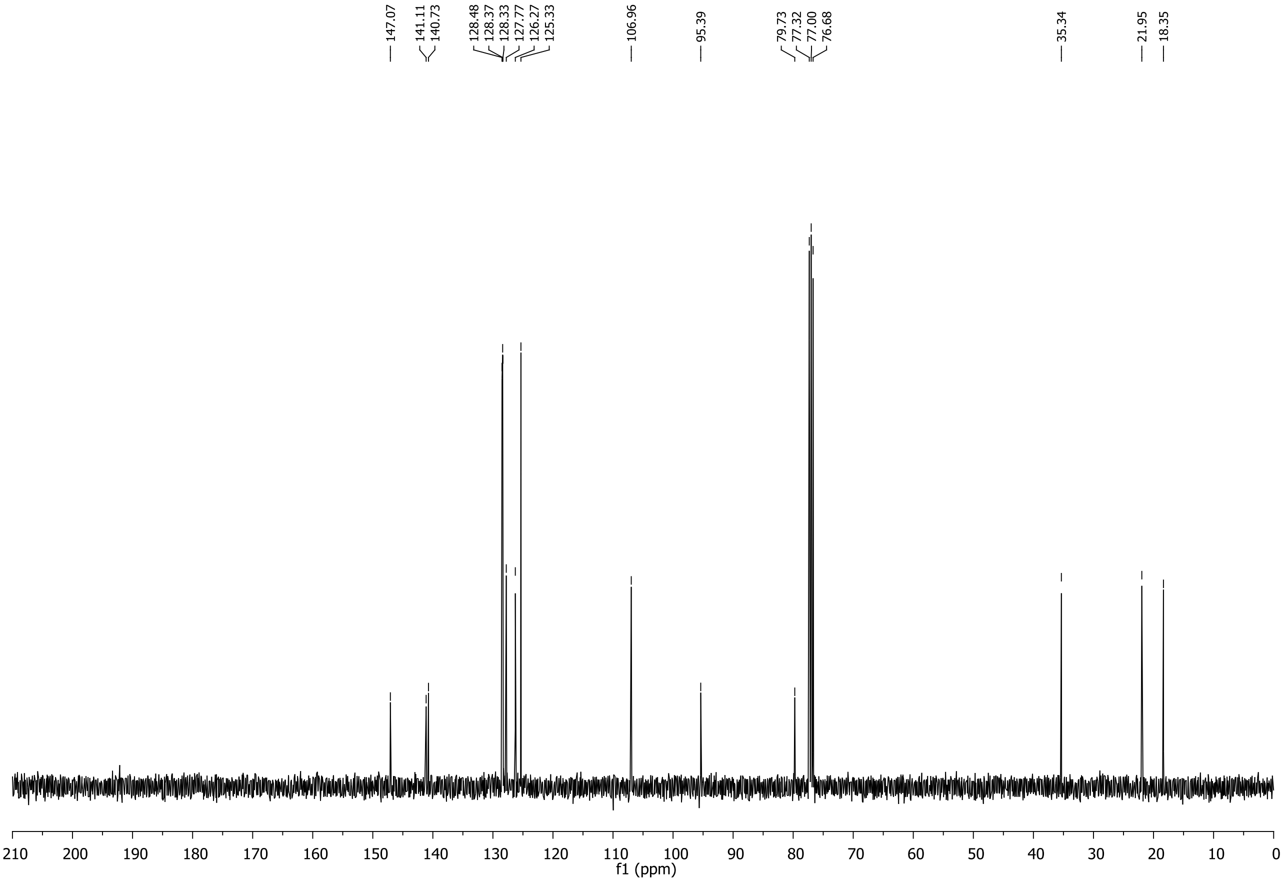
147.07
141.11
140.73
128.48
128.37
128.33
127.77
126.27
125.33
106.96
95.39
79.73
77.32
77.00
76.68
35.34
21.95
18.35
210 200 190 180 170 160 150 140 130 120 110 100 90 80 70 60 50 40 30 20 10 0
f1 (ppm)

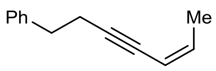
Ph
Me

**2e**

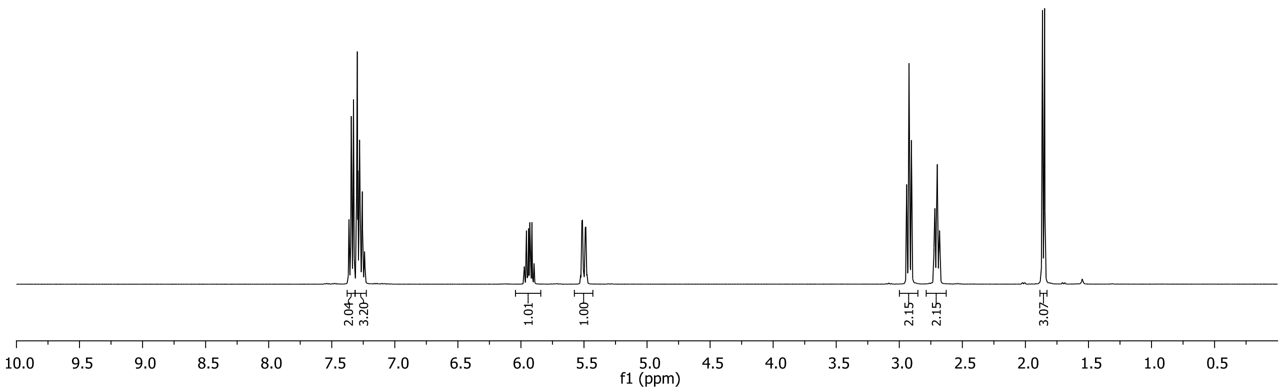
2.04
3.20
1.01
1.00
2.15
2.15
3.07
10.0 9.5 9.0 8.5 8.0 7.5 7.0 6.5 6.0 5.5 5.0 4.5 4.0 3.5 3.0 2.5 2.0 1.5 1.0 0.5
f1 (ppm)

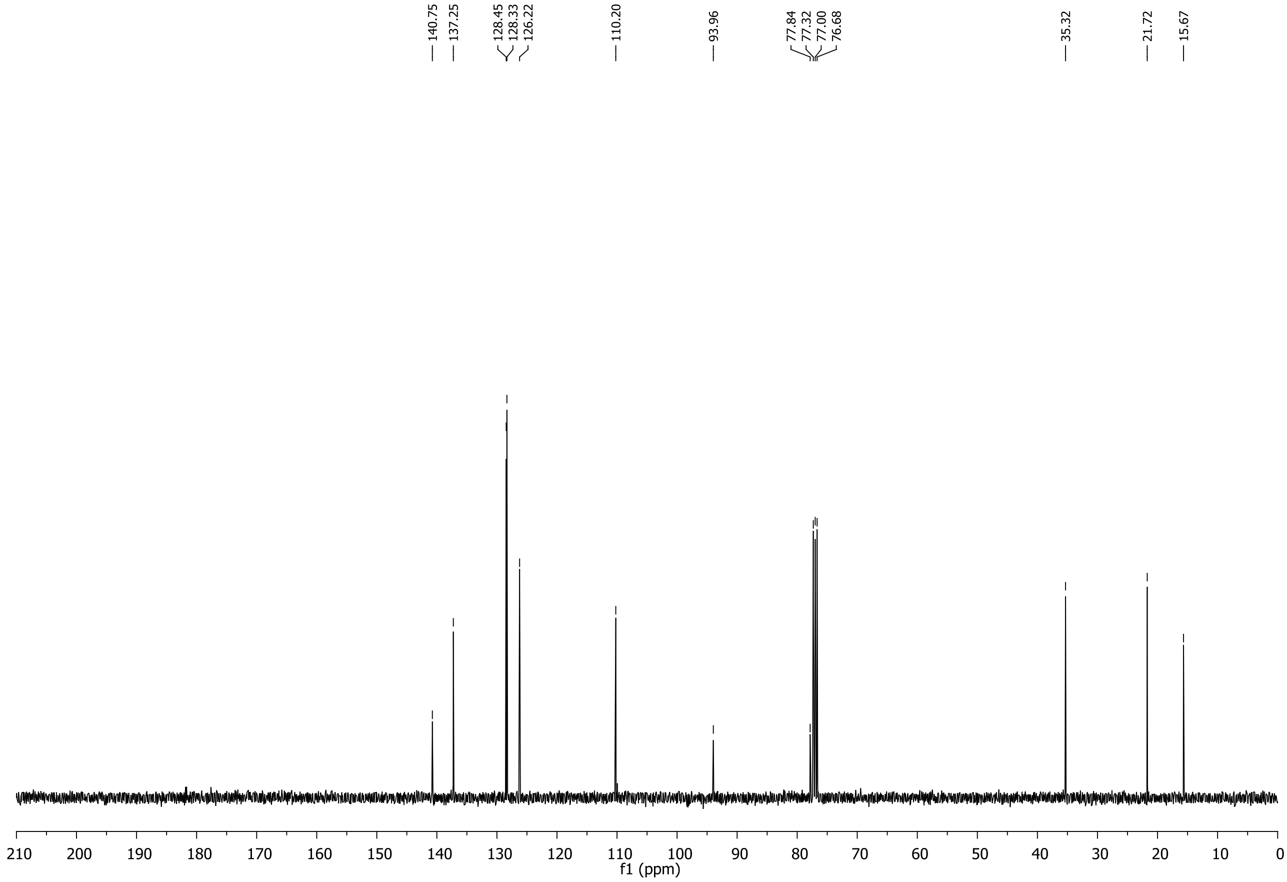
140.75
137.25
128.45
128.33
126.22
110.20
93.96
77.84
77.32
77.00
76.68
35.32
21.72
15.67
210 200 190 180 170 160 150 140 130 120 110 100 90 80 70 60 50 40 30 20 10 0
f1 (ppm)

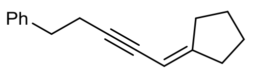
Ph

**2f**

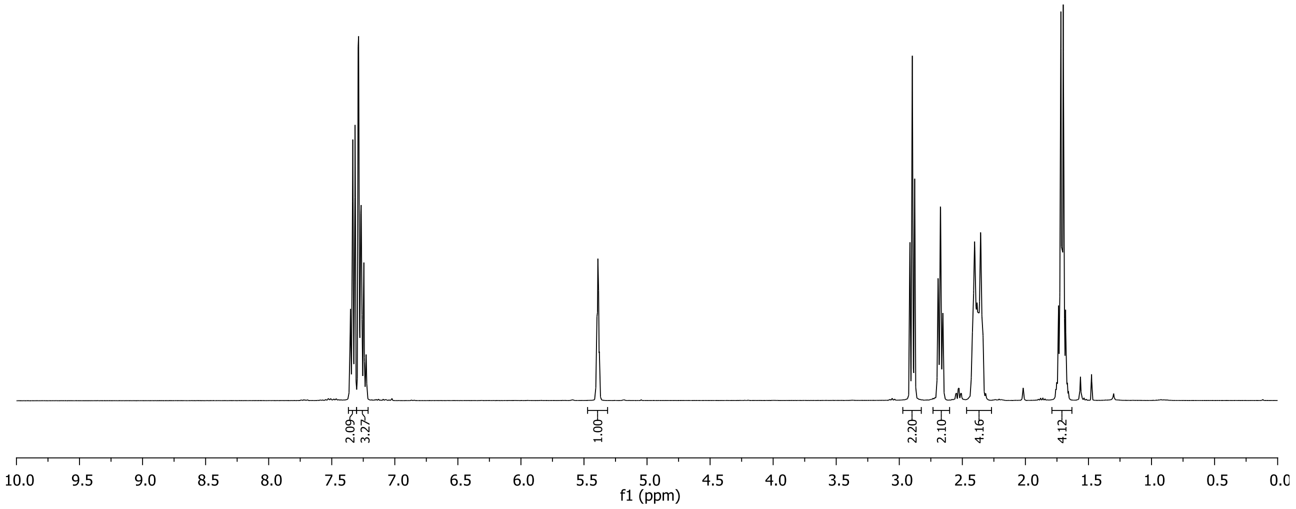
2.09
3.27
1.00
2.20
2.10
4.16
4.12
f1 (ppm)

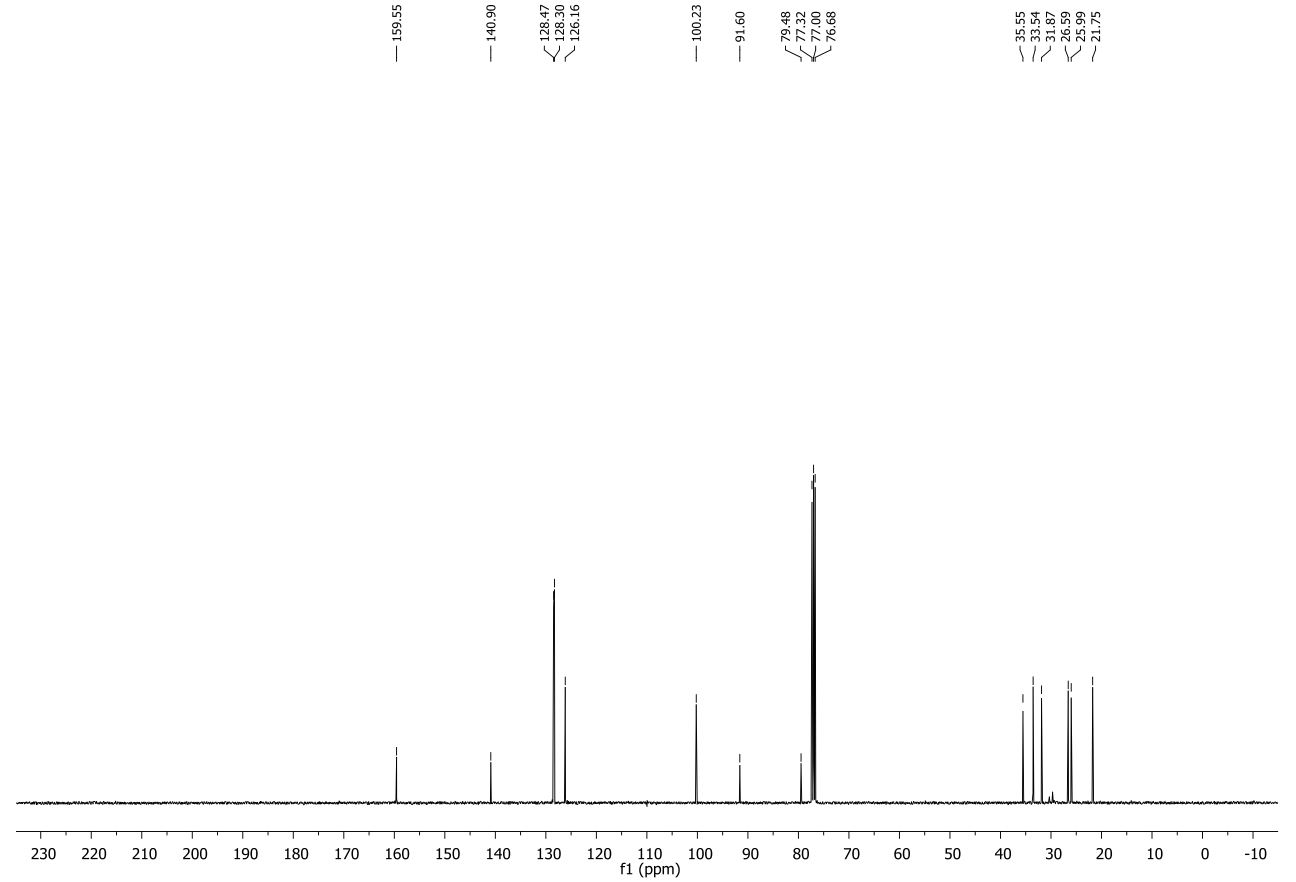
159.55
140.90
128.47
128.30
126.16
100.23
91.60
79.48
77.32
77.00
76.68
35.55
33.54
31.87
26.59
25.99
21.75
f1 (ppm)

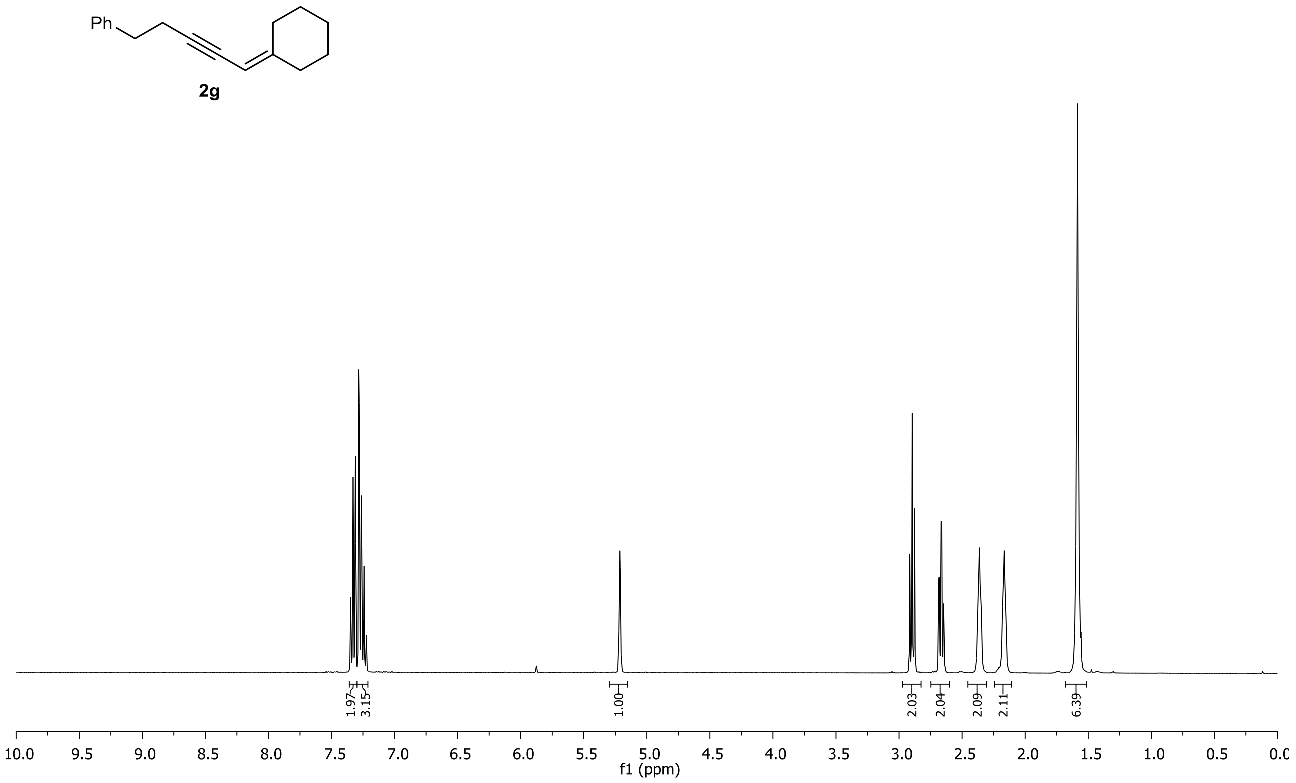
Ph
2g
1.97
3.15
1.00
2.03
2.04
2.09
2.11
6.39
10.0 9.5 9.0 8.5 8.0 7.5 7.0 6.5 6.0 5.5 5.0 4.5 4.0 3.5 3.0 2.5 2.0 1.5 1.0 0.5 0.0
f1 (ppm)

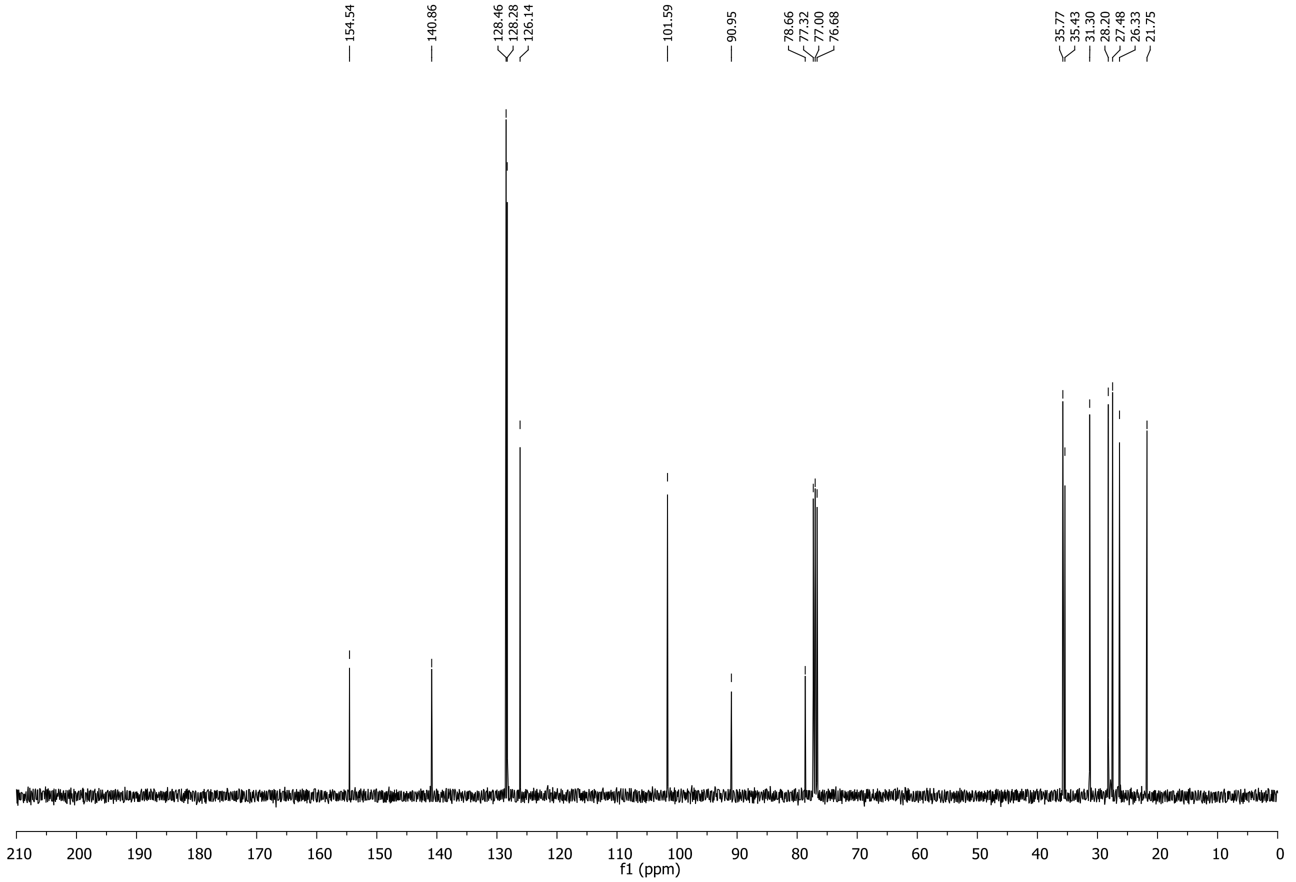
154.54
140.86
128.46
128.28
126.14
101.59
90.95
78.66
77.32
77.00
76.68
35.77
35.43
31.30
28.20
27.48
26.33
21.75
210 200 190 180 170 160 150 140 130 120 110 100 90 80 70 60 50 40 30 20 10 0
f1 (ppm)

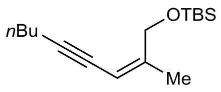
OTBS
nBu
Me

**2h**

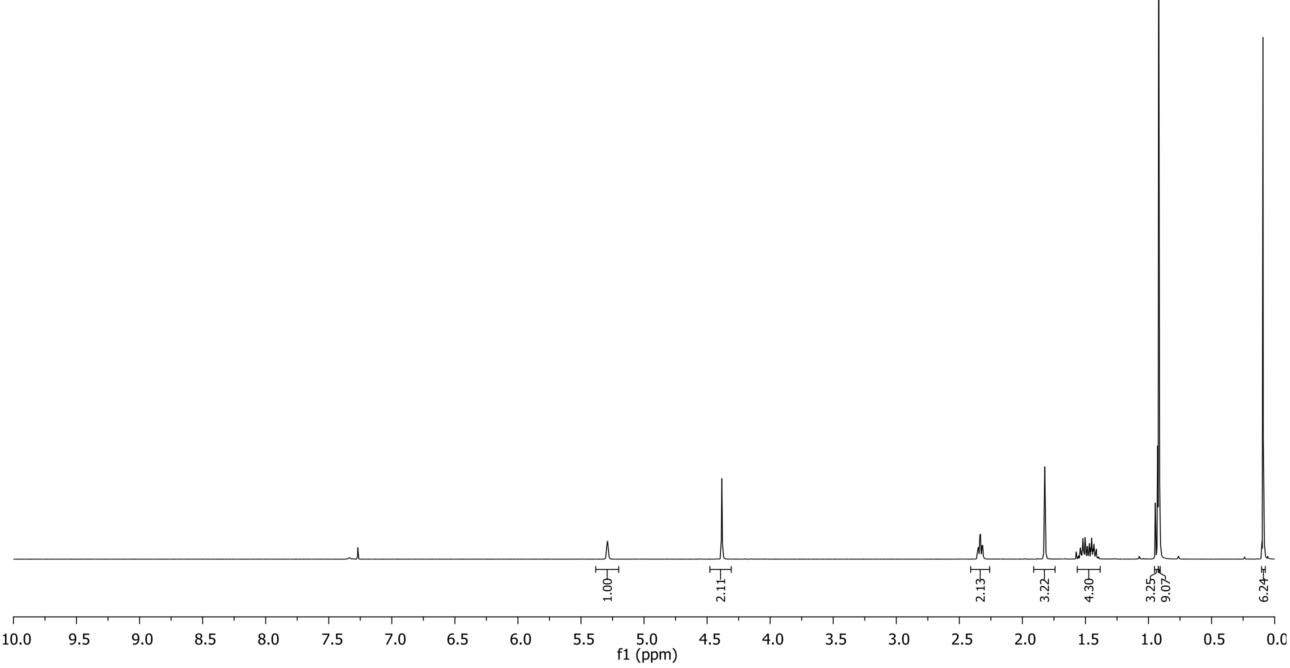
1.00
2.11
2.13
3.22
4.30
3.25
9.07
6.24
f1 (ppm)

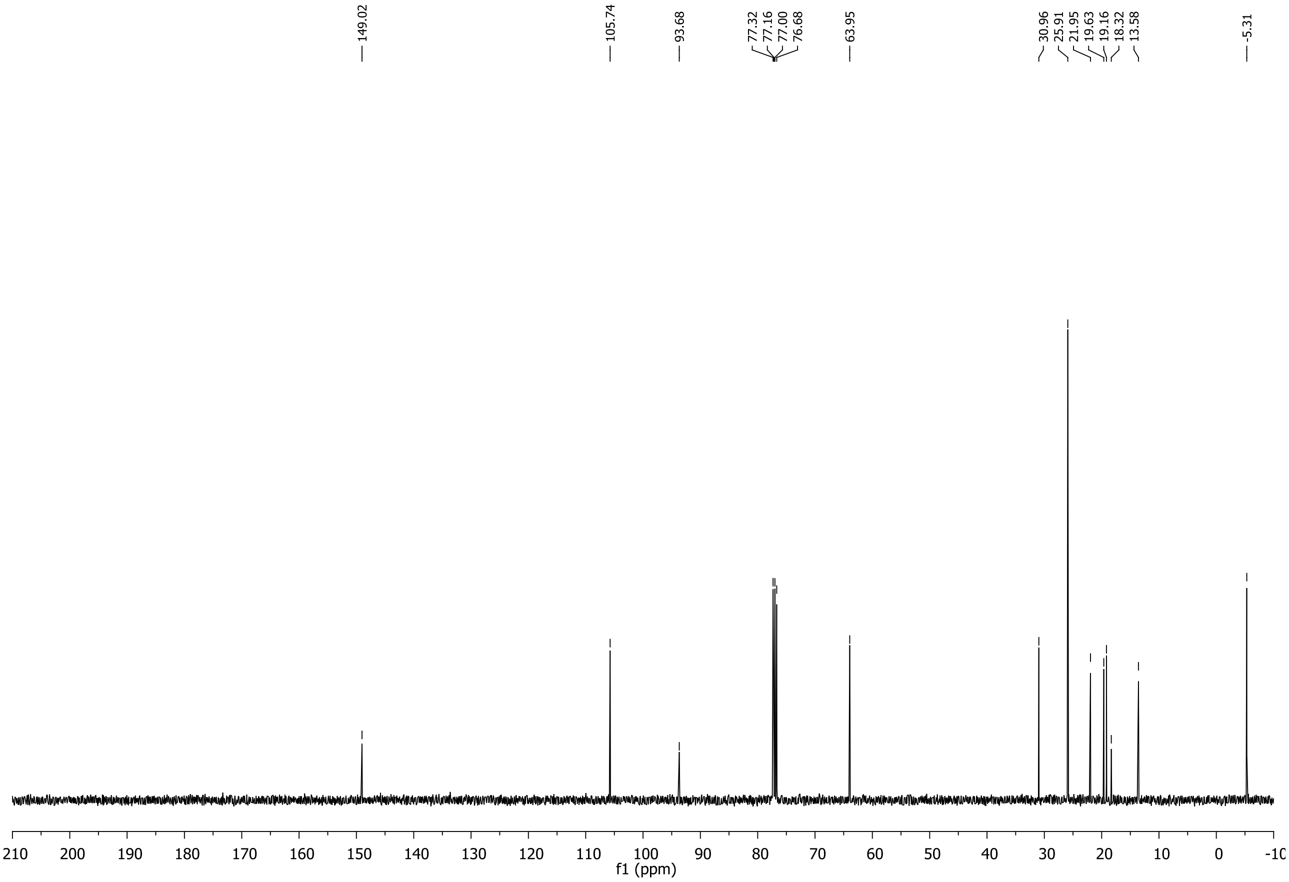
149.02
105.74
93.68
77.32
77.16
77.00
76.68
63.95
30.96
25.91
21.95
19.63
19.16
18.32
13.58
-5.31
f1 (ppm)

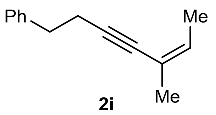
Ph
Me
Me

**2i**

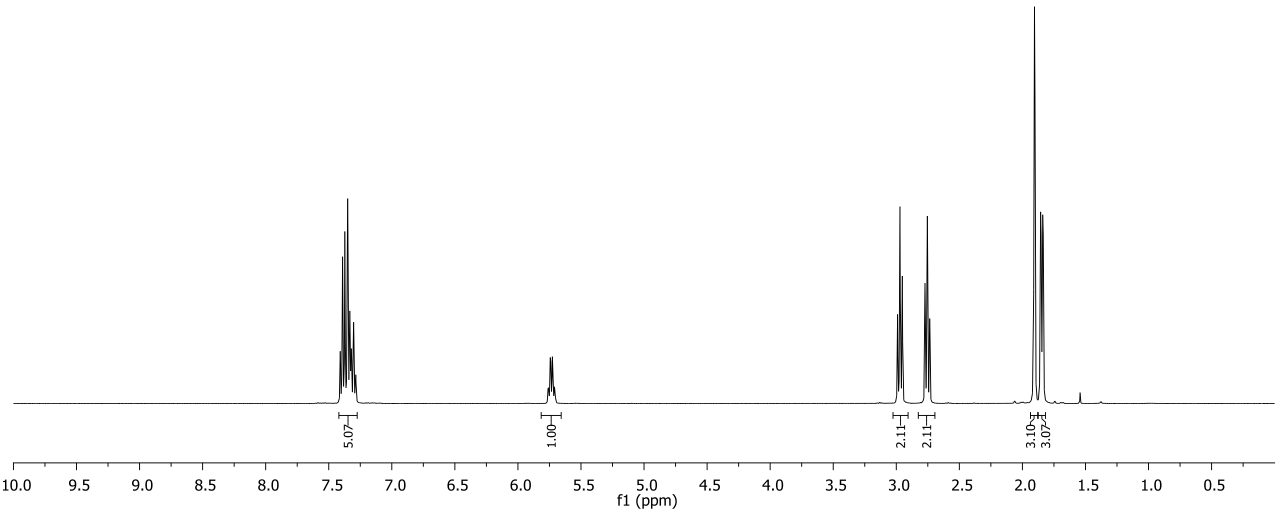
5.07
1.00
2.11
2.11
3.10
3.07
10.0 9.5 9.0 8.5 8.0 7.5 7.0 6.5 6.0 5.5 5.0 4.5 4.0 3.5 3.0 2.5 2.0 1.5 1.0 0.5
f1 (ppm)

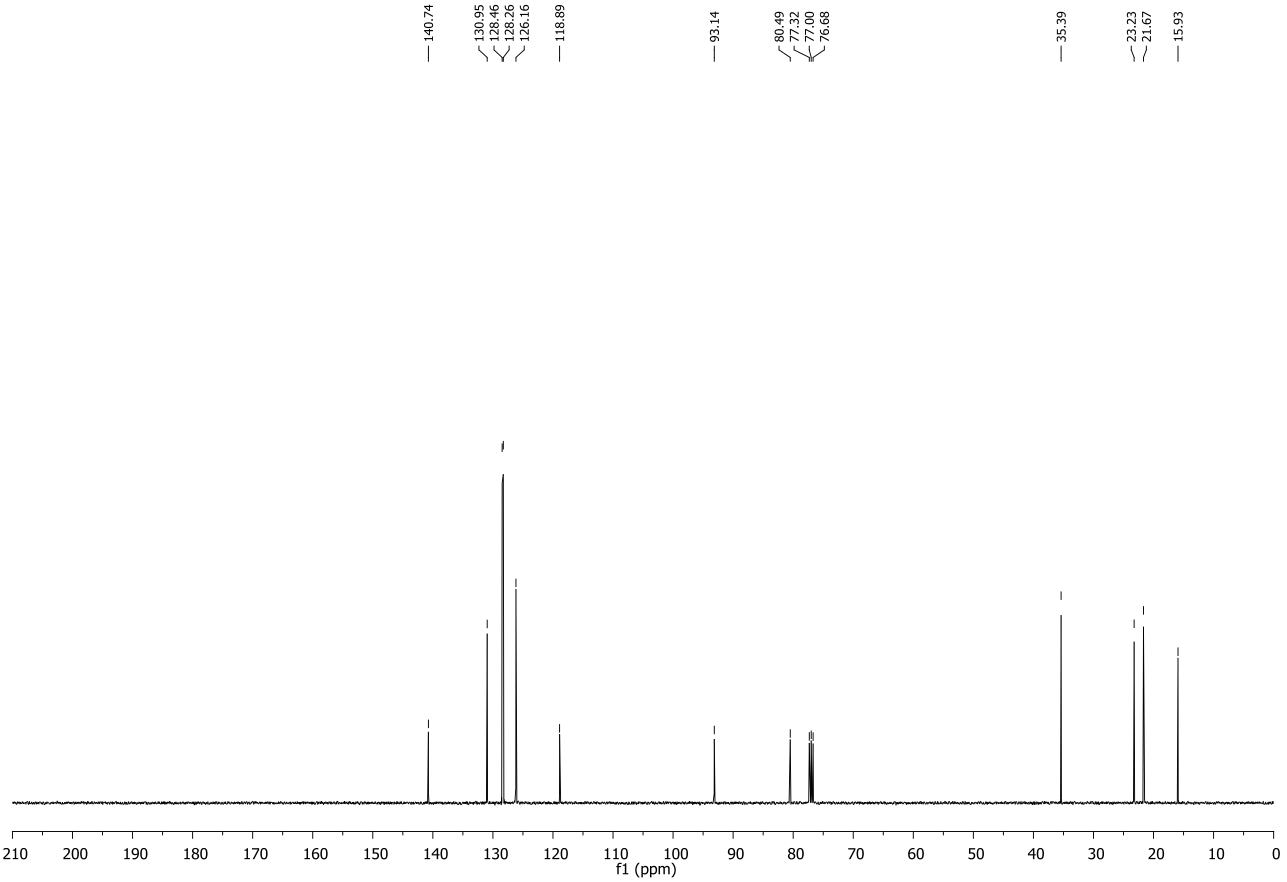
140.74
130.95
128.46
128.26
126.16
118.89
93.14
80.49
77.32
77.00
76.68
35.39
23.23
21.67
15.93
210 200 190 180 170 160 150 140 130 120 110 100 90 80 70 60 50 40 30 20 10 0
f1 (ppm)

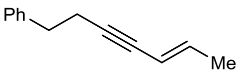
Ph
Me

**14**

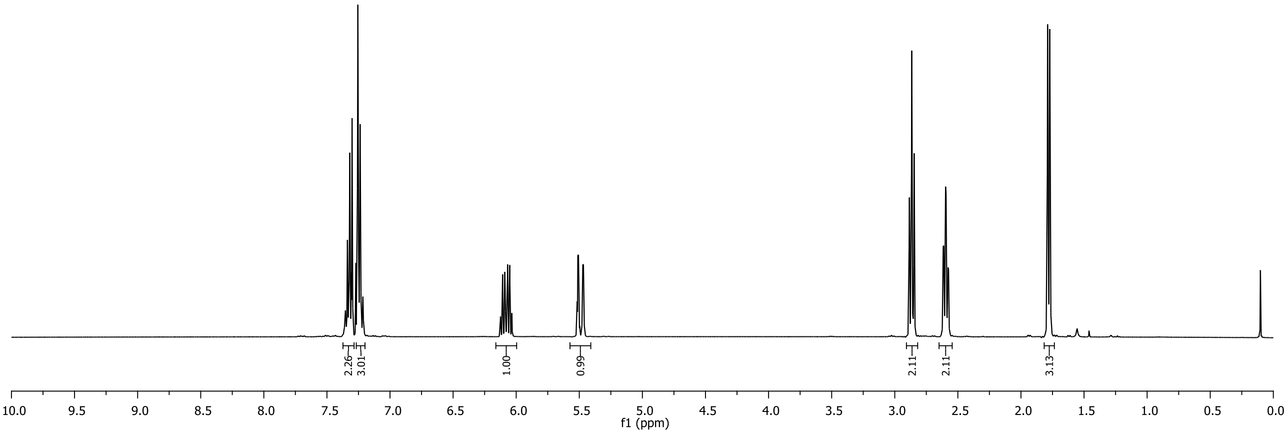
2.26
3.01
1.00
0.99
2.11
2.11
3.13
10.0
9.5
9.0
8.5
8.0
7.5
7.0
6.5
6.0
5.5
5.0
4.5
4.0
3.5
3.0
2.5
2.0
1.5
1.0
0.5
0.0
f1 (ppm)

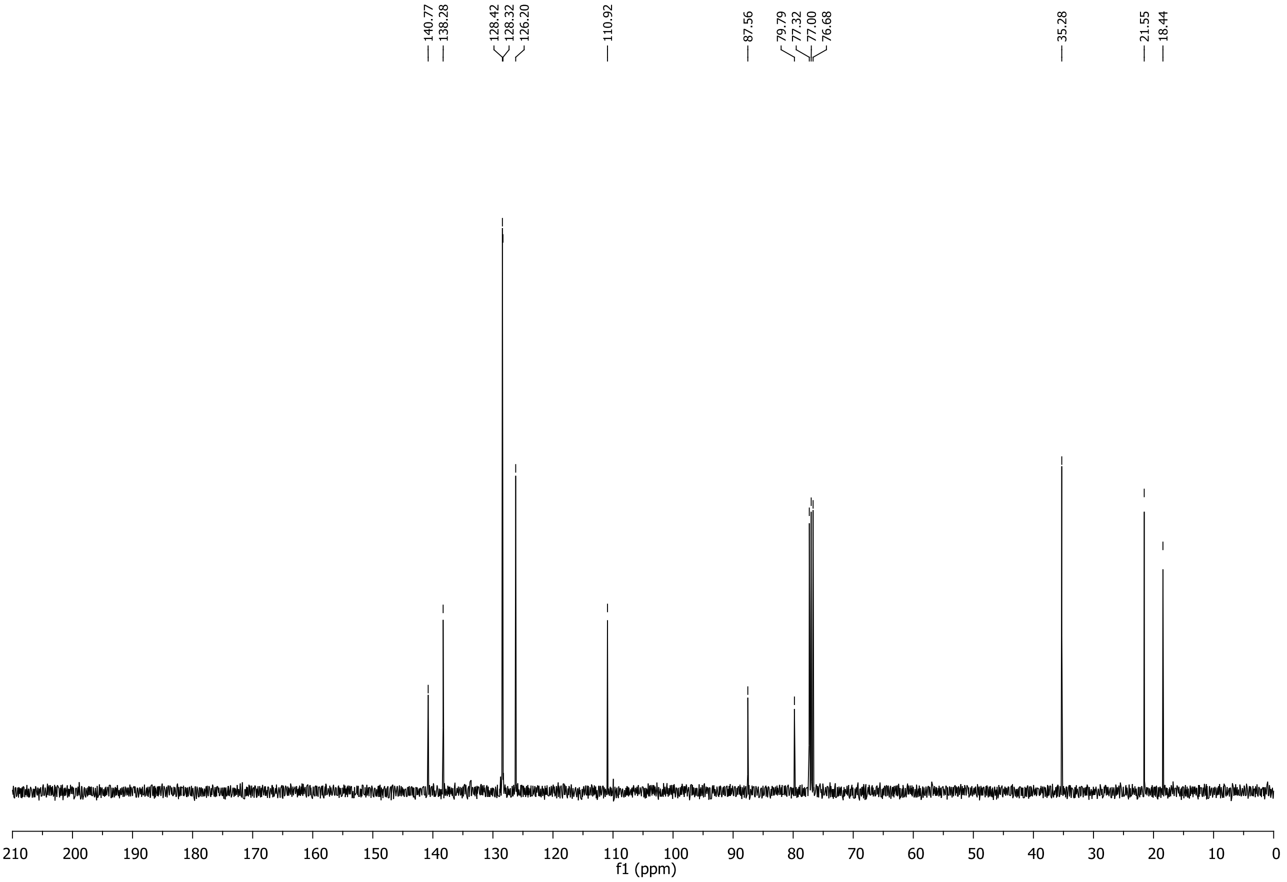
140.77
138.28
128.42
128.32
126.20
110.92
87.56
79.79
77.32
77.00
76.68
35.28
21.55
18.44
210
200
190
180
170
160
150
140
130
120
110
100
90
80
70
60
50
40
30
20
10
0
f1 (ppm)

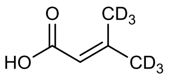
O
CD3
HO
CD3

$[D]_6$-**20**

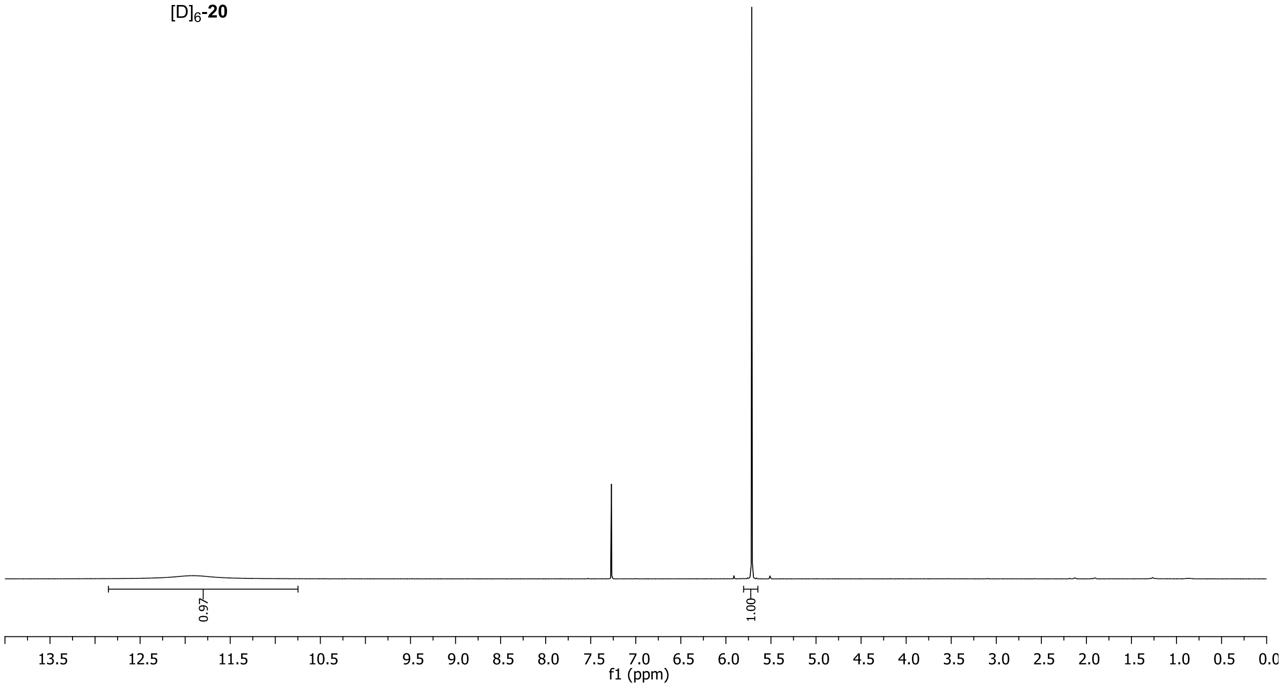
0.97
1.00
13.5
12.5
11.5
10.5
9.5
9.0
8.5
8.0
7.5
7.0
6.5
6.0
5.5
5.0
4.5
4.0
3.5
3.0
2.5
2.0
1.5
1.0
0.5
f1 (ppm)

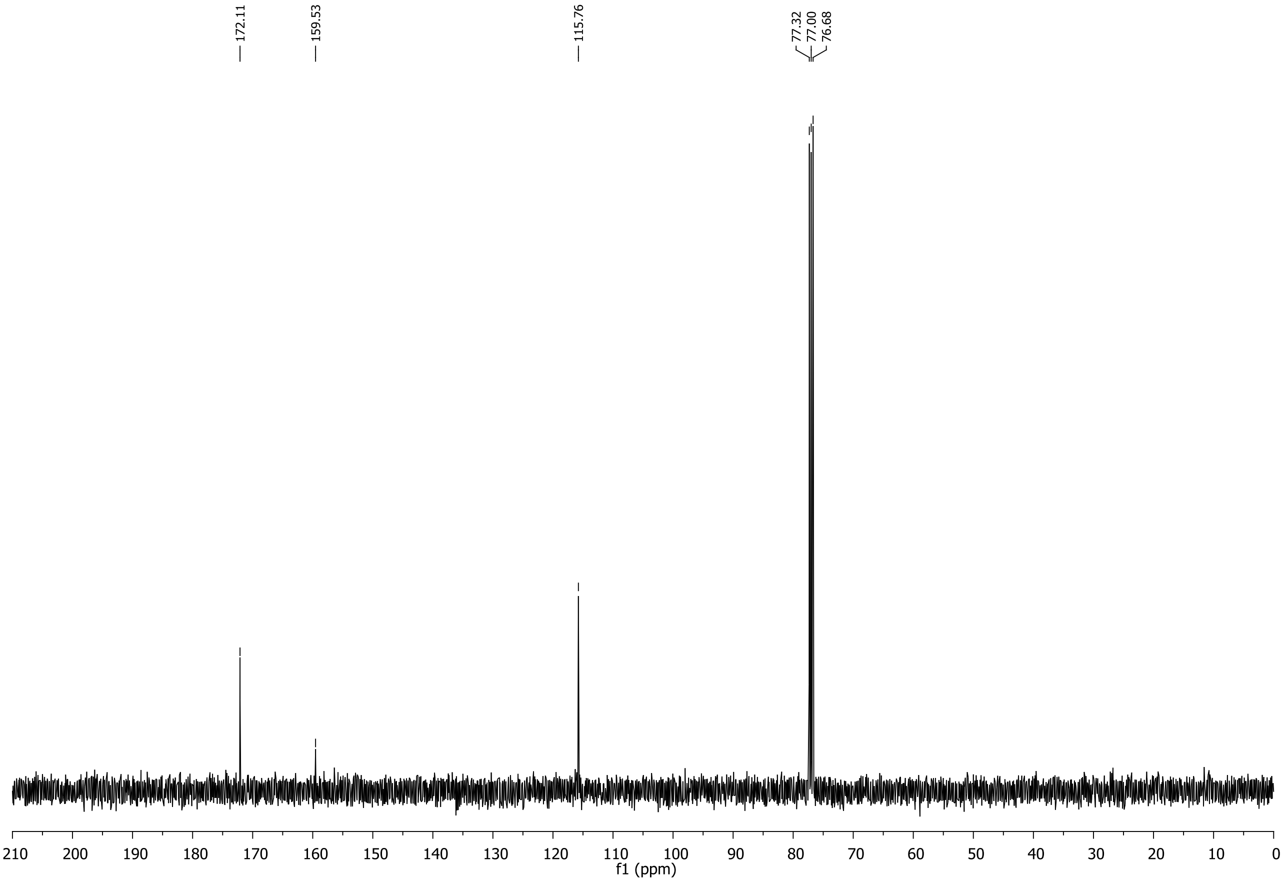
172.11
159.53
115.76
77.32
77.00
76.68
210
200
190
180
170
160
150
140
130
120
110
100
90
80
70
60
50
40
30
20
10
0
f1 (ppm)

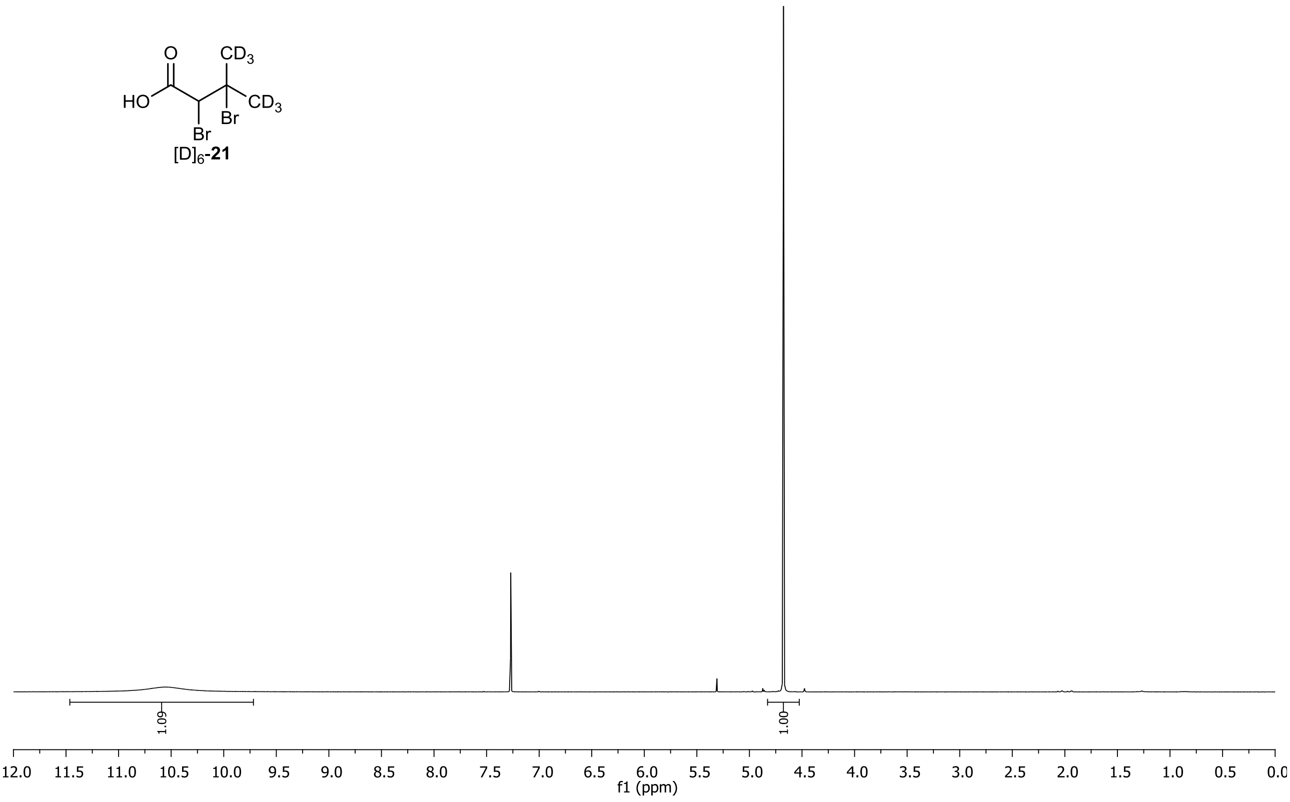
O
CD3
HO
CD3
Br
Br
1.09
1.00
12.0 11.5 11.0 10.5 10.0 9.5 9.0 8.5 8.0 7.5 7.0 6.5 6.0 5.5 5.0 4.5 4.0 3.5 3.0 2.5 2.0 1.5 1.0 0.5 0.0
f1 (ppm)

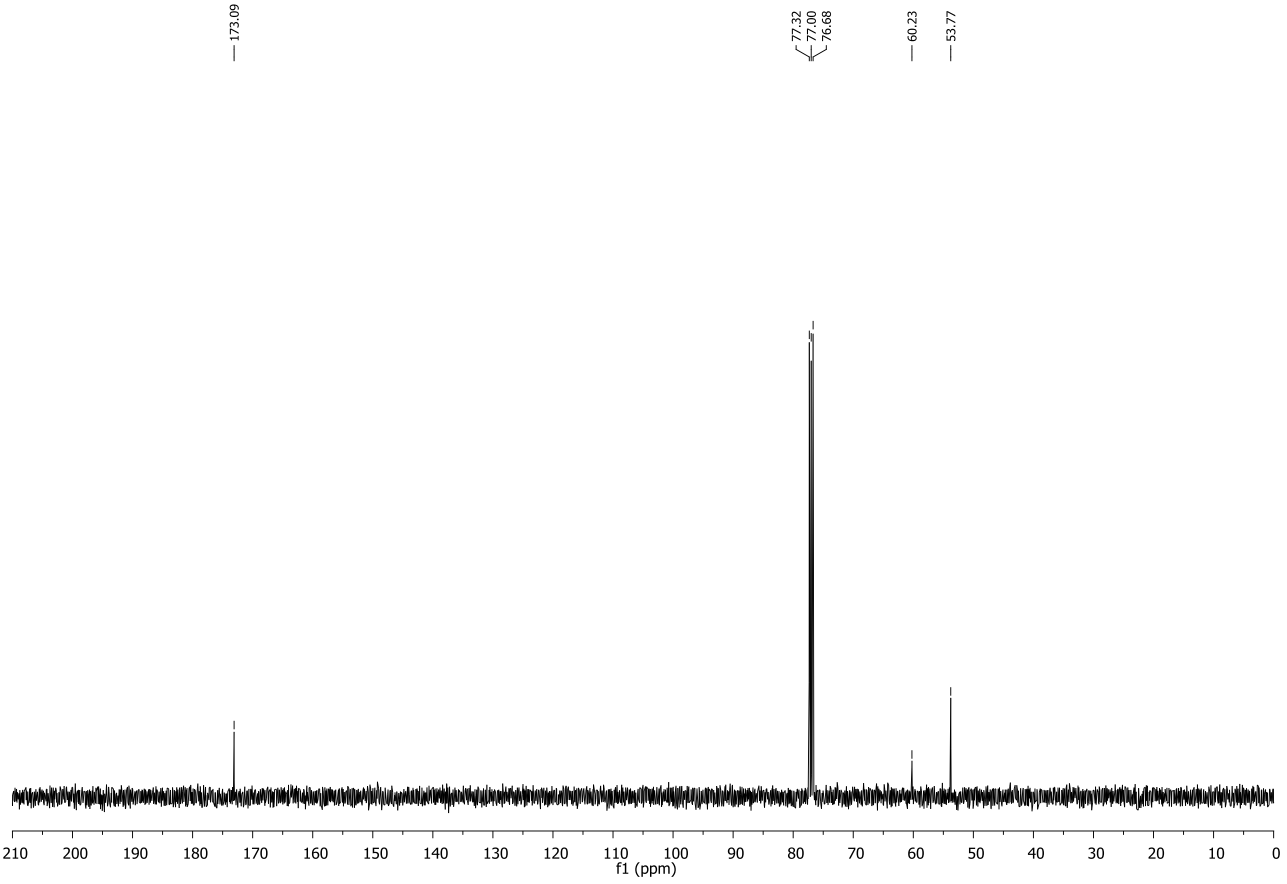
173.09
77.32
77.00
76.68
60.23
53.77
210 200 190 180 170 160 150 140 130 120 110 100 90 80 70 60 50 40 30 20 10 0
f1 (ppm)

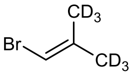
CD3
Br
CD3

$[D]_6$-**22**

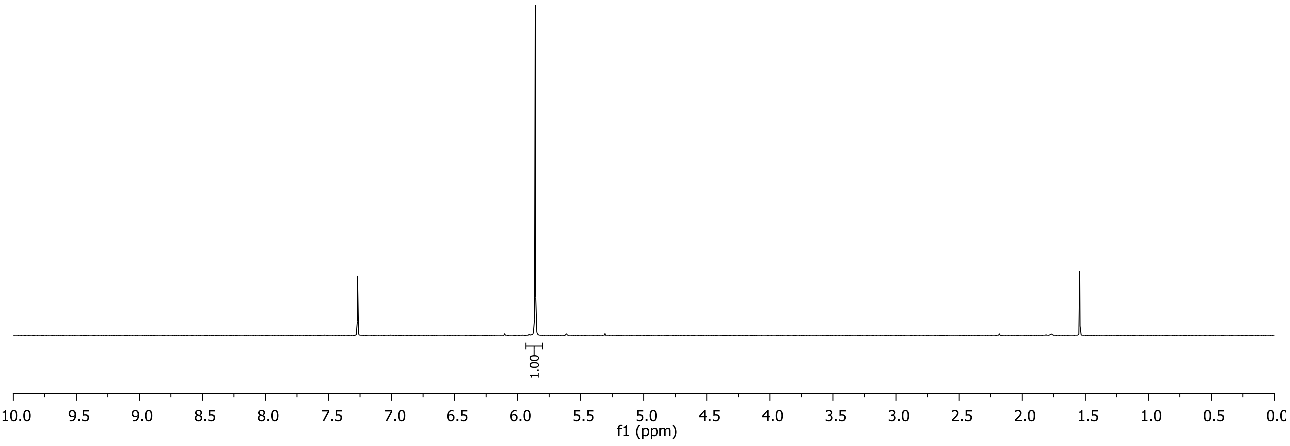
1.00
f1 (ppm)

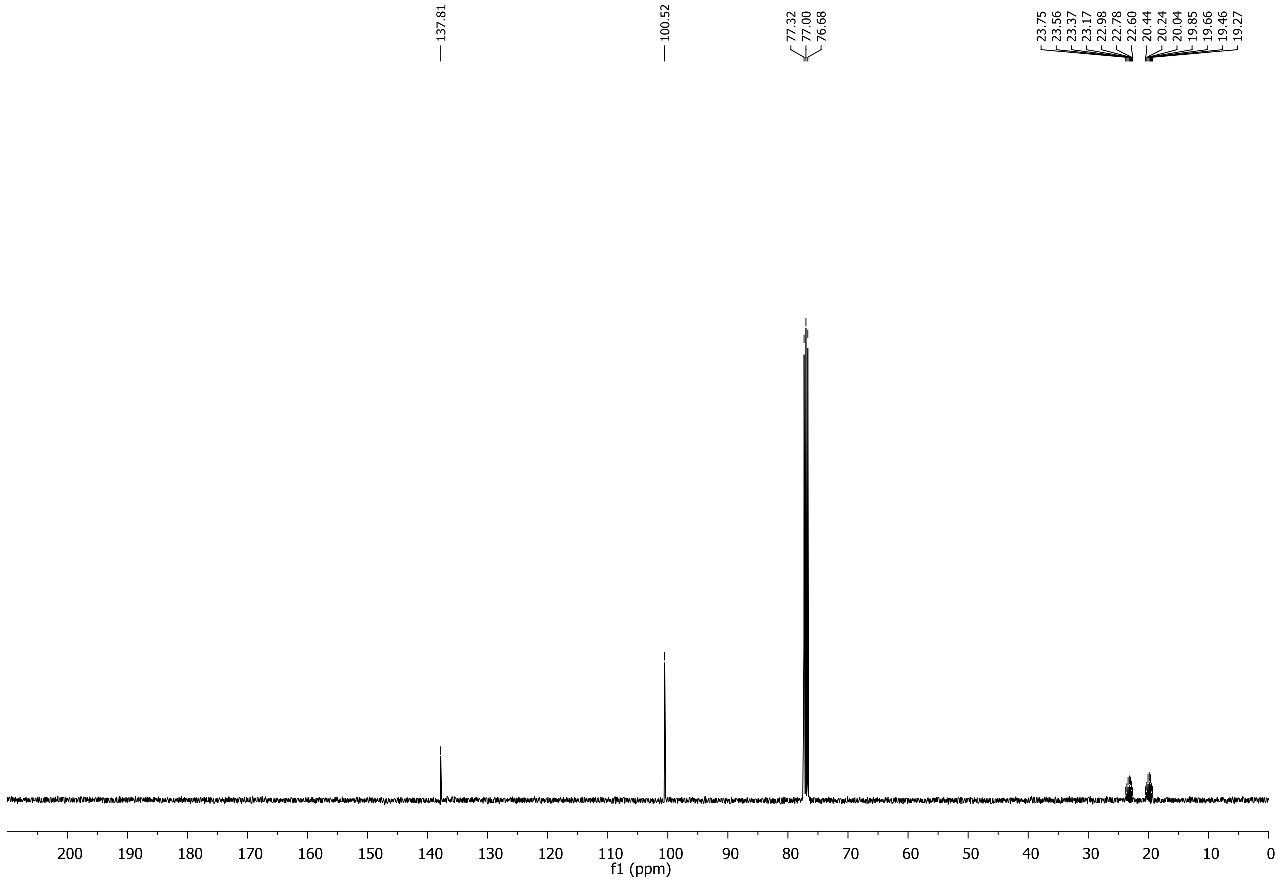
137.81
100.52
77.32
77.00
76.68
23.75
23.56
23.37
23.17
22.98
22.78
22.60
20.44
20.24
20.04
19.85
19.66
19.46
19.27
f1 (ppm)

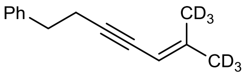
Ph
CD3
CD3

[D]$_6$-**2a**

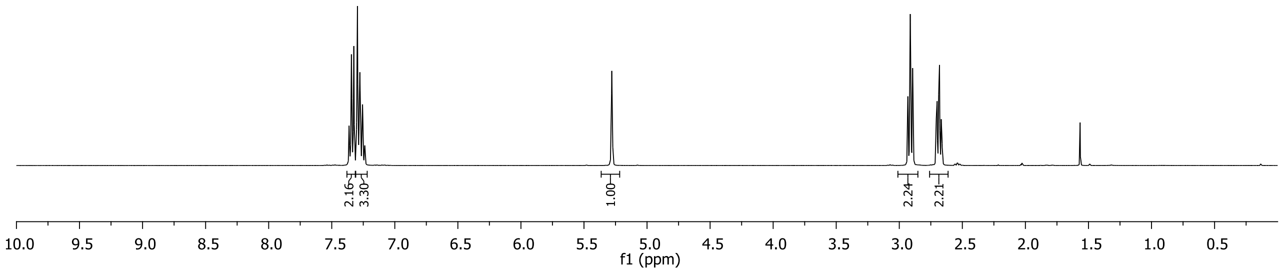
2.16
3.30
1.00
2.24
2.21
10.0 9.5 9.0 8.5 8.0 7.5 7.0 6.5 6.0 5.5 5.0 4.5 4.0 3.5 3.0 2.5 2.0 1.5 1.0 0.5
f1 (ppm)

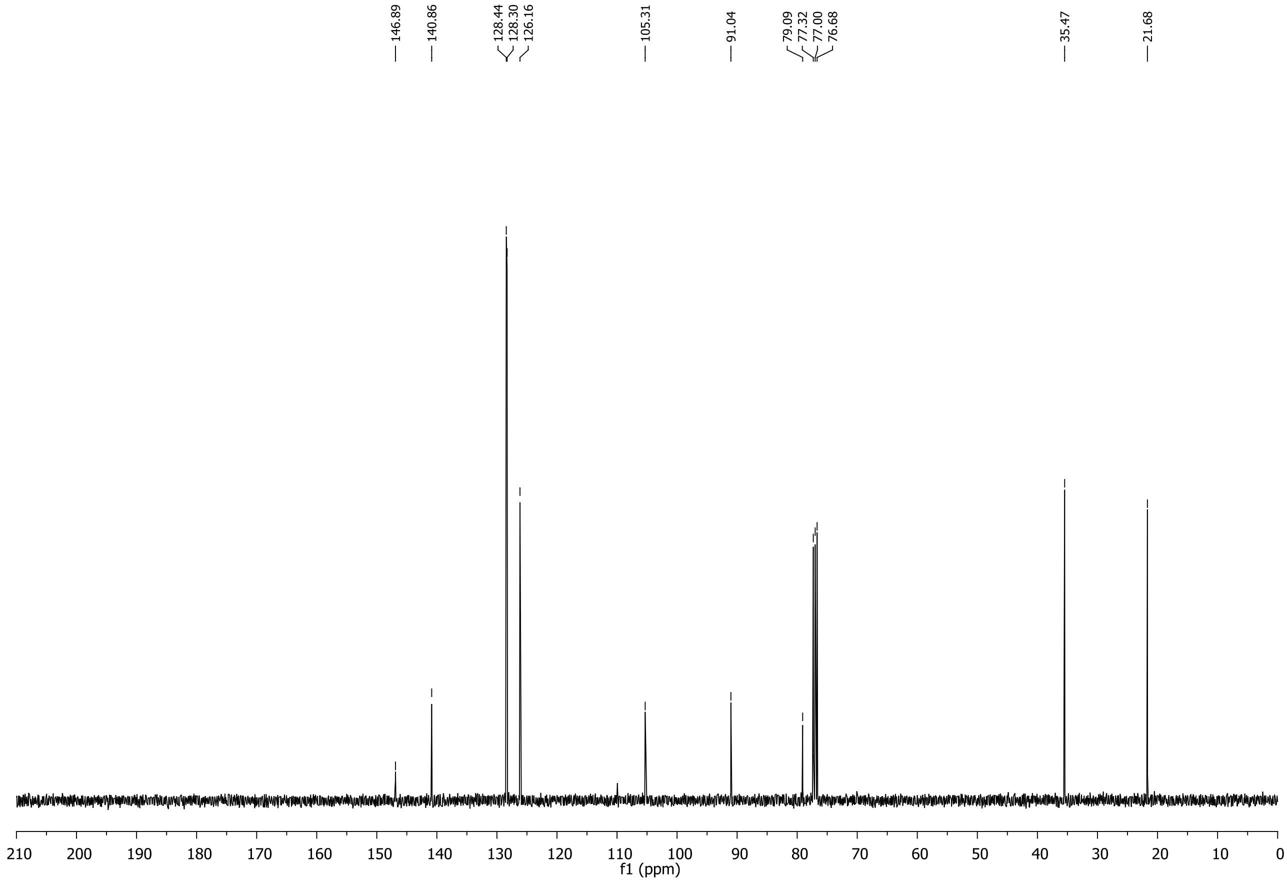
146.89
140.86
128.44
128.30
126.16
105.31
91.04
79.09
77.32
77.00
76.68
35.47
21.68
210 200 190 180 170 160 150 140 130 120 110 100 90 80 70 60 50 40 30 20 10 0
f1 (ppm)

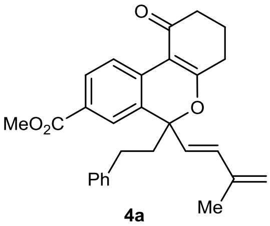
O
MeO$_2$C
O
Ph
Me

**4a**

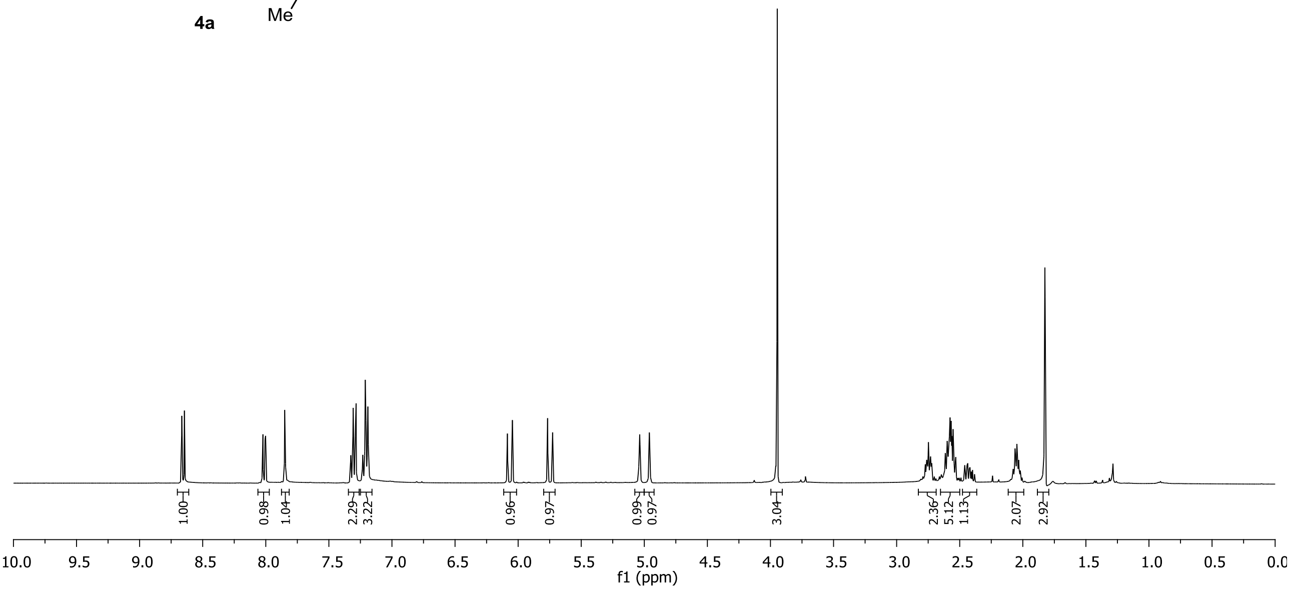
1.00
0.98
1.04
2.29
3.22
0.96
0.97
0.99
0.97
3.04
2.36
5.12
1.13
2.07
2.92
10.0 9.5 9.0 8.5 8.0 7.5 7.0 6.5 6.0 5.5 5.0 4.5 4.0 3.5 3.0 2.5 2.0 1.5 1.0 0.5 0.0
f1 (ppm)

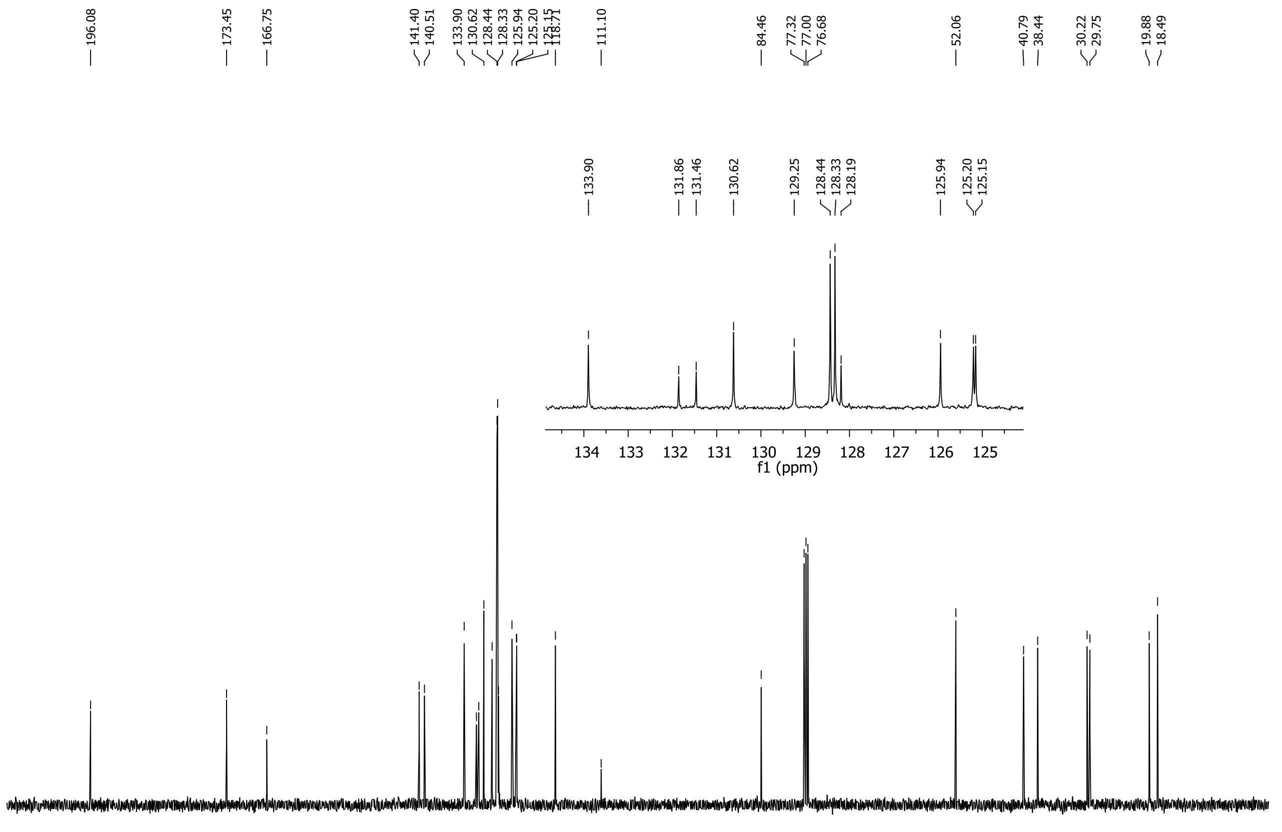
196.08
173.45
166.75
141.40
140.51
133.90
130.62
128.44
128.33
125.94
125.20
125.15
118.71
111.10
84.46
77.32
77.00
76.68
52.06
40.79
38.44
30.22
29.75
19.88
18.49
133.90
131.86
131.46
130.62
129.25
128.44
128.33
128.19
125.94
125.20
125.15
134 133 132 131 130 129 128 127 126 125
f1 (ppm)
210 200 190 180 170 160 150 140 130 120 110 100 90 80 70 60 50 40 30 20 10 0
f1 (ppm)

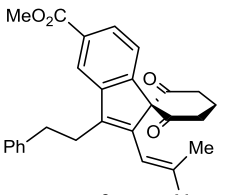

MeO2C
O
O
Ph
Me
Me


**3a**

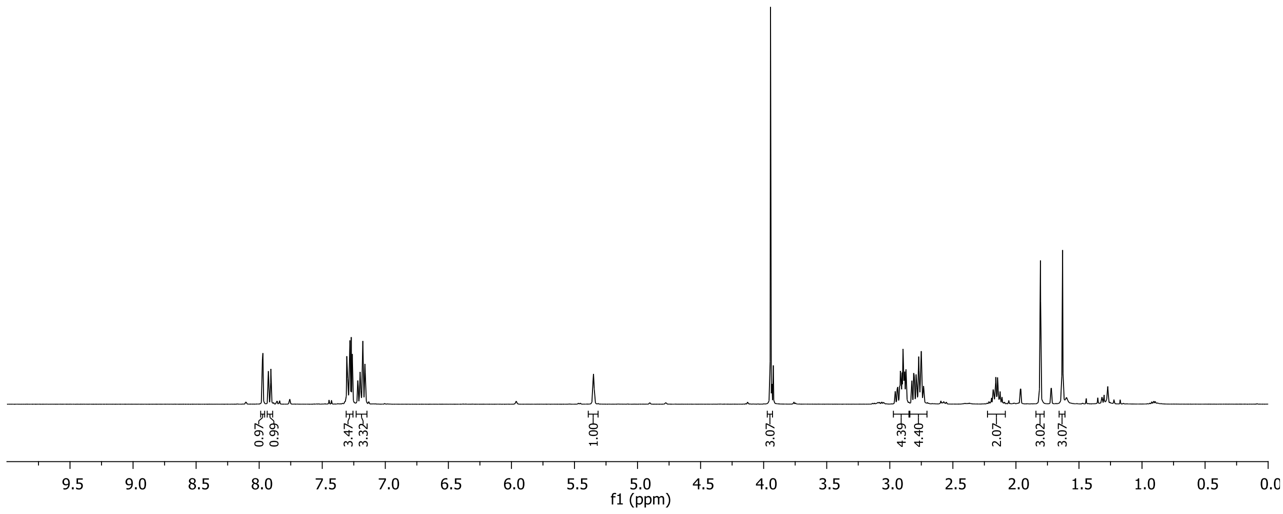

0.97
0.99
3.47
3.32
1.00
3.07
4.39
4.40
2.07
3.02
3.07
9.5 9.0 8.5 8.0 7.5 7.0 6.5 6.0 5.5 5.0 4.5 4.0 3.5 3.0 2.5 2.0 1.5 1.0 0.5 0.0
f1 (ppm)


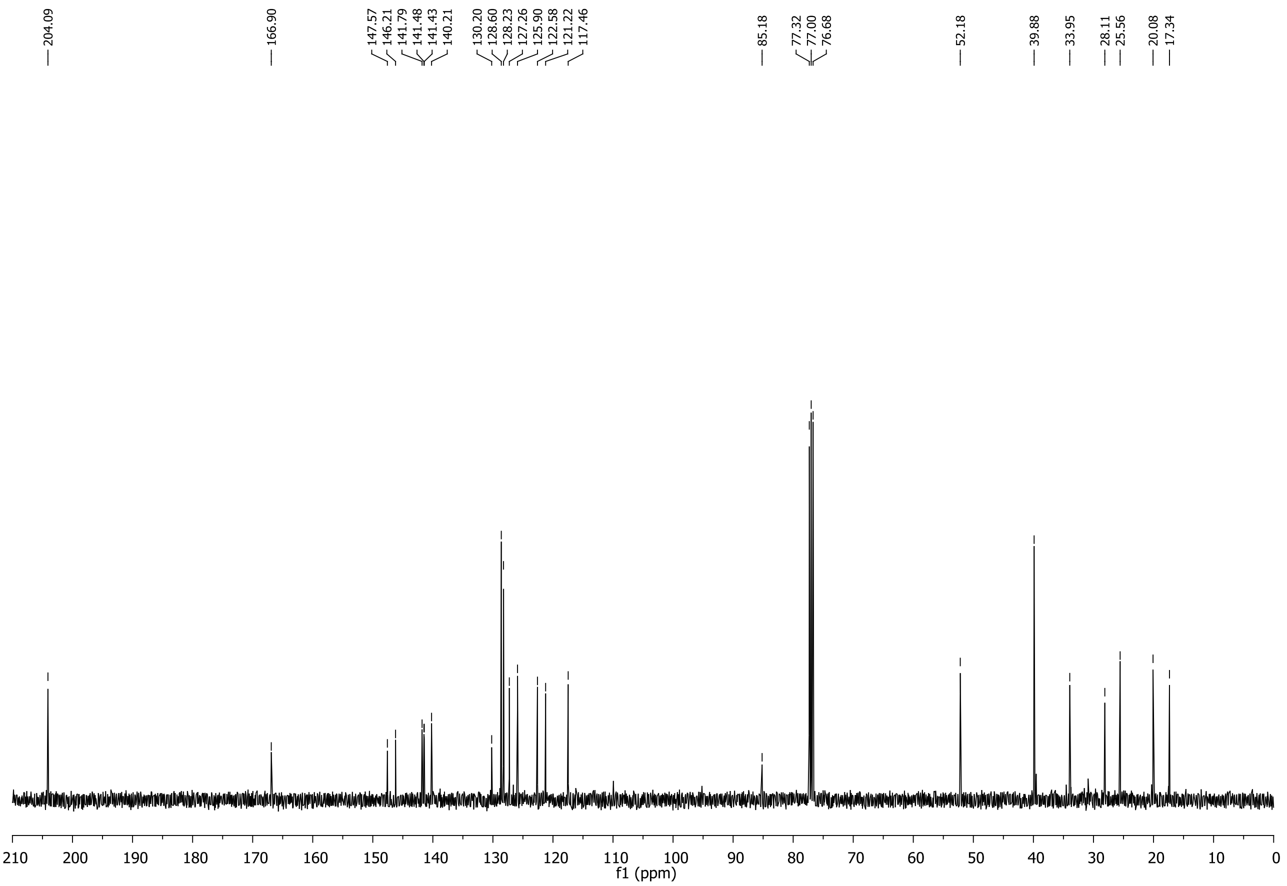

204.09
166.90
147.57
146.21
141.79
141.48
141.43
140.21
130.20
128.60
128.23
127.26
125.90
122.58
121.22
117.46
85.18
77.32
77.00
76.68
52.18
39.88
33.95
28.11
25.56
20.08
17.34
210 200 190 180 170 160 150 140 130 120 110 100 90 80 70 60 50 40 30 20 10 0
f1 (ppm)

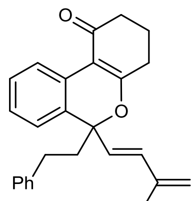

**4b**

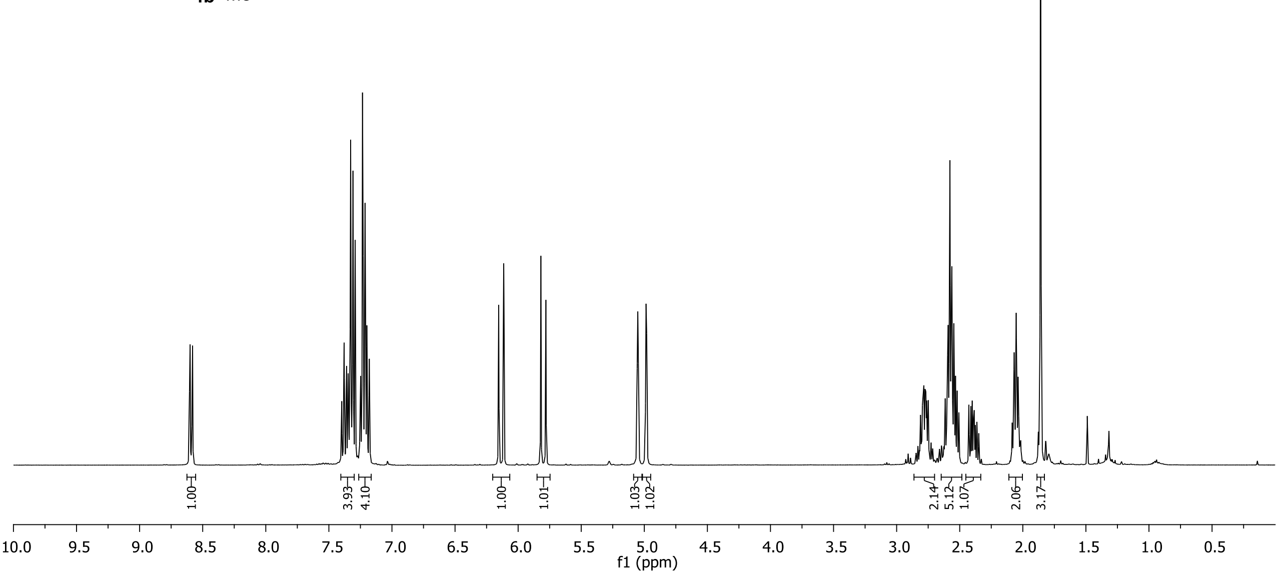
1.00
3.93
4.10
1.00
1.01
1.03
1.02
2.14
5.12
1.07
2.06
3.17
10.0 9.5 9.0 8.5 8.0 7.5 7.0 6.5 6.0 5.5 5.0 4.5 4.0 3.5 3.0 2.5 2.0 1.5 1.0 0.5
f1 (ppm)

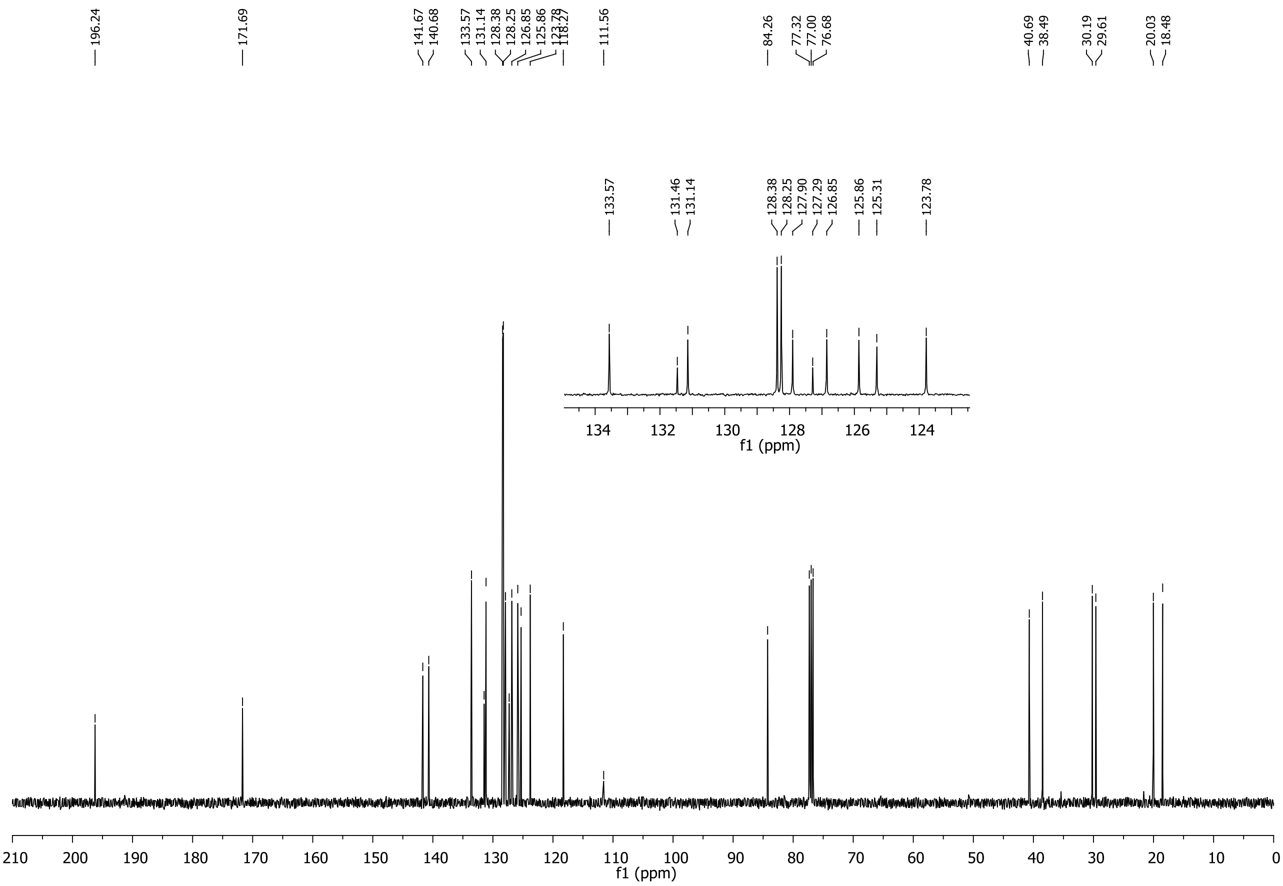
196.24
171.69
141.67
140.68
133.57
131.14
128.38
128.25
126.85
125.86
123.78
118.27
111.56
84.26
77.32
77.00
76.68
40.69
38.49
30.19
29.61
20.03
18.48
133.57
131.46
131.14
128.38
128.25
127.90
127.29
126.85
125.86
125.31
123.78
134 132 130 128 126 124
f1 (ppm)
210 200 190 180 170 160 150 140 130 120 110 100 90 80 70 60 50 40 30 20 10 0
f1 (ppm)

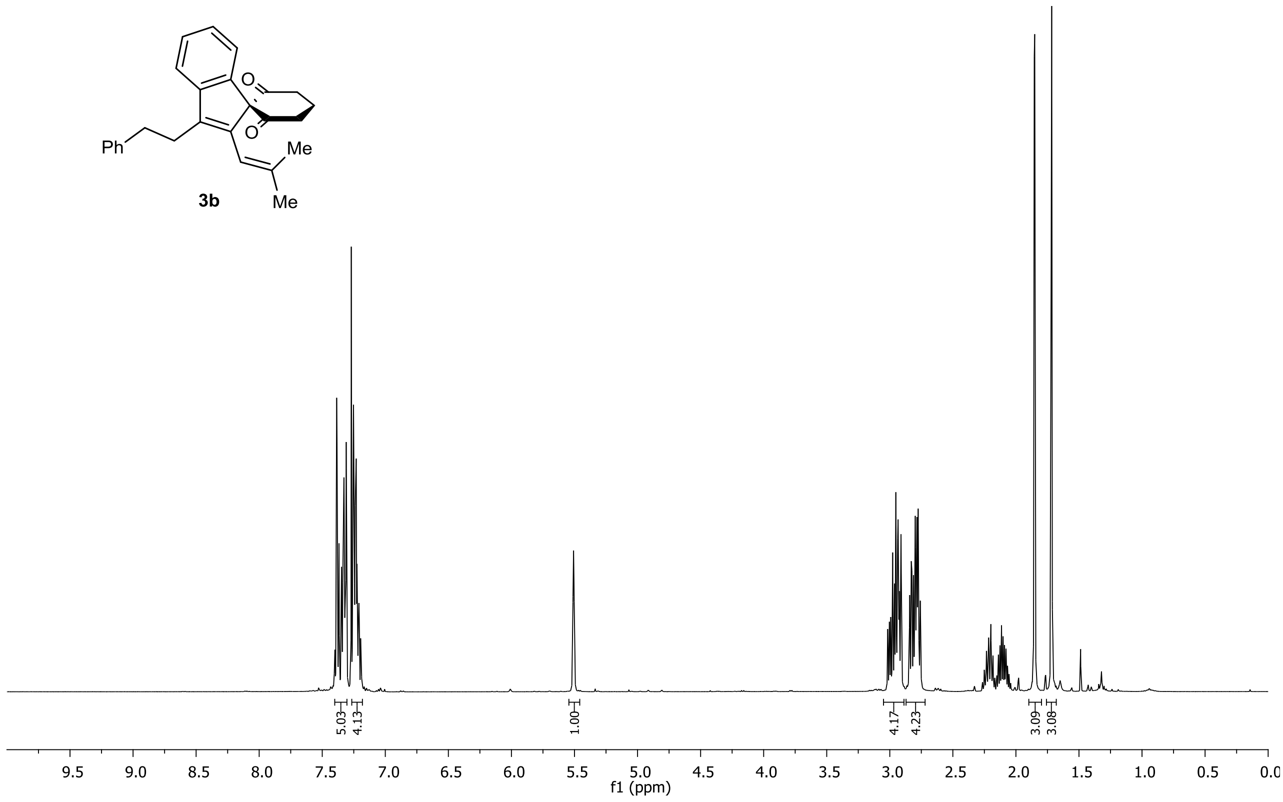
O
O
Ph
Me
Me
3b
5.03
4.13
1.00
4.17
4.23
3.09
3.08
f1 (ppm)

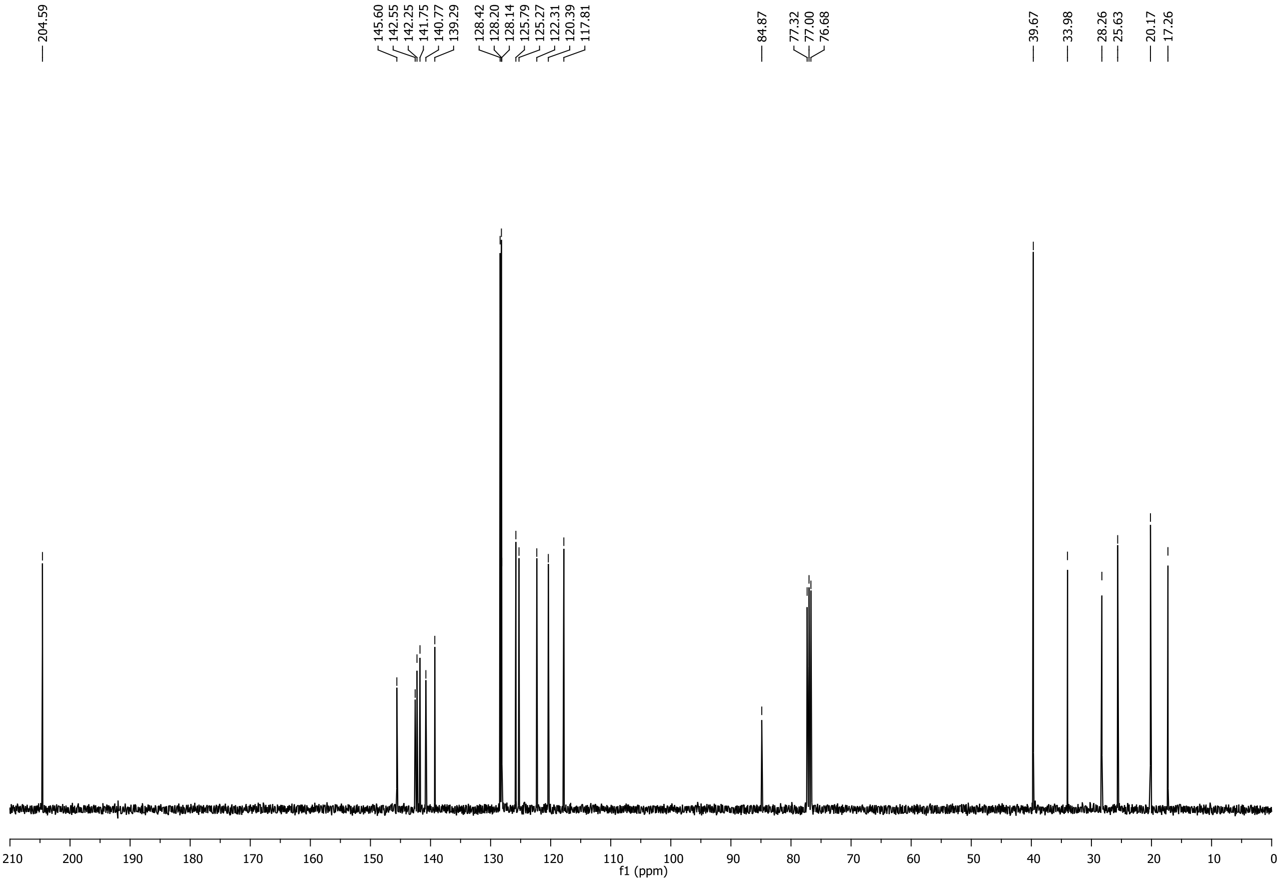
204.59
145.60
142.55
142.25
141.75
140.77
139.29
128.42
128.20
128.14
125.79
125.27
122.31
120.39
117.81
84.87
77.32
77.00
76.68
39.67
33.98
28.26
25.63
20.17
17.26
f1 (ppm)

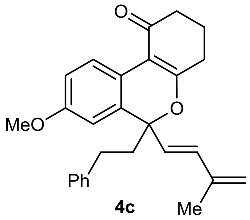
O
O
MeO
Ph
Me

**4c**

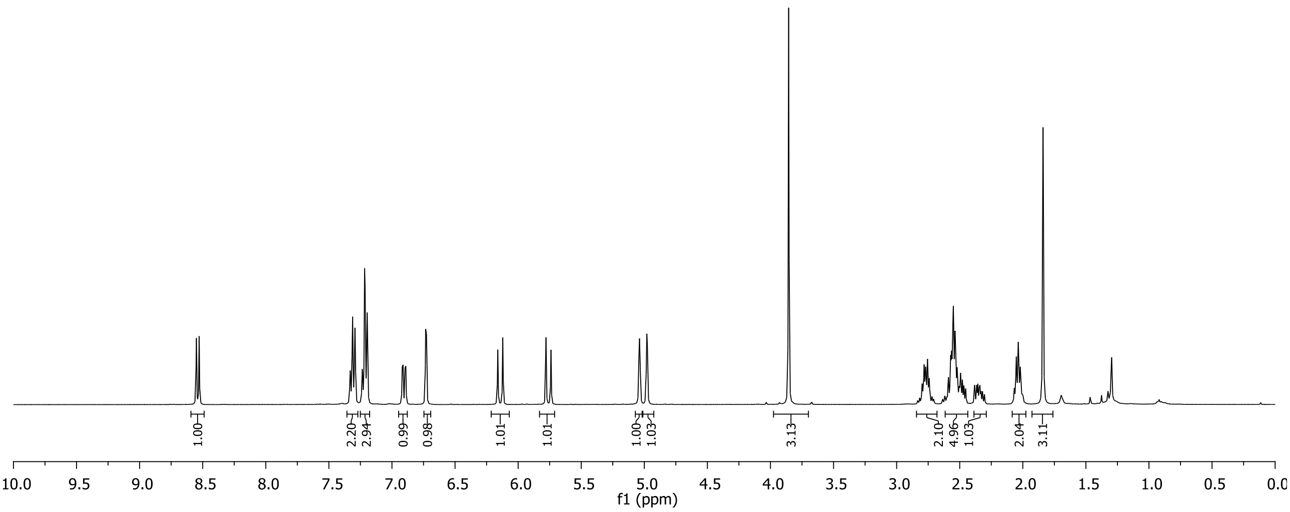
1.00
2.20
2.94
0.99
0.98
1.01
1.01
1.00
1.03
3.13
2.10
4.96
1.03
2.04
3.11
f1 (ppm)

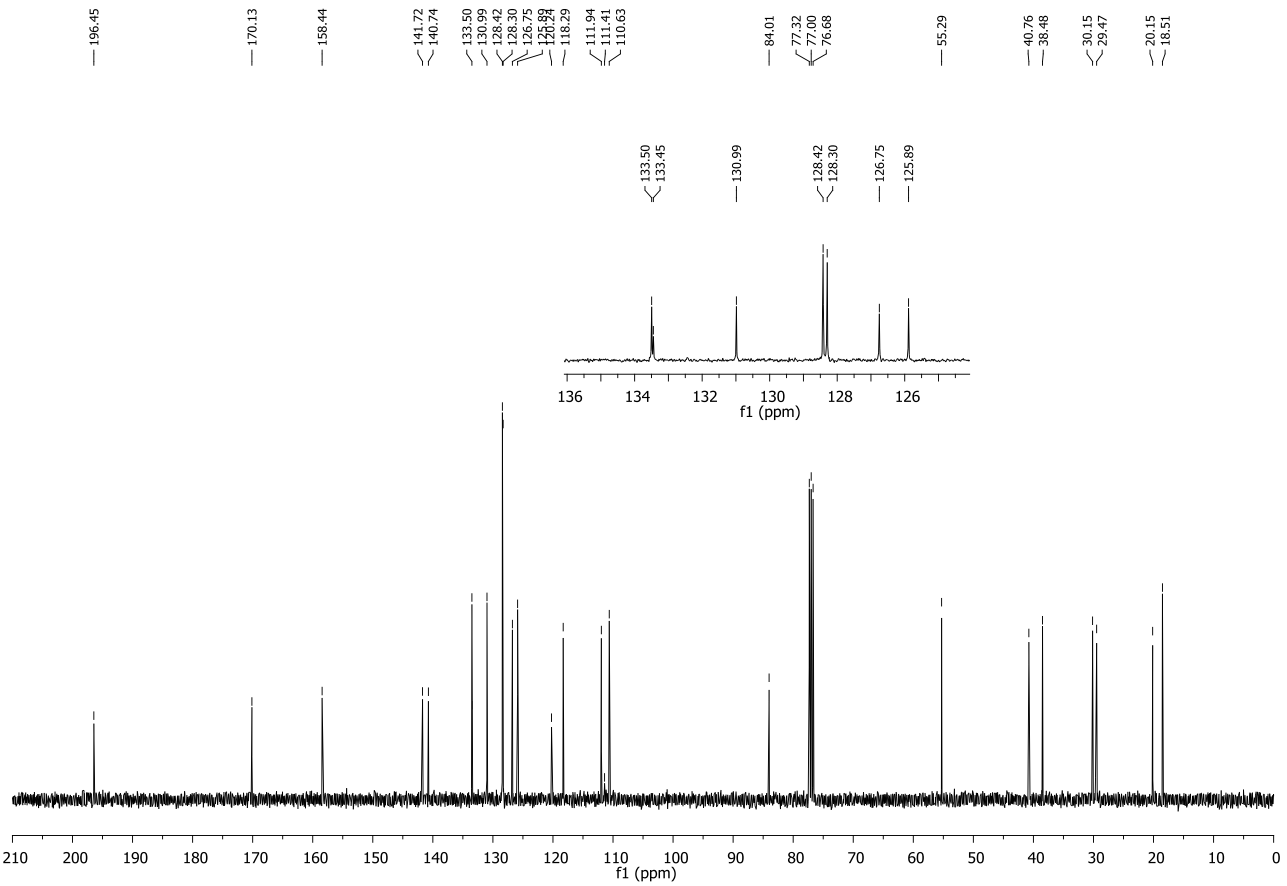
196.45
170.13
158.44
141.72
140.74
133.50
130.99
128.42
128.30
126.75
125.89
120.24
118.29
111.94
111.41
110.63
84.01
77.32
77.00
76.68
55.29
40.76
38.48
30.15
29.47
20.15
18.51
133.50
133.45
130.99
128.42
128.30
126.75
125.89
f1 (ppm)
f1 (ppm)

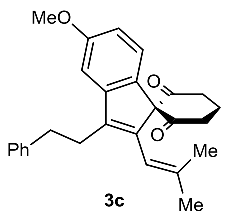
MeO
O
O
Ph
Me
Me

**3c**

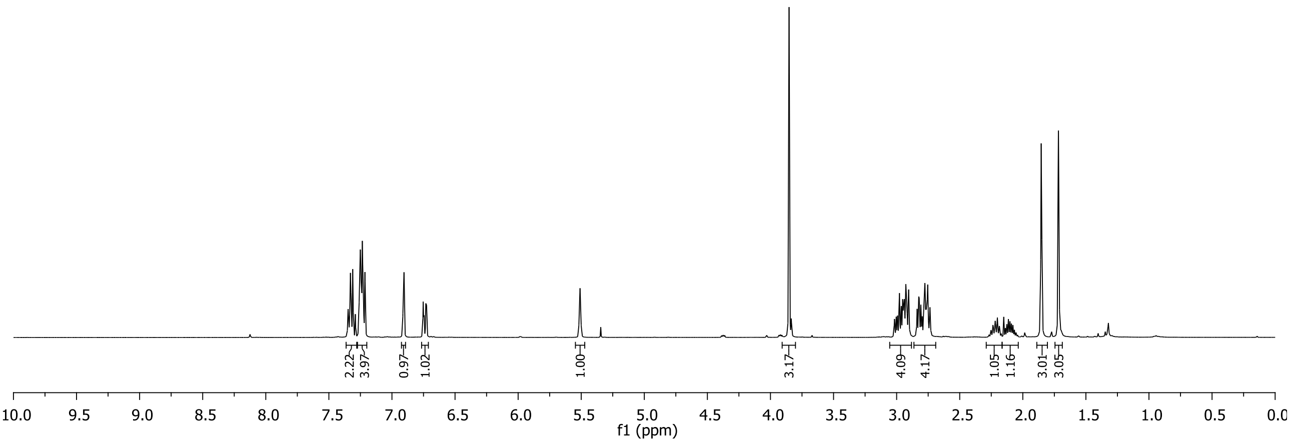
2.22
3.97
0.97
1.02
1.00
3.17
4.09
4.17
1.05
1.16
3.01
3.05
10.0 9.5 9.0 8.5 8.0 7.5 7.0 6.5 6.0 5.5 5.0 4.5 4.0 3.5 3.0 2.5 2.0 1.5 1.0 0.5 0.0
f1 (ppm)

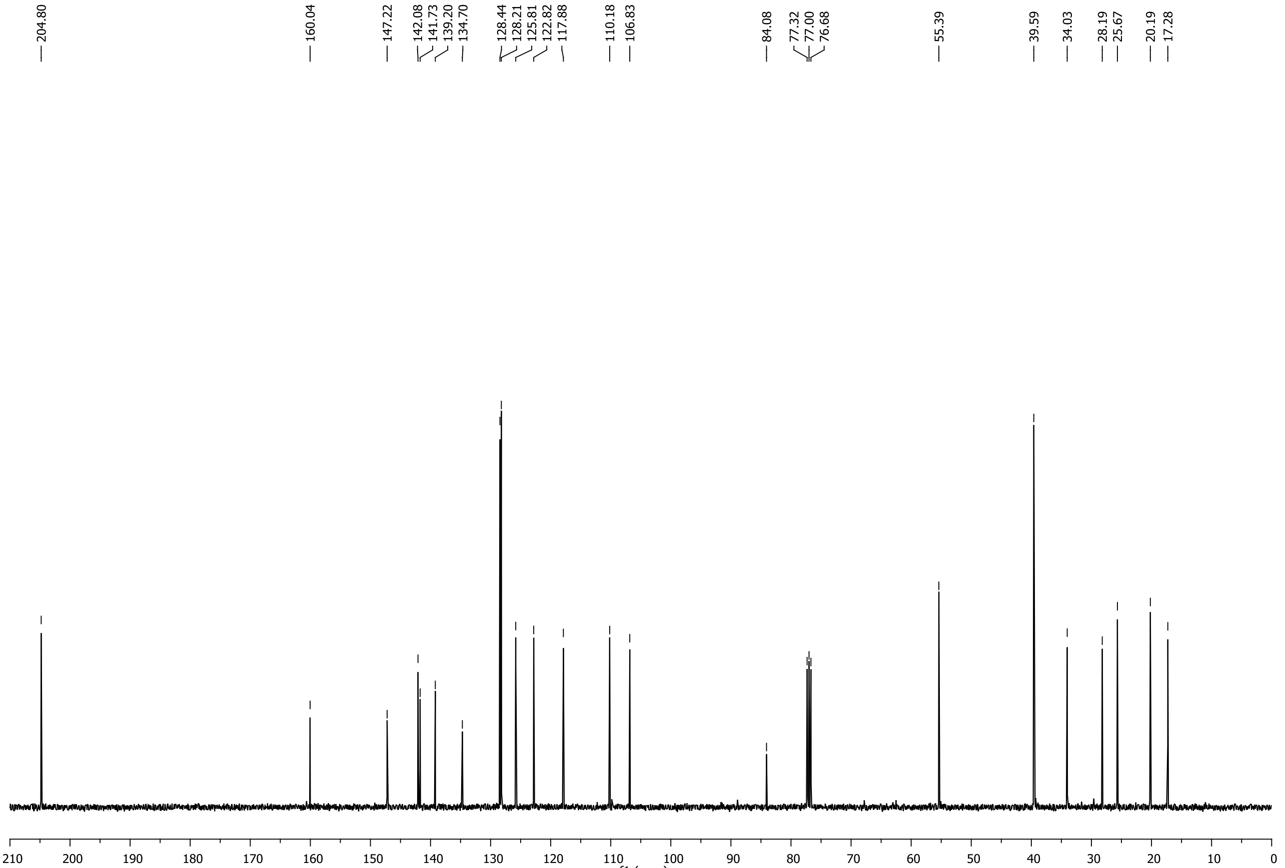
204.80
160.04
147.22
142.08
141.73
139.20
134.70
128.44
128.21
125.81
122.82
117.88
110.18
106.83
84.08
77.32
77.00
76.68
55.39
39.59
34.03
28.19
25.67
20.19
17.28
210 200 190 180 170 160 150 140 130 120 110 100 90 80 70 60 50 40 30 20 10 0
f1 (ppm)

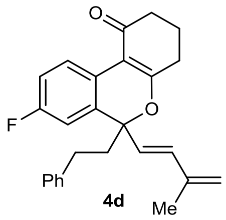


**4d**

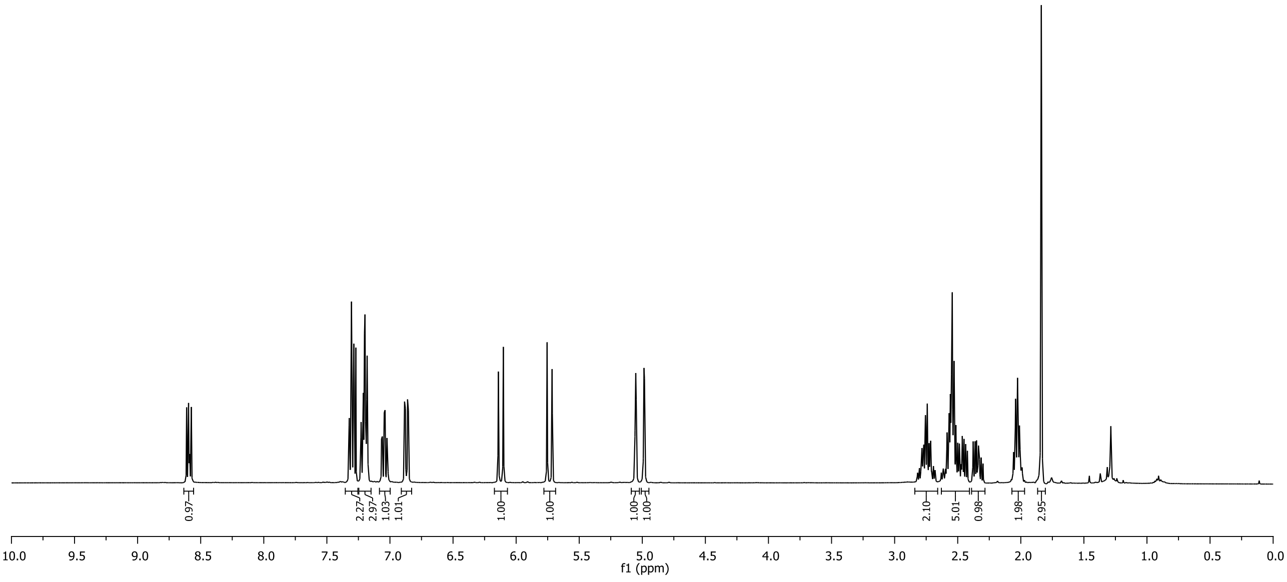

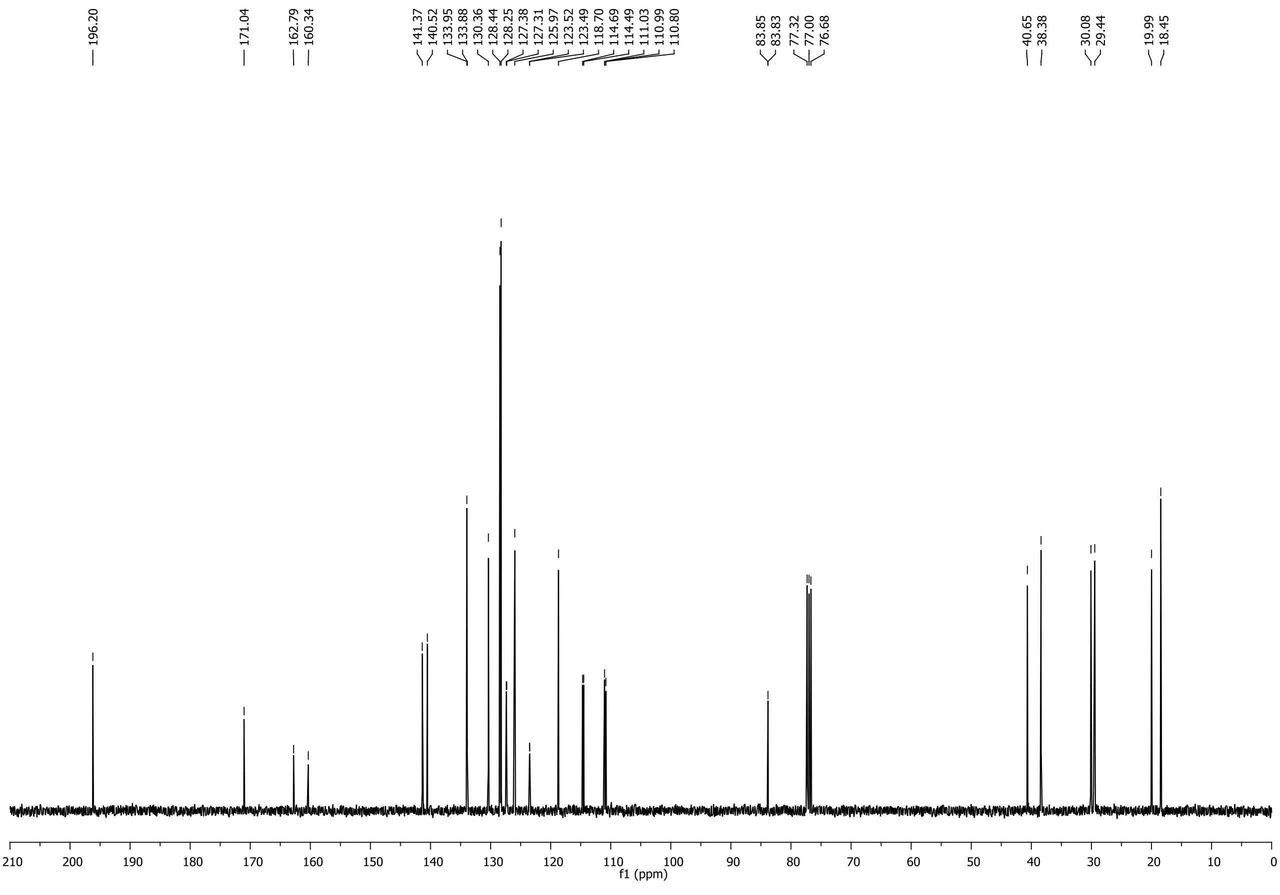

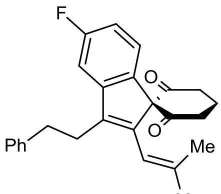


**3d**

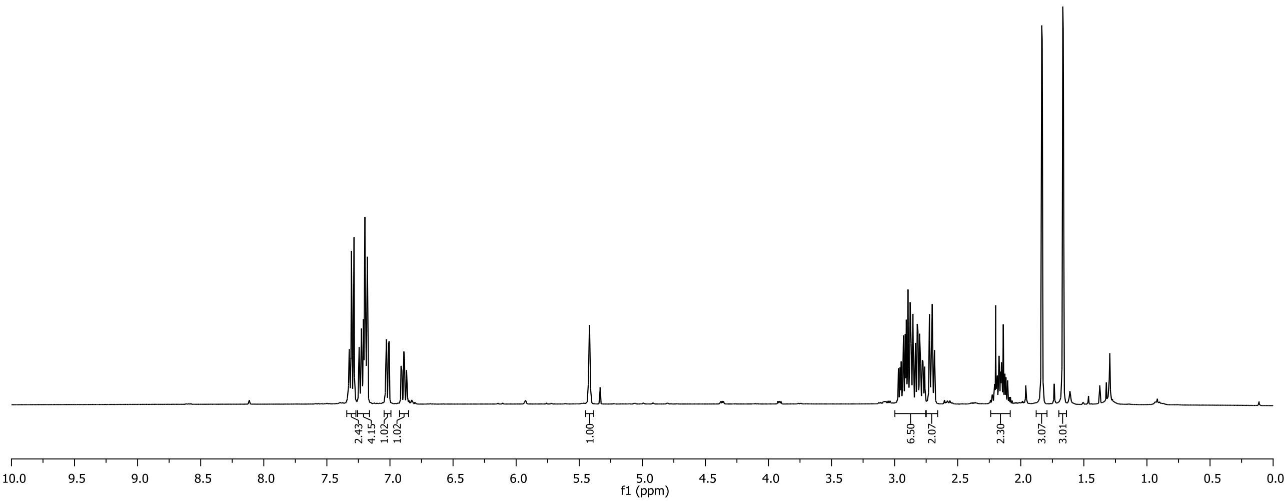


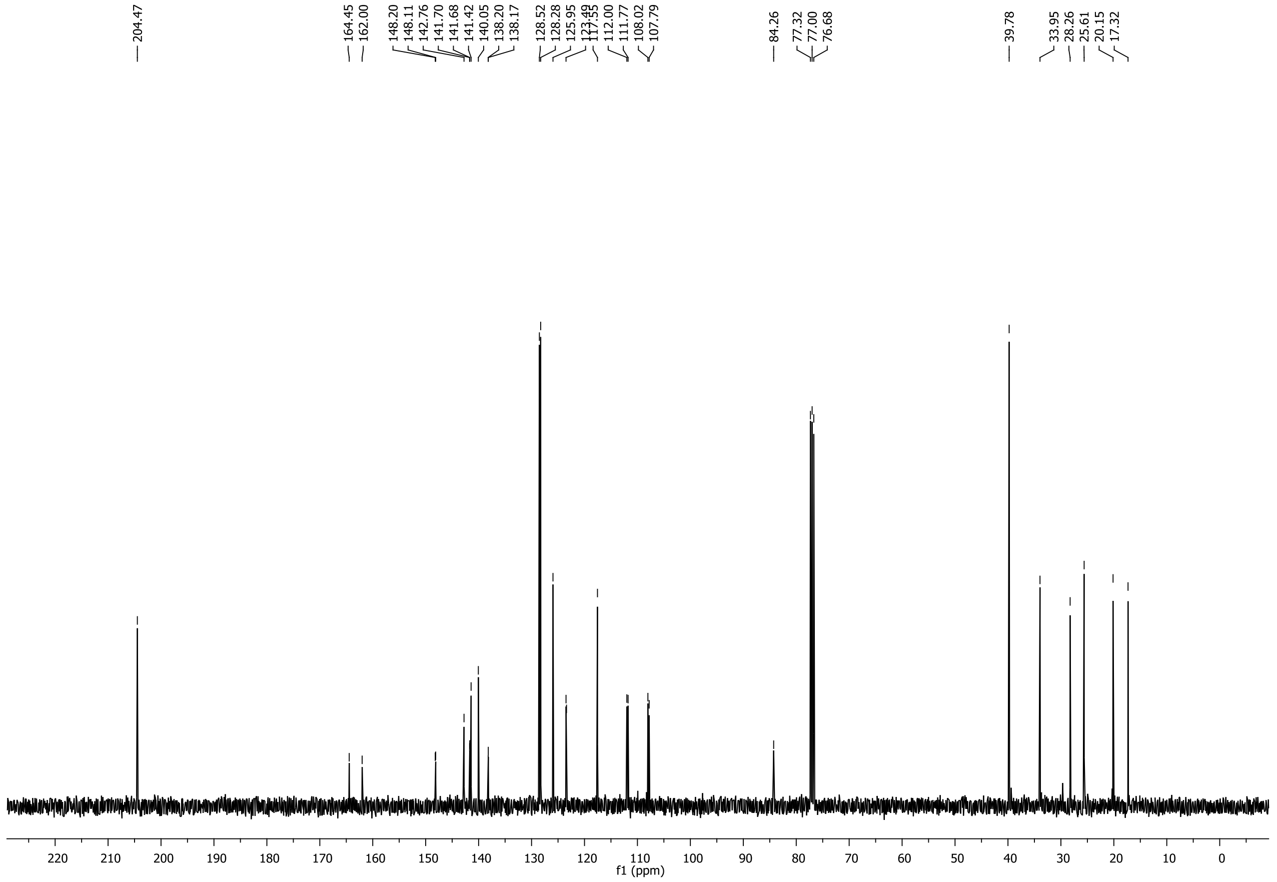

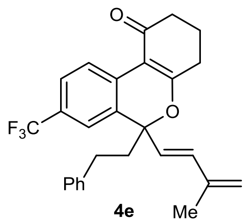
O
O
$F_3C$
Ph
Me

**4e**

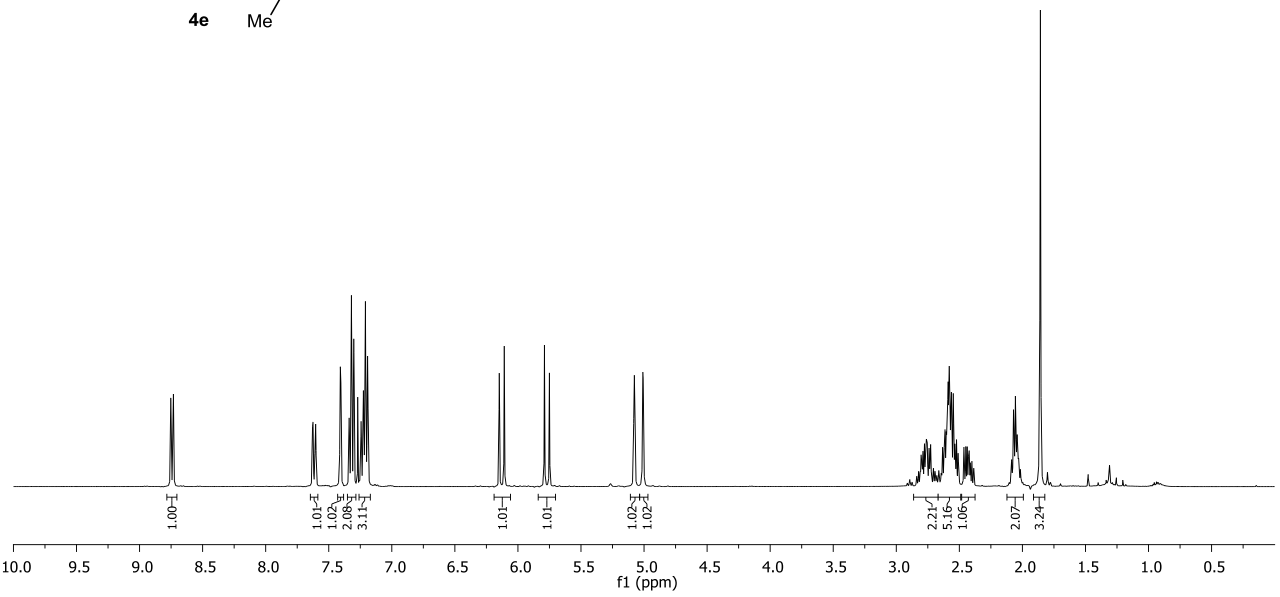
1.00
1.01
1.02
2.08
3.11
1.01
1.01
1.02
1.02
2.21
5.16
1.06
2.07
3.24
10.0 9.5 9.0 8.5 8.0 7.5 7.0 6.5 6.0 5.5 5.0 4.5 4.0 3.5 3.0 2.5 2.0 1.5 1.0 0.5
f1 (ppm)

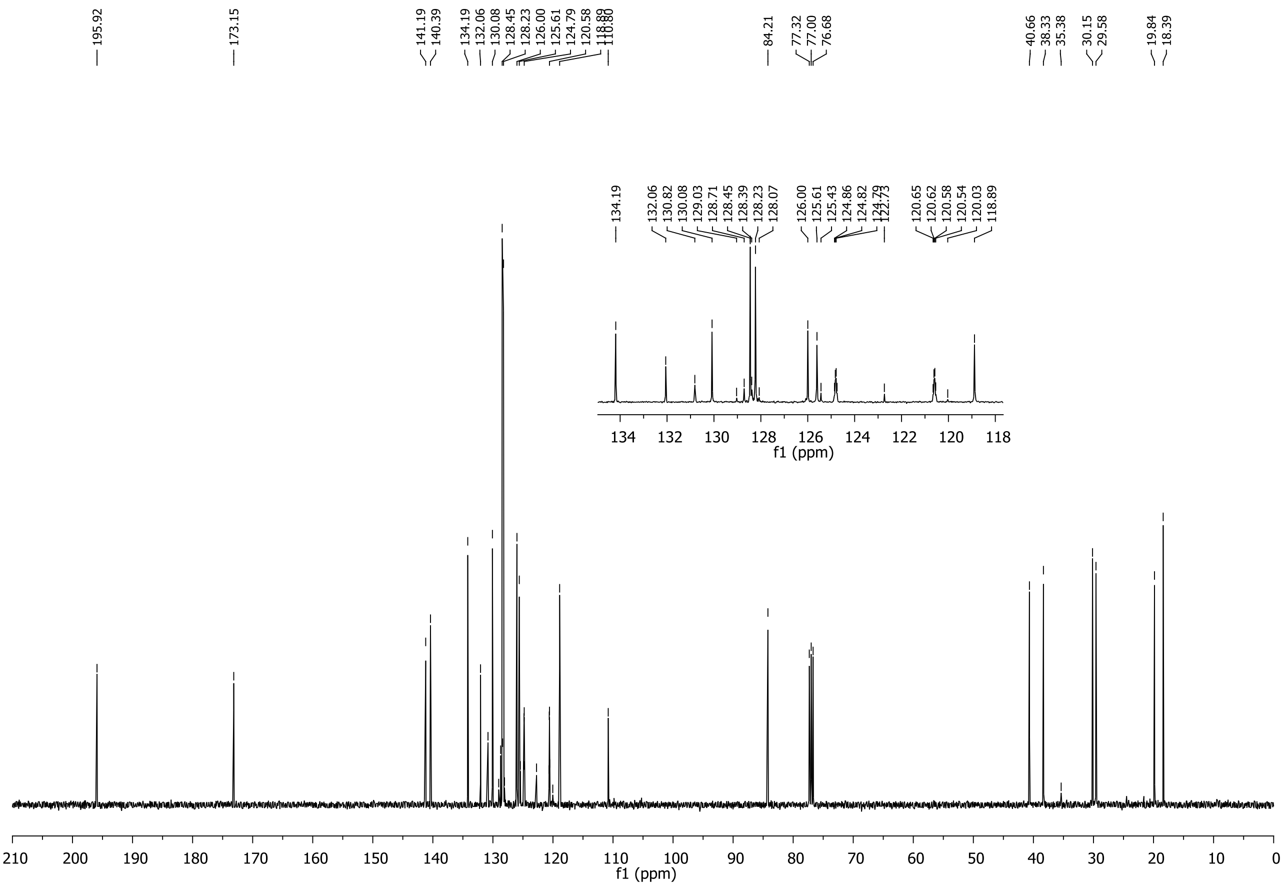
195.92
173.15
141.19
140.39
134.19
132.06
130.08
128.45
128.23
126.00
125.61
124.79
120.58
118.89
110.80
84.21
77.32
77.00
76.68
40.66
38.33
35.38
30.15
29.58
19.84
18.39
134.19
132.06
130.82
130.08
129.03
128.71
128.45
128.39
128.23
128.07
126.00
125.61
125.43
124.86
124.82
124.79
122.73
120.65
120.62
120.58
120.54
120.03
118.89
134 132 130 128 126 124 122 120 118
f1 (ppm)
210 200 190 180 170 160 150 140 130 120 110 100 90 80 70 60 50 40 30 20 10 0
f1 (ppm)

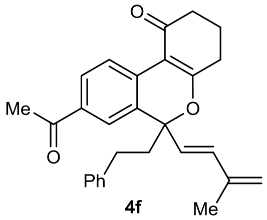
O
O
Me
O
Ph
Me

**4f**

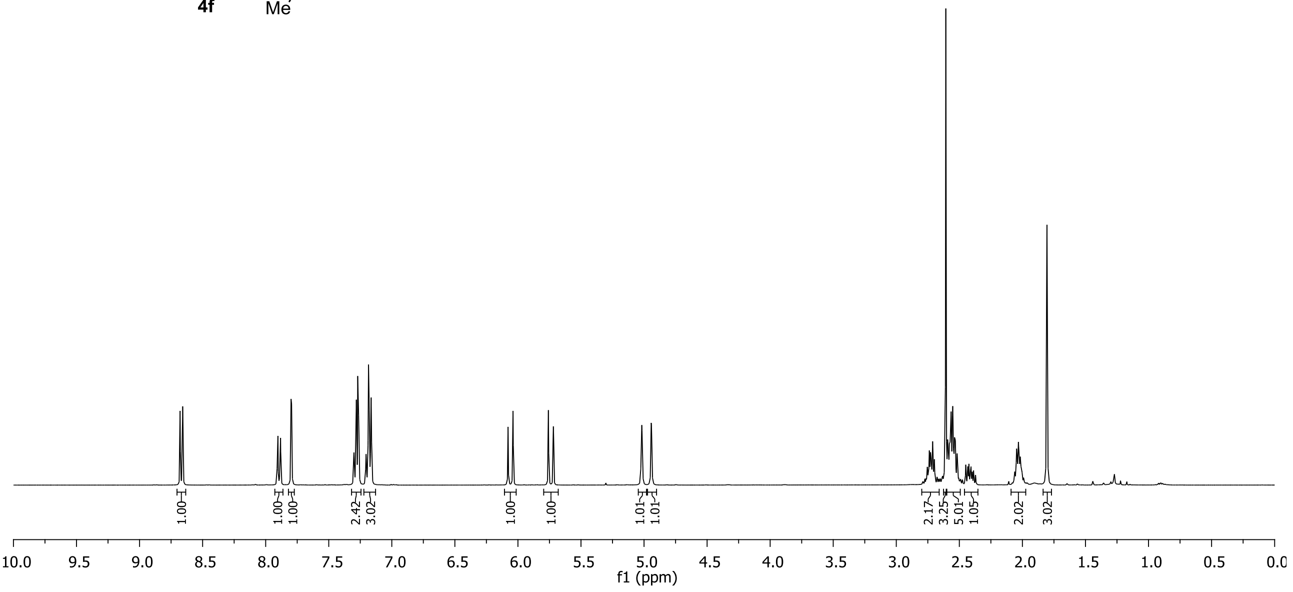
1.00
1.00
1.00
2.42
3.02
1.00
1.00
1.01
1.01
2.17
3.25
5.01
1.05
2.02
3.02
10.0 9.5 9.0 8.5 8.0 7.5 7.0 6.5 6.0 5.5 5.0 4.5 4.0 3.5 3.0 2.5 2.0 1.5 1.0 0.5 0.0
f1 (ppm)

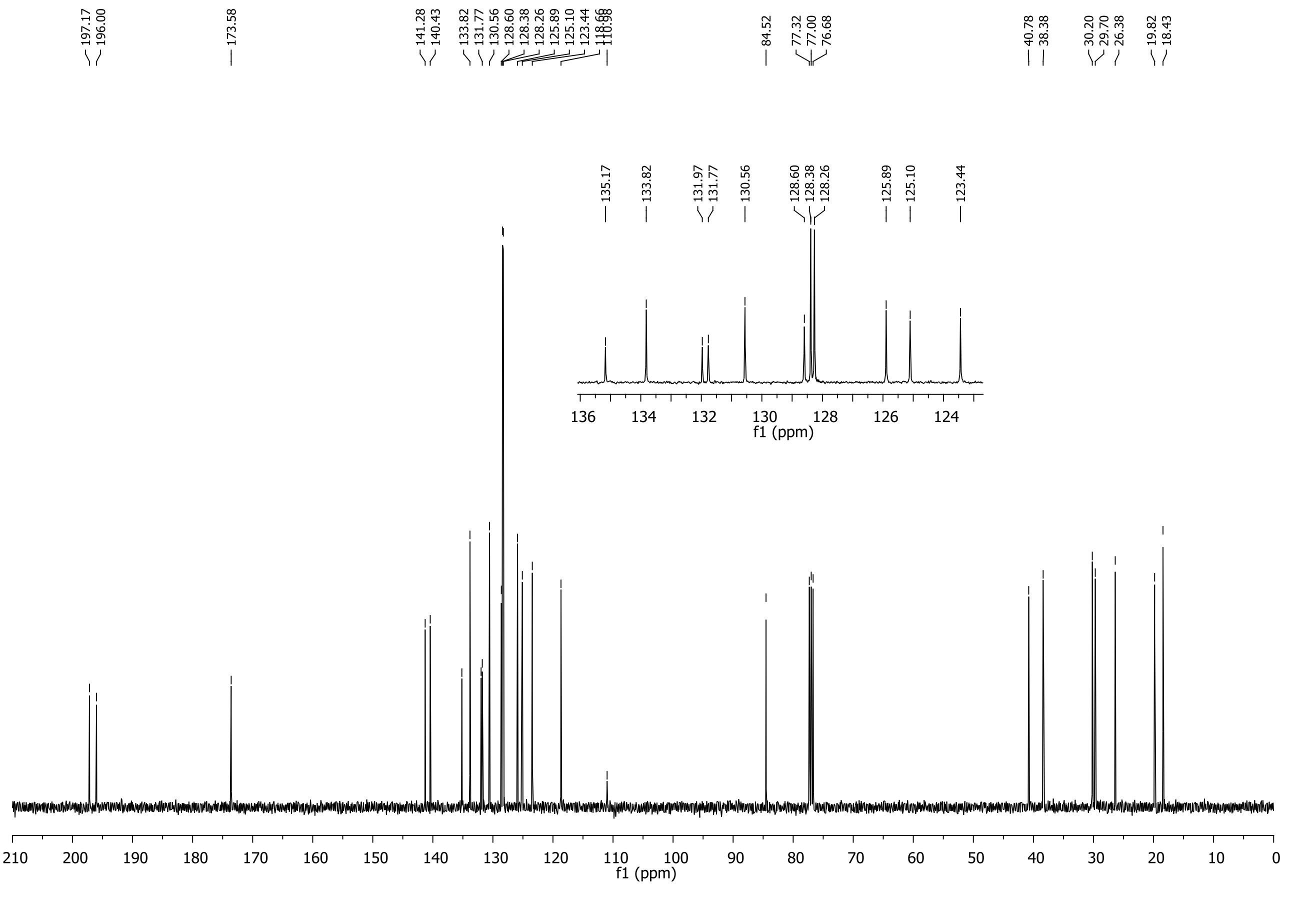
197.17
196.00
173.58
141.28
140.43
133.82
131.77
130.56
128.60
128.38
128.26
125.89
125.10
123.44
84.52
77.32
77.00
76.68
40.78
38.38
30.20
29.70
26.38
19.82
18.43
135.17
133.82
131.97
131.77
130.56
128.60
128.38
128.26
125.89
125.10
123.44
136 134 132 130 128 126 124
f1 (ppm)
210 200 190 180 170 160 150 140 130 120 110 100 90 80 70 60 50 40 30 20 10 0
f1 (ppm)

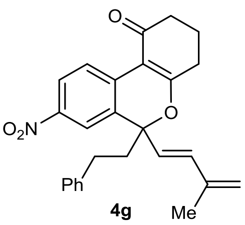
O
O
$O_2N$
Ph
Me

**4g**

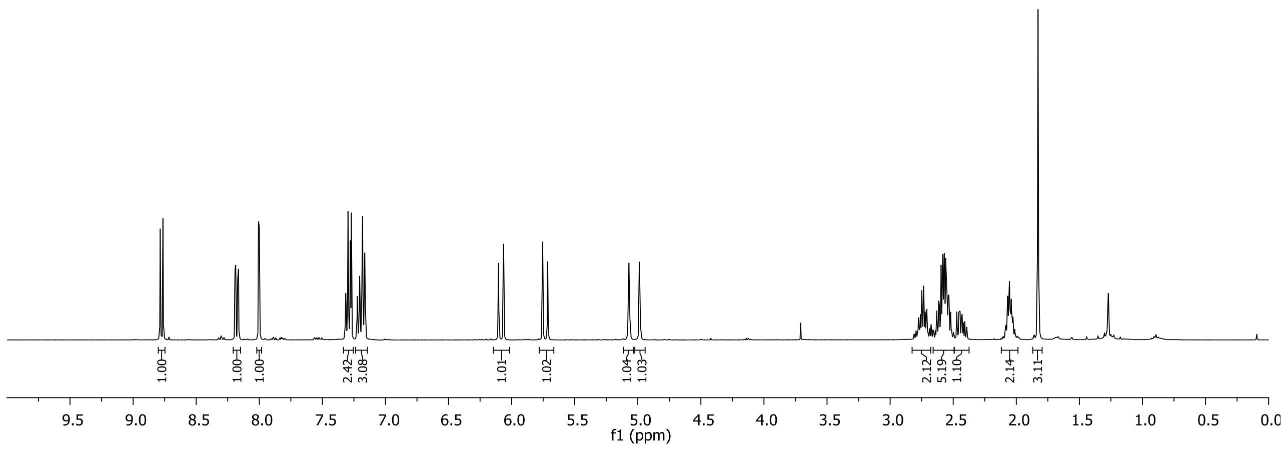
1.00
1.00
1.00
2.42
3.08
1.01
1.02
1.04
1.03
2.12
5.19
1.10
2.14
3.11
9.5 9.0 8.5 8.0 7.5 7.0 6.5 6.0 5.5 5.0 4.5 4.0 3.5 3.0 2.5 2.0 1.5 1.0 0.5 0.0
f1 (ppm)

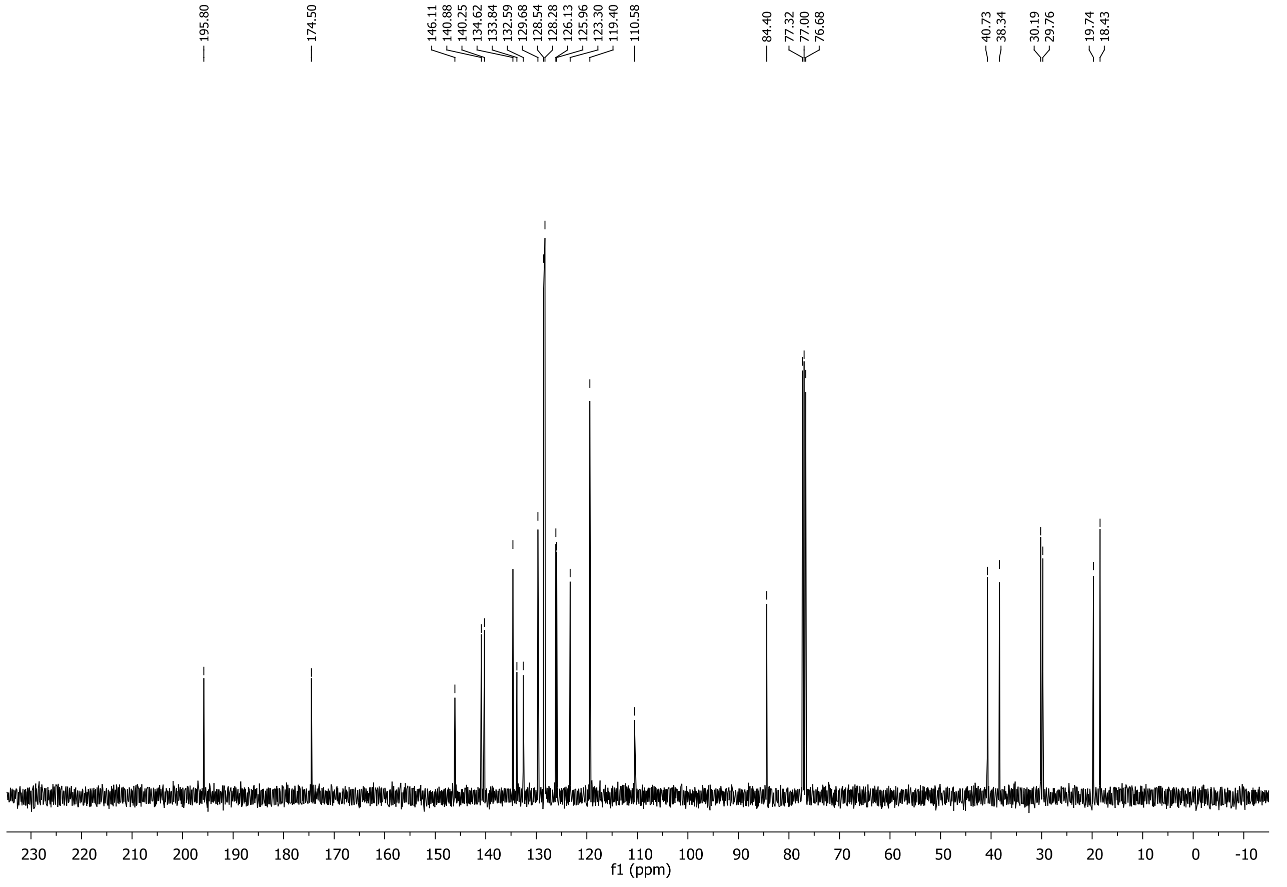
195.80
174.50
146.11
140.88
140.25
134.62
133.84
132.59
129.68
128.54
128.28
126.13
125.96
123.30
119.40
110.58
84.40
77.32
77.00
76.68
40.73
38.34
30.19
29.76
19.74
18.43
230 220 210 200 190 180 170 160 150 140 130 120 110 100 90 80 70 60 50 40 30 20 10 0 -10
f1 (ppm)

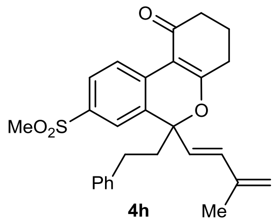
O
O
MeO$_2$S
Ph
Me

**4h**

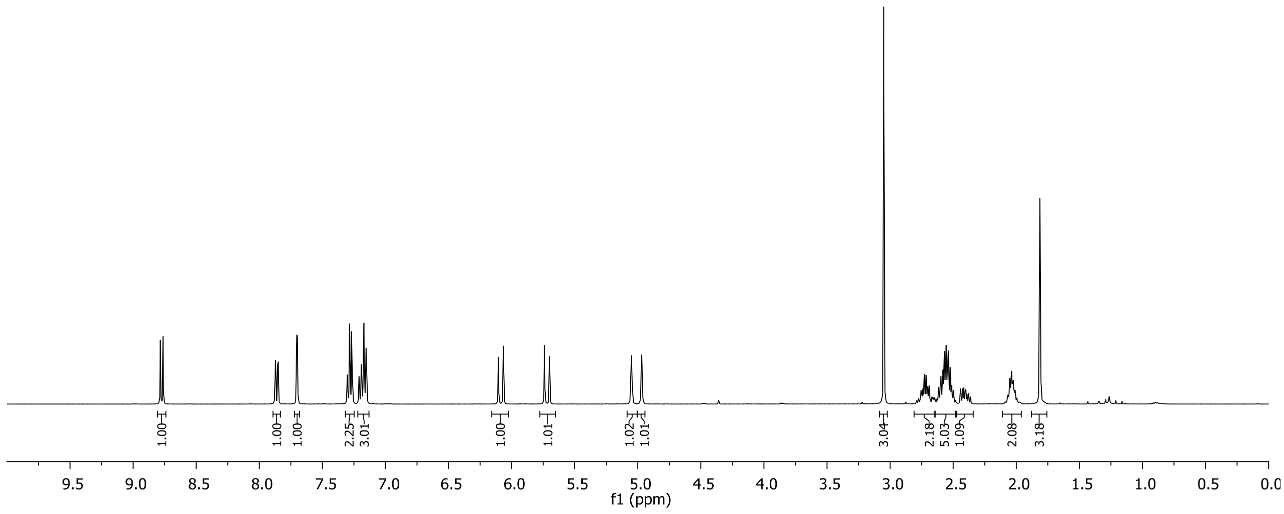
1.00
1.00
1.00
2.25
3.01
1.00
1.01
1.02
1.01
3.04
2.18
5.03
1.09
2.08
3.18
9.5 9.0 8.5 8.0 7.5 7.0 6.5 6.0 5.5 5.0 4.5 4.0 3.5 3.0 2.5 2.0 1.5 1.0 0.5 0.0
f1 (ppm)

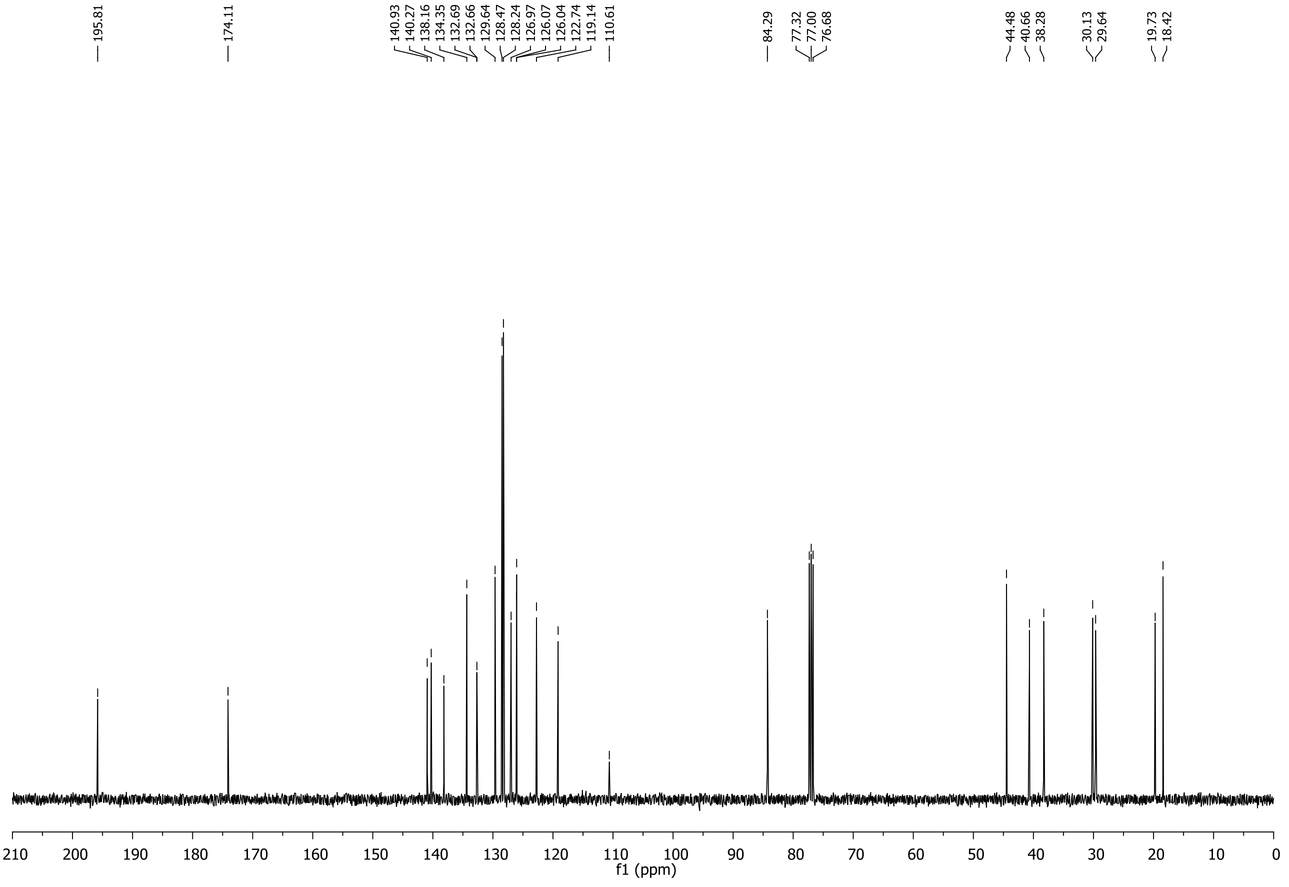
195.81
174.11
140.93
140.27
138.16
134.35
132.69
132.66
129.64
128.47
128.24
126.97
126.07
126.04
122.74
119.14
110.61
84.29
77.32
77.00
76.68
44.48
40.66
38.28
30.13
29.64
19.73
18.42
210 200 190 180 170 160 150 140 130 120 110 100 90 80 70 60 50 40 30 20 10 0
f1 (ppm)

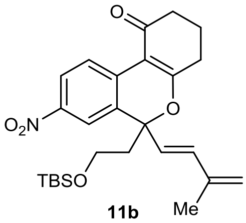
O
O
$O_2N$
TBSO
Me

**11b**

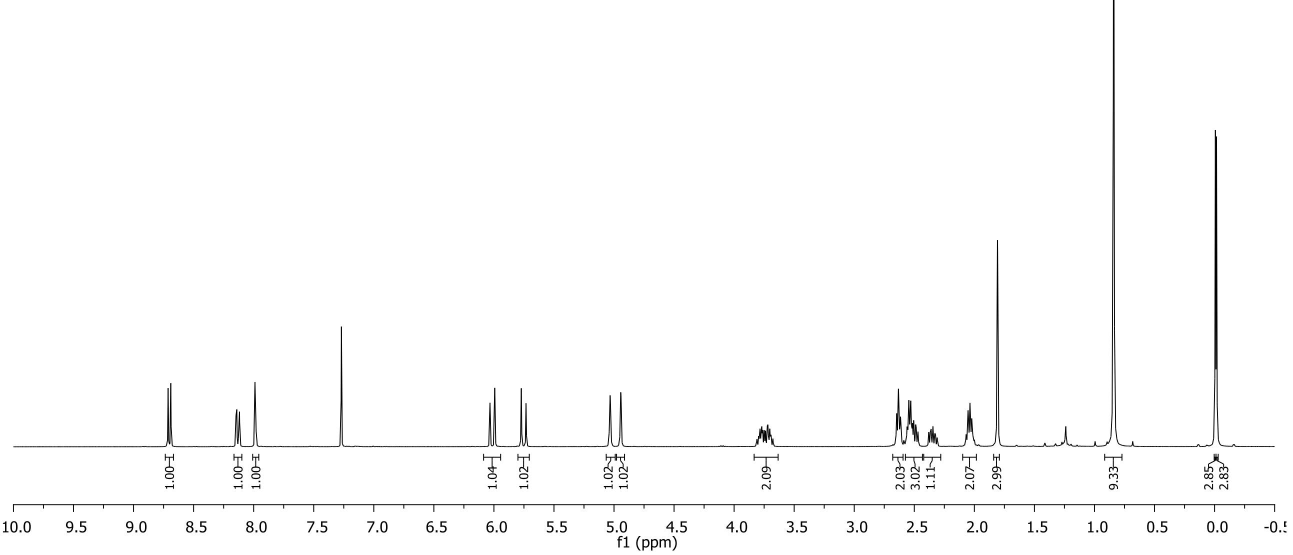
1.00
1.00
1.00
1.04
1.02
1.02
1.02
2.09
2.03
3.02
1.11
2.07
2.99
9.33
2.85
2.83
10.0 9.5 9.0 8.5 8.0 7.5 7.0 6.5 6.0 5.5 5.0 4.5 4.0 3.5 3.0 2.5 2.0 1.5 1.0 0.5 0.0
f1 (ppm)

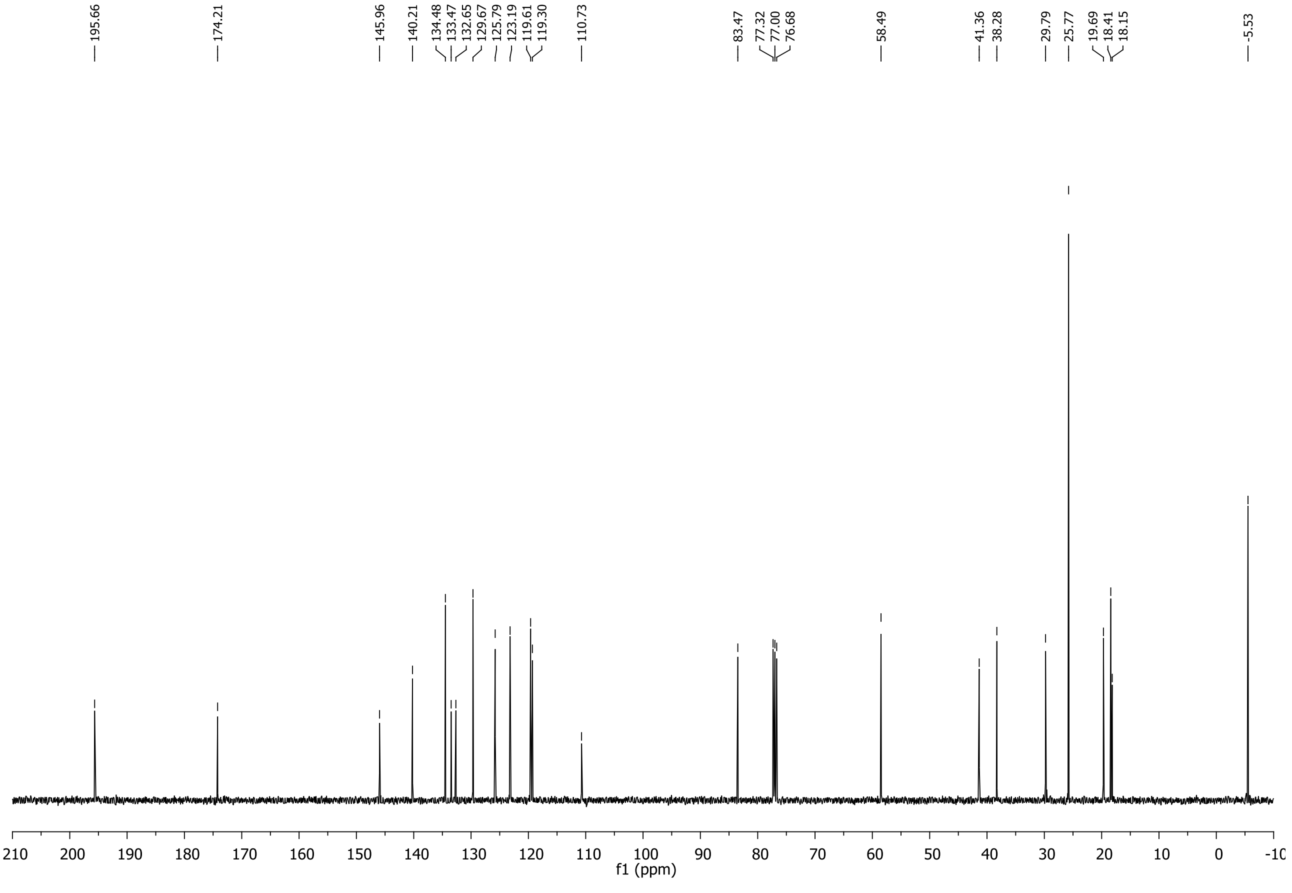
195.66
174.21
145.96
140.21
134.48
133.47
132.65
129.67
125.79
123.19
119.61
119.30
110.73
83.47
77.32
77.00
76.68
58.49
41.36
38.28
29.79
25.77
19.69
18.41
18.15
-5.53
210 200 190 180 170 160 150 140 130 120 110 100 90 80 70 60 50 40 30 20 10 0
f1 (ppm)

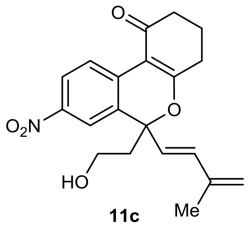
O
O
$O_2N$
HO
Me

**11c**

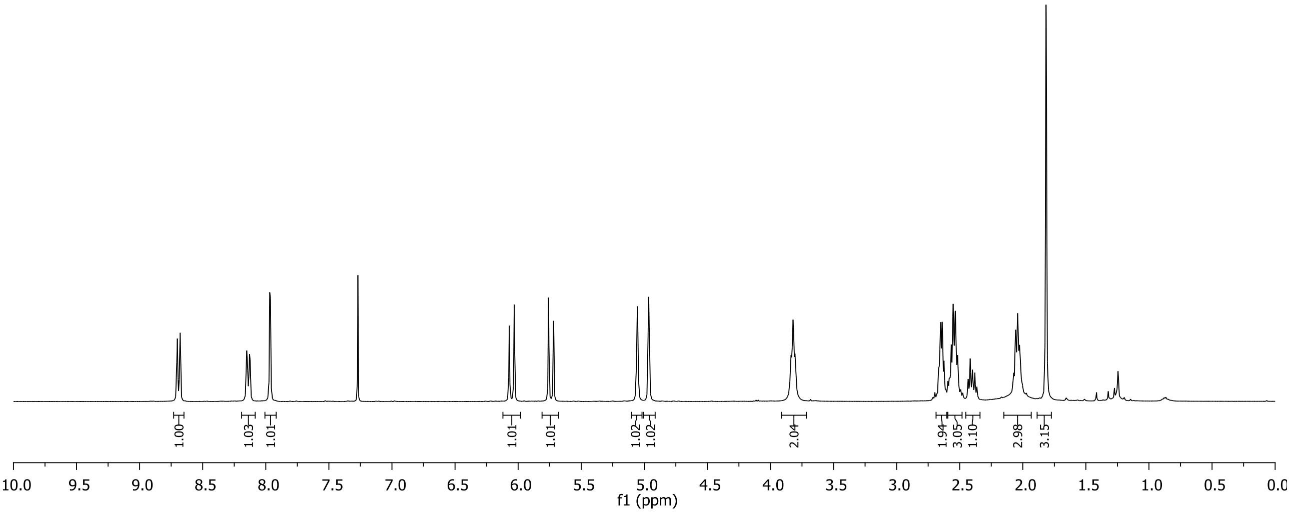
1.00
1.03
1.01
1.01
1.01
1.02
1.02
2.04
1.94
3.05
1.10
2.98
3.15
10.0 9.5 9.0 8.5 8.0 7.5 7.0 6.5 6.0 5.5 5.0 4.5 4.0 3.5 3.0 2.5 2.0 1.5 1.0 0.5 0.0
f1 (ppm)

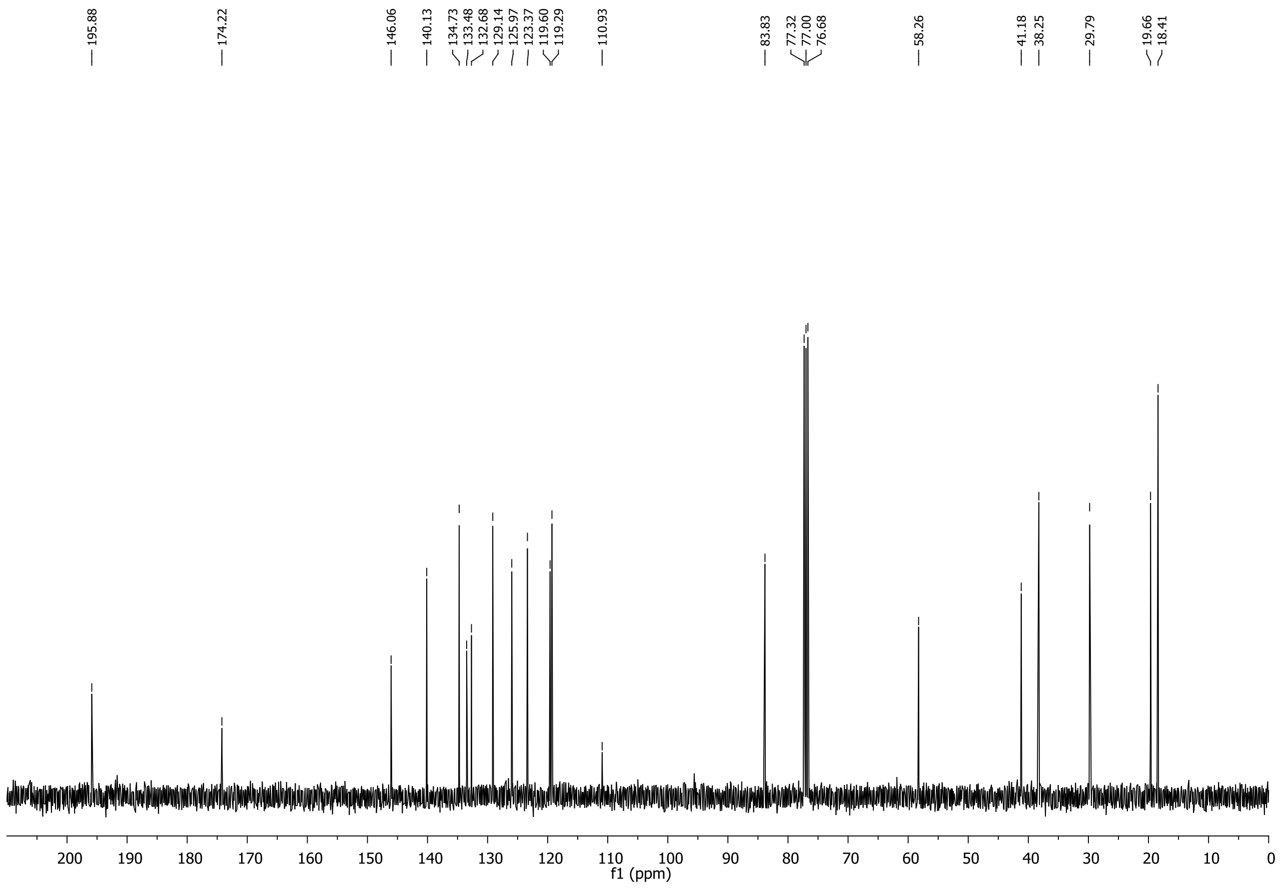
195.88
174.22
146.06
140.13
134.73
133.48
132.68
129.14
125.97
123.37
119.60
119.29
110.93
83.83
77.32
77.00
76.68
58.26
41.18
38.25
29.79
19.66
18.41
200 190 180 170 160 150 140 130 120 110 100 90 80 70 60 50 40 30 20 10 0
f1 (ppm)

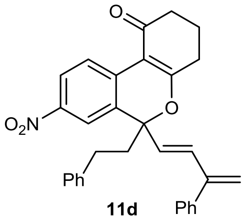
O
O
O2N
Ph
Ph

**11d**

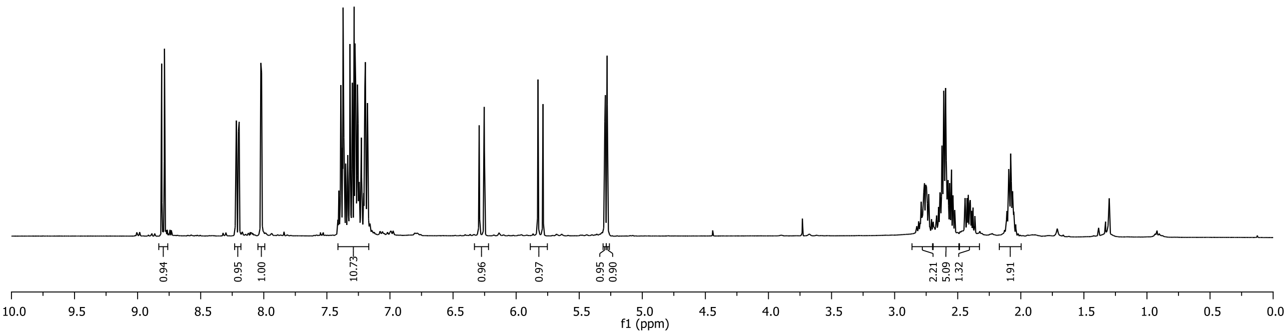
0.94
0.95
1.00
10.73
0.96
0.97
0.95
0.90
2.21
5.09
1.32
1.91
10.0 9.5 9.0 8.5 8.0 7.5 7.0 6.5 6.0 5.5 5.0 4.5 4.0 3.5 3.0 2.5 2.0 1.5 1.0 0.5 0.0
f1 (ppm)

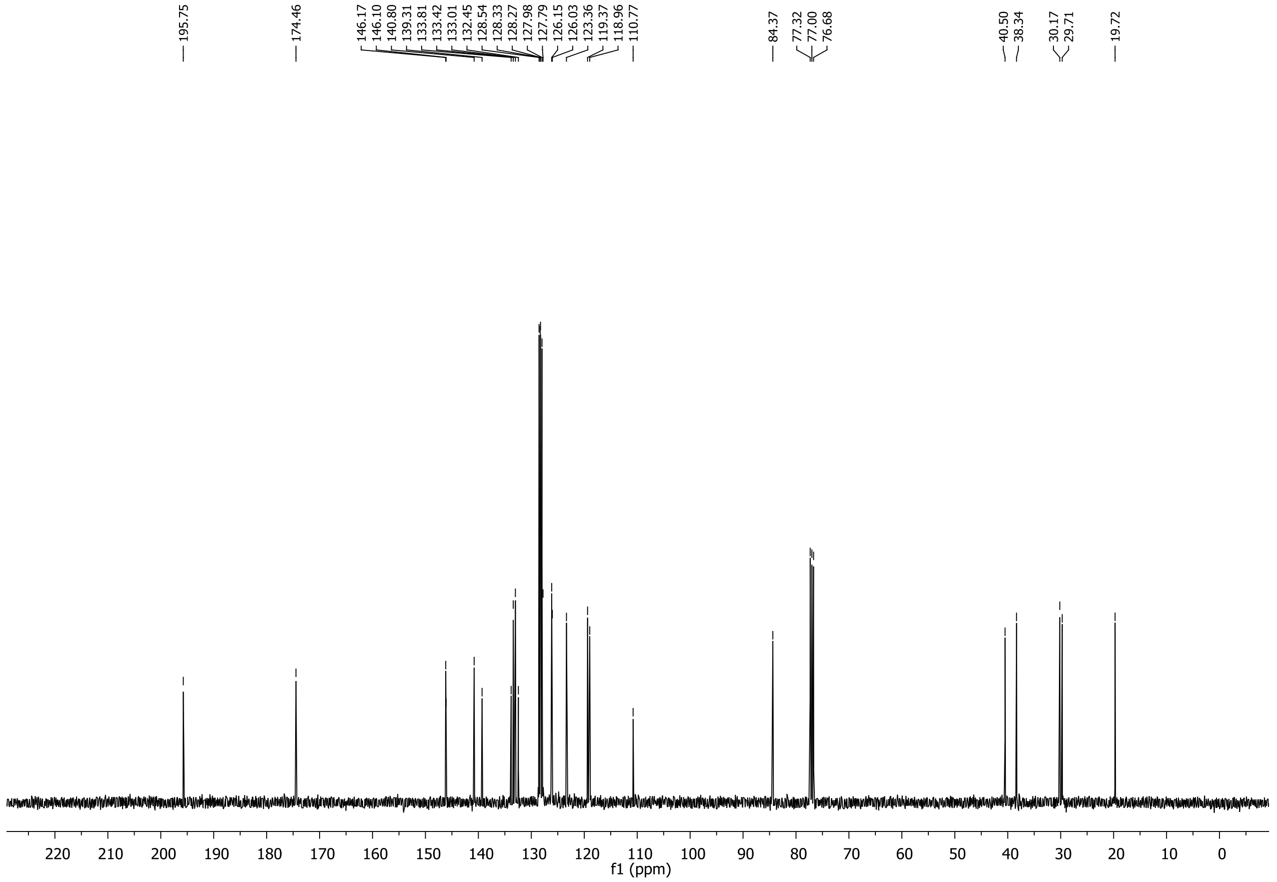
195.75
174.46
146.17
146.10
140.80
139.31
133.81
133.42
133.01
132.45
128.54
128.33
128.27
127.98
127.79
126.15
126.03
123.36
119.37
118.96
110.77
84.37
77.32
77.00
76.68
40.50
38.34
30.17
29.71
19.72
220 210 200 190 180 170 160 150 140 130 120 110 100 90 80 70 60 50 40 30 20 10 0
f1 (ppm)

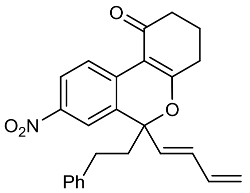
O
O
$O_2N$
Ph

**11e**

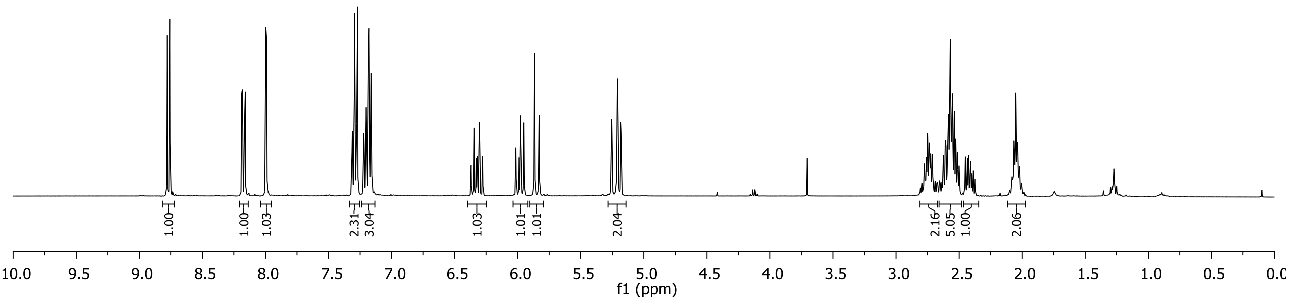
1.00
1.00
1.03
2.31
3.04
1.03
1.01
1.01
2.04
2.16
5.05
1.00
2.06
f1 (ppm)

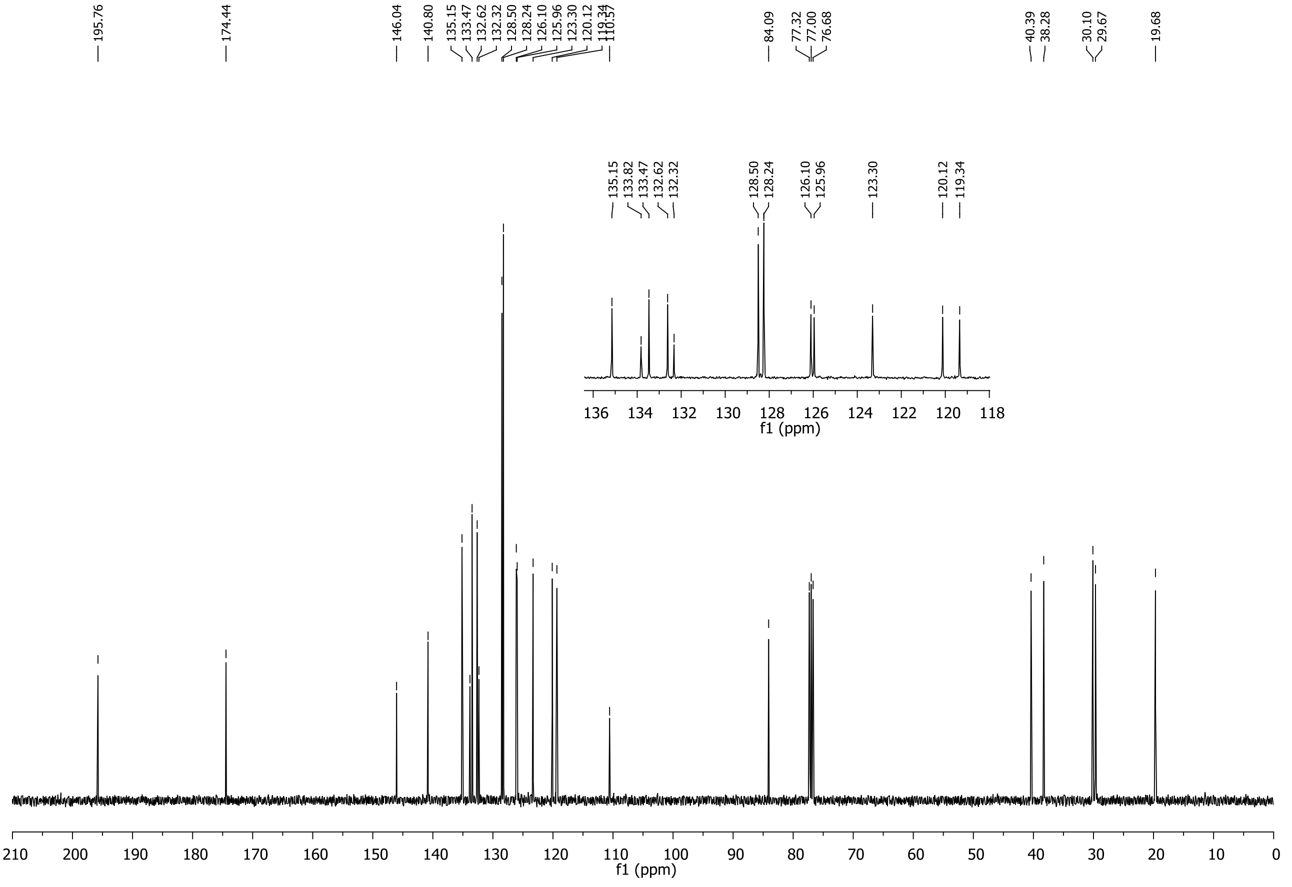
195.76
174.44
146.04
140.80
135.15
133.47
132.62
132.32
128.50
128.24
126.10
125.96
123.30
120.12
119.34
110.57
84.09
77.32
77.00
76.68
40.39
38.28
30.10
29.67
19.68
135.15
133.82
133.47
132.62
132.32
128.50
128.24
126.10
125.96
123.30
120.12
119.34
f1 (ppm)
f1 (ppm)

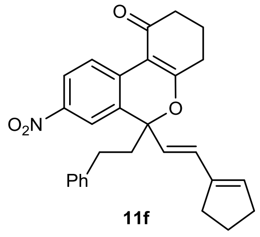
O
O
$O_2N$
Ph

**11f**

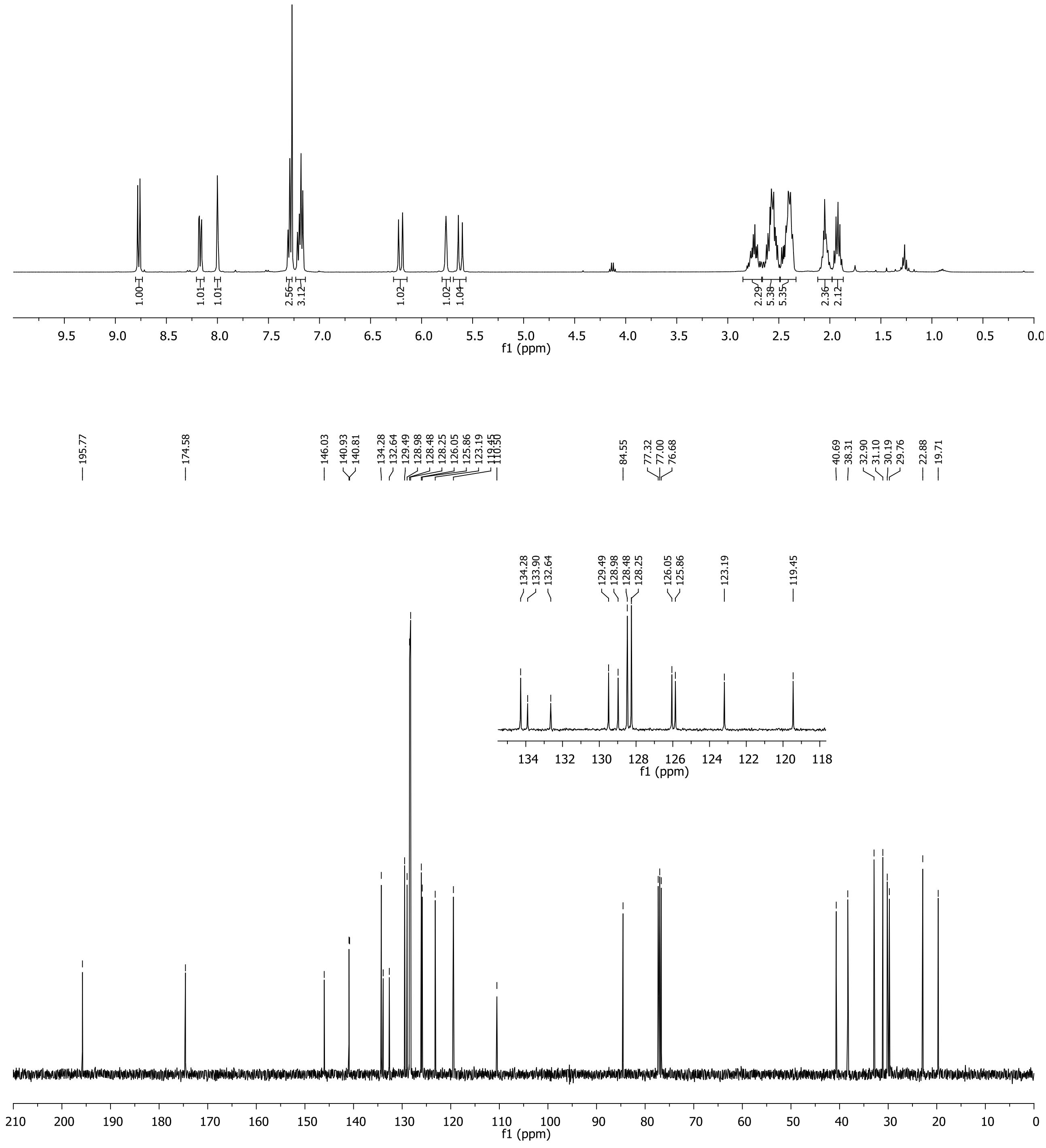
1.00
1.01
1.01
2.56
3.12
1.02
1.02
1.04
2.29
5.38
5.35
2.36
2.12
9.5 9.0 8.5 8.0 7.5 7.0 6.5 6.0 5.5 5.0 4.5 4.0 3.5 3.0 2.5 2.0 1.5 1.0 0.5 0.0
f1 (ppm)
195.77
174.58
146.03
140.93
140.81
134.28
132.64
129.49
128.98
128.48
128.25
126.05
125.86
123.19
119.45
110.50
84.55
77.32
77.00
76.68
40.69
38.31
32.90
31.10
30.19
29.76
22.88
19.71
134.28
133.90
132.64
129.49
128.98
128.48
128.25
126.05
125.86
123.19
119.45
134 132 130 128 126 124 122 120 118
f1 (ppm)
210 200 190 180 170 160 150 140 130 120 110 100 90 80 70 60 50 40 30 20 10 0
f1 (ppm)

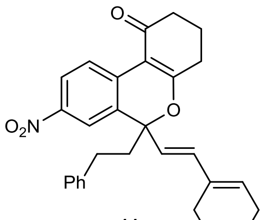
O
O
O$_2$N
Ph

**11g**

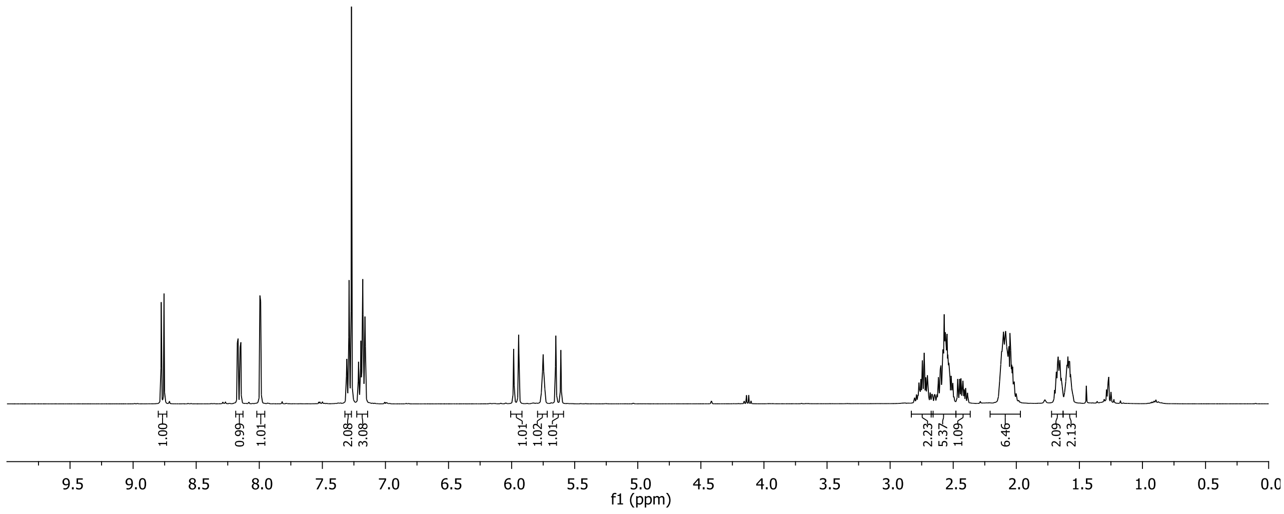
1.00
0.99
1.01
2.08
3.08
1.01
1.02
1.01
2.23
5.37
1.09
6.46
2.09
2.13
9.5 9.0 8.5 8.0 7.5 7.0 6.5 6.0 5.5 5.0 4.5 4.0 3.5 3.0 2.5 2.0 1.5 1.0 0.5 0.0
f1 (ppm)

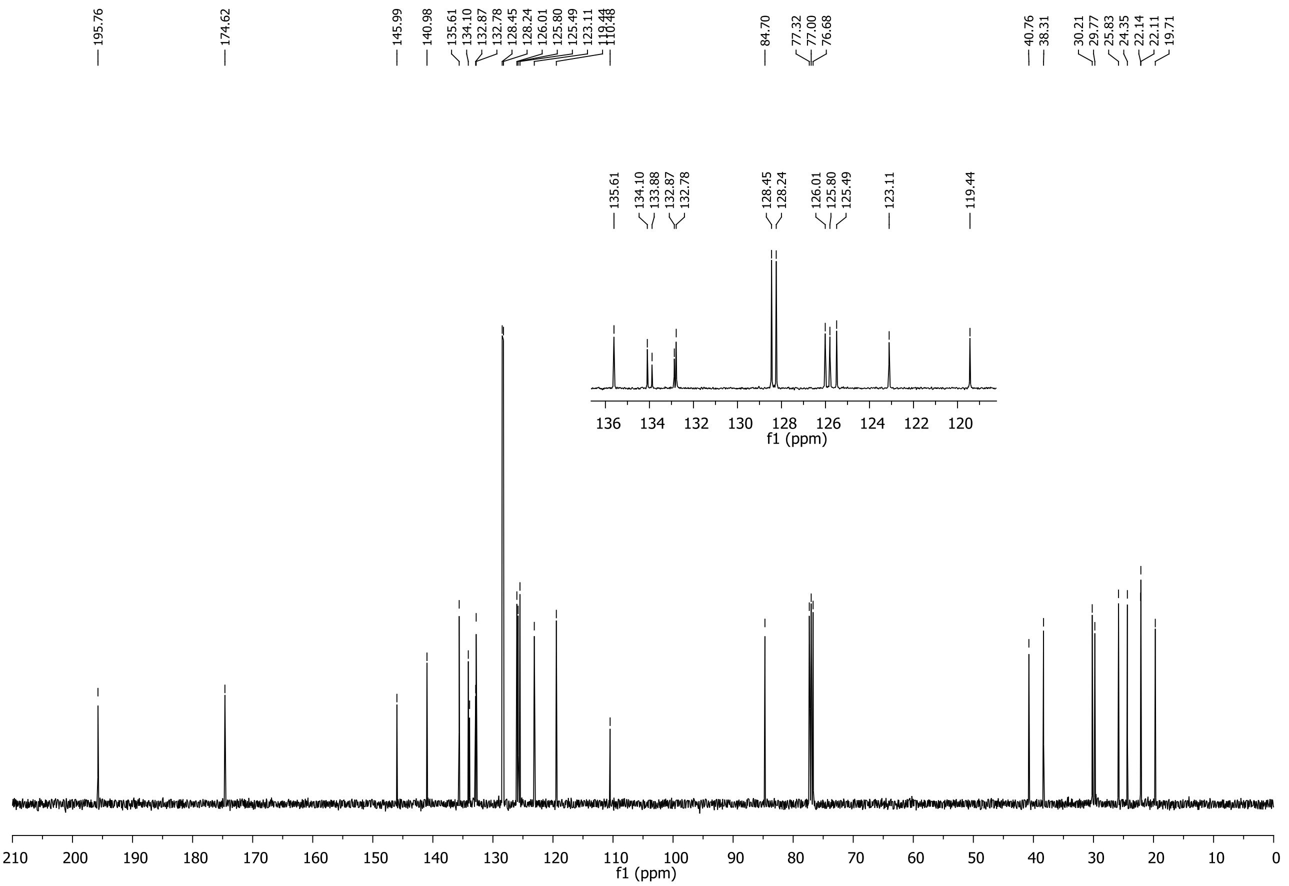
195.76
174.62
145.99
140.98
135.61
134.10
132.87
132.78
128.45
128.24
126.01
125.80
125.49
123.11
119.44
110.48
84.70
77.32
77.00
76.68
40.76
38.31
30.21
29.77
25.83
24.35
22.14
22.11
19.71
135.61
134.10
133.88
132.87
132.78
128.45
128.24
126.01
125.80
125.49
123.11
119.44
136 134 132 130 128 126 124 122 120
f1 (ppm)
210 200 190 180 170 160 150 140 130 120 110 100 90 80 70 60 50 40 30 20 10 0
f1 (ppm)

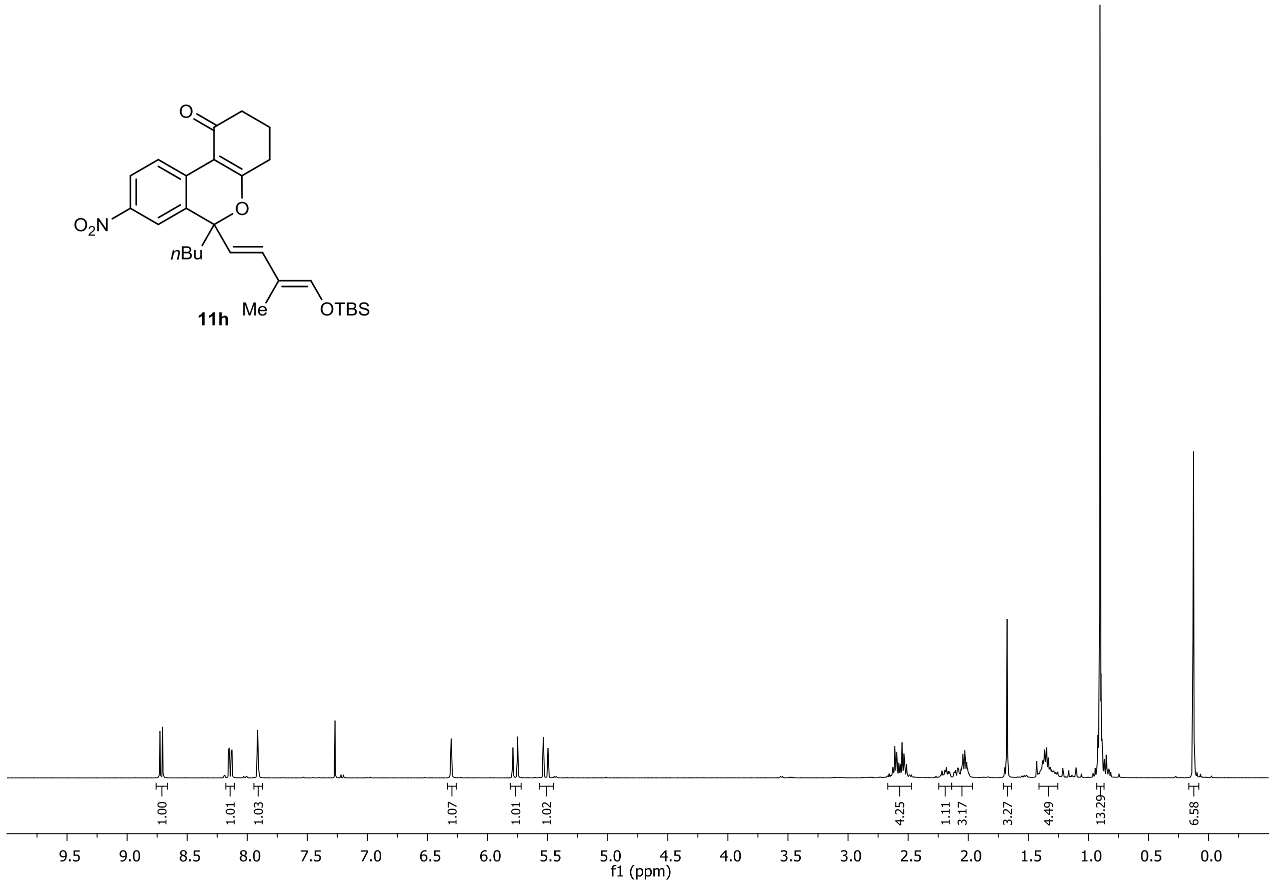
O
O
O2N
nBu
Me
OTBS
11h
1.00
1.01
1.03
1.07
1.01
1.02
4.25
1.11
3.17
3.27
4.49
13.29
6.58
9.5 9.0 8.5 8.0 7.5 7.0 6.5 6.0 5.5 5.0 4.5 4.0 3.5 3.0 2.5 2.0 1.5 1.0 0.5 0.0
f1 (ppm)

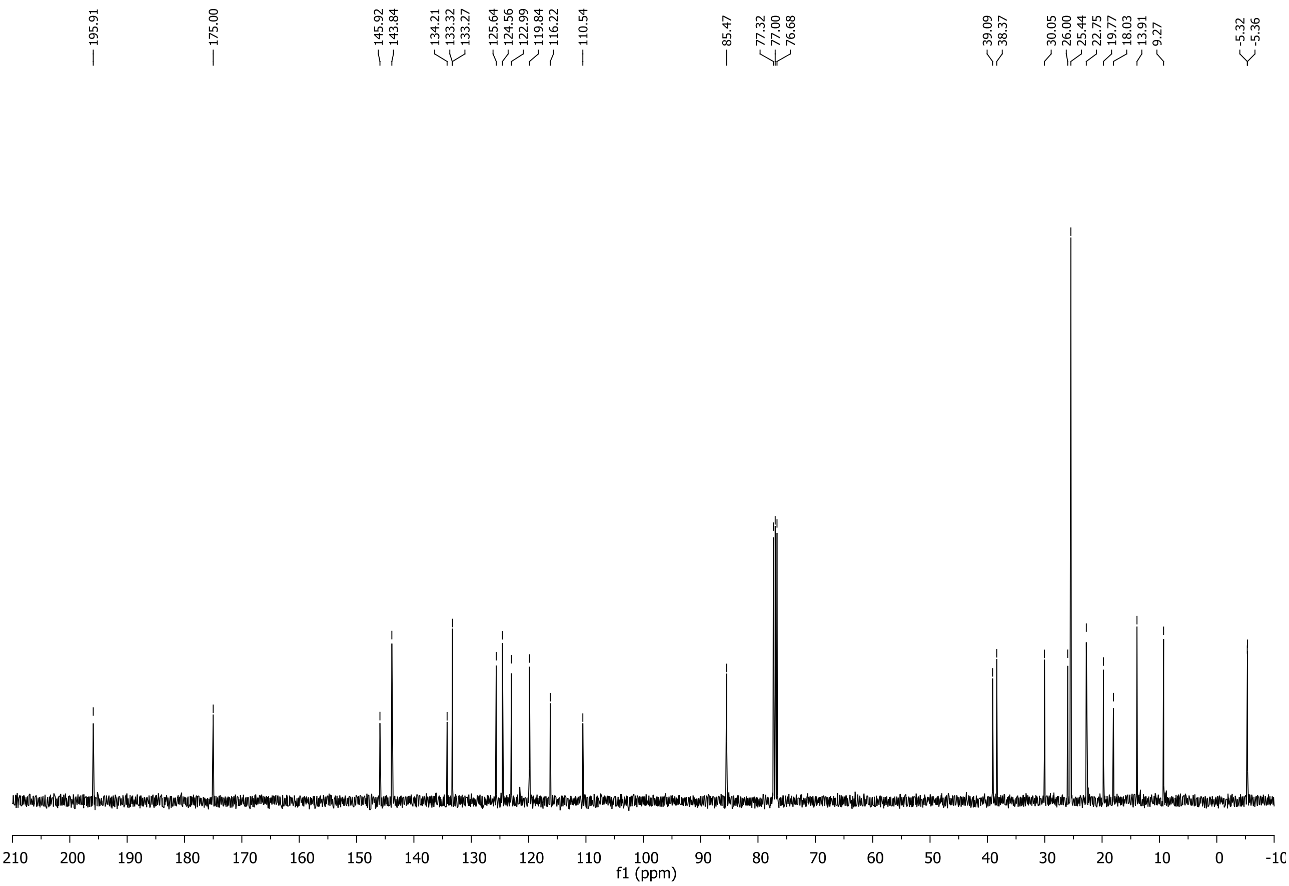
195.91
175.00
145.92
143.84
134.21
133.32
133.27
125.64
124.56
122.99
119.84
116.22
110.54
85.47
77.32
77.00
76.68
39.09
38.37
30.05
26.00
25.44
22.75
19.77
18.03
13.91
9.27
-5.32
-5.36
210 200 190 180 170 160 150 140 130 120 110 100 90 80 70 60 50 40 30 20 10 0 -10
f1 (ppm)

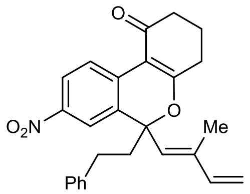
O
O
O2N
Me
Ph

**11i**

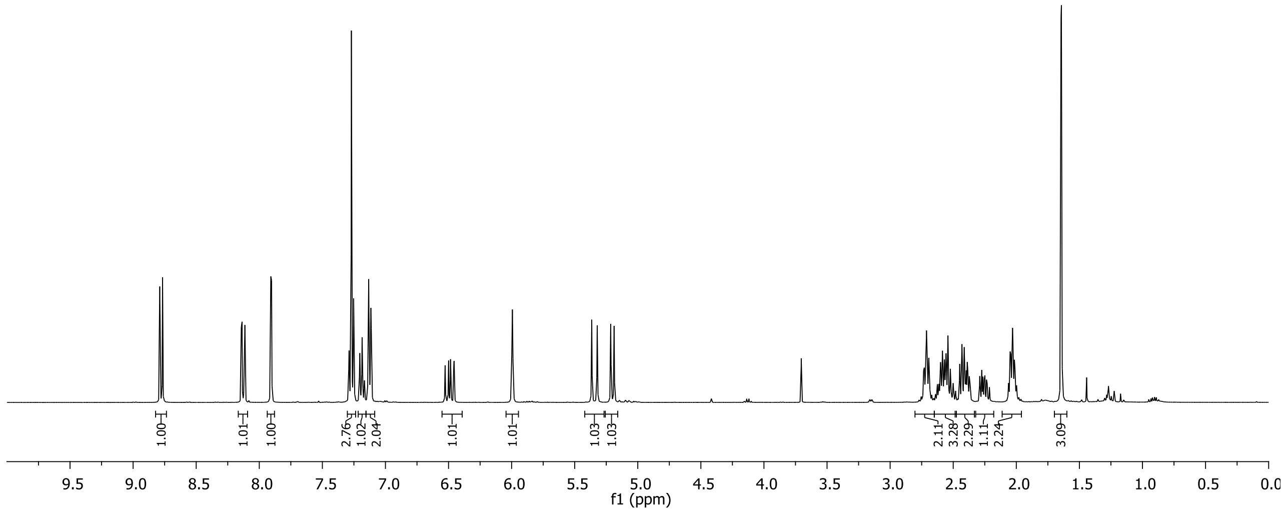
1.00
1.01
1.00
2.76
1.02
2.04
1.01
1.01
1.03
1.03
2.11
3.28
2.29
1.11
2.24
3.09
9.5 9.0 8.5 8.0 7.5 7.0 6.5 6.0 5.5 5.0 4.5 4.0 3.5 3.0 2.5 2.0 1.5 1.0 0.5 0.0
f1 (ppm)

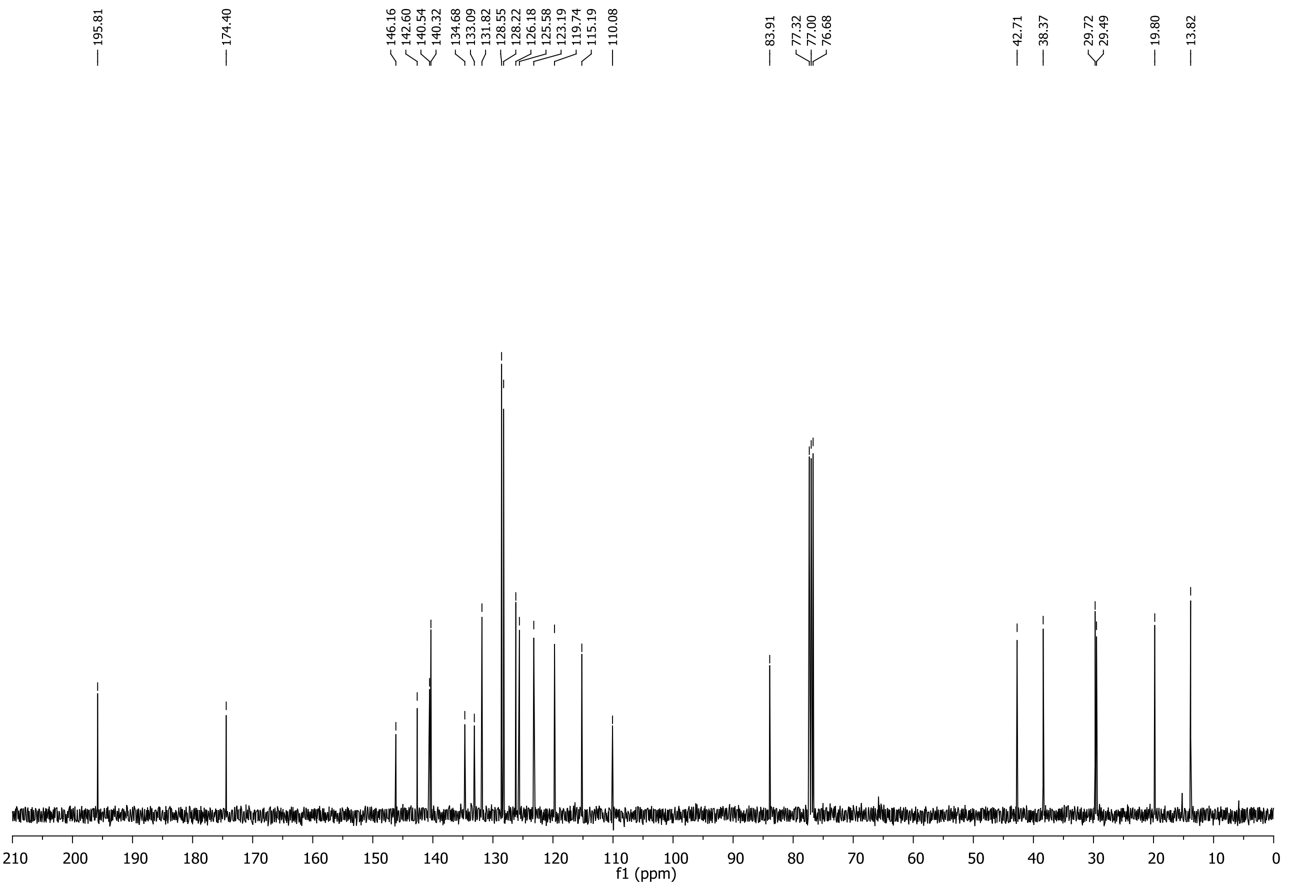
195.81
174.40
146.16
142.60
140.54
140.32
134.68
133.09
131.82
128.55
128.22
126.18
125.58
123.19
119.74
115.19
110.08
83.91
77.32
77.00
76.68
42.71
38.37
29.72
29.49
19.80
13.82
210 200 190 180 170 160 150 140 130 120 110 100 90 80 70 60 50 40 30 20 10 0
f1 (ppm)

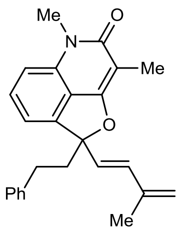
Me
N
O
Me
O
Ph
Me

**13a**

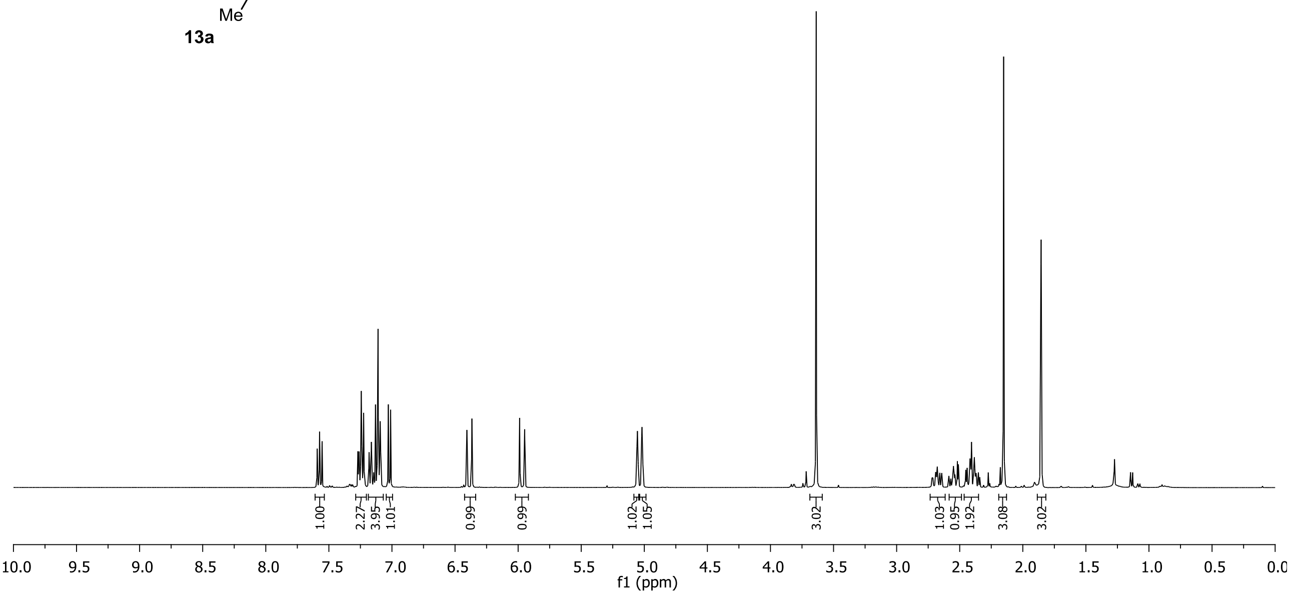
1.00
2.27
3.95
1.01
0.99
0.99
1.02
1.05
3.02
1.03
0.95
1.92
3.08
3.02
10.0 9.5 9.0 8.5 8.0 7.5 7.0 6.5 6.0 5.5 5.0 4.5 4.0 3.5 3.0 2.5 2.0 1.5 1.0 0.5 0.0
f1 (ppm)

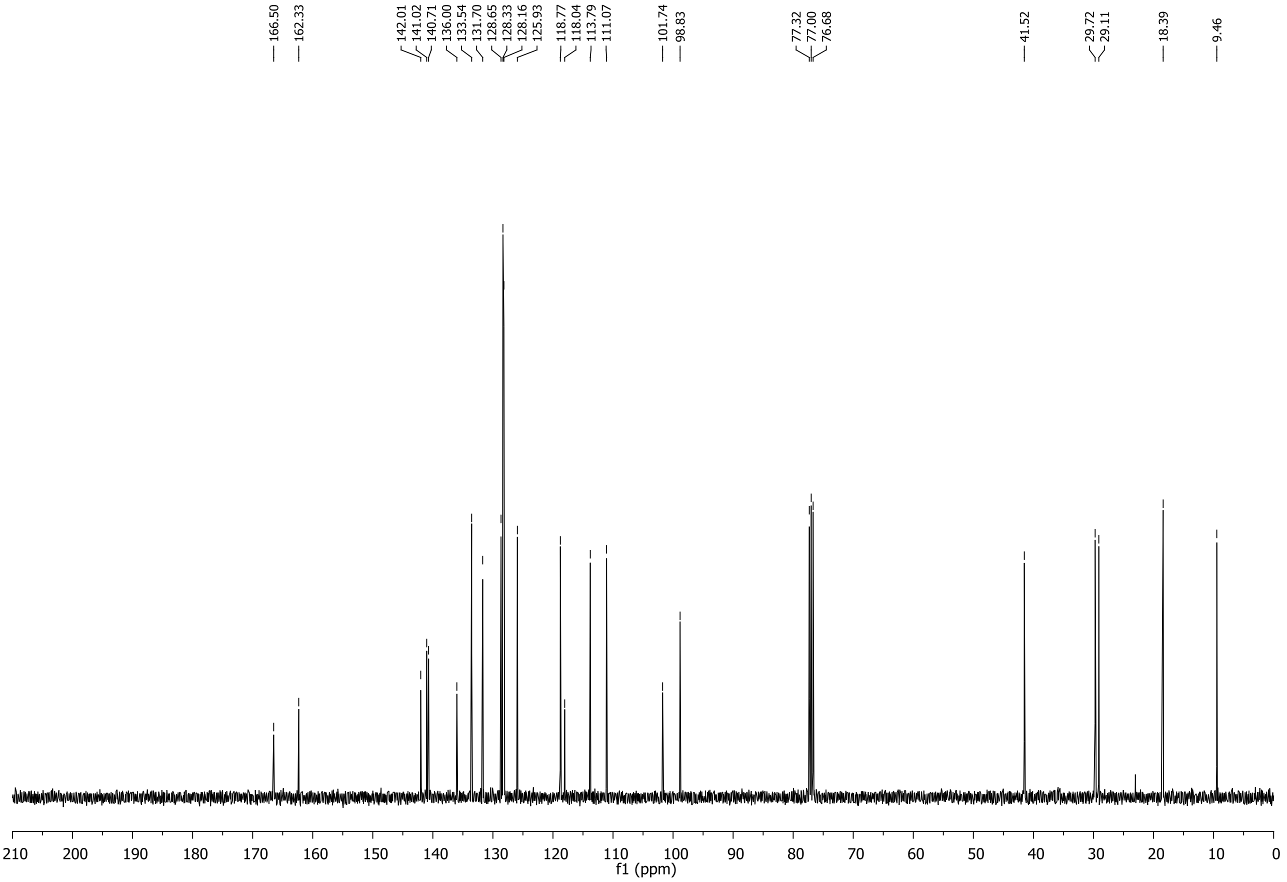
166.50
162.33
142.01
141.02
140.71
136.00
133.54
131.70
128.65
128.33
128.16
125.93
118.77
118.04
113.79
111.07
101.74
98.83
77.32
77.00
76.68
41.52
29.72
29.11
18.39
9.46
210 200 190 180 170 160 150 140 130 120 110 100 90 80 70 60 50 40 30 20 10 0
f1 (ppm)

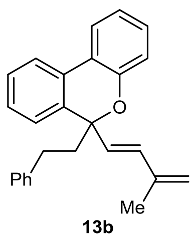
O
Ph
Me

**13b**

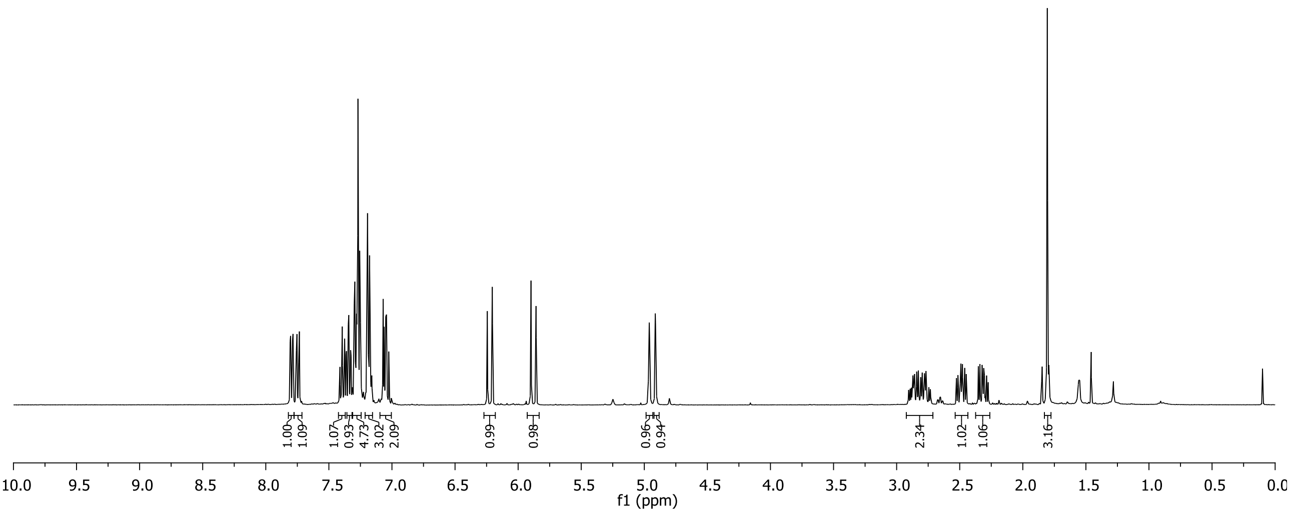
1.00
1.09
1.07
0.93
4.73
3.02
2.09
0.99
0.98
0.96
0.94
2.34
1.02
1.06
3.16
10.0 9.5 9.0 8.5 8.0 7.5 7.0 6.5 6.0 5.5 5.0 4.5 4.0 3.5 3.0 2.5 2.0 1.5 1.0 0.5 0.0
f1 (ppm)

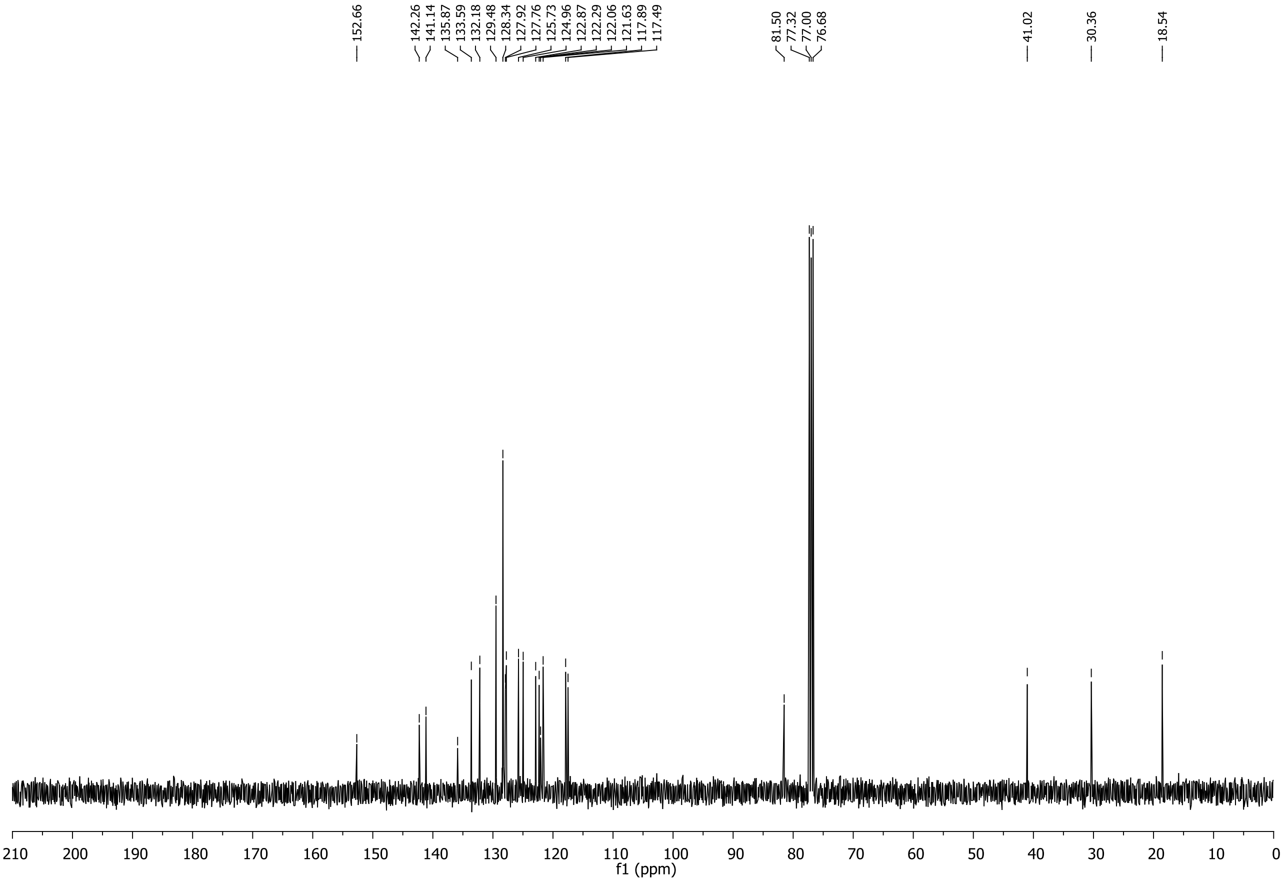
152.66
142.26
141.14
135.87
133.59
132.18
129.48
128.34
127.92
127.76
125.73
124.96
122.87
122.29
122.06
121.63
117.89
117.49
81.50
77.32
77.00
76.68
41.02
30.36
18.54
210 200 190 180 170 160 150 140 130 120 110 100 90 80 70 60 50 40 30 20 10 0
f1 (ppm)

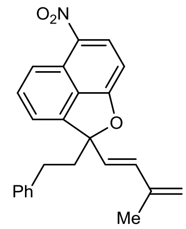
O2N
O
Ph
Me

**13c**

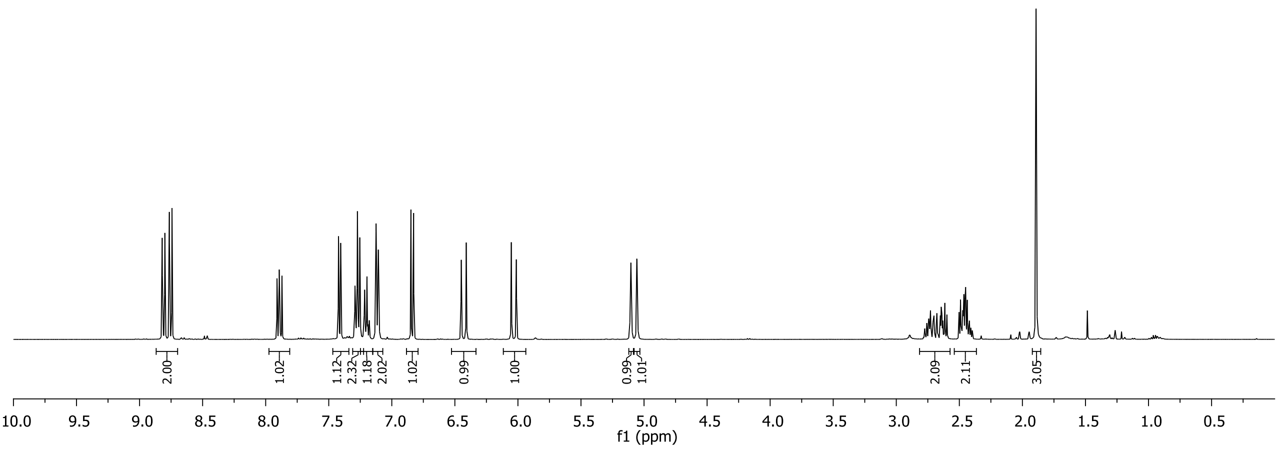
2.00
1.02
1.12
2.32
1.18
2.02
1.02
0.99
1.00
0.99
1.01
2.09
2.11
3.05
10.0 9.5 9.0 8.5 8.0 7.5 7.0 6.5 6.0 5.5 5.0 4.5 4.0 3.5 3.0 2.5 2.0 1.5 1.0 0.5
f1 (ppm)

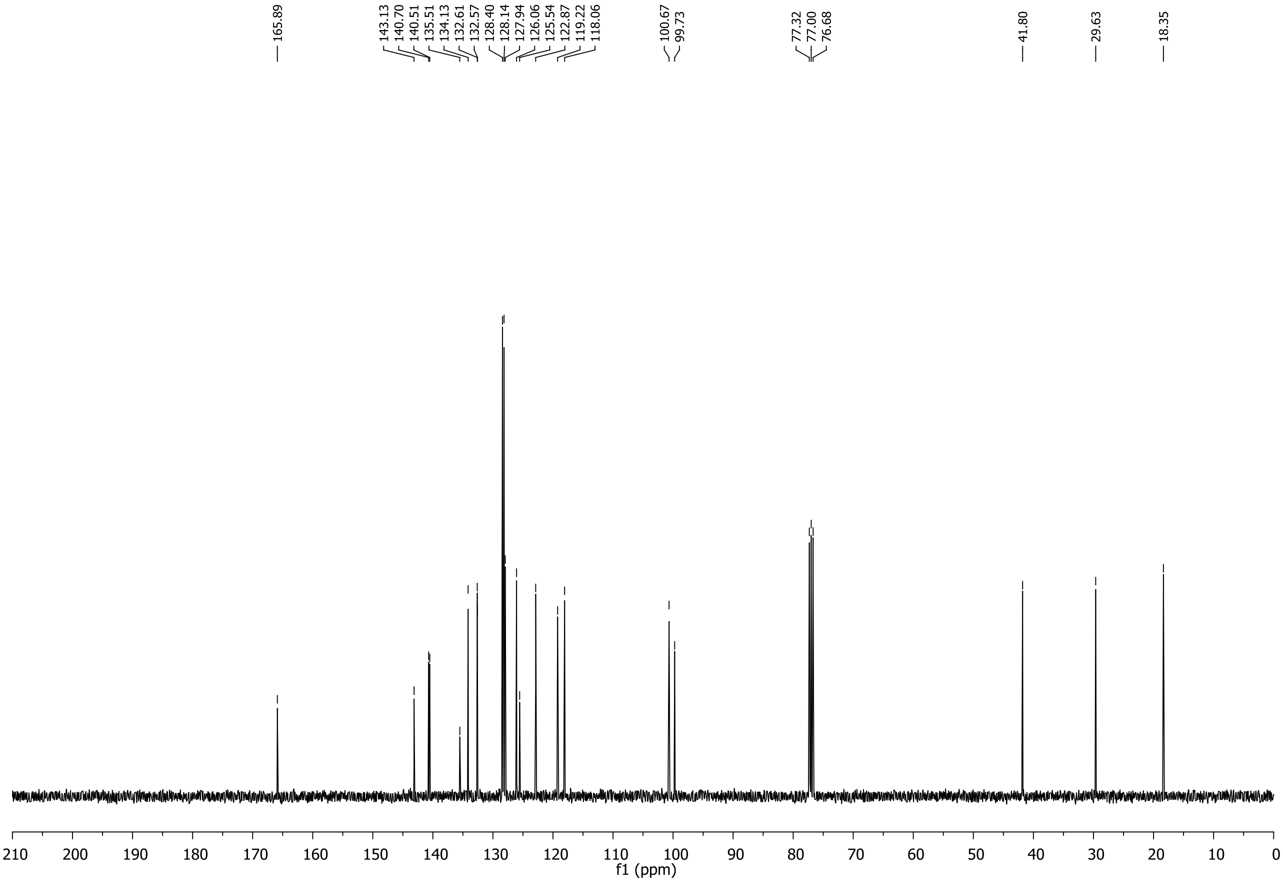
165.89
143.13
140.70
140.51
135.51
134.13
132.61
132.57
128.40
128.14
127.94
126.06
125.54
122.87
119.22
118.06
100.67
99.73
77.32
77.00
76.68
41.80
29.63
18.35
210 200 190 180 170 160 150 140 130 120 110 100 90 80 70 60 50 40 30 20 10 0
f1 (ppm)

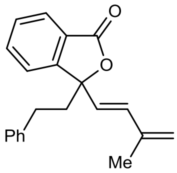
O
O
Ph
Me

**13d**

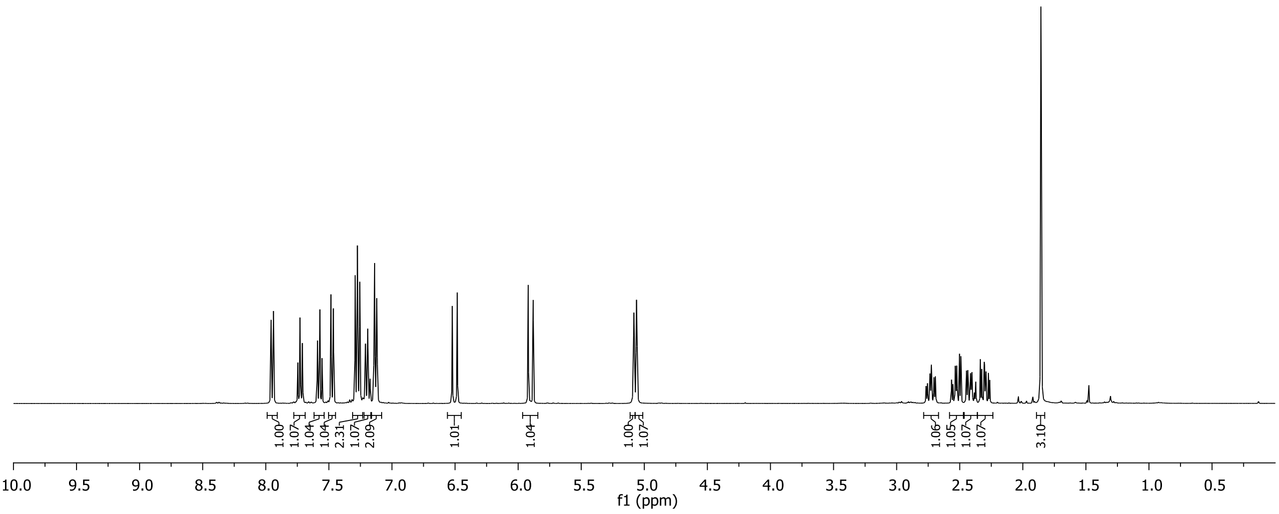
1.00
1.07
1.04
1.04
2.31
1.07
2.09
1.01
1.04
1.00
1.07
1.06
1.05
1.07
1.07
3.10
10.0 9.5 9.0 8.5 8.0 7.5 7.0 6.5 6.0 5.5 5.0 4.5 4.0 3.5 3.0 2.5 2.0 1.5 1.0 0.5
f1 (ppm)

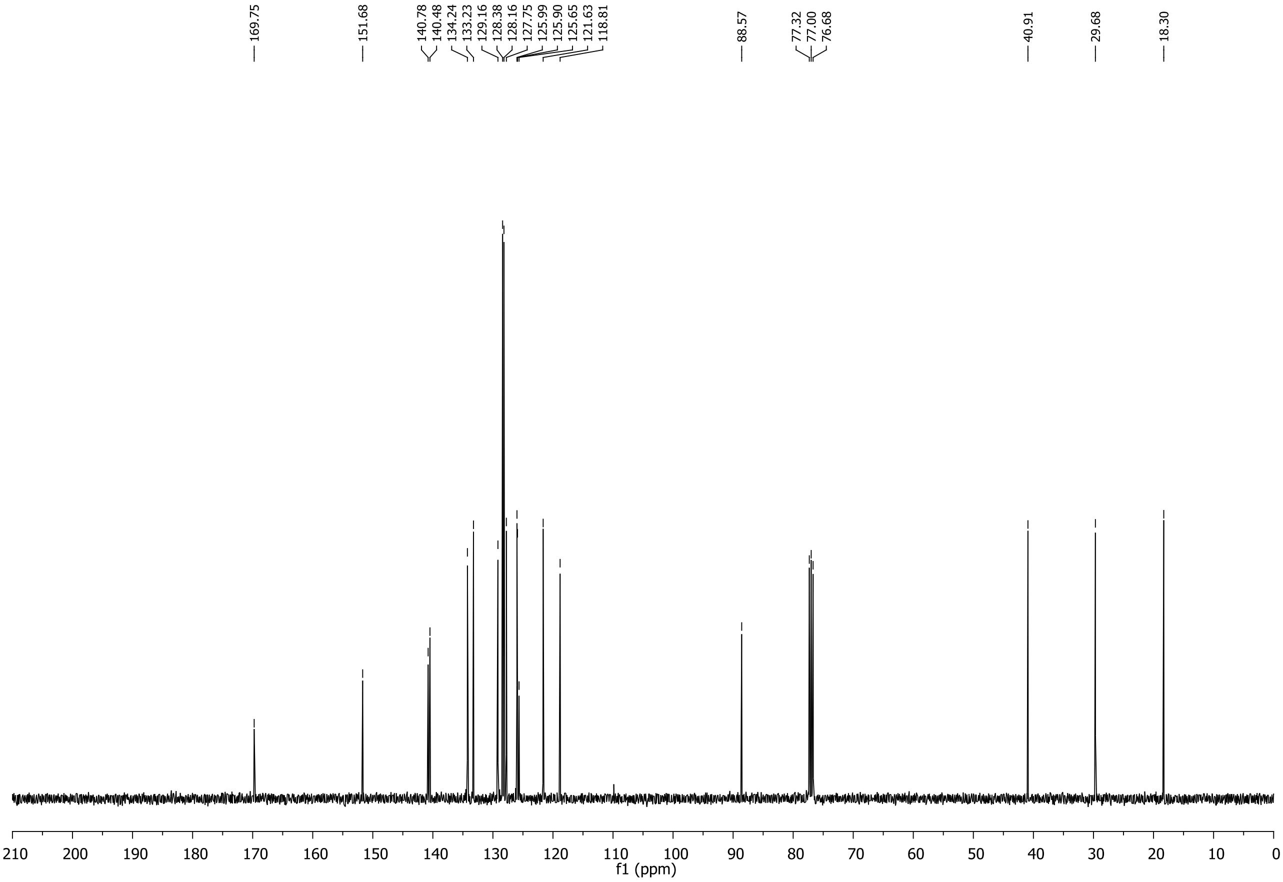
169.75
151.68
140.78
140.48
134.24
133.23
129.16
128.38
128.16
127.75
125.99
125.90
125.65
121.63
118.81
88.57
77.32
77.00
76.68
40.91
29.68
18.30
210 200 190 180 170 160 150 140 130 120 110 100 90 80 70 60 50 40 30 20 10 0
f1 (ppm)

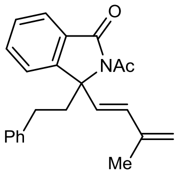
O
NAc
Ph
Me

**13e**

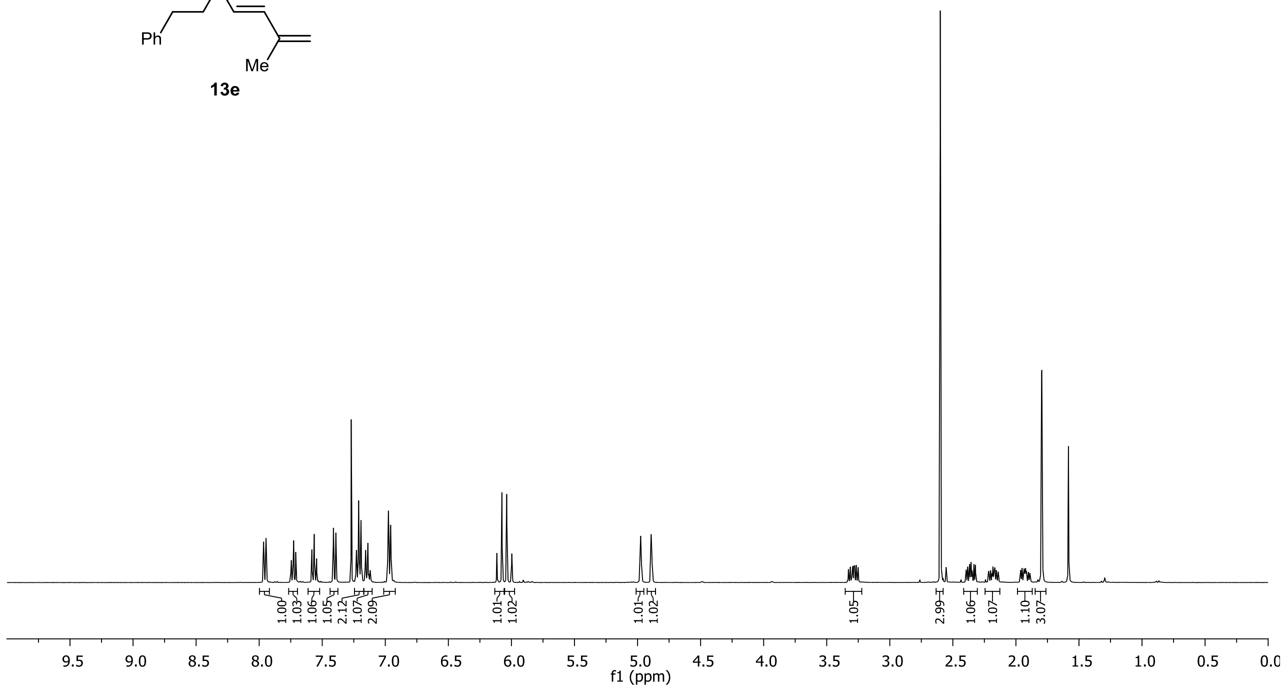
1.00
1.03
1.06
1.05
2.12
1.07
2.09
1.01
1.02
1.01
1.02
1.05
2.99
1.06
1.07
1.10
3.07
9.5 9.0 8.5 8.0 7.5 7.0 6.5 6.0 5.5 5.0 4.5 4.0 3.5 3.0 2.5 2.0 1.5 1.0 0.5
f1 (ppm)

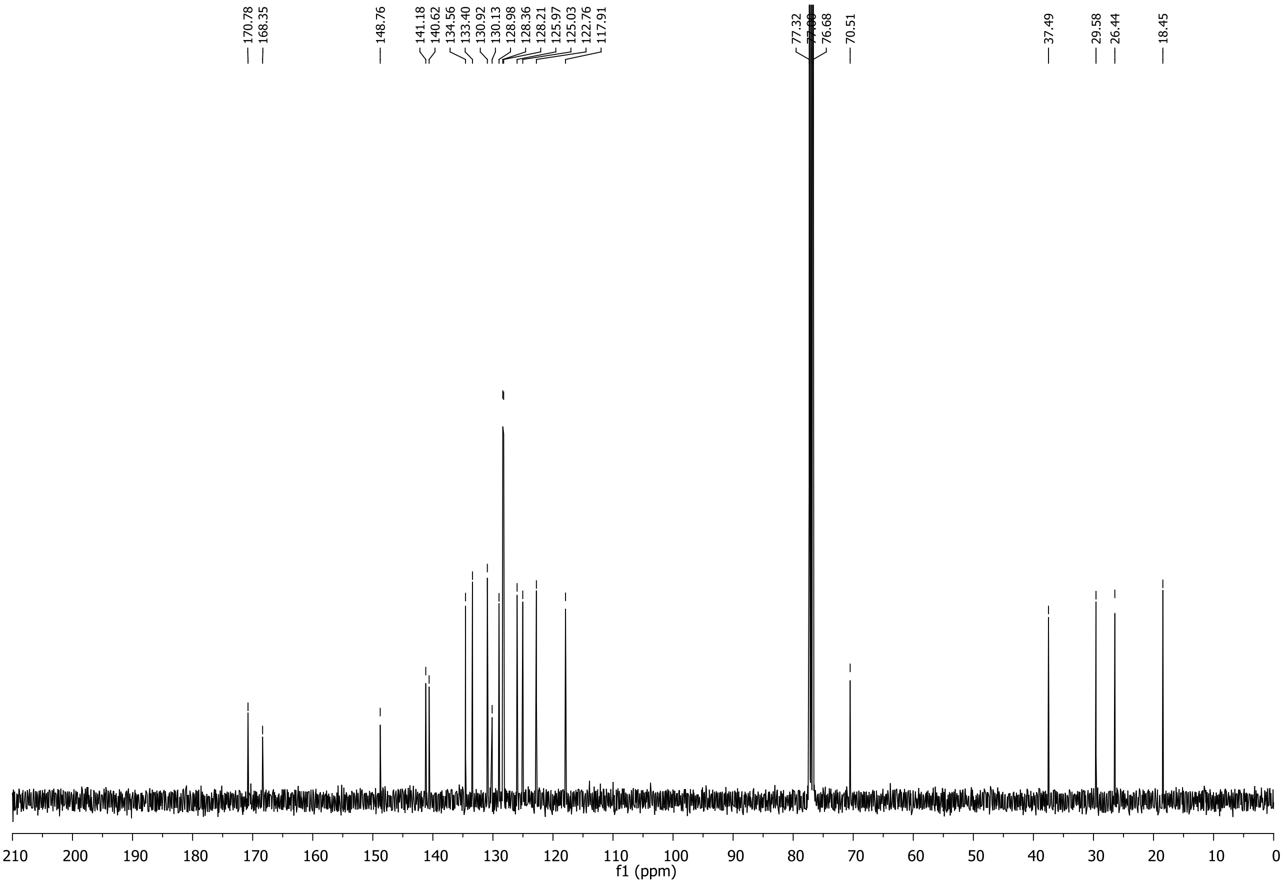
170.78
168.35
148.76
141.18
140.62
134.56
133.40
130.92
130.13
128.98
128.36
128.21
125.97
125.03
122.76
117.91
77.32
76.68
70.51
37.49
29.58
26.44
18.45
210 200 190 180 170 160 150 140 130 120 110 100 90 80 70 60 50 40 30 20 10 0
f1 (ppm)

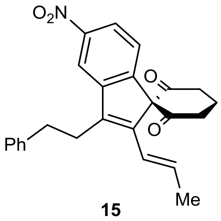
O2N
O
O
Ph
Me

**15**

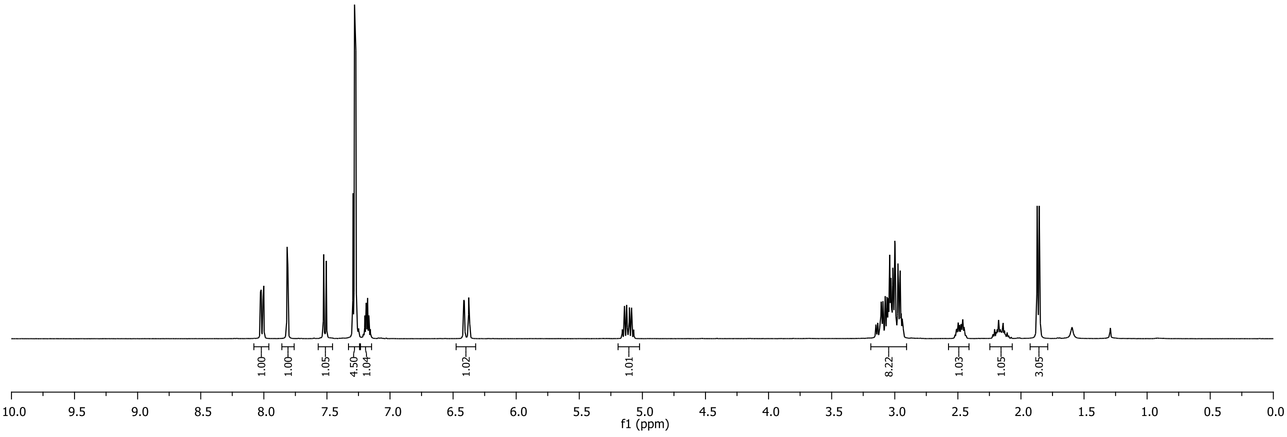
1.00
1.00
1.05
4.50
1.04
1.02
1.01
8.22
1.03
1.05
3.05
10.0 9.5 9.0 8.5 8.0 7.5 7.0 6.5 6.0 5.5 5.0 4.5 4.0 3.5 3.0 2.5 2.0 1.5 1.0 0.5 0.0
f1 (ppm)

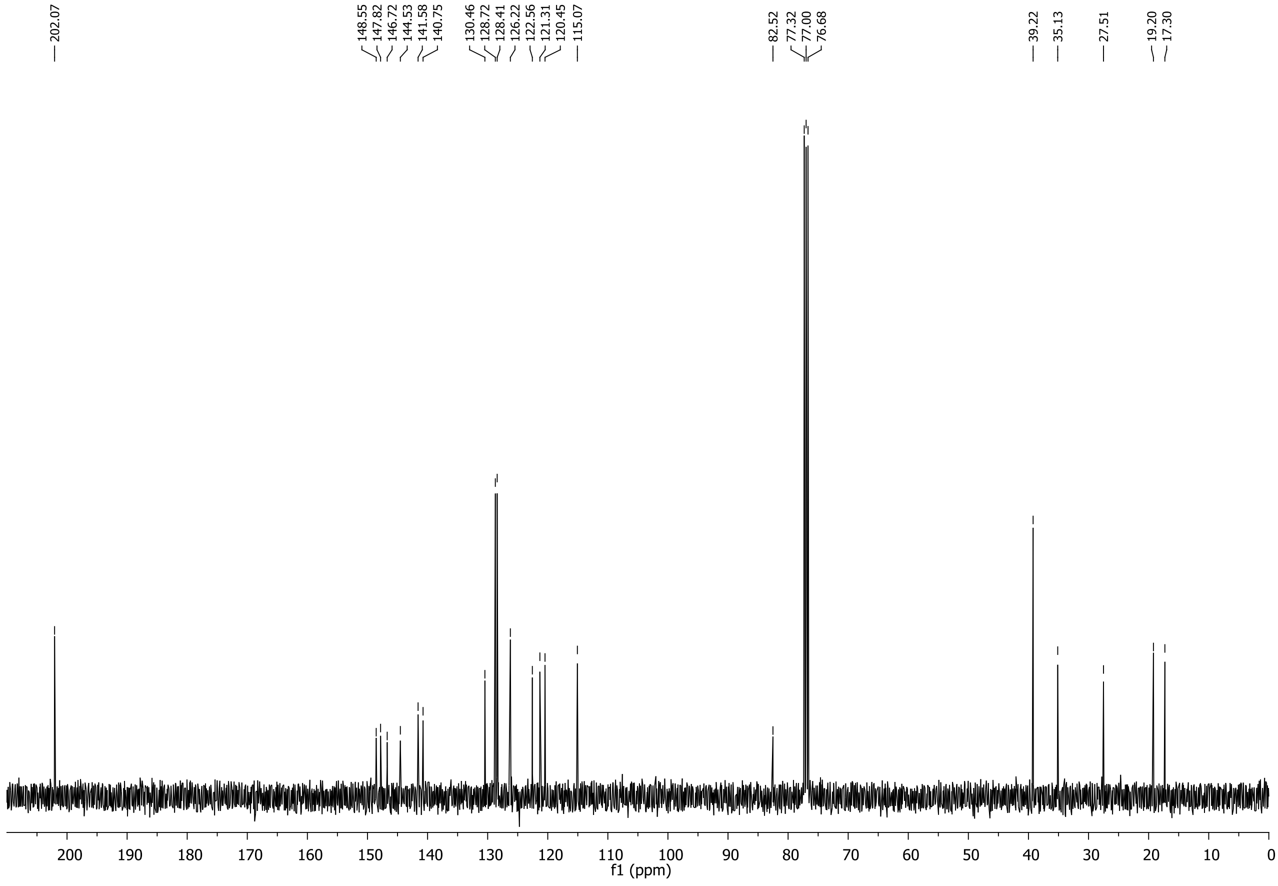
202.07
148.55
147.82
146.72
144.53
141.58
140.75
130.46
128.72
128.41
126.22
122.56
121.31
120.45
115.07
82.52
77.32
77.00
76.68
39.22
35.13
27.51
19.20
17.30
200 190 180 170 160 150 140 130 120 110 100 90 80 70 60 50 40 30 20 10 0
f1 (ppm)

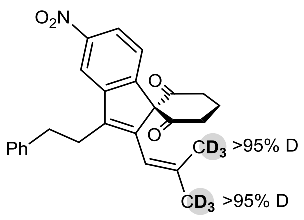
O2N
O
O
Ph
CD3 >95% D
CD3 >95% D

[D]$_6$-**3g**

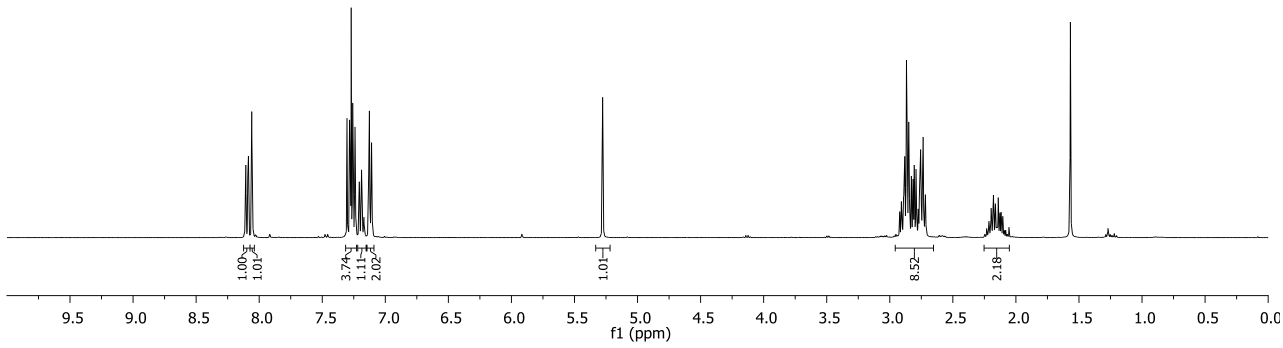
1.00
1.01
3.74
1.11
2.02
1.01
8.52
2.18
9.5 9.0 8.5 8.0 7.5 7.0 6.5 6.0 5.5 5.0 4.5 4.0 3.5 3.0 2.5 2.0 1.5 1.0 0.5 0.0
f1 (ppm)

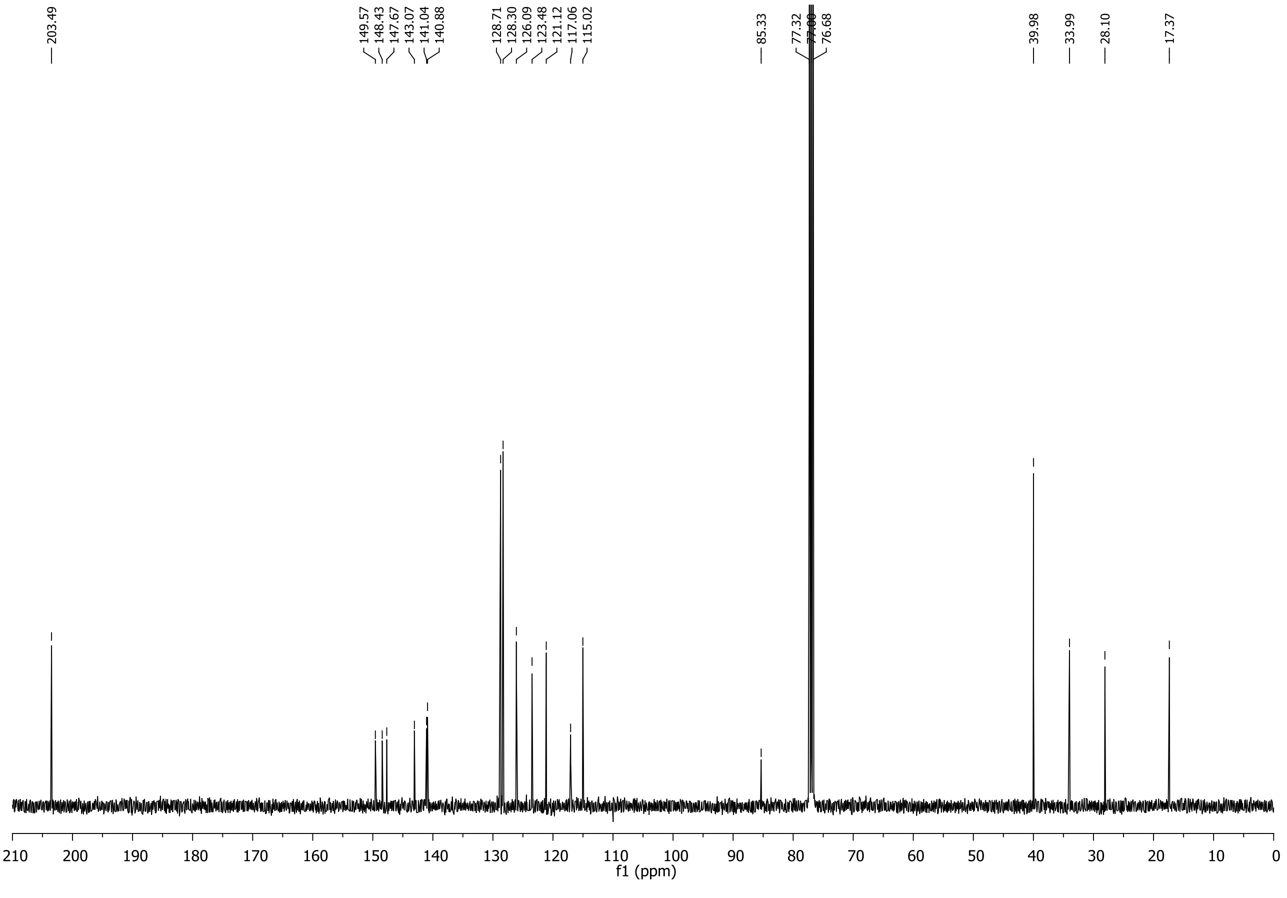
203.49
149.57
148.43
147.67
143.07
141.04
140.88
128.71
128.30
126.09
123.48
121.12
117.06
115.02
85.33
77.32
76.68
39.98
33.99
28.10
17.37
210 200 190 180 170 160 150 140 130 120 110 100 90 80 70 60 50 40 30 20 10 0
f1 (ppm)
